# 8-gingerol from *Zingiber officinale* induces ER stress-mediated ferroptosis and apoptosis in ovarian cancer involving TRPV1 activation

**DOI:** 10.1186/s12964-026-03014-8

**Published:** 2026-07-10

**Authors:** Tae Woo Kim

**Affiliations:** Department of Biopharmaceutical Engineering, Dongguk University-WISE, 123 Dongdae-ro, Gyeongju, 38066 Gyeongbuk Republic of Korea

**Keywords:** ER stress, 8-Gingerol, Ferroptosis, JMJD2C, TRPV1

## Abstract

**Background:**

Ovarian cancer remains among the most lethal gynecological malignancies, primarily due to the persistent challenges of chemotherapy resistance and limited effectiveness of existing therapeutic strategies.

**Methods:**

To evaluate the anti-tumor activity of 8-gingerol, its effects on cell viability, glutathione (GSH) levels, and the activities of caspase-3, lactate dehydrogenase (LDH), reactive oxygen species (ROS), and malondialdehyde (MDA) were examined in ovarian cancer cell lines. The underlying molecular mechanisms were investigated, involving the analysis of the TRPV1-PLCγ-NOX4 and PERK-ATF3-JMJD2C-SLC7A11-GPX4 signaling pathways. The functional role of ATF3 was further validated using gene knockdown approaches and promoter-binding assays. In addition, the *in vivo* therapeutic efficacy of 8-gingerol was assessed using a xenograft mouse model.

**Results:**

Treatment with 8-gingerol significantly reduced cell viability and intracellular GSH levels, while increasing caspase-3 activity, LDH release, ROS generation, and MDA levels. These molecular and cellular effects were associated with marked inhibition of tumor growth *in vivo*. Mechanistically, 8-gingerol induced intracellular Ca^2+^ accumulation and ROS production, thereby promoting endoplasmic reticulum (ER) stress-mediated apoptotic and ferroptotic cell death. This process was mediated in a manner involving activation of the TRPV1-PLCγ-NOX4 axis and ATF3-dependent suppression of SLC7A11 via the histone demethylase JMJD2C. Importantly, pharmacological inhibition of ROS or genetic targeting of ATF3 significantly attenuated 8-gingerol-induced cell death, confirming the critical functional roles of these signaling pathways.

**Conclusions:**

These findings demonstrate that 8-gingerol induces coordinated apoptotic and ferroptotic cell death through a Ca^2+^-dependent ER stress mechanism, highlighting its potential as a novel therapeutic strategy for the treatment of ovarian cancer.

**Graphical Abstract:**

**Figure:**
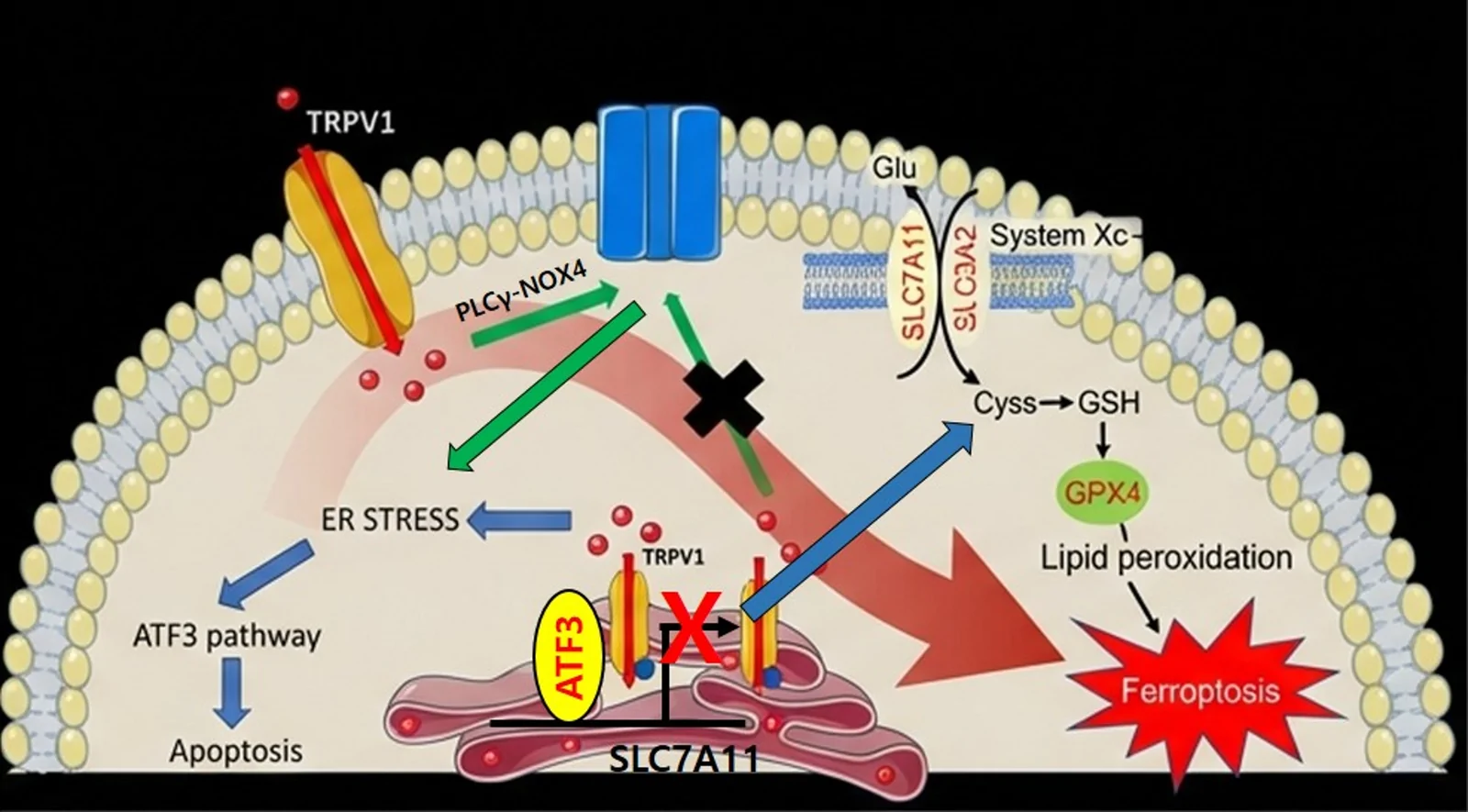

**Supplementary Information:**

The online version contains supplementary material available at https://doi.org/10.1186/s12964-026-03014-8.

## Introduction

Ovarian cancer is among the most aggressive malignancies affecting women, and the global incidence and mortality associated with this disease continue to rise. Current therapeutic strategies, including chemotherapy, radiotherapy, surgery, immunotherapy, and combination approaches, remain insufficient to effectively suppress tumor growth and progression [1]. Although chemotherapy represents the most widely used anti-tumor modality, the mechanisms underlying resistance to commonly used agents such as cisplatin, paclitaxel, and doxorubicin remain incompletely understood [2]. Consequently, there is a critical need to identify alternative therapeutic approaches, and natural products have emerged as promising candidates for reducing treatment-related toxicity and overcoming therapeutic resistance [3].

8-Gingerol, a bioactive phenolic compound derived from ginger (*Zingiber. officinale*), has been reported to exert immunomodulatory, anti-inflammatory, antioxidative, and anti-tumor effects [4]. Previous studies have demonstrated that 8-gingerol induces apoptosis by suppressing cell growth, proliferation, migration, and metastasis in multiple cancer types, including acute myeloid leukemia and cancer of the liver, prostate, and colon [5–8]. However, the role of 8-gingerol in ovarian cancer, particularly in the context of ER stress mediated signaling, has not been fully elucidated. Therefore, the present study aimed to define the molecular mechanisms underlying the anti-tumor effect of 8-gingerol and to establish a mechanistic framework for its potential application in overcoming therapeutic resistance in ovarian cancer.

The ER is a critical intracellular organelle involved in protein synthesis and the secretory pathway [9]. Disruption of ER protein-folding homeostasis activates an evolutionarily conserved stress response known as the unfolded protein response (UPR), which functions to restore cellular equilibrium [10]. The UPR is mediated through three principal signaling branches: inositol-requiring enzyme 1α (IRE1α), protein kinase RNA (PKR)-like ER kinase (PERK), and activating transcription factor 6 (ATF6) [11]. Under conditions of ER stress, PERK phosphorylates eukaryotic translation initiation factor 2α (eIF2α), leading to the activation of activating transcription factors 3 (ATF3) and ATF4. Activated ATF4 translocates to the nucleus, where it binds to the promoters of downstream UPR target genes, including growth arrest and DNA damage-inducible 34 (GADD34) and C/EBP homologous protein (CHOP), thereby promoting apoptotic cell death [12–14]. In parallel, ATF3 has been shown to bind to the promoter region of SLC7A11, suppressing its expression and thereby inducing ferroptotic cell death through inhibition of the SLC7A11-GPX4 axis [15]. Accumulating evidence indicates that ER stress mediated signaling pathways play a pivotal role in inducing tumor cell death and overcoming chemoresistance [16]. Recent studies further suggest that ginger-derived compounds, including 6-gingerol, 8-gingerol, 6-shogaol, and 8-shogaol, act as agonists of the transient receptor potential vanilloid 1 (TRPV1) channel [17]. TRPV1 is a nonselective cation channel with high permeability to Ca^2+^, and it contributes to the regulation of cell death, differentiation, and proliferation through oxidative stress-dependent mechanisms [18]. Activation of TRPV1 promotes intracellular Ca^2+^ influx, which subsequently activates phospholipase C (PLC), leading to hydrolysis of phosphatidylinositol 4,5-bisphosphate (PIP2) into diacylglycerol (DAG) and inositol triphosphate (IP3) [19]. DAG further activates protein kinase C (PKC), and activation of the PLC-PKC signaling axis has been implicated in the induction of ferroptosis through NOX4-mediated ROS production [20, 21].

Ferroptosis is a distinct form of regulated cell death characterized by iron-dependent lipid peroxidation and is driven by glutathione depletion, iron overload, and excessive ROS accumulation [22]. Increased lipid peroxidation and intracellular iron levels are key determinants of ferroptotic cell death, although these processes are tightly regulated by cellular antioxidant and pro-oxidant systems, as well as multiple regulatory proteins involved in lipid and iron metabolism [23, 24]. NADPH oxidases (NOXs) contribute to ROS generation required for lipid peroxidation during ferroptosis [25]. Glutathione peroxidase 4 (GPX4) serves as a central inhibitor of ferroptosis by utilizing GSH to prevent lipid peroxidation [26]. Accordingly, impairment of GPX4 activity promotes ferroptotic cell death through the accumulation of lipid peroxides [27]. Solute carrier family 7 member 11 (SLC7A11), which functions upstream of GPX4, plays a critical role in maintaining redox homeostasis, and its inhibition induces ferroptosis by suppressing GPX4 activity [28]. Among ER stress-associated transcription factors, ATF3 directly binds to the SLC7A11 promoter and represses its expression, thereby promoting ferroptosis through modulation of NOX4 and GPX4 [29]. In addition, p53 has been identified as a key regulator of ferroptosis through its interaction with the *SLC7A11* promoter [30]. Members of the Jumonji C domain-containing (JMJD) protein family are also involved in the regulation of diverse signaling pathways in inflammation and cancer [31]. For example, lysine-specific demethylase 6 A (KDM6A) regulates histone modifications at the MDM2 promoter, leading to increased MDM2 expression and reduced p53 levels, whereas inhibition of JMJD2C has been shown to enhance p53 expression [32]. Collectively, these findings highlight the ATF3-JMJD2C-SLC7A11-GPX4 axis as a critical regulatory pathway governing ferroptotic cell death [33].

In the present study, we demonstrate that 8-gingerol induces both apoptotic and ferroptotic cell death in ovarian cancer cells involving the TRPV1-PLCγ-NOX4 and ATF3-SLC7A11-GPX4 signaling pathways. Accordingly, this study aimed to define the mechanistic basis of 8-gingerol-mediated anti-tumor activity and to establish its potential as a novel therapeutic strategy for the treatment of ovarian cancer.

## Materials and methods

### Reagents

8-Gingerol (G5923), Z-VAD-FMK (627610), lipopolysaccharide (LPS; L4391), N-Acetyl-L-cysteine (NAC; A9165), diphenyleneiodonium chloride (DPI; D2926), Liproxstatin-1 (SML1414), erastin (326900), and SD-70 (5.31662) were obtained from Sigma-Aldrich (St. Louis, MO, USA). Thapsigargin (TG; T9033) was purchased from Millipore (Bedford, MA, USA).

### Cell cultures

Raw264.7 and J774.1 cells, murine macrophage cell lines, were obtained from the American Type Culture Collection (ATCC; Rockville, MD, United States) and maintained in Dulbecco Modified Eagle Medium (DMEM; Gibco, NY, United States) supplemented with 10% fetal bovine serum (FBS; HyClone, Logan, UT, United States) and streptomycin-penicillin (100 µg/mL streptomycin and 100 I.U./mL penicillin; Gibco-BRL). Human ovarian cancer cell lines (Caov-3, OVCAR-3, and A2780) were obtained from the Korean Cell Line Bank (Cancer Research Center, Seoul National University, Seoul, Korea). These cells were cultured in Roswell Park Memorial Institute 1640 (RPMI-1640) and DMEM media (Welgene, Daegu, Korea) supplemented with 10% inactivated FBS (HyClone, Logan, UT, United States) and streptomycin-penicillin (100 µg/mL streptomycin and 100 I.U./mL penicillin; Gibco-BRL). All cells were maintained at 37℃ in a humidified incubator containing 5% CO_2_.

### Cytokine measurements

Raw264.7 cells (1 × 10^4^ cells/well) were seeded into 96-well plates containing growth medium. To assess cytokine production in the conditioned medium, the cells were treated with LPS (1 µg/mL) in the absence or presence of 8-gingerol (0, 25, 50, and 100 µM) for 24 h, followed by enzyme-linked immunosorbent assay (ELISA). The levels of TNF-α, IL-6, and IL-1β were quantified using ELISA kits TNF-α (DY-410; R&D Systems), IL-6 (DY-406; R&D Systems), and IL-1β (DY-401; R&D Systems) according to the manufacturers’ protocols.

### Cell viability and proliferation assay

The effects of 8-gingerol on cell viability were evaluated using a WST-1 assay (Roche Applied Science, Indianapolis, IN, USA). Ovarian cancer cells (1 × 10^4^ cells/well) were seeded in 96-well plates and treated with 8-gingerol (0, 25, 50, and 100 µM) for 24 h. Subsequently, 10 µL of WST-1 reagent was added to each well, and cells were incubated for 1 h at 37℃. The conversion of WST-1 into formazan was quantified by measuring absorbance at 450 nm using a microplate reader (Molecular Devices, CA, USA), following the manufacturer’s instructions.

### LDH cytotoxicity assay

The cytotoxic effects of 8-gingerol were assessed using an LDH assay (Thermo Scientific, Waltham, MA, USA). Ovarian cancer cells (1 × 10^4^ cells/well) were seeded in 96-well plates and cultured in growth medium. LDH activity in the culture supernatants was measured by adding 100 µL of reaction mixture and incubating for 30 min in the dark. Absorbance was measured at 490 nm using a microplate reader (Molecular Devices, CA, USA), according to the manufacturer’s instructions.

### Caspase-3 colorimetric activity assay

Caspase-3 activity was evaluated in A2780 and OVCAR-3 cells following 8-gingerol treatment at the indicated time points. Cells (1 × 10^4^ cells/well) were seeded in 96-well plates and cultured under standard conditions. Cell lysates (50 µg protein) were incubated with reagents from a caspase-3 colorimetric activity assay kit (Abcam, Milpitas, CA, USA) to determine relative enzymatic activity. Absorbance was measured at 405 nm using a microplate reader (Molecular Devices, CA, USA), in accordance with the manufacturer’s protocol.

### RNA interference for knockdown experiment

A2780 and OVCAR-3 cells were seeded into 6-well plates (4 × 10^5^ cells/well) and incubated overnight in growth medium. Cells were subsequently washed with phosphate-buffered saline (PBS) and incubated in serum-free medium for 4 h. Double-stranded siRNAs (30 nmol/mL; Santa Cruz) targeting GRP78 (Santa Cruz; sc-29338) were transfected using Lipofectamine 2000 reagent (Invitrogen, Carlsbad, CA, USA), according to the manufacturer’s instructions.

### Transient and stable knockdown cell lines

For the generation of knockdown cell lines, shRNA lentiviral vectors targeting PERK (TRCN0000001400), CHOP (TRCN0000007264), TRPV1 (TRCN0000044188), JMJD2C (TRCN0000022054), and Nox4 (TRCN0000046088) (Sigma-Aldrich, Mission shRNA Plasmid DNA) were used. Purified shRNA-containing plasmids were transfected into Lenti-X 293T cells (Clontech, USA) using Lipofectamine 3000 (Invitrogen) to produce lentiviral particles. These viral particles were subsequently used to infect A2780 and OVCAR-3 cells following the manufacturer’s protocols (Clontech, USA). After 24 h of infection, stable knockdown cell lines were selected and maintained using puromycin (4 µg/mL; Sigma-Aldrich, USA).

### Quantitative real-time polymerase chain reaction

Total RNA was extracted from cultured cells (2 × 10^6^ cells/well) using TRIzol RNA isolation reagent, according to the manufacturer’s instructions (Invitrogen, Carlsbad, CA, USA). Complementary DNA (cDNA) was synthesized from 10 µg of total RNA using a reverse transcription kit (Power cDNA Synthesis Kit; iNTRON, Seongnam, Republic of Korea). Quantitative real-time PCR was performed using ABI Power SYBR Green PCR Master Mix (Applied Biosystems, Foster City, CA, USA), following the manufacturer’s protocol. The primer sequences used were as follows: JMJD2C (F) 5′-GAGGACTTGCGGAAGAAAGC-3′, JMJD2C (R) 5′- GAAATTGCATCAGCCCGTCC-3′; CHOP (F) 5′-ATGAGGACCTGCAAGAGGTCC-3′; CHOP (R) 5′- TCCTCCTCAGTCAGCCAAGC-3′; ATF3 (F) 5′- CGCTGGAATCAGTCACTGTCAG-3′; ATF3 (R) 5′- CTTGTTTCGGCACTTTGCAGCTG-3′; IL-6 (F) 5′- CTGATGCTGGTGACAACCAC-3′; IL-6 (R) 5′-TCCACGATTTCCCAGAGAAC-3′; IL-1β (F) 5′- GAGTGTGGATCCCAAGCAAT-3′; IL-1β (R) 5′- CTTGTGCTCTGCTTGTGAGG-3′; TNF-α (F), 5′-ACGGCATGGATCTCAAAGAC-3′; TNF-α (R), 5′-TGAGATAGCAAATCGGCTGAC-3′; COX-2 (F) 5′- CCACTTCAAGGGAGTCTGGA-3′; COX-2 (R) 5′-AGTCATCTGCTACGGGAGGA-3′. Quantitative real-time PCR was conducted using a Roche LightCycler 96 System (Roche, Mannheim, Germany). Relative mRNA expression levels were normalized to β-actin (F) 5′-AAGGCCAACCGCGAGAAGAT-3′; β-actin (R) 5′-TGATGACCTGGCCGTCAGG-3′ and calculated using the 2^−ΔΔCt^ method.

### Western blotting analyses

Cells (2 × 10^6^ cells/well) were lysed in radioimmunoprecipitation assay (RIPA) buffer (Biosesang, Inc., Seoul, Korea) supplemented with a protease inhibitor cocktail (Sigma-Aldrich, St. Louis, MO, USA) and incubated on ice for 30 min. Lysates were passed through an 18-gauge needle and centrifuged to remove debris. Protein concentrations were determined using a BCA protein assay kit (Thermo Fisher Scientific, Waltham, MA, USA). Equal amounts of total protein (20 µg) from A2780 and OVCAR-3 cells were separated by 8–15% SDS-PAGE and transferred onto nitrocellulose membranes (Millipore Corporation, Billerica, MA, USA). Membranes were incubated with primary antibodies (1:1000) against eIF2α (Santa Cruz; sc-133132), GPX4 (Santa Cruz; sc-166570), JMJD2C (Santa Cruz; sc-515767), NRF2 (Santa Cruz; sc-365949), KEAP1 (Santa Cruz; sc-515432), HO-1 (Santa Cruz; sc-136960), ATF3 (Santa Cruz; sc-518032), β-actin (Santa Cruz; sc-47778), H3 (Santa Cruz; sc-517576), CD63 (Proteintech; 25682-1-AP), Nox4 (Proteintech; 14347-1-AP), ATF3 (Proteintech; 15755-1-AP), PKCα (Abcam; ab32376), p-PKCα (T514) (Abcam; ab109539), TRPV1 (Novus; NBP1-97417), H3K4me1 (Cell Signaling; #5326), H3K4me2 (Cell Signaling; #9725), H3K4me3 (Cell Signaling; #9751), H3K9me1 (Abcam; ab8896), H3K9me2 (Cell Signaling; #4658), H3K9me3 (Cell Signaling; #13969), H3K27me1 (Cell Signaling; #84932), H3K27me2 (Cell Signaling; #9728), H3K27me3 (Cell Signaling; #9733), H3K36me1 (Cell Signaling; #14111), H3K36me2 (Cell Signaling; #2901), H3K36me3 (Cell Signaling; #4909), p-eIF2α (Cell Signaling; #3398), CHOP (Cell Signaling; #2895), SLC7A11 (Cell Signaling; #12691), PUMA (Cell Signaling; #98672), p-PERK (Cell Signaling; #12185), GRP78 (Cell Signaling; #3177), p-PLCγ1 (Tyr783) (Cell Signaling; #2821), PLCγ1 (Cell Signaling; #2822), cleaved caspase-3 (Cell Signaling; #9661), PERK (Cell Signaling; #5683), Bcl-2 (Cell Signaling; #2876), p-NF-kB (Cell Signaling; #3031), NF-κB (Cell Signaling; #3034), TNFα (Cell Signaling; #11948), IL-1β (Cell Signaling; #12703), IL-6 (Cell Signaling; #12912), and cleaved caspase-9 (Cell Signaling; #20750). After incubation with horseradish peroxidase-conjugated secondary antibodies (Santa Cruz; 1:6000; sc-2357, sc-358914), signals were detected using the D-Plus enhanced chemiluminescence Pico System (DonginLS, Korea, ECL-PS100), following the manufacturer’s instructions.

### Exosomes isolation from the cell culture media

A2780 and OVCAR-3 cells were seeded into 100 mm culture dishes (2 × 10^6^ cells/well) and incubated overnight in culture medium. Exosomes were isolated from conditioned media 8-gingerol (0, 8, 16, and 24 h; 50 µM) using a Total Exosome Isolation Reagent (Thermo Scientific, CA, USA), according to the manufacturer’s protocol.

### Intracellular Ca^2+^ assays

Intracellular Ca^2+^ levels in A2780 and OVCAR-3 cells were assessed using a Ca^2+^ assay kit (Abcam, Cambridge, MA, USA). Cells (1 × 10^4^ cells/well) were seeded in 96-well plates and cultured for 24 h. Following treatment with 8-gingerol for 24 h, and the chromogenic reagent and assay buffer were added, and samples were incubated for 10 min at room temperature. Ca^2+^ activity was measured using a colorimetric assay (Abcam), according to the manufacturer’s instructions.

### ROS detection cell-based assays

Cells (1 × 10^4^ cells/well) were seeded in 96-well plates and cultured for 24 h. Cells were then treated with 8-gingerol (0, 8, 16, and 24 h; 50 µM), with DPI (1 µM) and NAC (100 µM) used as negative controls. Cells were incubated with the cell-permeant dye 2’7’-dichlorodihydrofluorescein diacetate (CM-H_2_DCFDA; Invitrogen) for 30 min at 37 °C, according to the manufacturer’s instructions. Fluorescence intensity was measured at excitation/emission wavelengths of 495/525 nm using a microplate reader (Molecular Devices, USA).

### Malondialdehyde (MDA) colorimetric assays

Lipid peroxidation was evaluated by measuring the MDA levels in cell lysates using a lipid peroxidation (MDA) assay kit (MAK085; Sigma-Aldrich, St. Louis, MO, USA), according to the manufacturer’s instructions.

### Glutathione (GSH) assays

Glutathione levels in cell lysates were measured using a reduced glutathione (GSH) assay kit (MAK364; Sigma-Aldrich, St. Louis, MO, USA), according to the manufacturer’s instructions.

### Glutathione peroxidase 4 (GPX4) activity assays

The effects of 8-gingerol on GPX4 activity in A2780 and OVCAR-3 cells were evaluated at the indicated time points using a GPX4 activity assay kit (Elabscience, Houston, TX, USA). A2780 and OVCAR-3 cells (1 × 10^4^ cells/well) were seeded in a 96-well plates, cultured in growth medium, and incubated for 24 h. GPX4 enzymatic activity was subsequently measured in accordance with the manufacturer’s instructions.

### Measurement of the labile iron pool

The intracellular labile iron pool (LIP) was quantified by determining ferrous iron (Fe^2+^) concentrations using a colorimetric Iron Assay Kit (ab83366; Abcam, Cambridge, UK), according to the manufacturer’s protocol. A2780 and OVCAR-3 cells (1 × 10^4^ cells/well) were seeded in 96-well plates, cultured in growth medium, and incubated for 24 h. Cells were lysed in the provided assay buffer, and supernatants were reacted with an iron probe to generate a colored complex. Absorbance was measured at 593 nm using a microplate reader (Molecular Devices, CA, USA).

### Co-immunoprecipitation (Co-IP)

Co-immunoprecipitation assays were performed as previously described [34]. Cell lysates were prepared from A2780 and OVCAR-3 cells cultured in 100-mm dishes using IP lysis buffer (20–188; Sigma-Aldrich, St. Louis, MO, USA). Lysates were incubated with anti-SLC7A11 (Cell Signaling; #12691), anti-ATF3 (Santa Cruz; sc-518032), and anti-JMJD2C (Santa Cruz; sc-515767) antibodies at 4℃ for 24 h. Immune complexes were subsequently captured using protein A/G PLUS agarose (Santa Cruz).

### Chromatin immunoprecipitation(ChIP) assays

This assay was performed as described previously [35]. ChIP assays were conducted as previously described using an EZ ChIP Chromatin Immunoprecipitation Kit (Millipore, Billerica, MA, USA), following the manufacturer’s instructions. Cross-linked chromatin was sonicated after cell lysis and incubated overnight at 4 °C with antibodies against ATF3 (Santa Cruz; sc-518032) and JMJD2C (Santa Cruz; sc-515767). Immunocomplexes were precipitated using protein A–agarose (Millipore). PCR primers [5′-GAAGTGGCTATCGCCAGAGT-3′ (sense) and 5′- GCTGCTTGAAGGTCTTCTCC − 3′ (antisense)] were used to amplify the ATF3 and JMJD2C binding regions within the *SLC7A11* promoter. Quantitative PCR was performed under the following conditions: 40 cycles of 94 °C for 40 s, 60 °C for 1 min, and 72 °C for 40 s.

### Animal experiments

For *in vivo* studies, five-week-old female athymic BALB/c nude mice (*nu/nu*) were obtained from Jung-Ang Lab Animal, Inc. (Seoul, Korea) and acclimated for one week with ad libitum access to sterile standard chow (Rodent NIH-07 open formula) and water. All procedures were conducted in accordance with National Institutes of Health guidelines and were approved by the Institutional Animal Care and Use Committee of Kyung Hee University (KHSASP-20-250; August 24, 2020). Mice were randomly assigned to three groups: control, 10 mg/kg 8-gingerol, and 20 mg/kg 8-gingerol (*n* = 10 per group). A2780 cells (1 × 10^7^) were subcutaneously injected into the right flanks of mice. Upon tumor establishment, 8-gingerol was administered via intraperitoneal injection every other day. Tumor volume was measured three times per week along two axes (*L*, longest axis; *W*, shortest axis) and calculated using the formula: tumor volume = (*L* × *W*^2^) / 2. For assessment of inflammatory responses, mice were divided into three groups (PBS, LPS, and LPS + 8-gingerol), and inflammation was induced by intraperitoneal injection of LPS (20 mg/kg). 8-gingerol (10 mg/kg, dissolved in PBS) was administered intraperitoneally immediately following LPS injection in the treatment group. Mice were monitored for 12 days, after which blood and tissue samples were collected. For the septic shock model, cecal ligation and puncture (CLP) surgery was performed as previously described by Rittirsch et al. [36]. Following CLP surgery, mice received intraperitoneal injection of 8-gingerol (10 mg/kg). Survival rates were monitored and analyzed for 2 ~ 10 days after CLP and 2 ~ 12 days following LPS administration.

### Statistical analysis

Experimental data are presented as mean ± standard deviation (SD) from at least three independent experiments. Statistical analyses were performed using GraphPad Prism (version 9.0). Comparisons between two groups were conducted using an unpaired Student’s t-test. For experiments involving a single independent variable with multiple groups, one-way analysis of variance (ANOVA) followed by Tukey’s post hoc test was applied. For multi-factorial experiments incorporating both pharmacological treatment and genetic manipulation, two-way ANOVA followed by Sidak’s or Tukey’s multiple comparisons tests was used to evaluate main effects and interactions. A p-value < 0.05 was considered statistically significant.

## Results

### 8-Gingerol decreases LPS-induced pro-inflammatory cytokines in Raw264.7 and J774.1 cells

**Fig. 1:**
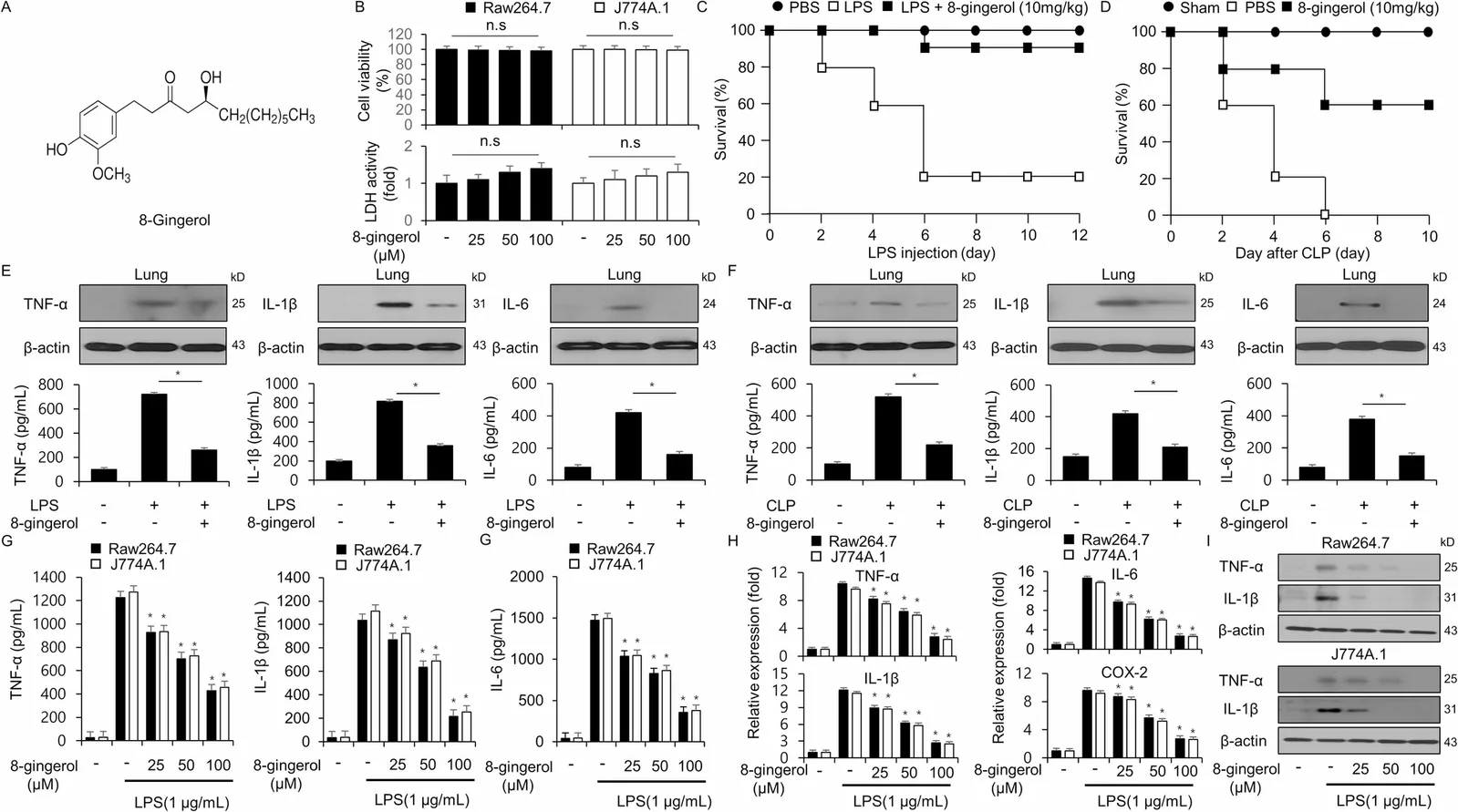
Effects of 8-gingerol on mRNA and protein expression levels of inflammatory response markers in inflammatory mouse models and LPS-treated Raw264.7 and J774.1 cells. (**A**) Chemical structure of 8-gingerol. (**B**) Cell viability and LDH cytotoxicity following treatment with 8-gingerol at the indicated concentrations (0, 25, 50, and 100 µM, 24 h). * *p* < 0.05; n.s., not significant. All experiments were performed in triplicate. (**C**) C57BL/6 mice were intraperitoneally (i.p.) injected with LPS (20 mg/kg) alone or in combination with 8-gingerol (10 mg/kg). Survival rates of the LPS alone and LPS + 8-gingerol groups (*n* = 10/group) were monitored daily for 12 days. (**D**) Following CLP surgery, mice were intraperitoneally injected with PBS or 8-gingerol (10 mg/kg), and survival rates were monitored daily for 5 days. (**E**,** F**) Protein expression levels of TNF-α, IL-6, and IL-1β in the lung tissues and serum samples from LPS- and CLP-induced mouse models treated with 8-gingerol. (**G-I**) Protein expression levels of TNF-α, IL-6, and IL-1β and mRNA expression levels of TNF-α, IL-6, COX-2, and IL-1β in LPS-treated (1 µg/mL) Raw264.7 and J774.1 cells in the presence or absence of 8-gingerol (0, 25, 50, and 100 µM, 24 h), as determined by ELISA, western blotting, and quantitative real-time PCR analyses. β-actin was used as an internal control for normalization of mRNA and protein expression levels. * *p* < 0.05; n.s., not significant. All experiments were performed in triplicate

Figure 1A illustrates the chemical structure of 8-gingerol. To initially evaluate its potential cytotoxicity effects in macrophage cell lines, cell viability and cytotoxicity assays were performed in Raw264.7 and J774.1 cells following 8-gingerol treatment. Cell viability remained largely unaffected, and no significant cytotoxicity was observed in a dose-dependent manner (Fig. 1B). To further investigate the anti-inflammatory properties of 8-gingerol, *in vivo* experiments were conducted using LPS-induced acute inflammatory and CLP-induced septic mouse models. Compared with the LPS-treated group, administration of 8-gingerol (10 mg/kg) increased the survival rate by approximately 4-fold in the LPS-induced inflammatory model (Fig. 1C). Similarly, intraperitoneal administration of 8-gingerol (10 mg/kg) following CLP surgery increased survival by approximately 6-fold (Fig. 1D). To further characterize the anti-inflammatory effects of 8-gingerol in LPS- and CLP-mediated inflammatory responses, the expression levels of inflammatory cytokines, including TNF-α, IL-6, and IL-1β, were evaluated in mouse tissue and serum samples. Treatment with 8-gingerol markedly reduced the protein expression levels of TNF-α, IL-6, and IL-1β in both tissue and serum obtained from LPS- and CLP-treated mice (Fig. 1E, F, Fig. S1A, B). Previous studies have demonstrated that naturally derived compounds suppress pro-inflammatory mediators, including TNF-α, IL-6, and IL-1β [37]. To determine whether 8-gingerol exerts anti-inflammatory effects in LPS-stimulated macrophages, real-time PCR, western blotting, and ELISA were performed. 8-Gingerol significantly reduced the secretion of IL-6, IL-1β, and TNF-α in LPS-treated Raw264.7 and J774.1 cells (Fig. 1G). Consistently, LPS treatment increased the mRNA expression levels of IL-6, IL-1β, COX-2, and TNF-α, whereas treatment with 8-gingerol markedly suppressed the expression of these inflammatory mediators in both Raw264.7 and J774.1 cells (Fig. 1H). Western blot analyses further demonstrated that 8-gingerol reduced the protein expression levels of IL-1β and TNF-α in LPS-treated Raw264.7 and J774.1 cells (Fig. 1I, Fig. S1C). Collectively, these findings indicate that 8-gingerol suppresses inflammatory cytokine production and inflammatory signaling in activated macrophages.

Given that chronic inflammation within the tumor microenvironment is a critical driver of ovarian cancer progression, metastasis, and therapeutic resistance, we next sought to determine whether the anti-inflammatory activity of 8-gingerol may also contribute to direct anti-tumor effects in ovarian cancer cells. Based on the anti-inflammatory findings presented in Fig. 1, we subsequently investigated the ability of 8-gingerol to induce apoptotic cell death and ER stress-associated signaling in ovarian cancer models.

### 8-Gingerol mediates apoptotic cell death in ovarian cancer cells

**Fig. 2:**
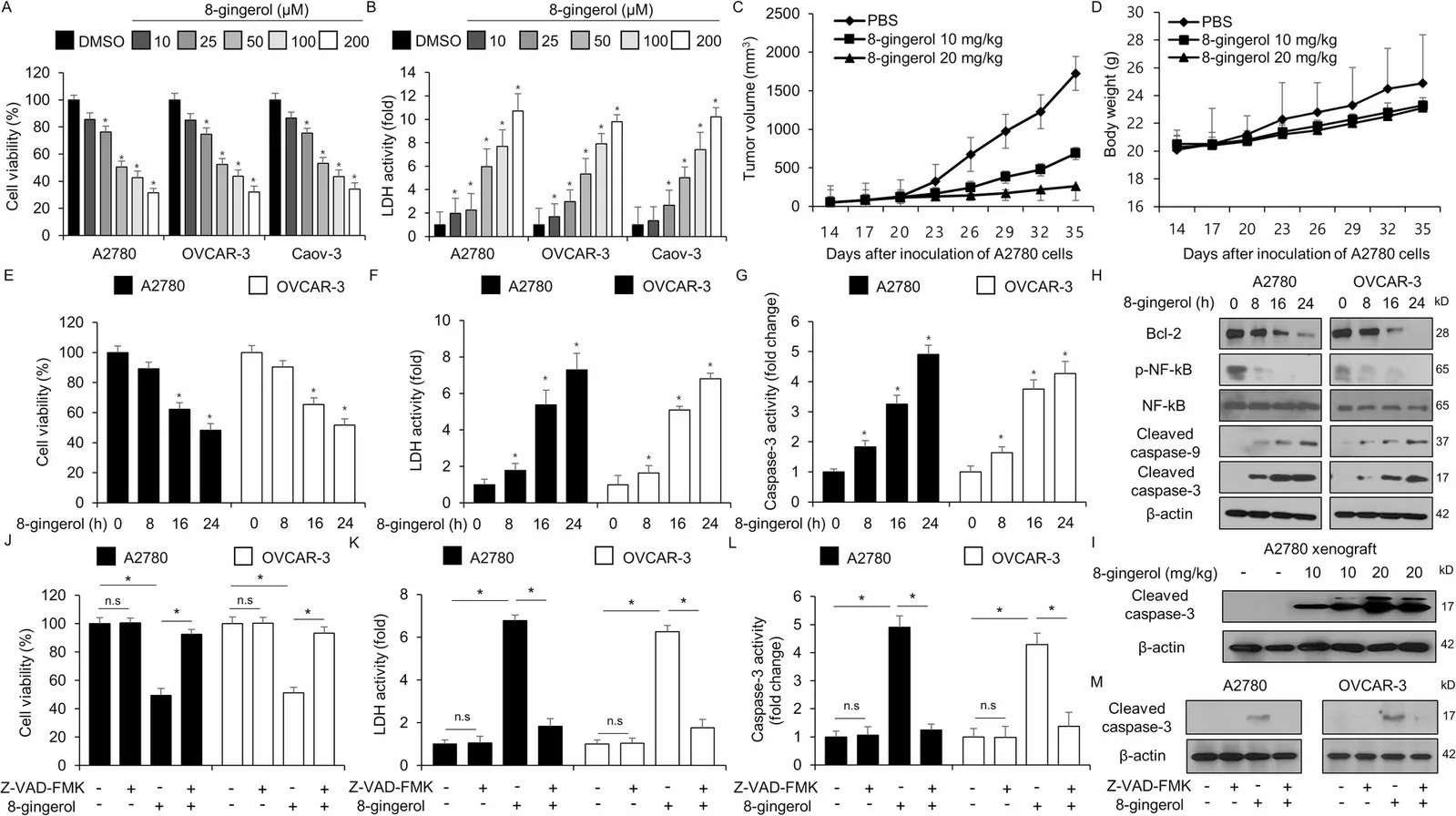
Anti-tumor effects of 8-gingerol in ovarian cancer *in vitro* and *in vivo*. (**A**,** B**) Cell viability and LDH cytotoxicity in ovarian cancer cell lines (A2780, OVCAR-3, and Caov-3) treated with 8-gingerol at the indicated concentrations (10, 25, 50, 100, and 200 µM, 24 h), as assessed by WST-1 and LDH cytotoxicity assays. DMSO-treated cells were normalized to 100%. * *p* < 0.05; n.s., not significant. All experiments were performed in triplicate. (**C**,** D)** Xenograft nude mice were randomly assigned to three groups (PBS, 10 mg/kg 8-gingerol, and 20 mg/kg 8-gignerol; *n* = 10/group) and subcutaneously implanted with 1 × 10^7^ A2780 cells. 8-Gingerol was intraperitoneally administered every 2 days. Tumor volume and body weight were measured twice weekly. (**E–H**) Cell viability, LDH cytotoxicity, and caspase-3 activity in ovarian cancer cell lines treated with 8-gingerol (50 µM) at the indicated time points (0, 8, 16, and 24 h), as determined by WST-1, LDH cytotoxicity, and caspase-3 activity assays. * *p* < 0.05; n.s., not significant. Protein expression levels of Bcl-2, p-NF-κB, NF-κB, cleaved caspase-9, and cleaved caspase-3 were analyzed in A2780 and OVCAR-3 cells treated with 8-gingerol in a time-dependent manner by western blotting. β-actin was used as a loading control. All experiments were performed in triplicate. (**I**) Western blot analysis of cleaved caspase-3 expression in tumor tissues obtained from mice treated with 8-gingerol (10 and 20 mg/kg). β-actin was used as a loading control. (J-M) Effects of the pan-caspase inhibitor Z-VAD-FMK (50 µM, 24 h) on 8-gingerol-induced apoptotic cell death. Ovarian cancer cell lines were pretreated with Z-VAD-FMK (50 µM, 4 h) followed by treatment with 8-gingerol (50 µM, 24 h). Cell viability, caspase-3 activity, and LDH cytotoxicity were evaluated using WST-1, caspase-3 activity, and LDH assays. * *p* < 0.05; n.s., not significant. Cleaved caspase-3 expression was analyzed by western blotting. β-actin was used as a loading control. All experiments were performed in triplicate

We next examined the anti-proliferative and cytotoxic effects of 8-gingerol in the human ovarian cancer cell lines A2780, OVCAR-3, and Caov-3 following treatment with increasing concentrations of 8-gingerol (10, 25, 50, 100, and 200 µM) for 24 h. WST-1 and lactate dehydrogenase (LDH) assays demonstrated that 8-Gingerol significantly reduced cell viability and increased cytotoxicity in all three ovarian cancer cell lines in a dose-dependent manner (Fig. 2A, B). As shown in Fig. 2A, the calculated IC_50_ values for 8-gingerol were 50.5 µM in A2780 cells and 52.4 µM in OVCAR-3 cells. Based on these findings, 50 µM 8-gingerol was selected for subsequent mechanistic analyses. To evaluate the anti-tumor activity of 8-gingerol *in vivo*, an A2780 xenograft mouse model was established. Mice treated with 8-gingerol (10 or 20 mg/kg) exhibited significantly reduced tumor volumes compared with control mice (Fig. 2C). In contrast, no significant differences in body weight were observed among the experimental groups (Fig. 2D), suggesting limited systemic toxicity under the treatment conditions used. To further characterize the cytotoxic effects of 8-gingerol over time, A2780 and OVCAR-3 cells were treated with 8-gingerol (50 µM) for 0, 8, 16, and 24 h, followed by WST-1, caspase-3 activity, and LDH assays. Treatment with 8-gingerol progressively reduced cell viability and significantly increased LDH release and caspase-3 activity in a time-dependent manner in both A2780 and OVCAR-3 cells (Fig. 2E-G). To investigate whether 8-gingerol-induced cytotoxicity was associated with caspase-dependent apoptosis, western blot analyses were subsequently performed. Treatment with 8-gingerol markedly increased the cleavage of caspase-3 and caspase-9, while simultaneously reducing the expression levels of Bcl-2 and phosphorylated-NF-κB in a time-dependent manner (0, 8, 16, and 24 h; 50 µM) (Fig. 2H, Fig. S2A). Consistent with the *in vitro* findings, tumor tissues isolated from mice treated with 8-gingerol (10 and 20 mg/kg) exhibited higher levels of cleaved caspase-3 expression than tissues obtained from control mice (Fig. 2I, Fig. S2B). To further determine whether 8-gingerol-induced cytotoxicity was mediated through caspase activation, A2780 and OVCAR-3 cells were co-treated with 8-gingerol (50 µM, 24 h) and the pan-caspase inhibitor Z-VAD-FMK (50 µM, 24 h). Inhibition of caspase activity significantly restored cell viability and reduced caspase-3 and LDH activities in 8-gingerol-treated cells (Fig. 2J-L). Moreover, co-treatment with Z-VAD-FMK markedly attenuated 8-gingerol-induced caspase-3 cleavage (Fig. 2M, Fig. S2C). These findings indicate that 8-gingerol induces caspase-dependent apoptotic cell death in ovarian cancer cells. Furthermore, to determine whether 8-gingerol could overcome radioresistance, WST-1 assays were performed using radio-resistant ovarian cancer cell lines, A2780R and OVCAR-3R. In both radio-resistant cell lines, 8-gingerol significantly reduced cell viability, and combination treatment with radiation (2 Gy) further enhanced this effect. In contrast, radiation treatment alone (2 Gy) did not significantly alter the viability of A2780R or OVCAR-3R cells (Fig. S2D).

### 8-Gingerol mediates apoptotic cell death through the ER stress pathway in ovarian cancer cells

**Fig. 3:**
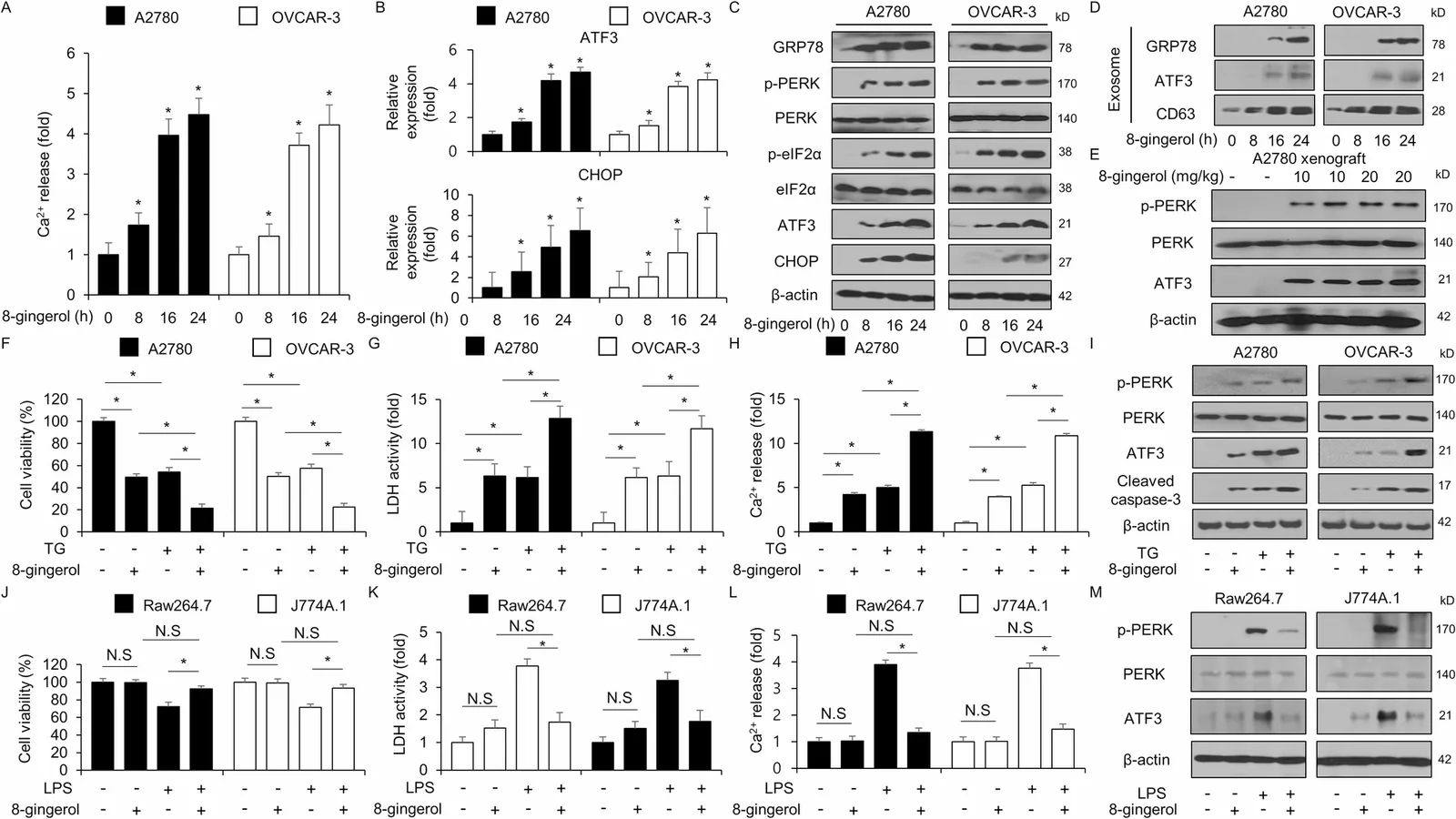
8-Gingerol induces ER stress-mediated cell death through intracellular Ca^2+^ release in ovarian cancer cell lines and macrophages. (**A**) A2780 and OVCAR-3 cells were treated with 8-gingerol (50 µM) at the indicated time points, and intracellular Ca^2+^ levels were analyzed. * *p* < 0.05; n.s., not significant. (**B**) mRNA expression levels of CHOP and ATF3 were analyzed by quantitative real-time PCR. β-actin was used as an internal control for normalization. (**C**) Protein expression levels of eIF2α, p-PERK, CHOP, GRP78, ATF3, p-eIF2α, and PERK were analyzed by western blotting. β-actin was used as a loading control. (**D**) Ovarian cancer cell lines were treated with 8-gingerol (50 µM) under the indicated conditions, and exosomes (30 µg) were isolated from conditioned culture media. Expression levels of the exosomal marker CD63 and the ER stress-related proteins GRP78 and ATF3 were analyzed in exosome fractions by western blotting. (**E**) Western blot analysis of p-PERK, PERK, and ATF3 expression in tumor tissues obtained from mice treated with 8-gingerol (10 and 20 mg/kg). β-actin was used as a loading control. (**F–I**) Intracellular Ca^2+^ levels, LDH cytotoxicity, and cell viability were evaluated in A2780 and OVCAR-3 cells treated with thapsigargin (TG; 3 µM, 24 h) and 8-gingerol (50 µM, 24 h). Expression levels of ER stress-related proteins, including ATF3, cleaved caspase-3, p-PERK, and PERK, were analyzed by western blotting. * *p* < 0.05; n.s., not significant. β-actin was used as a loading control. All experiments were performed in triplicate. (**J–M**) Raw264.7 and J774.1 cells were treated with 8-gingerol (50 µM, 24 h), LPS (1 µg/mL), or a combination of LPS and 8-gingerol. Cell viability, intracellular Ca^2+^ levels, LDH cytotoxicity, and protein expression levels were analyzed using WST-1, intracellular Ca^2+^ assays, LDH assays, and western blotting. * *p* < 0.05; n.s., not significant. Protein expression levels of ATF3, p-PERK, and PERK were analyzed by western blotting. β-actin was used as a loading control. All experiments were performed in triplicate

Previous studies have demonstrated that ER stress induces UPR-mediated apoptosis and cell death in multiple cancer cell types [38, 39]. To determine whether 8-gingerol activates ER stress signaling and the UPR in ovarian cancer models, intracellular Ca^2+^ release assays were performed in A2780 and OVCAR-3 cells. Treatment with 8-gingerol induced intracellular Ca^2+^ release in a time-dependent manner (Fig. 3A). To further examine the transcriptional activation of ER stress-associated genes, quantitative real-time PCR analyses were conducted to evaluate CHOP and ATF3 expression. The mRNA expression levels of CHOP and ATF3 were significantly increased in A2780 and OVCAR-3 cells following 8-gingerol treatment at the indicated time points (Fig. 3B). To further characterize the ER stress response induced by 8-gingerol, western blot analyses were performed to evaluate the expression levels of key ER stress-associated proteins, including eIF2α, PERK, CHOP, phosphorylated eIF2α, phosphorylated PERK, ATF3, and GRP78. Treatment with 8-gingerol markedly increased GRP78 and enhanced phosphorylation of eIF2α and PERK. In addition, 8-gingerol significantly upregulated ATF3 and CHOP expression in ovarian cancer cells (Fig. 3C, Fig. S3A). Recent studies have suggested that GRP78-containing exosomes released during ER stress contribute to intracellular communication associated with cancer cell survival and death [40, 41]. To investigate the potential role of GRP78-containing exosomes in 8-gingerol-treated ovarian cancer cells, western blot analyses were performed using exosome fractions isolated from conditioned media. Treatment with 8-gingerol increased CD63 expression, indicating enhanced exosome release at the indicated time points, and markedly elevated GRP78 and ATF3 expression levels within the exosomal fraction compared with control cells (Fig. 3D). These findings suggest that GRP78- and ATF3-containing exosomes may contribute to 8-gingerol-mediated ER stress signaling. Consistent with the *in vitro* findings, tumor tissues isolated from mice treated with 8-gingerol (10 and 20 mg/kg) exhibited increased expression levels of phosphorylated PERK and ATF3 compared with tissues obtained from control mice (Fig. 3E, Fig. S3B). Thapsigargin (TG), an inhibitor of sarco/endoplasmic reticulum calcium ATPase (SERCA), is a well-established inducer of ER stress through disruption of intracellular Ca^2+^ homeostasis [42]. To further investigate whether 8-gingerol induces apoptotic cell death through ER stress signaling, the combinatory effects of TG and 8-gingerol were examined in ovarian cancer cells. Combined treatment with TG and 8-gingerol significantly reduced cell viability and increased LDH cytotoxicity and intracellular Ca^2+^ release compared with control cells (Fig. 3F-H). Western blot analyses further demonstrated that co-treatment with TG and 8-gingerol markedly increased the expression levels of ATF3, cleaved caspase-3, and phosphorylated PERK (Fig. 3I, Fig. S3C). These findings support the involvement of ER stress signaling in 8-gingerol-induced apoptotic cell death. To further determine whether 8-gingerol modulates ER stress signaling under inflammatory conditions, WST-1, LDH, intracellular Ca^2+^, and western blot analyses were performed in LPS-, 8-gingerol-, and LPS + 8-gingerol-treated Raw264.7 and J774.1 cells. LPS treatment reduced cell viability and increased LDH release and intracellular Ca^2+^ levels, whereas these effects remained largely unchanged in the 8-gingerol alone and LPS + 8-gingerol treatment groups (Fig. 3J-L). Western blot analyses demonstrated that LPS treatment alone increased ATF3 and phosphorylated PERK expression, whereas co-treatment with LPS and 8-gingerol suppressed the induction of ATF3 and phosphorylated PERK observed following LPS stimulation. Treatment with 8-gingerol alone did not significantly alter these expression levels in macrophage cells (Fig. 3M, Fig. S3D).

### Targeting the ER stress proteins suppresses apoptotic cell death in 8-gingerol-treated ovarian cancer cell lines

**Fig. 4:**
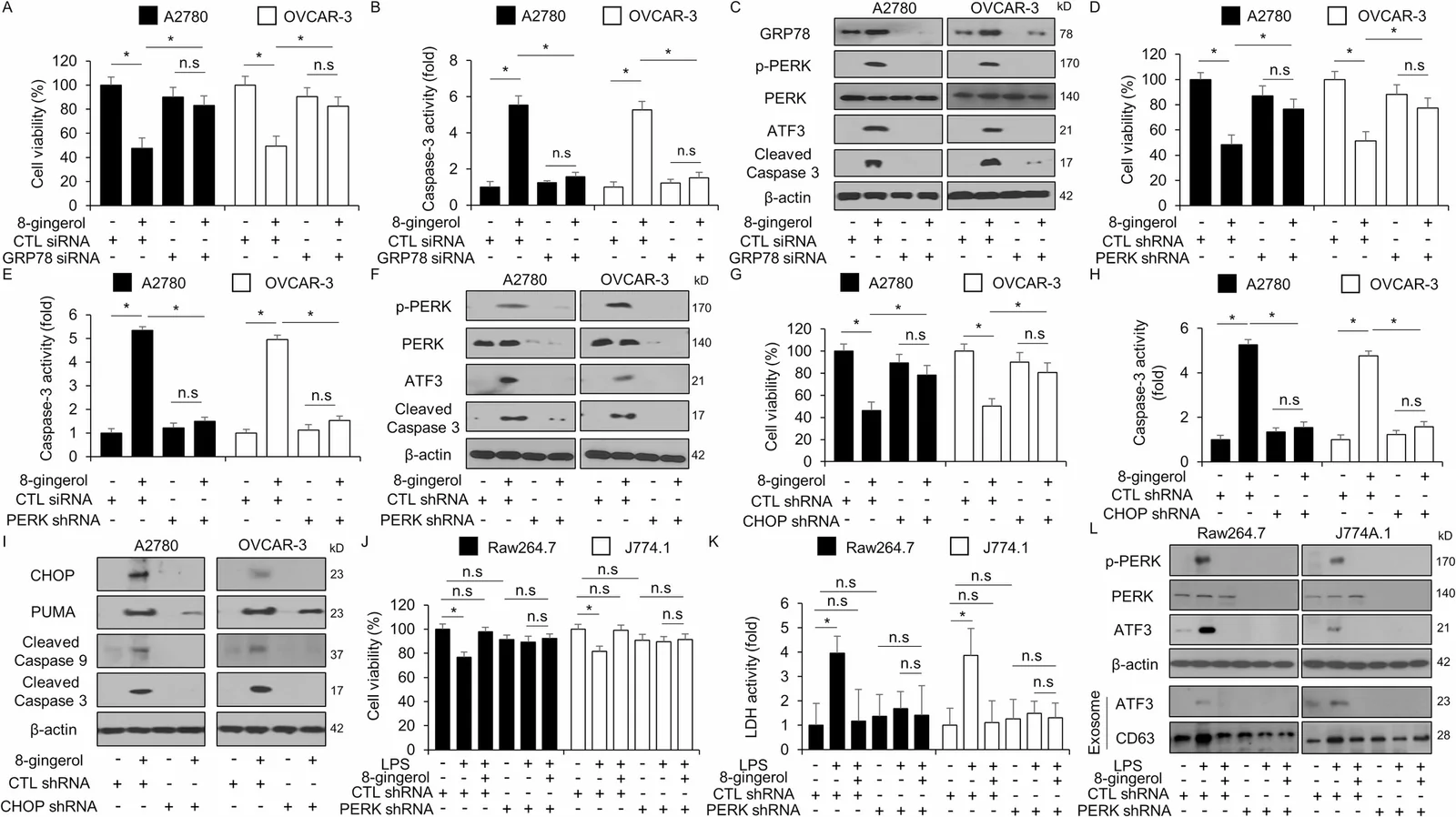
Targeting ER stress-related proteins suppresses apoptotic cell death in 8-gingerol-treated ovarian cancer cell lines. (**A–C**) Ovarian cancer cell lines were transfected with GRP78 siRNA (30 nM, 24 h) followed by treatment with 8-gingerol (50 µM, 24 h). Caspase-3 activity and cell viability were evaluated using caspase-3 activity and WST-1 assays. Protein expression levels of PERK, p-PERK, ATF3, GRP78, and cleaved caspase-3 were analyzed by western blotting. * *p* < 0.05; n.s., not significant. β-actin was used as a loading control. All experiments were performed in triplicate. (**D–F**) Caspase-3 activity and WST-1 assays were performed in PERK shRNA knockdown stable ovarian cancer cell lines following treatment with 8-gingerol (50 µM, 24 h). Protein expression levels of cleaved caspase-3, p-PERK, ATF3, and PERK were analyzed by western blotting. * *p* < 0.05; n.s., not significant. β-actin was used as a loading control. All experiments were performed in triplicate. (**G–I**) Caspase-3 activity and WST-1 assays were performed in CHOP shRNA knockdown stable ovarian cancer cell lines following treatment with 8-gingerol (50 µM, 24 h). Protein expression levels of cleaved caspase-9, cleaved caspase-3, CHOP, and PUMA were analyzed by western blotting. * *p* < 0.05; n.s., not significant. β-actin was used as a loading control. All experiments were performed in triplicate. (**J–L**) LDH cytotoxicity and WST-1 assays were performed in PERK shRNA knockdown stable murine macrophage cell lines, Raw264.7 and J774.1, following treatment with 8-gingerol (50 µM, 24 h), LPS (1 µg/mL), or a combination of 8-gingerol and LPS. Protein expression levels of p-PERK, ATF3, and PERK in whole-cell lysates and CD63 and ATF3 in exosome fractions were analyzed by western blotting. * *p* < 0.05; n.s., not significant. β-actin was used as a loading control for whole-cell lysates. All experiments were performed in triplicate

To determine whether GRP78 contributes to apoptosis and cell death in 8-gingerol-treated ovarian cancer cells, loss-of-function experiments targeting GRP78 were performed. In A2780 and OVCAR-3 cells treated with 8-gingerol, GRP78 knockdown using RNA interference (30 nM, 24 h) significantly attenuated the reduction in cell viability and suppressed the increase in caspase-3 activity compared with control cells (Fig. 4A, B). Western blot analyses further demonstrated that GRP78 knockdown reduced 8-gingerol-induced caspase-3 cleavage and suppressed the expression levels of ATF3, GRP78, and phosphorylated PERK in A2780 and OVCAR-3 cells (Fig. 4C, Fig. S4A). These findings suggest that GRP78-mediated ER stress signaling contributes to 8-gingerol-induced apoptotic cell death. Because PERK signaling plays a critical role in determining cancer cell survival and death [43], stable PERK knockdown ovarian cancer cell lines were generated using PERK shRNA to investigate downstream signaling events. In control A2780 and OVCAR-3 cells, 8-gingerol treatment reduced cell viability and increased caspase-3 activity. In contrast, PERK knockdown significantly attenuated 8-gingerol-induced cytotoxicity and caspase-3 activation (Fig. 4D, E). Western blot analyses further demonstrated that 8-gingerol increased the expression levels of ATF3, phosphorylated PERK, and cleaved caspase-3 in control cells, whereas these effects were markedly suppressed in PERK knockdown ovarian cancer cells (Fig. 4F, Fig. S4B). To investigate whether CHOP, a downstream mediator of ER stress signaling, contributes to 8-gingerol induced apoptosis, CHOP knockdown stable cell lines were generated using CHOP shRNA. Treatment with 8-gingerol reduced cell viability and increased caspase-3 activity in control cells, whereas CHOP knockdown cells exhibited significantly higher viability and lower caspase-3 activity following 8-gingerol treatment (Fig. 4G, H). To further determine whether CHOP regulates PUMA expression during 8-gingerol-mediated ER stress signaling, western blot analyses were performed. In control ovarian cancer cells, 8-gignerol treatment increased CHOP expression and caspase-3 cleavage; however, CHOP knockdown suppressed the 8-gingerol-induced upregulation of CHOP, PUMA, and caspase-9 and caspase-3 cleavage (Fig. 4I, Fig. S4C). These findings indicate that CHOP-dependent signaling contributes to apoptotic cell death induced by 8-gingerol. To further investigate the role of PERK signaling in inflammatory macrophage models, stable PERK knockdown Raw264.7 and J774.1 cells were generated using PERK shRNA. In control macrophage cells, LPS treatment (1 µg/mL) reduced cell viability and increased caspase-3 activity, whereas co-treatment with LPS and 8-gingerol (50 µM, 24 h) produced minimal additional effects. In contrast, PERK knockdown significantly attenuated the reduction in cell viability and increase in LDH cytotoxicity induced by both LPS alone and LPS + 8-gingerol treatment (Fig. 4J, K). Western blot analyses of both cell lysates and exosome fractions demonstrated that LPS treatment increased ATF3, CD63, and phosphorylated PERK expression in control macrophage cells, whereas these proteins were absent or markedly reduced following co-treatment with LPS and 8-gingerol. Furthermore, PERK knockdown suppressed the expression of ATF3, CD63, and phosphorylated PERK in response to both LPS and LPS + 8-gingerol treatment (Fig. 4L, Fig. S4D).

### 8-Gingerol-mediated ferroptosis is associated with the ATF3-GPX4 axis and NOX4-mediated ROS release in ovarian cancer cell lines

**Fig. 5:**
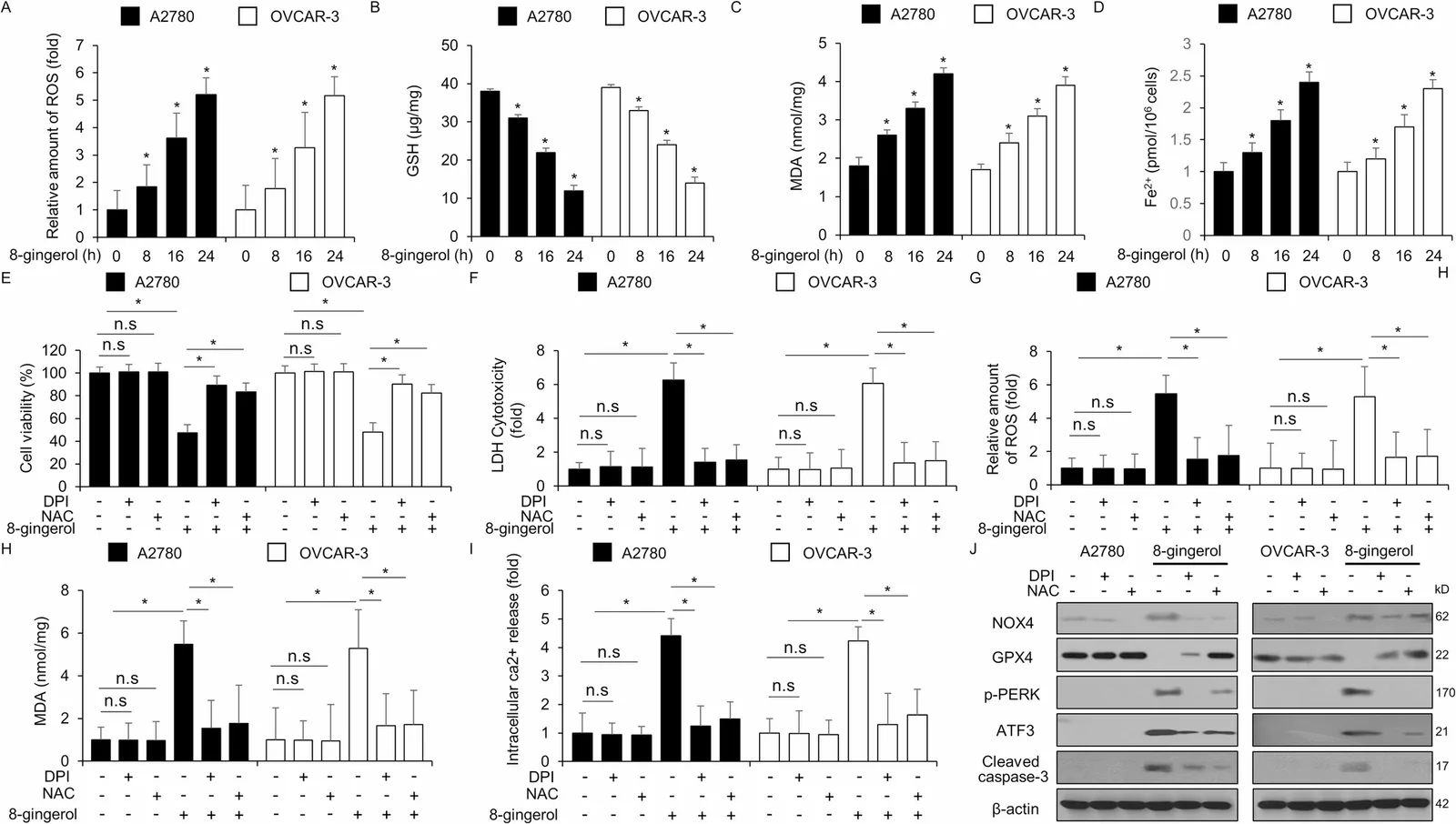
8-Gingerol induces apoptotic and ferroptotic cell death through intracellular ROS generation in ovarian cancer cell lines. (**A**) Intracellular ROS generation in ovarian cancer cell lines treated with 8-gingerol (50 µM) at the indicated time points (0, 8, 16, and 24 h), as measured using the DCFDA assay. * *p* < 0.05. (**B**,** C**) A2780 and OVCAR-3 cells were treated with 8-gingerol (50 µM) for the indicated time points, and intracellular GSH and MDA levels were analyzed. * *p* < 0.05. (**D**) Intracellular Fe^2+^ levels were measured using a colorimetric iron assay to evaluate the labile iron pool (LIP). * *p* < 0.05. (**E-I**) A2780 and OVCAR-3 cells were treated with NAC (100 µM) or DPI (1 µM) followed by treatment with 8-gingerol (50 µM, 24 h). LDH cytotoxicity, cell viability, intracellular ROS, intracellular Ca^2+^, and MDA levels were evaluated using LDH, WST-1, ROS, intracellular Ca^2+^, and MDA assays. * *p* < 0.05. (**J**) Protein expression levels of cleaved caspase-3, p-PERK, ATF3, GPX4, and NOX4 were analyzed by western blotting. β-actin was used as a loading control. All experiments were performed in triplicate

Recent studies have demonstrated that ROS regulate multiple forms of programmed cell death, including apoptosis and ferroptosis [44, 45]. To determine whether 8-gingerol modulates ROS production in ovarian cancer cells, intracellular ROS levels were assessed in A2780 and OVCAR-3 cells following 8-gingerol treatment. Treatment with 8-gingerol significantly increased intracellular ROS generation in a time-dependent manner (Fig. 5A). Ferroptosis is an iron-dependent form of cell death characterized by excessive lipid peroxidation and depletion of the antioxidant glutathione (GSH) [46]. To investigate whether 8-gingerol-induced ROS accumulation affects lipid peroxidation and antioxidant capacity, malondialdehyde (MDA) and GSH levels were evaluated. Treatment with 8-gingerol progressively reduced intracellular GSH levels while increasing MDA accumulation in a time-dependent manner (Fig. 5B, C). To further determine the effects of 8-gingerol on intracellular iron homeostasis, the labile iron pool (LIP) was measured using an iron assay. Treatment with 8-gingerol significantly increased intracellular Fe^2+^ concentrations in ovarian cancer cells compared with control cells, indicating expansion of the LIP (Fig. 5D). To investigate whether ROS inhibition suppresses 8-gingerol-induced cytotoxicity and oxidative stress, intracellular Ca^2+^, ROS, LDH, MDA, and WST-1 assays were performed in the presence of the ROS inhibitors, NAC and DPI. Co-treatment with NAC or DPI significantly attenuated the reduction in cell viability and suppressed LDH cytotoxicity, ROS generation, MDA accumulation, and intracellular Ca^2+^ release compared with treatment with 8-gingerol alone (Fig. 5E-I). Western blot analyses further demonstrated that co-treatment with NAC or DPI suppressed the 8-gingerol-induced upregulation of cleaved caspase-3, NOX4, ATF3, and phosphorylated PERK, while restoring GPX4 expression levels relative to cells treated with 8-gingerol alone (Fig. 5J, Fig. S5A). Collectively, these findings indicate that 8-gingerol induces apoptotic and ferroptotic cell death through ROS-dependent signaling pathways in ovarian cancer cells.

### 8-Gingerol induces apoptotic and ferroptotic cell death involving the TRPV1-PLCγ-NOX4 axis in ovarian cancer cell lines

**Fig. 6:**
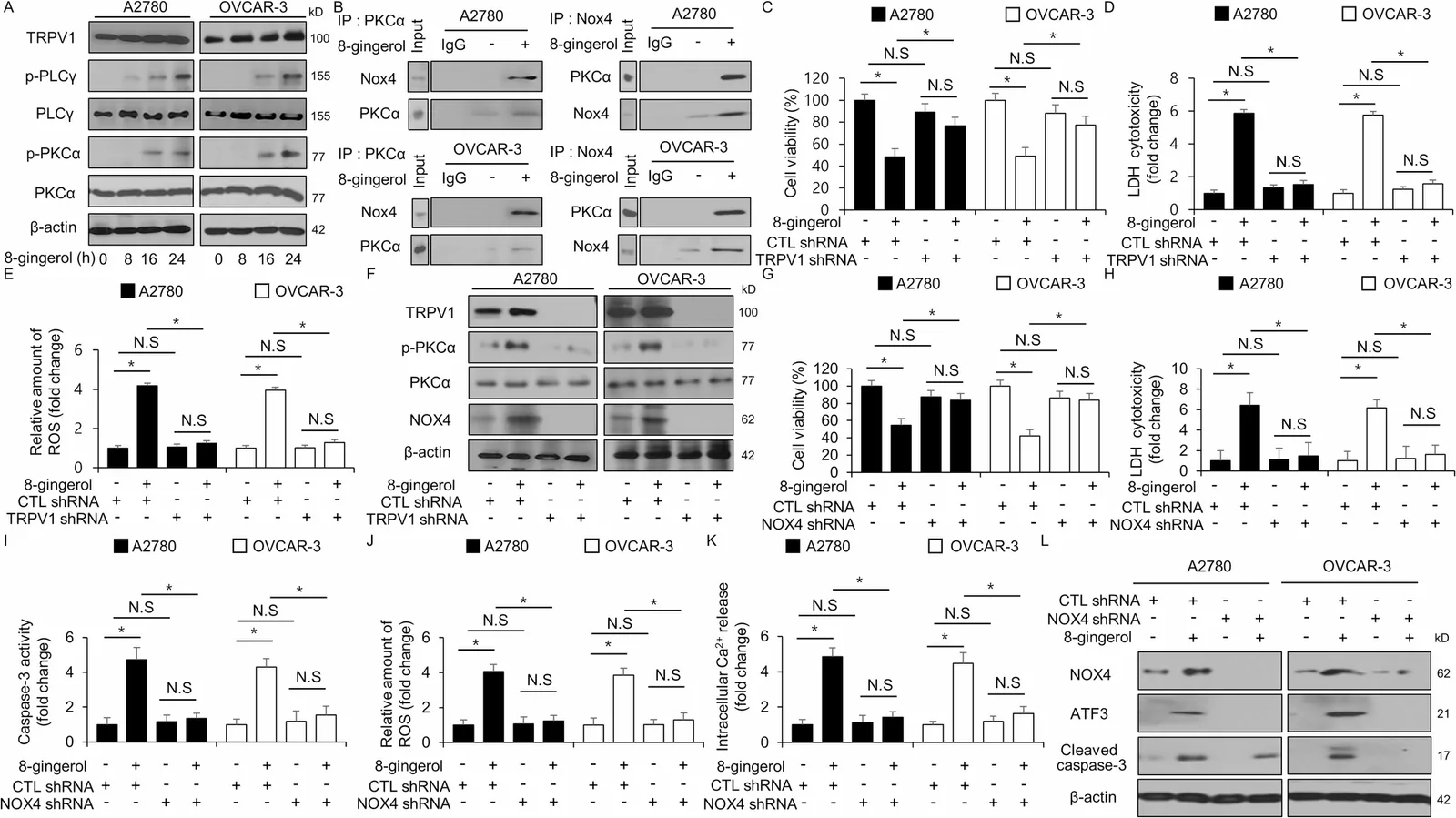
8-Gingerol induces apoptotic and ferroptotic cell death involving the TRPV1-PLCγ-NOX4 signaling axis in ovarian cancer cell lines. (**A**) A2780 and OVCAR-3 cells were treated with 8-gingerol (50 µM) at the indicated time points. Protein expression levels of TRPV1, PLCγ, p-PLCγ, PKCα, and p-PKCα were analyzed by western blotting. β-actin was used as a loading control. (**B**) Ovarian cancer cells were treated with 8-gingerol (50 µM, 24 h), and co-immunoprecipitation assays were performed to evaluate the interaction between NOX-4 and PKCα. NOX4 was detected in immunoprecipitates prepared with anti-PKCα antibody, and PKCα was detected in immunoprecipitates prepared with anti-NOX4 antibody by western blotting. (**C-F**) LDH cytotoxicity, intracellular ROS, and WST-1 assays were performed in TRPV1 shRNA knockdown stable ovarian cancer cell lines following treatment with 8-gingerol (50 µM, 24 h). Protein expression levels of TRPV1, PKCα, p-PKCα, and NOX4 were analyzed by western blotting. * *p* < 0.05; n.s., not significant. β-actin was used as a loading control. All experiments were performed in triplicate. (**G-L**) Caspase-3 activity, LDH cytotoxicity, intracellular ROS, intracellular Ca^2+^, and WST-1 assays were performed in NOX4 shRNA knockdown stable ovarian cancer cell lines following treatment with 8-gingerol (50 µM, 24 h). Protein expression levels of ATF3, cleaved caspase-3, and NOX4 were analyzed by western blotting. * *p* < 0.05; n.s., not significant. β-actin was used as a loading control. All experiments were performed in triplicate

Recent studies have identified 8-gingerol as a natural agonist of transient receptor potential vanilloid 1 (TRPV1), a critical regulator of intracellular calcium homeostasis and signaling [47, 48]. Activation of TRPV1 promotes intracellular Ca^2+^ release, leading to phosphorylation of phospholipase C (PLC) and subsequent activation of protein kinase C (PKC) [49]. To investigate whether 8-gingerol activates calcium signaling involving TRPV1 in ovarian cancer cells, western blot analyses were performed in A2780 and OVCAR-3 cells. Treatment with 8-gingerol significantly increased the expression levels of TRPV1, phosphorylated PLCγ, and phosphorylated PKCα in a time-dependent manner (Fig. 6A, Fig. S6A). PKCα is a key regulator of calcium-dependent ROS generation and oxidative stress, and NOX4 functions as a major downstream mediator of ROS production [50]. To determine whether 8-gingerol modulates the interaction between PKCα and NOX4, co-immunoprecipitation analyses were performed. Immunoprecipitation using anti-PKCα antibodies demonstrated enhanced interaction with NOX4 in 8-gingerol-treated A2780 and OVCAR-3 cells. Similarly, immunoprecipitation using anti-NOX4 antibodies confirmed increased PKCα-NOX4 complex formation following 8-gingerol treatment (Fig. 6B). These findings suggest that 8-gingerol promotes PKCα-NOX4 interaction, thereby contributing to calcium-dependent ROS generation and oxidative stress signaling. To further investigate the role of TRPV1 in 8-gingerol-mediated oxidative stress, stable TRPV1 knockdown ovarian cancer cell lines were generated using TRPV1 shRNA. In control ovarian cancer cells, treatment with 8-gingerol reduced cell viability and increased LDH cytotoxicity and intracellular ROS production. In contrast, TRPV1 knockdown significantly attenuated 8-gingerol-induced cytotoxicity, LDH release, and ROS generation (Fig. 6C-E). Western blot analyses further demonstrated that 8-gingerol increased the expression levels of TRPV1, phosphorylated PKCα, and NOX4 in control cells, whereas these effects were markedly suppressed in TRPV1 knockdown cells (Fig. 6F, Fig. S6B). These findings indicate that TRPV1 signaling contributes to 8-gingerol-induced oxidative stress and cell death in ovarian cancer cells. To further determine whether NOX4-mediated ROS production contributes to 8-gingerol-induced cytotoxicity, NOX4 knockdown stable ovarian cancer cell lines were generated using NOX4 shRNA. Following treatment with 8-gingerol, intracellular ROS, intracellular Ca^2+^, caspase-3 activity, WST-1, and LDH cytotoxicity assays were performed. Knockdown of NOX4 significantly attenuated the reduction in cell viability and suppressed intracellular ROS generation, intracellular Ca^2+^ accumulation, caspase-3 activation, and LDH release in 8-gingerol-treated A2780 and OVCAR-3 cells compared with control cells (Fig. 6G-K). In addition, NOX4 knockdown markedly reduced the expression levels of NOX4, cleaved caspase-3, and ATF3 relative to control groups (Fig. 6L, Fig. S6C). These findings suggest that NOX4 contributes to oxidative stress and cell death by promoting intracellular ROS and Ca^2+^ accumulation in 8-gingerol-induced ovarian cancer cells.

### 8-Gingerol induces ferroptotic cell death by regulating the SLC7A11-GPX4 axis and the NRF2-KEAP1-HO-1 axis in ovarian cancer cell lines

**Fig. 7:**
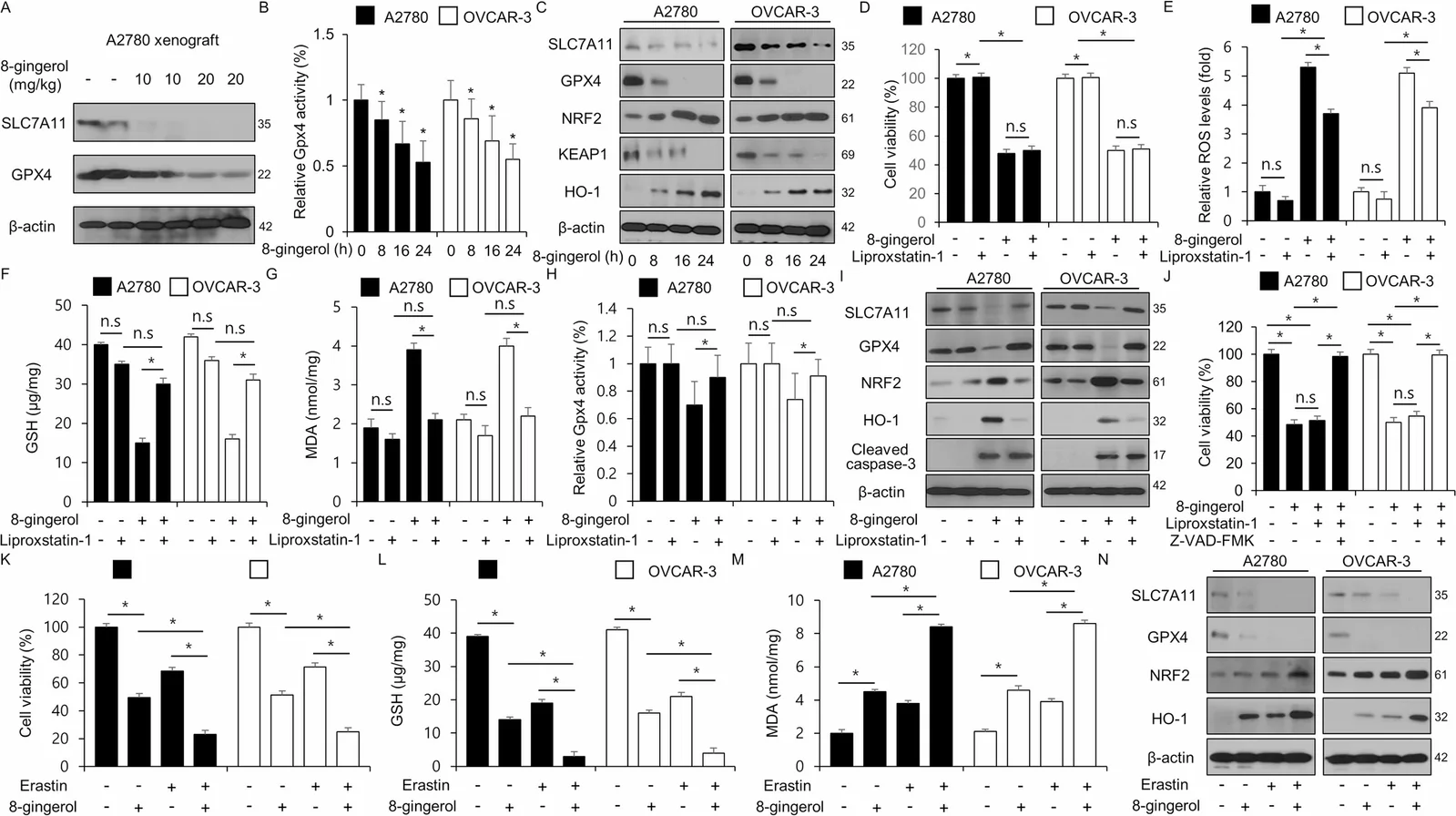
8-Gingerol induces ferroptotic cell death through regulation of the SLC7A11-GPX4-NRF2-HO-1 signaling axis in ovarian cancer cells. (**A**) Western blot analysis of SLC7A11 and GPX4 expression in tumor tissues obtained from mice treated with 8-gingerol (10 and 20 mg/kg). β-actin was used as a loading control. (**B**,** C**) A2780 and OVCAR-3 cells were treated with 8-gingerol (50 µM) at the indicated time points, and GPX4 activity and protein expression levels of SLC7A11, GPX4, NRF2, KEAP1, and HO-1 were analyzed using GPX4 activity assays and western blotting, respectively. β-actin was used as a loading control. * *p* < 0.05. (**D-I**) A2780 and OVCAR-3 cells were pretreated with Liproxstatin-1 (ferroptosis inhibitor, 2 µM, 24 h) followed by treatment with 8-gingerol (50 µM, 24 h). GPX4 activity, cell viability, intracellular ROS levels, MDA levels, and GSH levels were analyzed using GPX4 activity assays, WST-1, ROS, MDA, and GSH assays. * *p* < 0.05; n.s., not significant. Protein expression levels of cleaved caspase-3, SLC7A11, NRF2, GPX4, and HO-1 were analyzed by western blotting. β-actin was used as a loading control. All experiments were performed in triplicate. (**J**) Cells were pre-treated with the apoptosis inhibitor Z-VAD-FMK (50 µM, 4 h) and/or the ferroptosis inhibitor Liproxstatin-1 (2 µM, 4 h) prior to treatment with 8-gingerol (50 µM, 24 h). Cell viability was evaluated using a WST-1 assay. Although single-agent inhibition exerted minimal protective effects, combined treatment with Z-VAD-FMK and Liproxstatin-1 significantly restored cell viability. * *p* < 0.05; n.s., not significant. (**K-N**) A2780 and OVCAR-3 cells were treated with Erastin (ferroptosis inducer; 10 µM, 24 h) followed by treatment with 8-gingerol (50 µM, 24 h). GPX4 activity, cell viability, MDA levels, and GSH levels were evaluated using GPX4 activity, WST-1, MDA, and GSH assays. * *p* < 0.05; n.s., not significant. Protein expression levels of SLC7A11, NRF2, GPX4, and HO-1 were analyzed by western blotting. β-actin was used as a loading control. All experiments were performed in triplicate

Previous studies have suggested that crosstalk between apoptosis and ferroptosis plays an important role in ER stress-mediated cell death. During the ER stress response, p53 upregulated modulator of apoptosis (PUMA) and CHOP-mediated transcriptional regulation of the PUMA promoter have been implicated in coordinating apoptotic signaling [51]. In this context, ER stress-mediated Ca^2+^ release may function as a mechanistic link between apoptosis and ferroptosis, suggesting that ferroptotic signaling may precede apoptotic cell death [52]. To further elucidate the molecular mechanisms underlying 8-gingerol-induced ferroptosis, we investigated whether 8-gingerol modulates the expression of ferroptosis-associated proteins, including SLC7A11, GPX4, NRF2, KEAP1, and HO-1, in A2780 and OVCAR-3 cells. Tumor tissues isolated from mice treated with 8-gingerol (10 and 20 mg/kg) exhibited lower expression levels of SLC7A11 and GPX4 than tissues obtained from control mice (Fig. 7A, Fig. S7A). To further determine whether 8-gingerol regulates GPX4 activity in ovarian cancer cells, GPX4 activity assays were performed in A2780 and OVCAR-3 cells at the indicated time points. Treatment with 8-gingerol significantly reduced GPX4 activity in a time-dependent manner (Fig. 7B). Western blot analyses further demonstrated that 8-gingerol markedly reduced the protein expression levels of SLC7A11, GPX4, and KEAP1, while increasing NRF2 and HO-1 expression in a time-dependent manner (Fig. 7C, Fig. S7B). To further investigate the relationship between 8-gingerol-induced apoptosis and ferroptosis, WST-1, intracellular ROS, GSH, MDA, and GPX4 activity assays were performed in A2780 and OVCAR-3 cells following treatment with the ferroptosis inhibitor Liproxstatin-1 (2 µM, 24 h) and 8-gingerol (50 µM, 24 h). In control ovarian cancer cells, treatment with 8-gingerol reduced cell viability, GPX4 activity, and GSH levels while increasing intracellular ROS accumulation and MDA levels. Treatment with Liproxstatin-1 alone did not significantly alter these parameters. In contrast, co-treatment with Liproxstatin-1 and 8-gingerol attenuated the reduction in GPX4 activity and GSH levels and suppressed the increase in intracellular ROS and MDA accumulation induced by 8-gingerol treatment. Notably, co-treatment with Liproxstatin-1 did not restore the reduction in cell viability induced by 8-gingerol (Fig. 7D-H). These findings suggest that 8-gingerol induces cell death through interconnected ferroptotic and apoptotic signaling pathways. Western blot analyses further demonstrated that treatment with Liproxstatin-1 alone or in combination with 8-gingerol reduced NRF2 and HO-1 expression while restoring SLC7A11 and GPX4 protein levels relative to cells treated with 8-gingerol alone (Fig. 7I, Fig. S7C). Interestingly, Liproxstatin-1 treatment did not alter 8-gingerol-induced caspase-3 cleavage (Fig. 7I, Fig. S7C). Notably, combined treatment with both Liproxstatin-1 and Z-VAD-FMK significantly rescued cells from 8-gingerol-induced cell death (Fig. 7J). These findings further support the possibility that ferroptotic signaling occurs prior to apoptotic cell death in response to 8-gingerol treatment. To further investigate the ferroptosis-inducing effects of 8-gingerol, WST-1, GSH, and MDA assays were performed in A2780 and OVCAR-3 cells following treatment with the ferroptosis inducer erastin (10 µΜ, 24 h) and 8-gingerol (50 µM, 24 h). In control ovarian cancer cells, treatment with either 8-gingerol or erastin reduced cell viability and GSH levels while increasing MDA levels. Combined treatment with erastin and 8-gingerol further reduced cell viability and GSH levels and synergistically enhanced MDA accumulation (Fig. 7K-M). Western blot analyses demonstrated that treatment with either 8-gingerol or erastin alone increased NRF2 and HO-1 expression while suppressing SLC7A11 and GPX4 expression. Moreover, co-treatment with 8-gingerol and erastin synergistically enhanced NRF2 and HO-1 expression and further reduced SLC7A11 and GPX4 protein levels (Fig. 7N, Fig. S7D). Collectively, these findings suggest that 8-gingerol induces cell death through coordinated ferroptotic and apoptotic signaling pathways in ovarian cancer cells.

### 8-gingerol-mediated ferroptosis is regulated through JMJD2C signaling

**Fig. 8:**
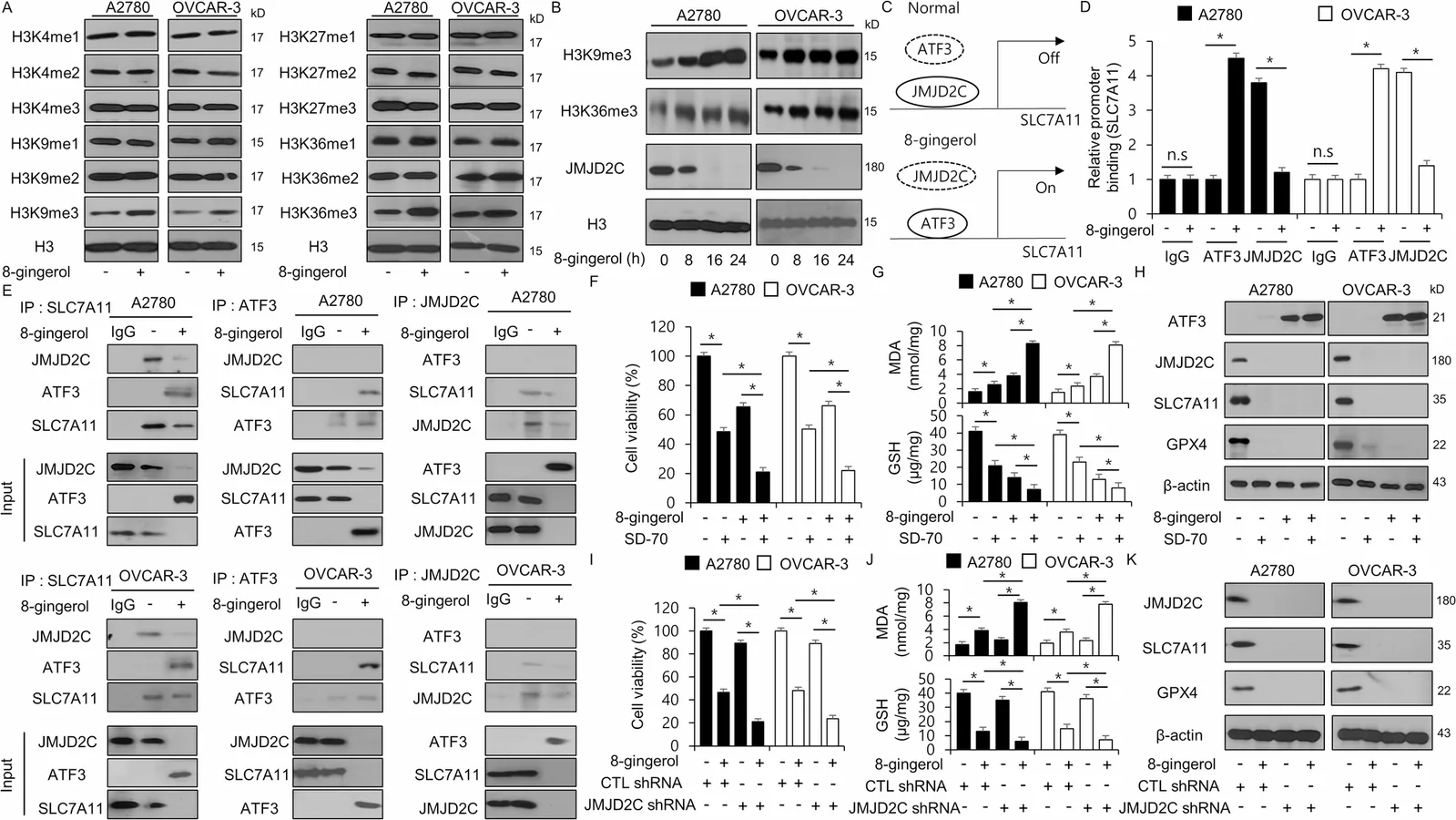
8-Gingerol-mediated ferroptosis is regulated through the ATF3-JMJD2C-SLC7A11-GPX4 signaling pathway in ovarian cancer cells. (**A**) Protein expression levels of H3K4me1, H3K4me2, H3K4me3, H3K9me1, H3K9me2, H3K9me3, H3K27me1, H3K27me2, H3K27me3, H3K36me1, H3K36me2, and H3K36me3 were analyzed by western blotting using nuclear fractions isolated from A2780 and OVCAR-3 cells treated with 8-gingerol (50 µM, 24 h). H3 was used as a loading control. (**B**) Protein expression levels of JMJD2C, H3K9me3, and H3K36me3 were analyzed by western blotting at the indicated time points (0, 8, 16, and 24 h) in 8-gingerol-treated A2780 and OVCAR-3 cells. H3 was used as a loading control. (**C**) Schematic representation of JMJD2C and ATF3 localization on the *SLC7A11* promoter region. (**D**) A2780 and OVCAR-3 cells were treated with 8-gingerol (50 µM, 24 h), and quantitative ChIP assays targeting the *SLC7A11* promoter region (-94 to -68) were performed using antibodies against JMJD2C and ATF3. (**E**) A2780 and OVCAR-3 cells were treated with 8-gingerol (50 µM, 24 h), followed by co-immunoprecipitation assays. Western blot analysis of the immunoprecipitates revealed physical interactions among ATF3, JMJD2C, and SLC7A11. SLC7A11 was detected in immunoprecipitates prepared with anti-ATF3 and anti-JMJD2C antibodies, whereas ATF3 was detected in immunoprecipitates prepared with anti-SLC7A11 and anti-JMJD2C antibodies. JMJD2C was also detected in immunoprecipitates prepared with anti-ATF3 and anti-SLC7A11 antibodies. (**F-H**) A2780 and OVCAR-3 cells were treated with 8-gingerol (50 µM, 24 h) and SD-70 (JMJD2C inhibitor; 30 µM, 24 h). Cell viability, MDA levels, and GSH levels were analyzed using WST-1, MDA, and GSH assays. Protein expression levels of ATF3, JMJD2C, SLC7A11, and GPX4 were analyzed by western blotting. β-actin was used as a loading control. * *p* < 0.05; n.s., not significant. (**I-K**) JMJD2C knockdown stable A2780 and OVCAR-3 cells were treated in the presence or absence of 8-gingerol (50 µM, 24 h). Cell viability, MDA levels, and GSH levels were analyzed using WST-1, MDA, and GSH assays. Protein expression levels of JMJD2C, SLC7A11, and GPX4 were analyzed by western blotting. β-actin was used as a loading control. * *p* < 0.05; n.s., not significant

Jumonji domain containing 2 C (JMJD2C/KDM4C) is a histone demethylase that catalyzes the demethylation of trimethylated H3K9 (H3K9me3) and H3K36 (H3K36me3) [53]. JMJD2C is frequently upregulated in multiple cancer types, including ovarian, breast, and prostate cancers [54–56]. Increased JMJD2C expression has been associated with elevated HIF-1α and MDM2 expression and reduced p53 levels [57]. To determine whether 8-gingerol regulates histone methylation in ovarian cancer cells, western blot analyses were performed to evaluate multiple histone methylation marks, including H3 lysine 4 (H3K4me1, H3K4me2, and H3K4me3), H3 lysine 9 (H3K9me1, H3K9me2, and H3K9me3), H3 lysine 27 (H3K27me1, H3K27me2, and H3K27me3), and H3 lysine 36 (H3K36me1, H3K36me2, and H3K36me3). Among these histone modifications, 8-gingerol treatment selectively increased H3K9me3 and H3K36me3 levels in A2780 and OVCAR-3 cells, whereas other histone methylation marks were not significantly altered (Fig. 8A, Fig. S8A). Notably, 8-gingerol treatment induced a time-dependent increase in H3K9me3 and H3K36me3 levels while simultaneously reducing JMJD2C expression (Fig. 8B, Fig. S8B). To determine whether the reduction in JMJD2C protein expression was associated with transcriptional regulation, JMJD2C mRNA levels were further evaluated by RT-qPCR. Treatment with 8-gingerol significantly reduced JMJD2C mRNA expression in a time-dependent manner (Fig. S8C), suggesting that 8-gingerol-mediated suppression of JMJD2C occurs, at least in part, at the transcriptional level. Previous studies have demonstrated that the ER stress-associated transcription factor ATF3 promotes erastin-induced ferroptosis through direct binding to the *SLC7A11* promoter [58]. Based on these observations, chromatin immunoprecipitation (ChIP) assays were performed to determine whether JMJD2C and ATF3 regulate the *SLC7A11* promoter region (-94 to -68) in 8-gingerol-treated A2780 and OVCAR-3 cells (Fig. 8C). Chromatin isolated from control and 8-gingerol-treated ovarian cancer cells was immunoprecipitated using antibodies against ATF3 or JMJD2C, followed by quantitative PCR amplification of the *SLC7A11* promoter region (-94 to -68) (Fig. 8D). These analyses demonstrated that JMJD2C strongly associated with the *SLC7A11* promoter in control A2780 and OVCAR-3 cells, whereas ATF3 binding was minimal under basal conditions. In contrast, 8-gingerol treatment suppressed JMJD2C occupancy while markedly enhancing ATF3 binding at the *SLC7A11* promoter region (Fig. 8D). These findings suggest that JMJD2C binding to the *SLC7A11* promoter suppresses ferroptotic signaling, whereas 8-gingerol promotes ferroptosis through inhibition of JMJD2C and enhanced recruitment of ATF3 to the *SLC7A11* promoter. To further investigate whether 8-gingerol modulates the interaction among JMJD2C, ATF3, and SLC7A11 during ferroptotic signaling, co-immunoprecipitation analyses were performed using antibodies against JMJD2C, ATF3, and SLC7A11. Treatment with 8-gingerol reduced JMJD2C levels and increased ATF3 levels in SLC7A11-immunoprecipitated fractions, while decreasing JMJD2C levels and increasing SLC7A11-associated ATF3 complexes in ATF3-immunoprecipitated fractions. In JMJD2C-immunoprecipitated fractions, 8-gingerol reduced JMJD2C levels without substantially altering ATF3 or SLC7A11 expression (Fig. 8E). These findings indicate that 8-gingerol dynamically regulates the interaction among JMJD2C, ATF3, and SLC7A11 during ferroptotic signaling. Previous studies have shown that selective inhibition of JMJD2C can induce apoptotic and ferroptotic cell death [59]. To determine whether suppression of JMJD2C and SLC7A11, together with increased H3K9me3 and H3K36me3 levels, contributes to 8-gingerol-mediated ferroptosis, WST-1, MDA, and GSH assays were performed in A2780 and OVCAR-3 cells treated with the JMJD2C inhibitor SD-70. Treatment with either 8-gingerol or SD-70 reduced cell viability and GSH levels while increasing MDA accumulation (Fig. 8F, G). Notably, combined treatment with SD-70 and 8-gingerol synergistically enhanced the reduction in cell viability and GSH levels and further increased MDA accumulation (Fig. 8F, G). Western blot analyses demonstrated that treatment with either 8-gingerol or SD-70 reduced JMJD2C, SLC7A11, and GPX4 expression. In addition, 8-gingerol alone increased ATF3 expression, whereas SD-70 treatment alone did not significantly alter ATF3 levels (Fig. 8H, Fig. S8C). Combined treatment with SD-70 and 8-gingerol further suppressed JMJD2C, SLC7A11, and GPX4 expression while synergistically increasing ATF3 expression (Fig. 8H). To further determine whether inhibition of JMJD2C regulates 8-gingerol-mediated ferroptotic and apoptotic cell death, WST-1, MDA, and GSH assays were performed in JMJD2C knockdown stable A2780 and OVCAR-3 cells. In control cells, 8-gingerol treatment reduced cell viability and GSH levels while increasing MDA levels (Fig. 8I, J). Notably, JMJD2C knockdown further enhanced the reduction in cell viability and GSH levels and increased MDA accumulation in 8-gingerol-treated cells (Fig. 8I, J). Western blot analyses demonstrated that 8-gingerol treatment reduced JMJD2C, SLC7A11, and GPX4 expression in control cells. Similarly, JMJD2C knockdown further suppressed JMJD2C, SLC7A11, and GPX4 expression in 8-gingerol-treated JMJD2C knockdown stable cells (Fig. 8K, Fig. S8D). Collectively, these findings suggest that 8-gingerol promotes ferroptotic cell death through activation of the ATF3-JMJD2C-SLC7A11-GPX4 signaling axis in ovarian cancer cells.

## Discussion

Previous studies have demonstrated the anti-inflammatory properties of 8-gingerol; however, the present study further establishes that 8-gingerol suppresses tumor growth and induces cytotoxic effects in ovarian cancer both *in vitro* and *in vivo*. Mechanistically, our findings demonstrate that 8-gingerol-induced intracellular Ca^2+^ release activates ER stress signaling, thereby promoting both ferroptotic and apoptotic cell death involving the TRPV1-PLCγ-NOX4 cascade and the ATF3-JMJD2C-SLC7A11-GPX4 signaling axis in ovarian cancer cells. Importantly, this study further reveals that dynamic and competitive promoter-associated interactions between JMJD2C and ATF3 contribute to 8-gingerol-induced ferroptotic signaling prior to the induction of apoptosis.

Inflammation plays a critical role in tumor initiation, progression, metastasis, and remodeling of the tumor microenvironment [60]. In addition, pro-tumorigenic cytokines, including IL-6, IL-11, and IL-22, are closely associated with the development of chemoresistance and radioresistance [61]. Increasing evidence suggests that natural compounds with anti-inflammatory activity may provide promising therapeutic approaches for cancer treatment [62]. Curcumin, a polyphenolic compound isolated from the rhizome of *Curcuma longa (Jianghuang)*, has been reported to exert anti-inflammatory effects through regulation of cytokines such as TNF-α, IL-6, and NF-kB, while also inducing anti-cancer activity through activation of both intrinsic and extrinsic apoptotic pathways within the tumor-immune microenvironment [63, 64].

In the present study, LPS stimulation markedly increased the expression levels of TNF-α, IL-1β, and IL-6 in Raw264.7 cells. In contrast, co-treatment with 8-gingerol significantly suppressed the LPS-induced upregulation of TNF-α, IL-1β, and IL-6. These findings suggest that 8-gingerol exerts potent anti-inflammatory effects in immune cells and may modulate inflammatory signaling associated with tumor progression. Therefore, the anti-inflammatory properties of 8-gingerol may contribute substantially to its anti-tumor activity. Collectively, these findings support the therapeutic potential of 8-gingerol as a candidate anti-cancer agent.

Previous studies have reported that ginger extracts and their bioactive constituents, including 6-gingerol, 8-gingerol, 10-gingerol, 6-shogaol, 6-paradol, and zingerone, exert potent anti-cancer effects through caspase-dependent apoptotic signaling in a wide range of malignancies, including breast, prostate, ovarian, colon, hepatocellular, gastric, non-small cell lung, melanoma, endometrial adenocarcinoma, cervical, pancreatic, and head and neck squamous cell carcinoma [65]. 6-Shogaol has been shown to induce apoptotic cell death through activation of ER stress pathways, including PERK-eIF2α-CHOP signaling, and to overcome gefitinib resistance [66]. In addition, combined treatment with 6-gingerol and cisplatin exerts synergistic anti-tumor effects through suppression of Bcl-2, VEGF, KDR, and FLT1 signaling in ovarian cancer [67]. Despite these observations, the molecular mechanisms underlying the anti-cancer effects of 8-gingerol in ovarian cancer have remained incompletely understood. Our findings demonstrate that 8-gingerol exerts potent anti-cancer activity by suppressing cell viability and tumor growth while increasing LDH release and caspase-3 activity in ovarian cancer models both *in vitro* and *in vivo*. Furthermore, 8-gingerol induced apoptotic signaling through activation of caspase-9 and caspase-3 cleavage, whereas treatment with the pan-caspase inhibitor Z-VAD-FMK attenuated the reduction in cell viability, LDH release, caspase-3 activity, and caspase-3 cleavage induced by 8-gingerol in A2780 and OVCAR-3 cells. These findings indicate that 8-gingerol promotes anti-tumor activity, at least in part, through caspase-dependent apoptotic signaling pathways. Importantly, the translational relevance of these findings is strengthened by the observed activity of 8-gingerol in treatment-resistant ovarian cancer cell populations. Ongoing investigations into multiple forms of therapeutic resistance suggest that 8-gingerol effectively suppresses the survival of ovarian cancer cells that have acquired resistance to conventional anti-cancer therapies. These findings raise the possibility that 8-gingerol-mediated activation of apoptotic and ferroptotic pathways may circumvent key molecular mechanisms underlying clinical therapeutic resistance. Despite the significance of these findings, several limitations should be acknowledged. First, the present study employed a subcutaneous xenograft model to evaluate the *in vivo* anti-tumor effects of 8-gingerol. Although this model is advantageous for monitoring tumor growth and validating molecular signaling alterations, it does not fully recapitulate the complex peritoneal tumor microenvironment characteristic of ovarian cancer. Future studies utilizing orthotopic or intraperitoneal ovarian cancer models will be necessary to further establish the biological and clinical relevance of the PERK-ATF3-JMJD2C signaling axis. In addition, the translation of 8-gingerol remains limited by important pharmacokinetic challenges. Similar to other ginger-derived compounds, 8-gingerol exhibits relatively low systemic bioavailability because of poor aqueous solubility and extensive first-pass metabolism within the liver and gastrointestinal tract [68]. Previous studies have demonstrated that gingerols undergo rapid conversion into glucuronide and sulfate conjugates, potentially limiting the plasma concentrations required to achieve optimal therapeutic efficacy *in vivo* [69]. To address these limitations, several advanced delivery approaches are currently under investigation. Nanoformulation-based delivery systems, including liposomes, polymeric nanoparticles, and self-microemulsifying drug delivery systems (SMEDDS), have shown promise in enhancing the solubility, stability, and systemic retention of gingerol compounds [70]. In addition, structural modification strategies or co-administration with selective metabolic inhibitors may further delay systemic clearance and improve therapeutic bioavailability. Future translational and clinical investigations should prioritize optimization of these advanced delivery systems to enhance tumor-specific accumulation of 8-gingerol and maximize its therapeutic efficacy in ovarian cancer.

Excessive or sustained ER stress has been shown to induce multiple forms of programmed cell death, including ferroptosis and apoptosis, through dysregulated intracellular calcium signaling in a variety of cancer models, thereby representing a promising therapeutic target in oncology [71]. Consistent with these observations, our findings demonstrate that 8-gingerol induces both ferroptotic and apoptotic cell death through activation of ER stress-associated signaling pathways, including the PERK-ATF3-CHOP-PUMA axis, in ovarian cancer cell lines. Numerous upstream regulatory pathways, including Bcl-2 family proteins, kinase-mediated signaling cascades, and transcriptional regulators, contribute to caspase activation and apoptotic cell death [72]. In the present study, 8-gingerol induced caspase-9 and caspase-3-dependent apoptotic signaling in A2780 and OVCAR-3 cells. To further determine whether 8-gingerol-mediated cytotoxicity depends on caspase activation, pharmacological inhibition studies were performed using the pan-caspase inhibitor Z-VAD-FMK. Co-treatment with Z-VAD-FMK markedly attenuated 8-gingerol-induced apoptotic cell death in ovarian cancer cells, supporting the conclusion that 8-gingerol induces apoptosis through a caspase-dependent mechanism. In ovarian cancer cells, Interestingly, the present study also revealed a contrasting regulatory effect of 8-gingerol in LPS-stimulated macrophages, in which 8-gingerol suppressed PERK-ATF3 signaling rather than activating it, as observed in ovarian cancer cells. This differential response likely reflects the distinct basal proteostatic environments and signaling landscapes characteristic of malignant versus immune cell populations. Ovarian cancer cells frequently operate under chronic ER stress conditions to sustain rapid proliferation and metabolic adaptation; therefore, 8-gingerol may function as a pro-oxidant stimulus or promote TRPV1-mediated calcium overload, thereby driving ER stress beyond its adaptive threshold and triggering apoptotic signaling [73]. In contrast, under acute inflammatory conditions in macrophages, 8-gingerol may act as a homeostatic regulator that suppresses excessive inflammatory activation. This protective effect may be mediated, at least in part, through activation of the NRF2/HO-1 signaling axis, which antagonizes TLR4-dependent ROS generation and prevents excessive activation of PERK signaling [74]. Collectively, these findings suggest that the pharmacological activity of 8-gingerol is highly context-dependent and may be determined by the intrinsic ER stress “set-point” of individual cell types, functioning as a pro-apoptotic agent in malignant cells while exerting anti-inflammatory activity in immune cells.

We next investigated whether the combined treatment of 8-gingerol and thapsigargin (TG) further potentiates apoptotic signaling. Co-treatment with 8-gingerol and TG induced greater apoptotic cell death than treatment with 8-gingerol alone, accompanied by enhanced activation of caspase-3 and increased expression of ER stress-associated proteins, including GRP78, p-PERK, p-eIF2α, ATF3, CHOP, and PUMA. Previous studies have demonstrated that CHOP directly binds to the PUMA promoter and that activation of the CHOP-PUMA signaling axis contributes to both apoptotic and ferroptotic cell death [75]. Consistent with these findings, our results demonstrated that 8-gingerol induced time-dependent upregulation of GRP78, ATF3, and CHOP, together with increased phosphorylation of PERK and eIF2α, in association with intracellular Ca^2+^ release in ovarian cancer cells. In contrast, knockdown of ER stress-associated proteins, including CHOP, GRP78, and PERK, significantly attenuated apoptotic cell death by suppressing caspase-3 activity, reducing caspase-3 cleavage, and decreasing expression of CHOP, PUMA, p-PERK, and GRP78 in 8-gingerol-treated ovarian cancer cells. These findings indicate that 8-gingerol-induced calcium signaling promotes both apoptotic and ferroptotic cell death through activation of the PERK-ATF3-CHOP-PUMA signaling pathway in ovarian cancer cells. Furthermore, exosome fractions isolated from 8-gingerol-treated ovarian cancer cell culture media exhibited a time-dependent increase in exosomal GRP78 and ATF3 expression. Previous studies have reported that cancer cell-derived exosomes can induce apoptosis through activation of ER stress signaling pathways [76, 77]. However, to the best of our knowledge, the present study is the first to demonstrate a potential relationship between 8-gingerol treatment and ER stress-associated exosomal signaling. Our findings suggest that exosomal GRP78 and ATF3 derived from 8-gingerol-treated ovarian cancer cells may contribute to apoptotic signaling through activation of the PERK pathway. In the present study, exosome isolation was primarily validated using the tetraspanin marker CD63. However, according to the Minimal Information for Studies of Extracellular Vesicles (MISEV) guidelines, more comprehensive extracellular vesicle characterization, including multiple positive markers and at least one negative marker, is recommended to minimize the possibility of non-vesicular contamination. Therefore, reliance on a single exosomal marker represents a limitation of the current study. Future investigations incorporating a broader panel of extracellular vesicle markers will be necessary to further validate the purity and identity of the isolated vesicle populations.

TRPV1 represents an attractive therapeutic target in inflammatory disease because of its regulatory effects on inflammatory cytokines, including IL-6 [78]. Our findings demonstrated that the TRPV1 agonist 8-gingerol suppresses LPS-induced inflammatory cytokine production both *in vitro* and *in vivo*. Activation of TRPV1 has been reported to induce multiple forms of cell death, including apoptosis and ferroptosis, through intracellular calcium release and ROS generation in several cancer types, including gastric and breast cancer [79–81]. TRPV1 is frequently expressed within the ER membrane, where it contributes to ER stress-associated cell death signaling [82]. Activation of TRPV1 has been shown to induce ER stress through calcium-dependent signaling pathways, including the PLC-PKC-NOX4 and EGFR-RAS-RAF-MEK-ERK cascades [83]. PLC-mediated hydrolysis of PIP_2_ subsequently activates PKC signaling [84]. PKCα plays a critical role in regulating intracellular ROS production, calcium signaling, and oxidative stress and functions as an important upstream regulator of NOX4 activation [85]. Recent studies have further demonstrated that Ca^2+^ influx promotes ferroptosis through the TRPM7-PLC-PKC-NOX4 signaling pathway [21]. Moreover, NOX4-mediated ROS production has been implicated in multiple forms of programmed cell death, including apoptosis, ferroptosis, and autophagy [86]. Consistent with these observations, our findings demonstrate that 8-gingerol induces ER stress-mediated apoptotic and ferroptotic cell death involving the activation of the TRPV1-PLCγ-PKCα-NOX4 signaling axis. Treatment with 8-gingerol promoted both the up-regulation of PKCα and NOX4 and the interaction between these proteins in a TRPV1-dependent manner. Inhibition of TRPV1 suppressed activation of the PKCα-NOX4 signaling axis, accompanied by reduction in LDH cytotoxicity and intracellular ROS production in 8-gingerol-treated ovarian cancer cells. Furthermore, our findings presented in Fig. 6F demonstrated that suppression of p-PKCα and NOX4 expression by 8-gingerol was markedly attenuated following TRPV1 knockdown, highlighting TRPV1 as a critical upstream regulator of this signaling pathway. Nevertheless, although TRPV1 appears to play a dominant role in mediating the effects of 8-gingerol, the possibility that 8-gingerol may also influence downstream signaling molecules or parallel stress-sensing pathways cannot be completely excluded. Additional mechanistic studies will therefore be required to determine whether 8-gingerol directly interacts with these signaling components and to further define its molecular binding properties.

Previous studies have demonstrated that the interplay between ER stress and reduced redox homeostasis is a critical determinant of cellular fate and stress-responsive signaling processes [87]. Oxidative stress disrupts intracellular redox balance through excessive ROS generation [88]. Major contributors to ROS production include endoplasmic reticulum oxidoreductin (ERO1), protein disulfide isomerase (PDI), intracellular calcium signaling, and NADPH oxidase complexes such as NOX4, all of which participate in the regulation of UPR signaling and ER stress responses [89]. Consistent with these observations, our findings indicate that 8-gingerol promotes intracellular ROS generation, calcium release, and ER stress signaling through the activation of NOX4 in the ovarian cancer cell lines A2780 and OVCAR-3. Furthermore, both genetic suppression of NOX4 using NOX4-specific shRNA and pharmacological inhibition using DPI or NAC significantly attenuated apoptotic and ferroptotic cell death in 8-gingerol-treated ovarian cancer cells. These inhibitory effects were accompanied by reductions in LDH cytotoxicity, intracellular ROS accumulation, MDA levels, and PERK-ATF3 signaling activity, further supporting the central role of oxidative stress in 8-gingerol-mediated cytotoxicity.

Ferroptosis is a ROS-dependent form of regulated cell death that is critically controlled through suppression of the SLC7A11-GPX4 signaling axis in cancer cells [90]. SLC7A11 promotes the uptake of extracellular cystine for glutathione synthesis and contributes to activation of the NRF2-HO-1 antioxidant defense pathway, whereas inhibition of SLC7A11 reduces glutathione availability and subsequently suppresses GPX4 activity [91]. Our findings demonstrated that 8-gingerol induces ferroptotic cell death through modulation of the SLC7A11-GPX4-NRF2-HO-1 signaling network, whereas treatment with the ferroptosis inhibitor Liproxstatin-1 attenuated ferroptotic signaling and partially restored SLC7A11-GPX4-NRF2-HO-1 pathway activity in 8-gingerol-treated ovarian cancer cells. These findings support the conclusion that disruption of antioxidant defense mechanisms contributes substantially to 8-gingerol-induced ferroptosis. In addition, our findings suggest that epigenetic regulation of SLC7A11 represents a critical mechanism underlying 8-gingerol-induced ferroptotic cell death. Previous studies have reported that ER stress-associated transcription factors ATF3 and ATF4 bind to the SLC7A11 promoter, although ATF3 appears to play a more dominant role than ATF4 in promoting erastin-induced ferroptosis [55, 92]. In the present study, western blot analyses demonstrated that 8-gingerol increased H3K9me3 and H3K36me3 levels in ovarian cancer cells. Moreover, ChIP and immunoprecipitation analyses demonstrated that JMJD2C interacted with SLC7A11 under basal conditions, whereas 8-gingerol treatment disrupted the JMJD2C-SLC7A11 interaction and promoted recruitment of ATF3 and SLC7A11. These findings suggest that 8-gingerol promotes ferroptotic signaling through epigenetic remodeling of the SLC7A11 regulatory complex. Consistent with this interpretation, inhibition of JMJD2C further enhanced ferroptotic cell death through suppression of the SLC7A11-GPX4 signaling axis.

Despite the potent *in vitro* anti-tumor activity observed in the present study, the systemic bioavailability of 8-gingerol remains an important limitation for clinical translation. Similar to many lipophilic polyphenolic compounds, 8-gingerol is likely subject to rapid metabolic degradation and systemic clearance. Future investigations should therefore focus on the development of nanoformulations and advanced drug delivery systems capable of improving its pharmacokinetic stability and tumor-specific accumulation. In addition, although the present study primarily focused on PERK-mediated ER stress signaling, the potential contribution of the IRE1α-XBP1 signaling axis warrants further investigation. Because 8-gingerol modulates ER stress-associated apoptotic signaling, it is plausible that this compound may also influence XBP1 splicing and broader proteostatic regulatory mechanisms.

Collectively, the present study demonstrates that 8-gingerol, an anti-inflammatory compound and TRPV1 agonist, suppresses ovarian cancer cell proliferation and tumor growth through coordinated induction of ferroptotic and apoptotic cell death both *in vitro* and *in vivo*. Our findings further demonstrate that 8-gingerol induces ER stress-mediated apoptotic and ferroptotic signaling in a TRPV1-dependent manner, involving the PLCγ-NOX4 signaling cascade and the ATF3-JMJD2C-SLC7A11-GPX4 signaling axis in ovarian cancer cells. These findings provide important mechanistic insights into the anti-tumor activity of 8-gingerol and support its potential therapeutic application as a targeted strategy for ovarian cancer treatment.

## Supplementary Information

Supplementary Material 1

Supplementary Material 2

## Data Availability

No datasets were generated or analysed during the current study.
