# Supplementary material for: 8-gingerol from Zingiber officinale induces ER stress-mediated ferroptosis and apoptosis in ovarian cancer involving TRPV1 activation
Source: Cell Commun Signal. 2026 Jul 10;24:522. doi: 10.1186/s12964-026-03014-8 (PMC13629130; doi:10.1186/s12964-026-03014-8)

Western blotting whole blot: 8-gingerol

Tae Woo Kim

Whole blots for Figure 1E

β-actin (43 kDa)

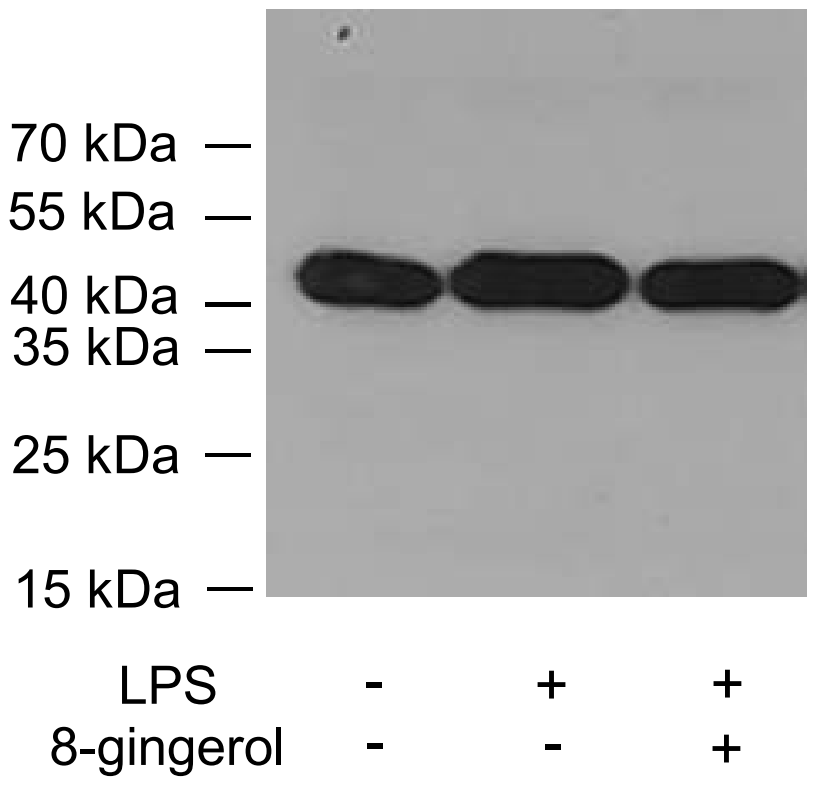

TNF-a (25 kDa)

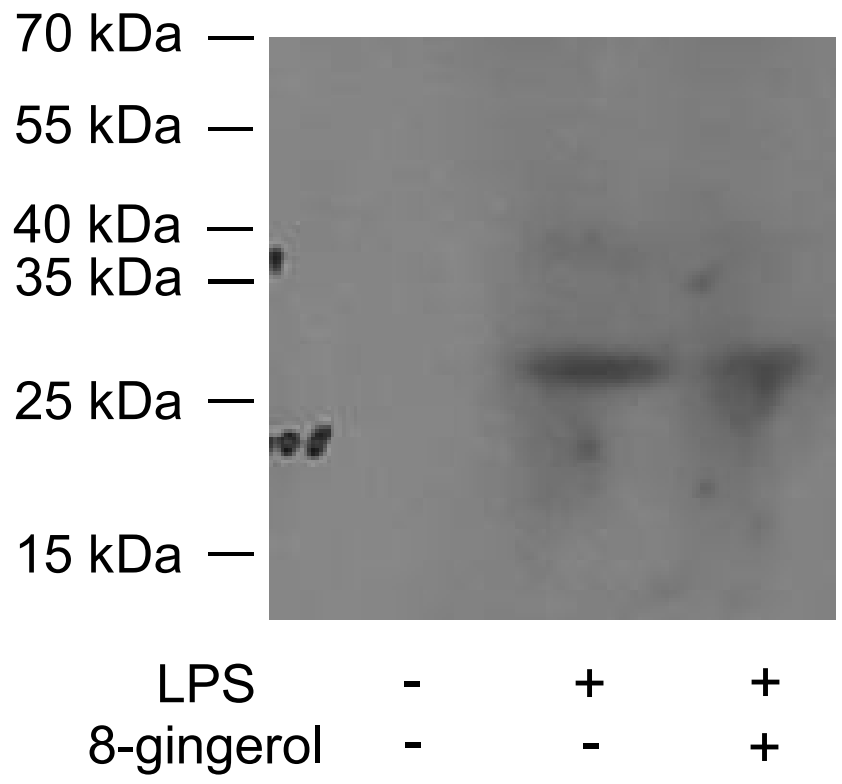

β-actin (43 kDa)

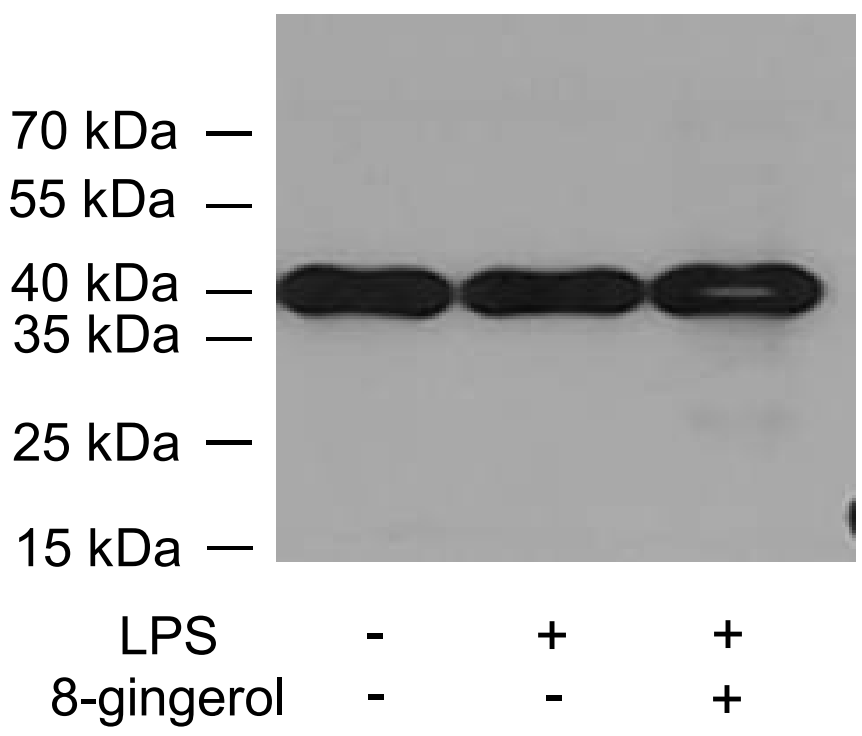

IL-b (31 kDa)

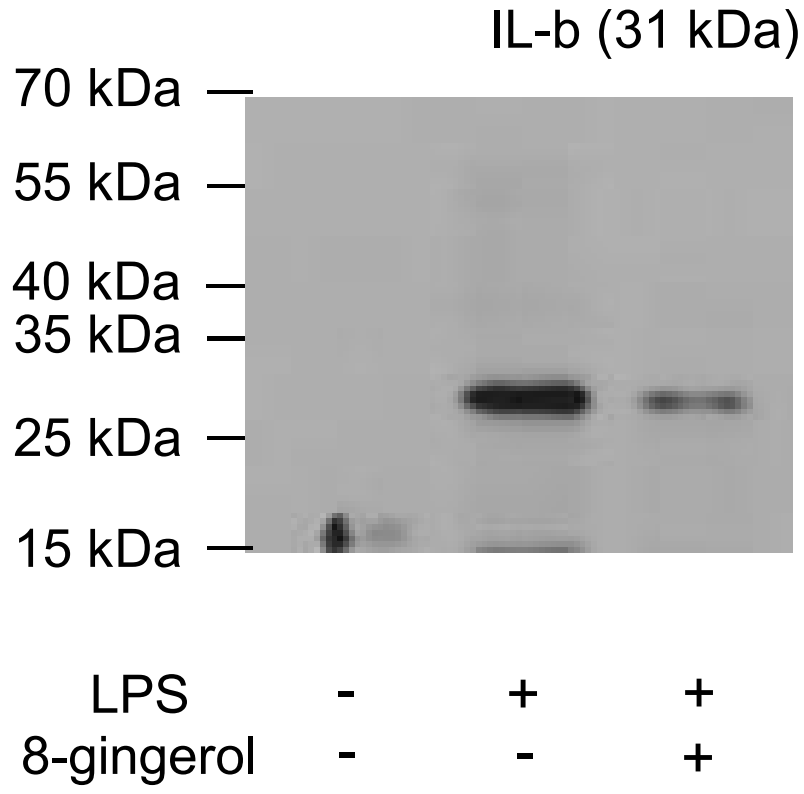

β-actin (43 kDa)

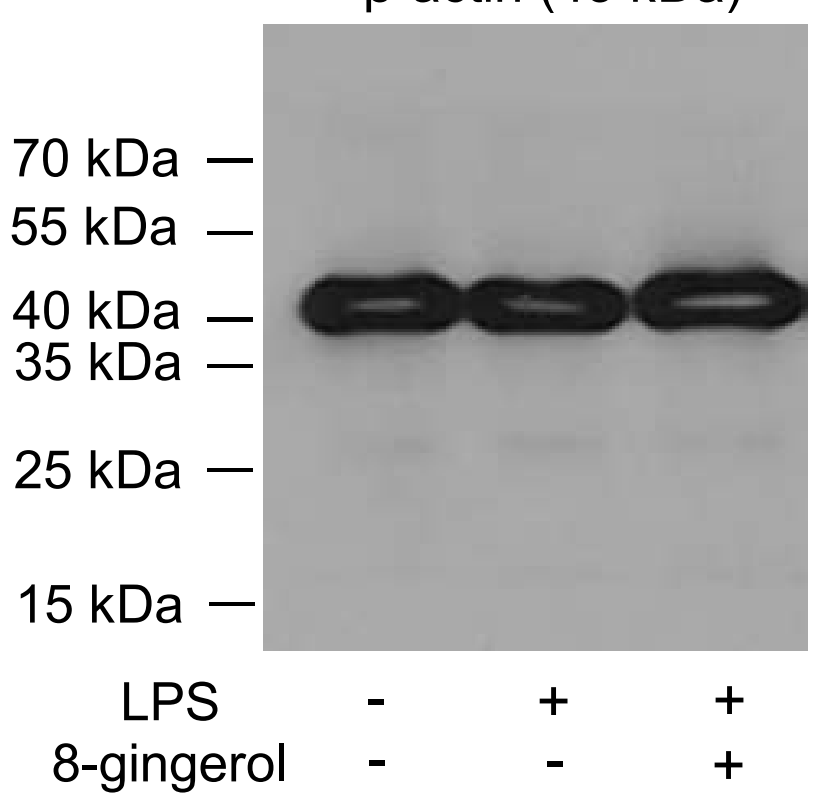

IL-6 (24 kDa)

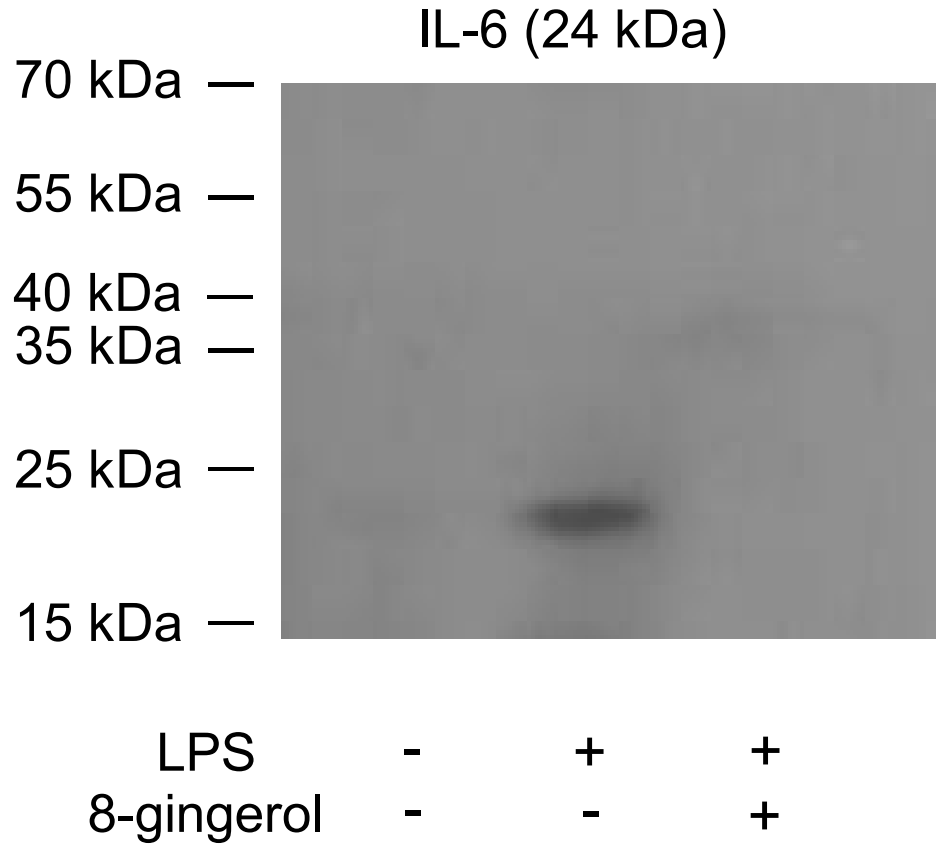

Whole blots for Figure 1F

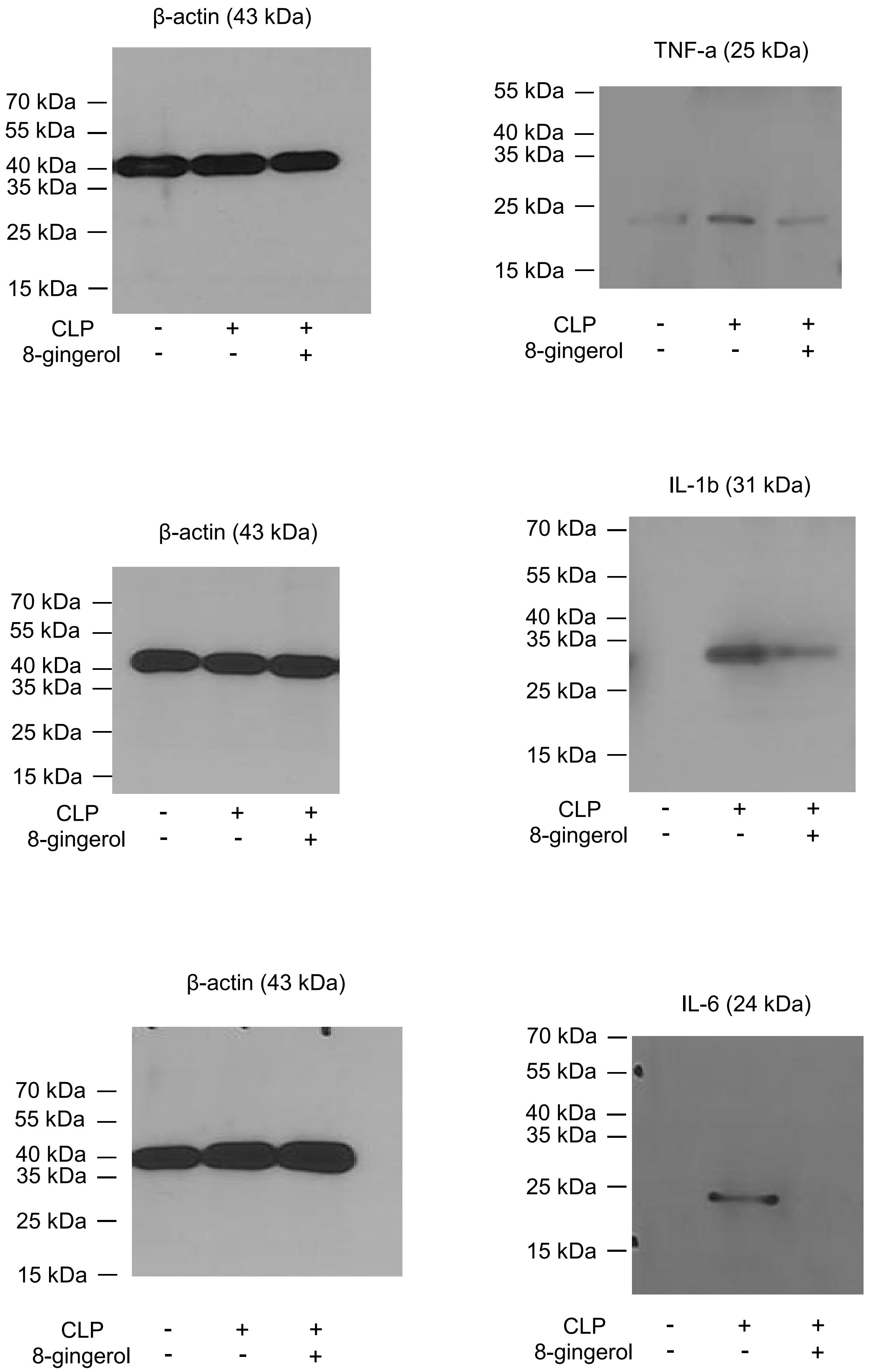

Whole blots for Figure 1I

β-actin (43 kDa)

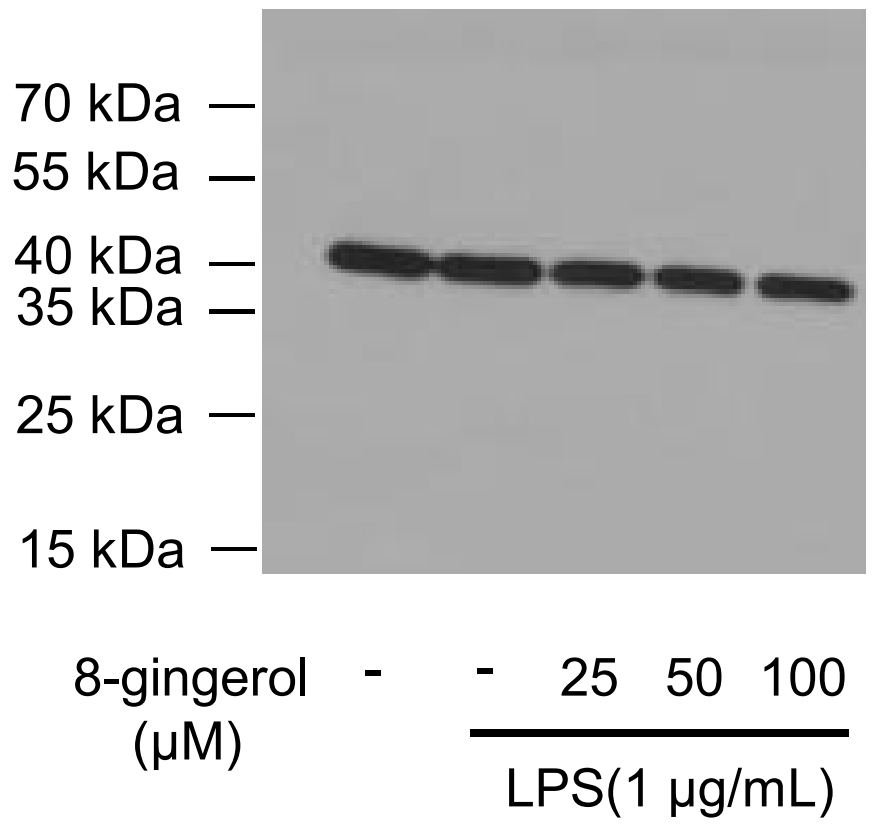

Raw264.7

β-actin (43 kDa)

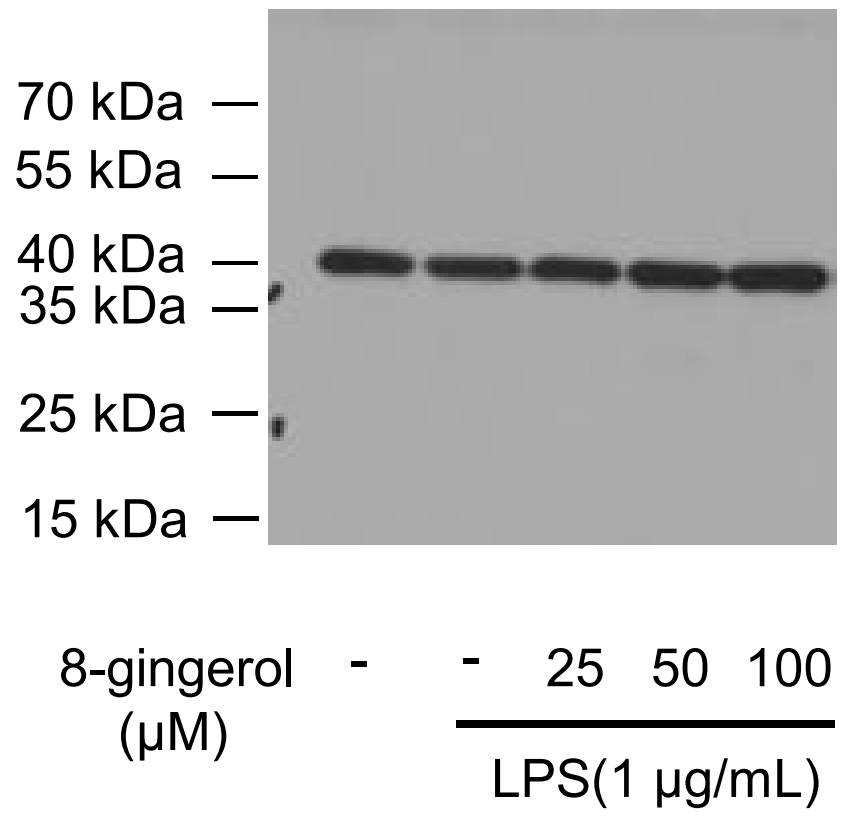

J774A.1

TNF-a (25 kDa)

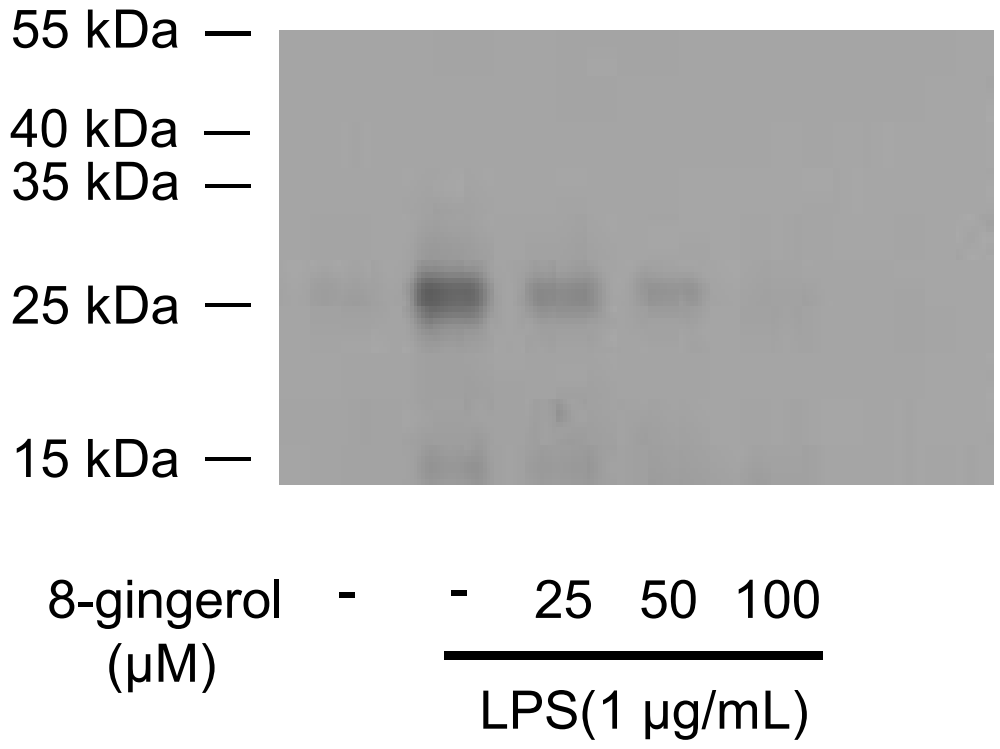

Raw264.7

TNF-a (25 kDa)

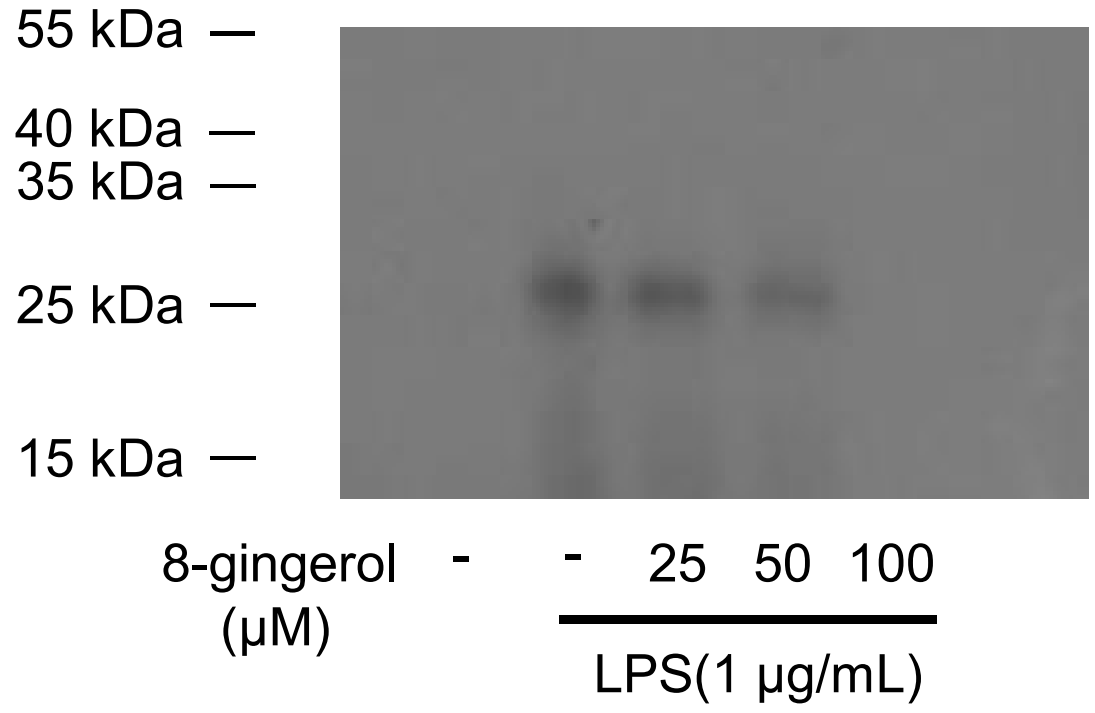

J774A.1

IL-1b (31 kDa)

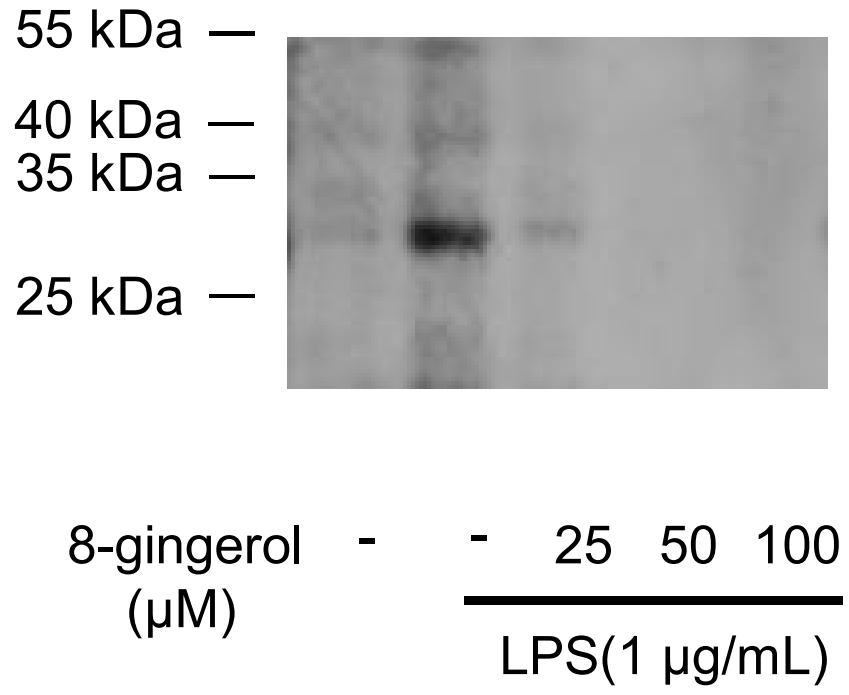

Raw264.7

IL-1b (31 kDa)

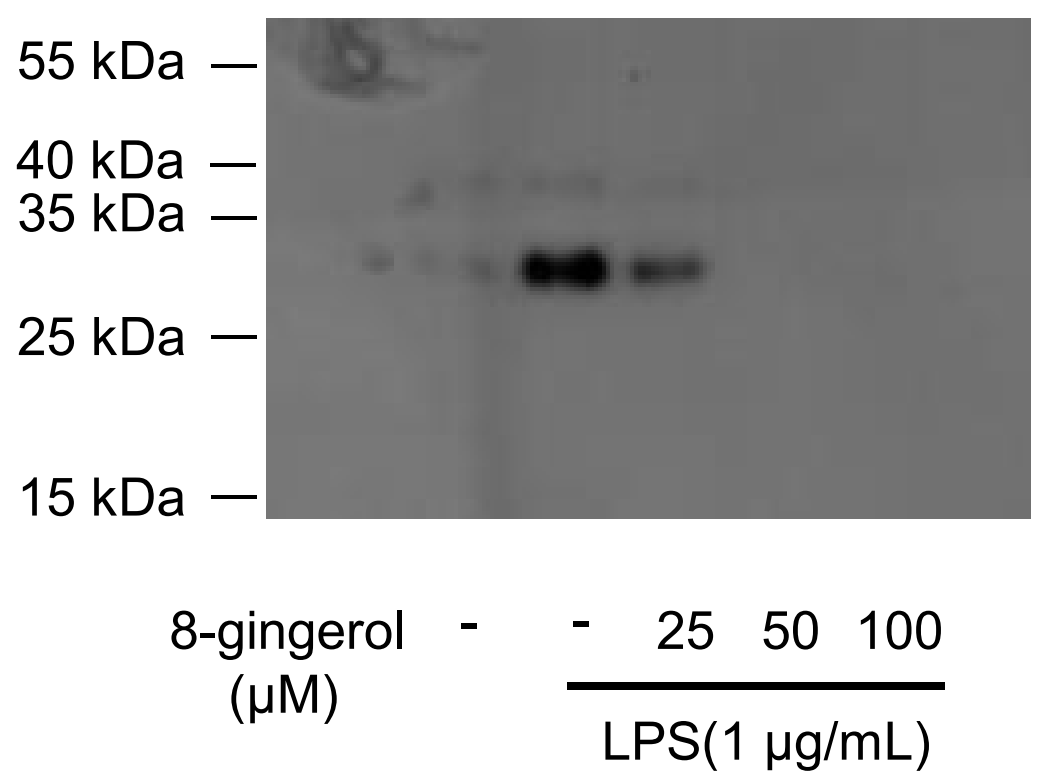

J774A.1

Whole blots for Figure 2H

β-actin (43 kDa)

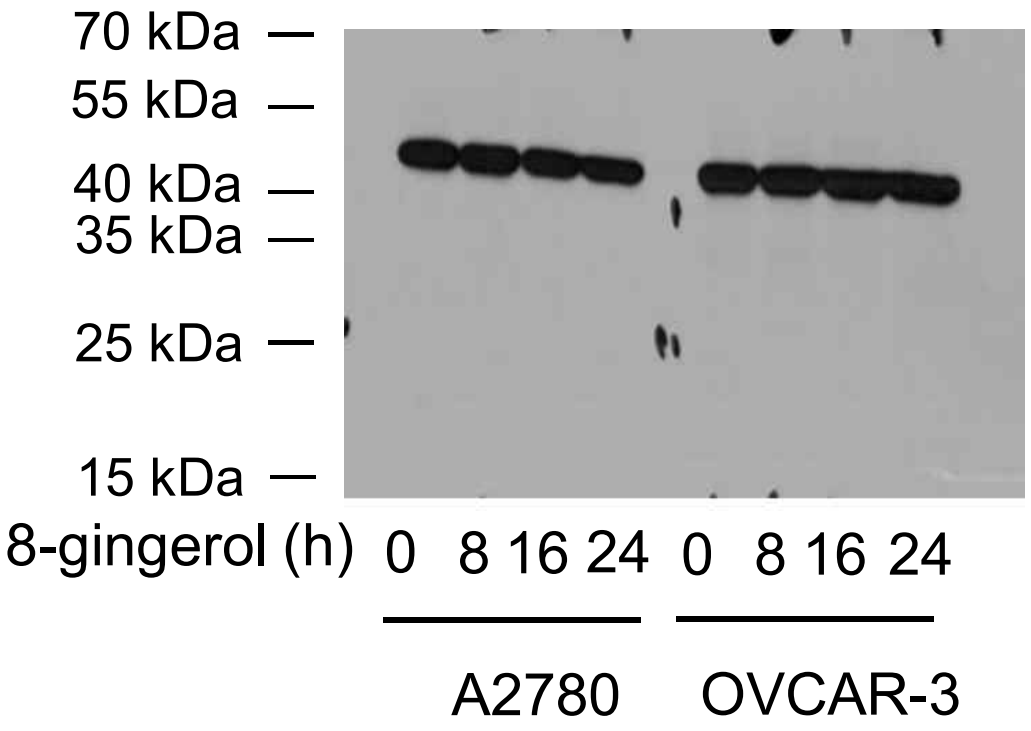

Bcl-2 (28 kDa)

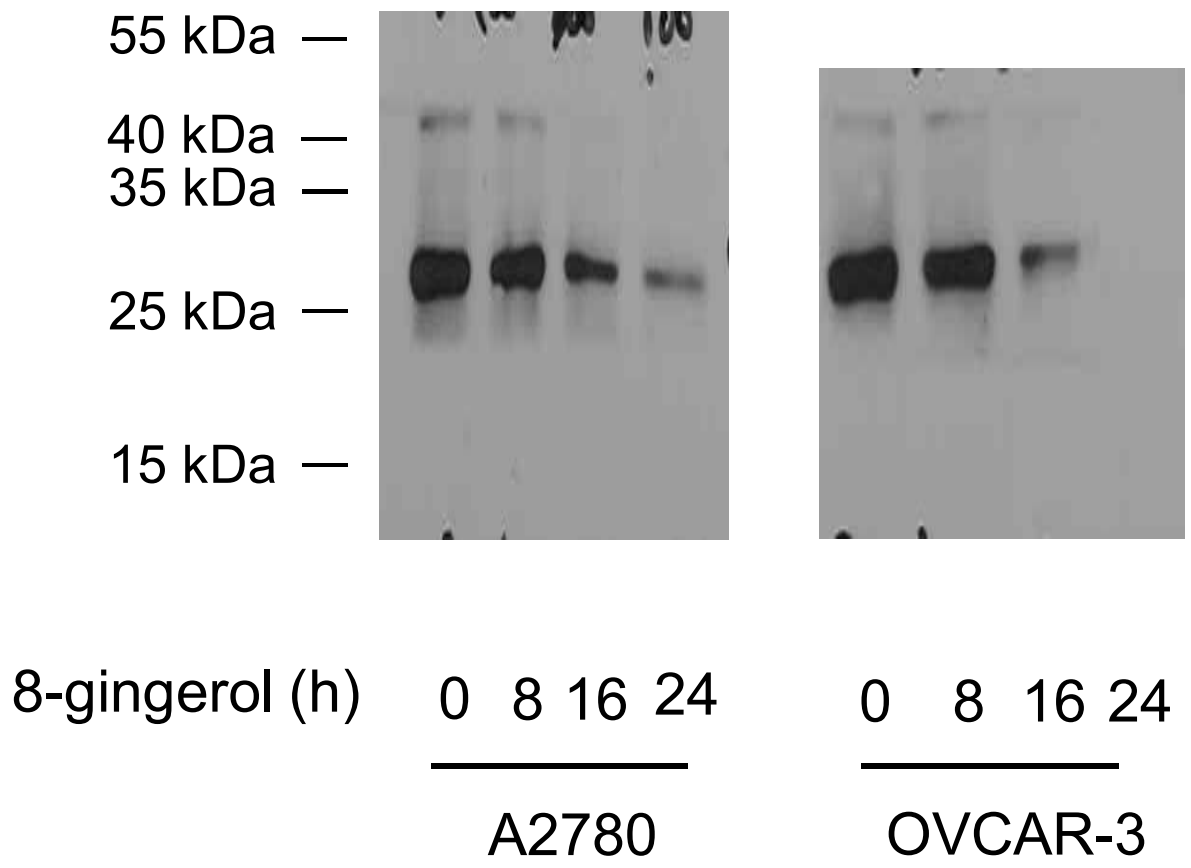

Cleaved caspase-3 (17-19 kDa)

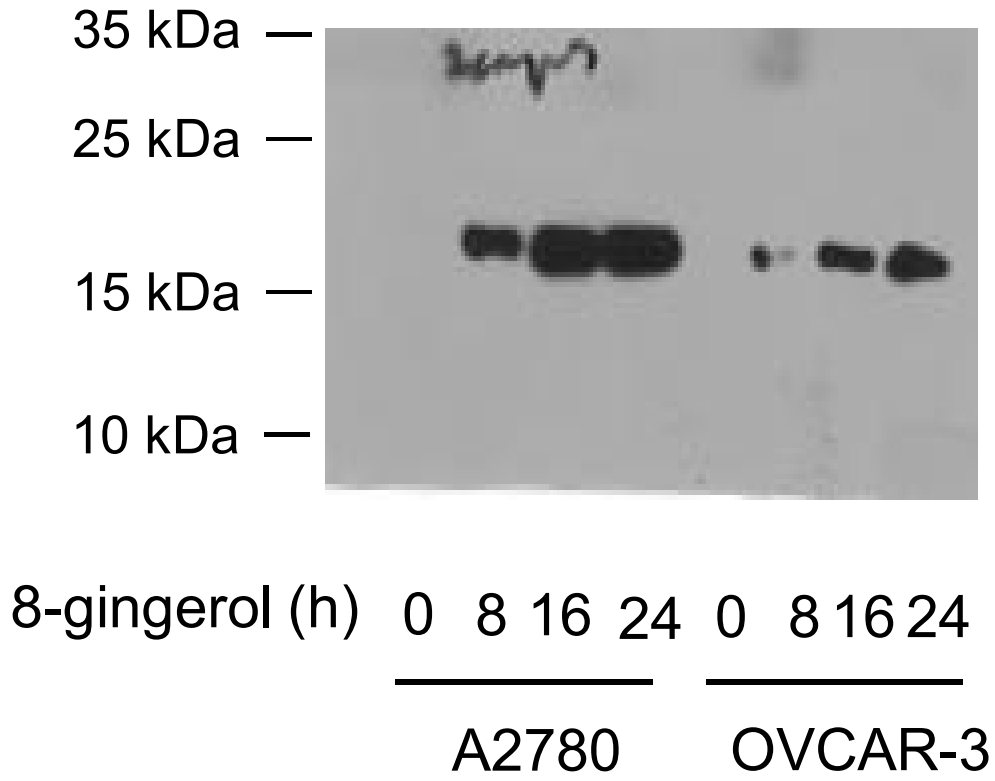

Cleaved caspase-9 (37 kDa)

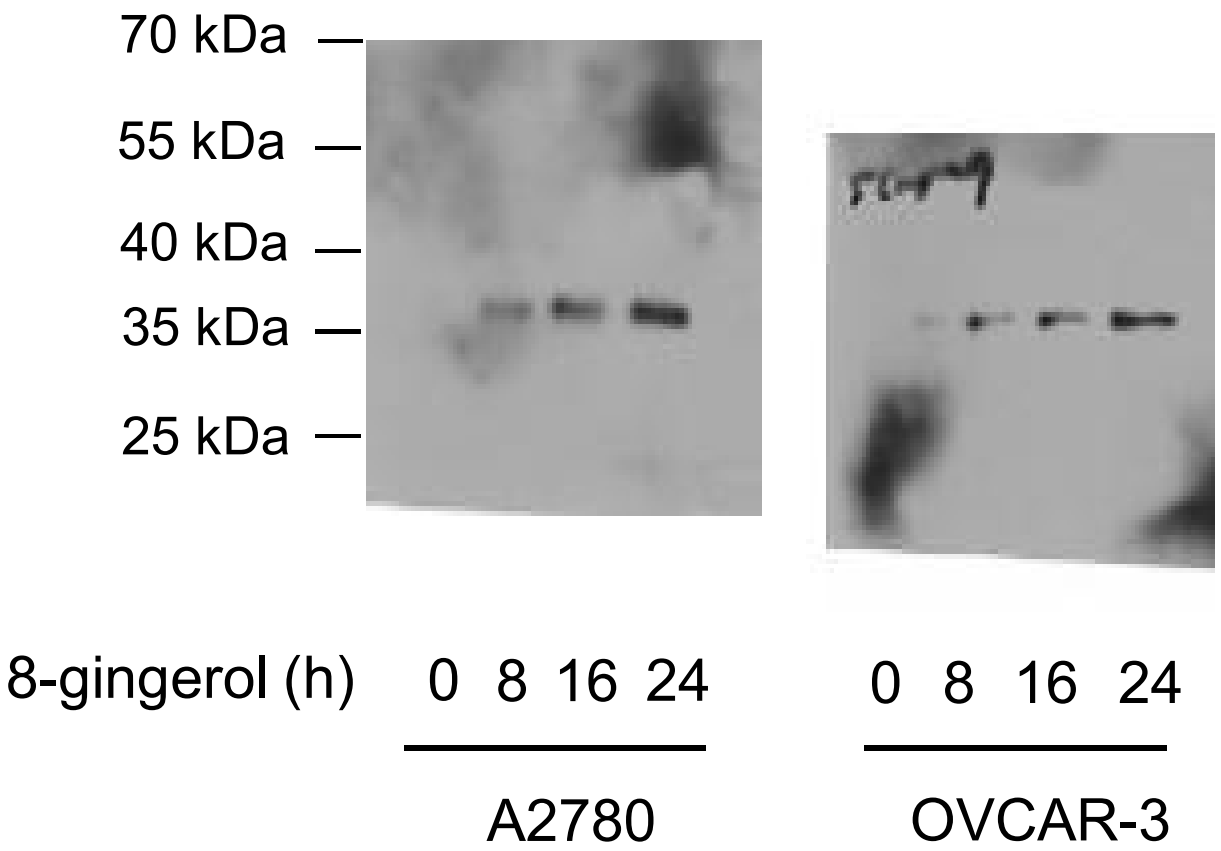

Whole blots for Figure 2H

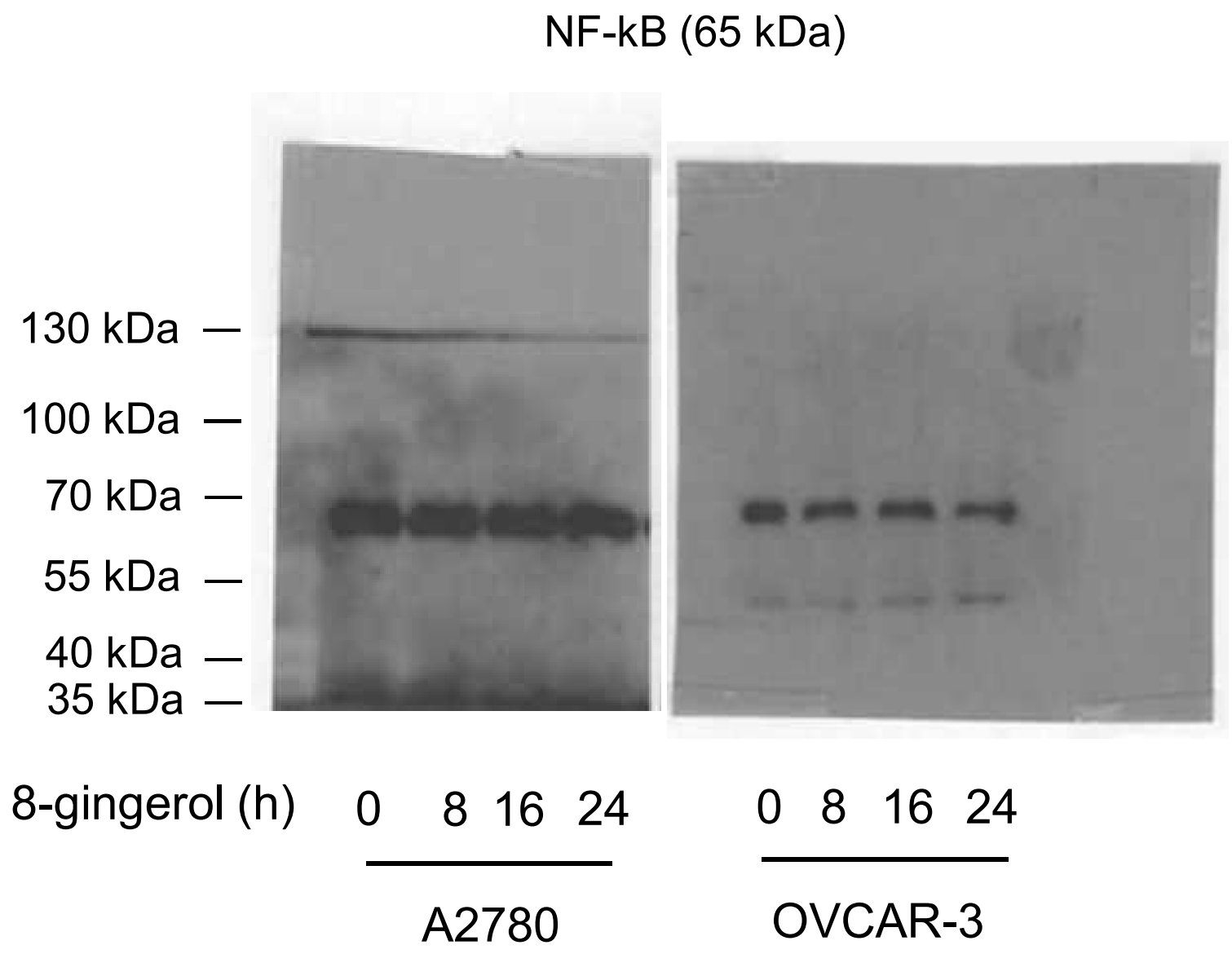

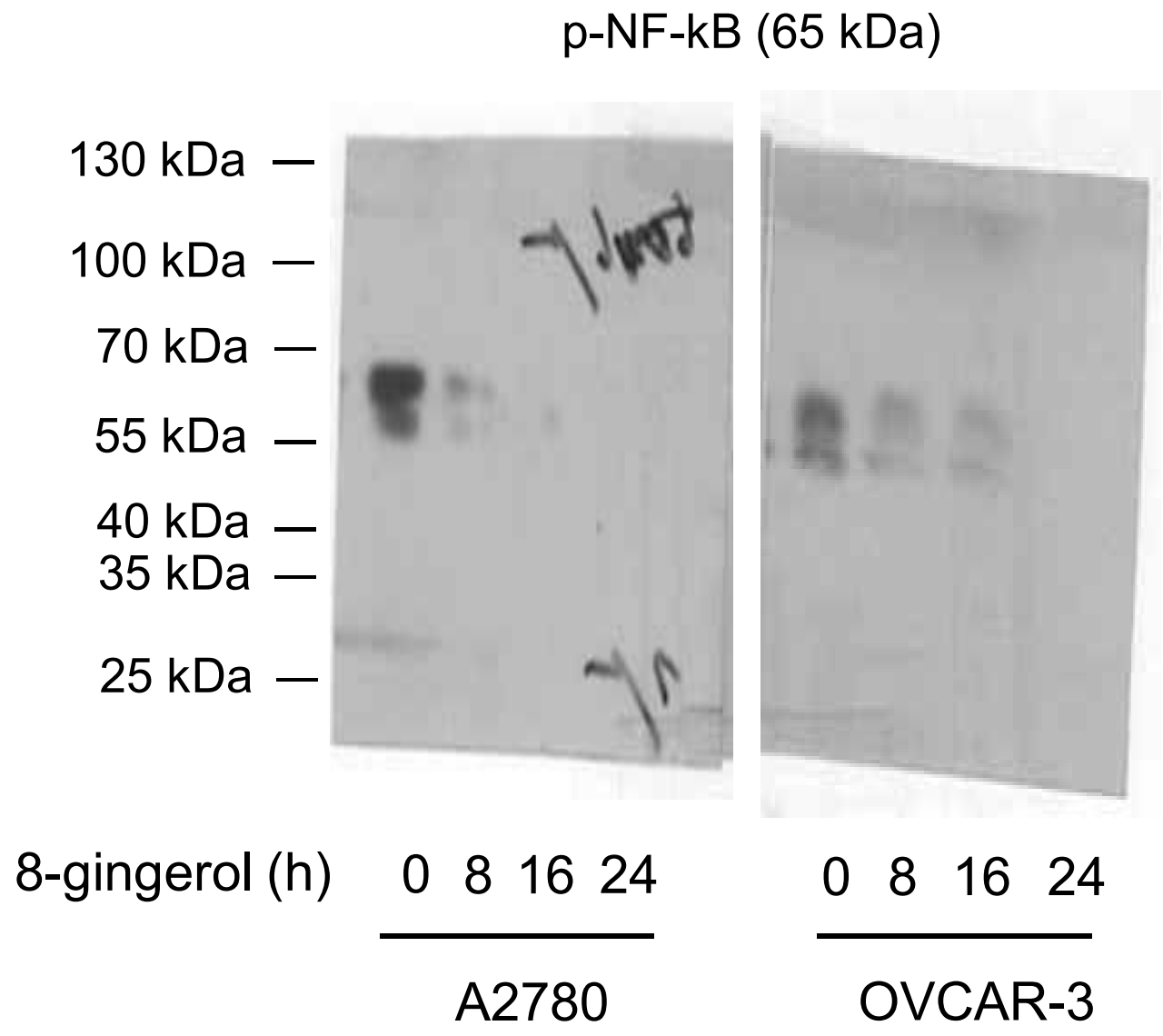

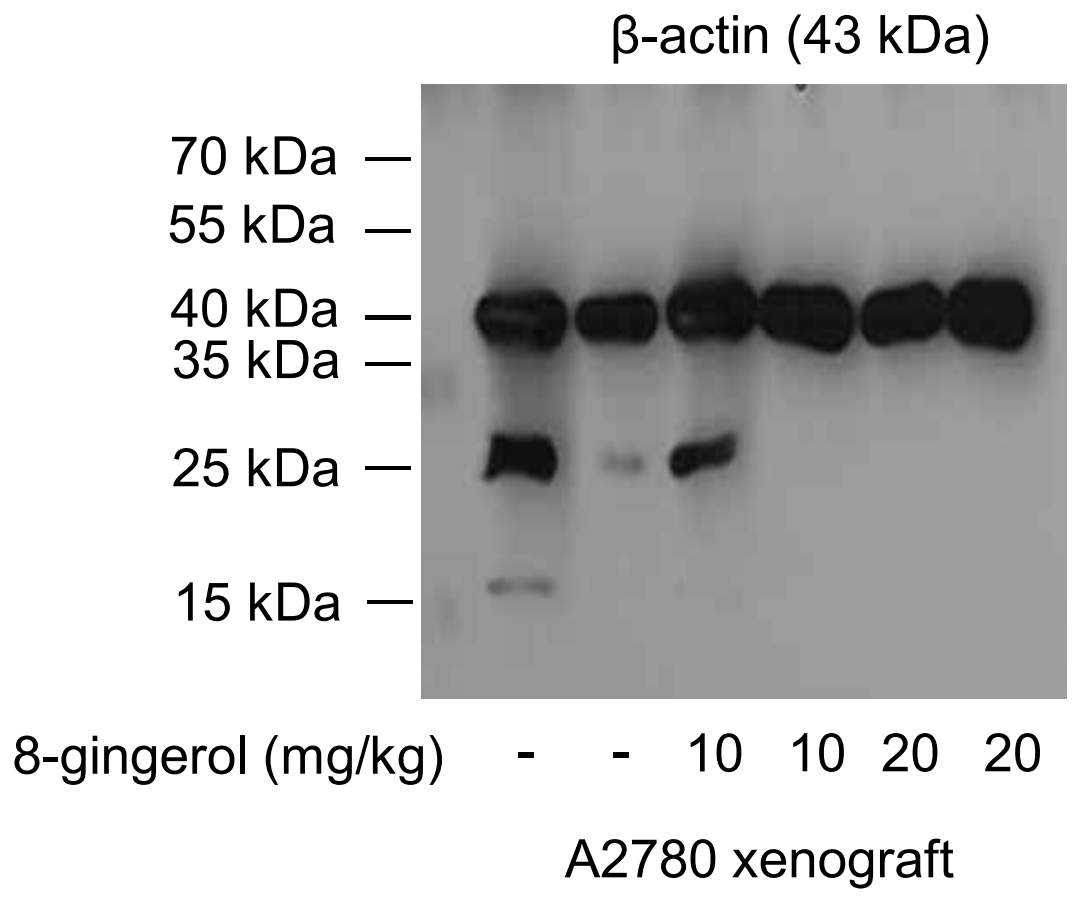

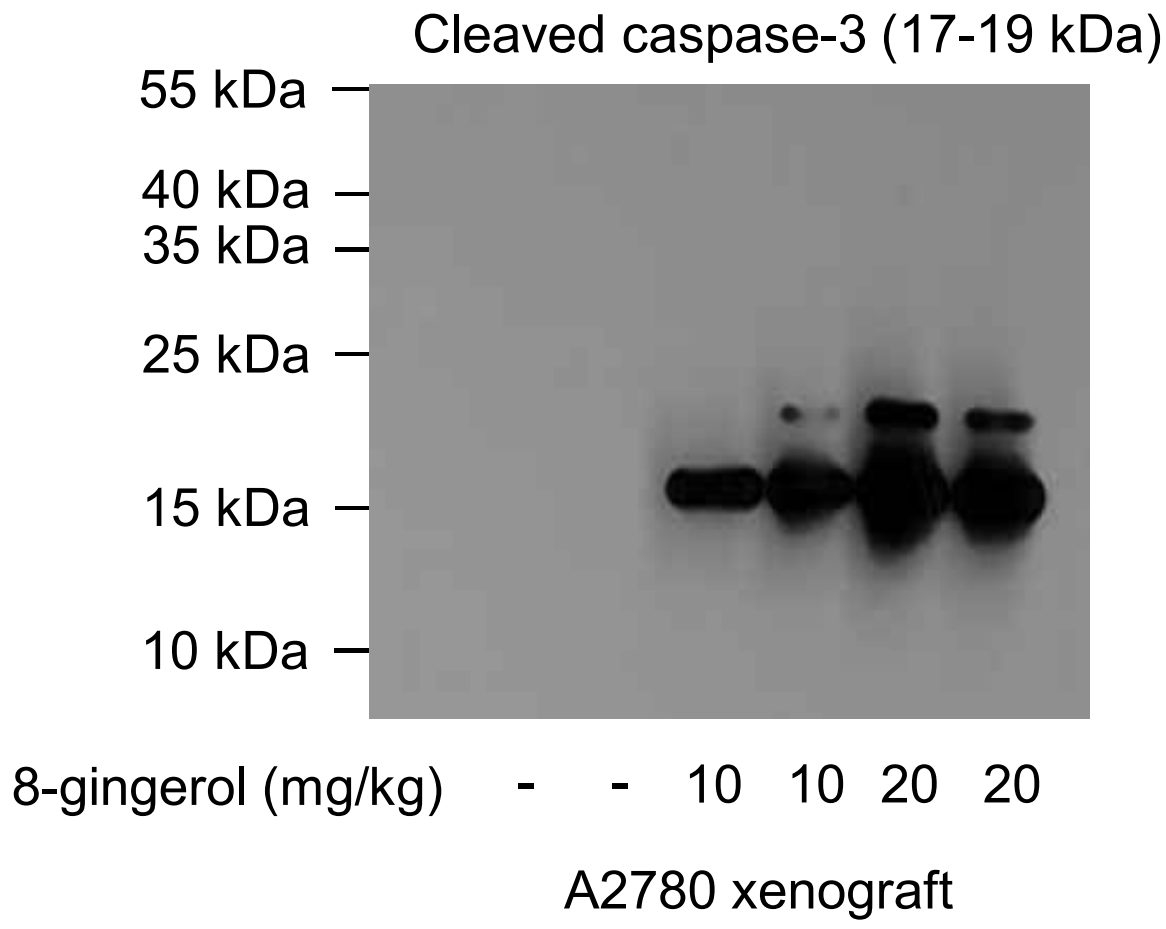

Whole blots for Figure 2L

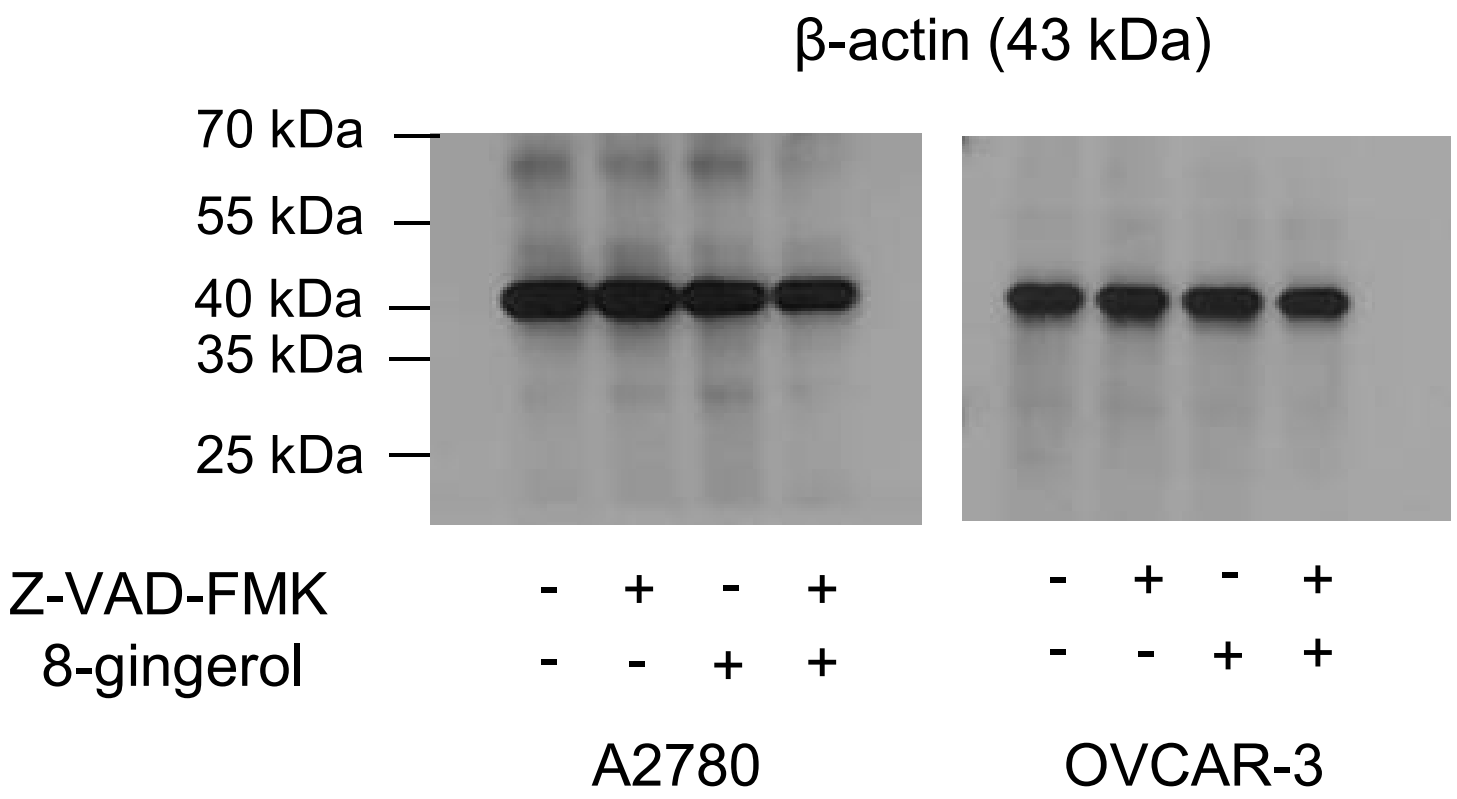

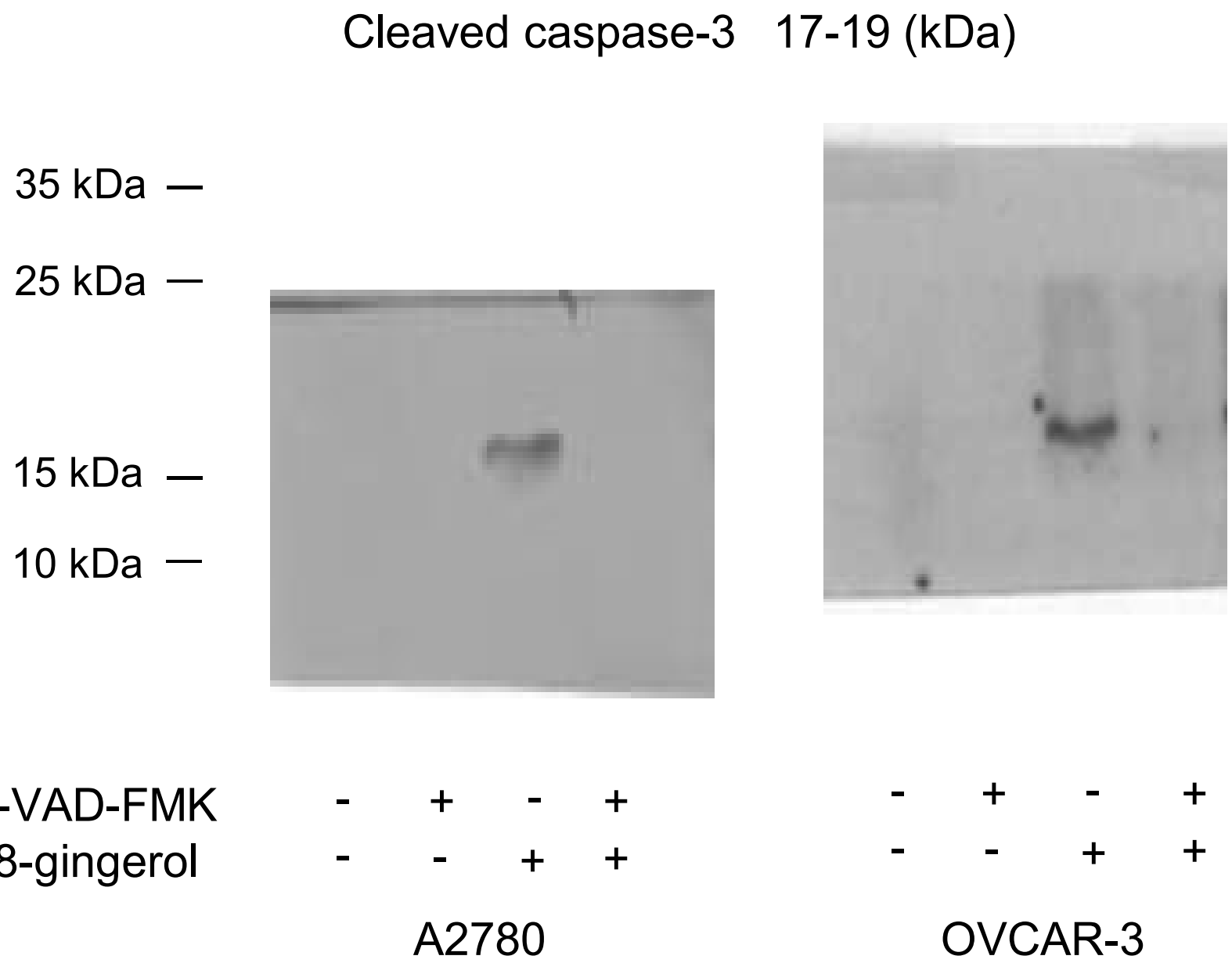

Whole blots for Figure 3C

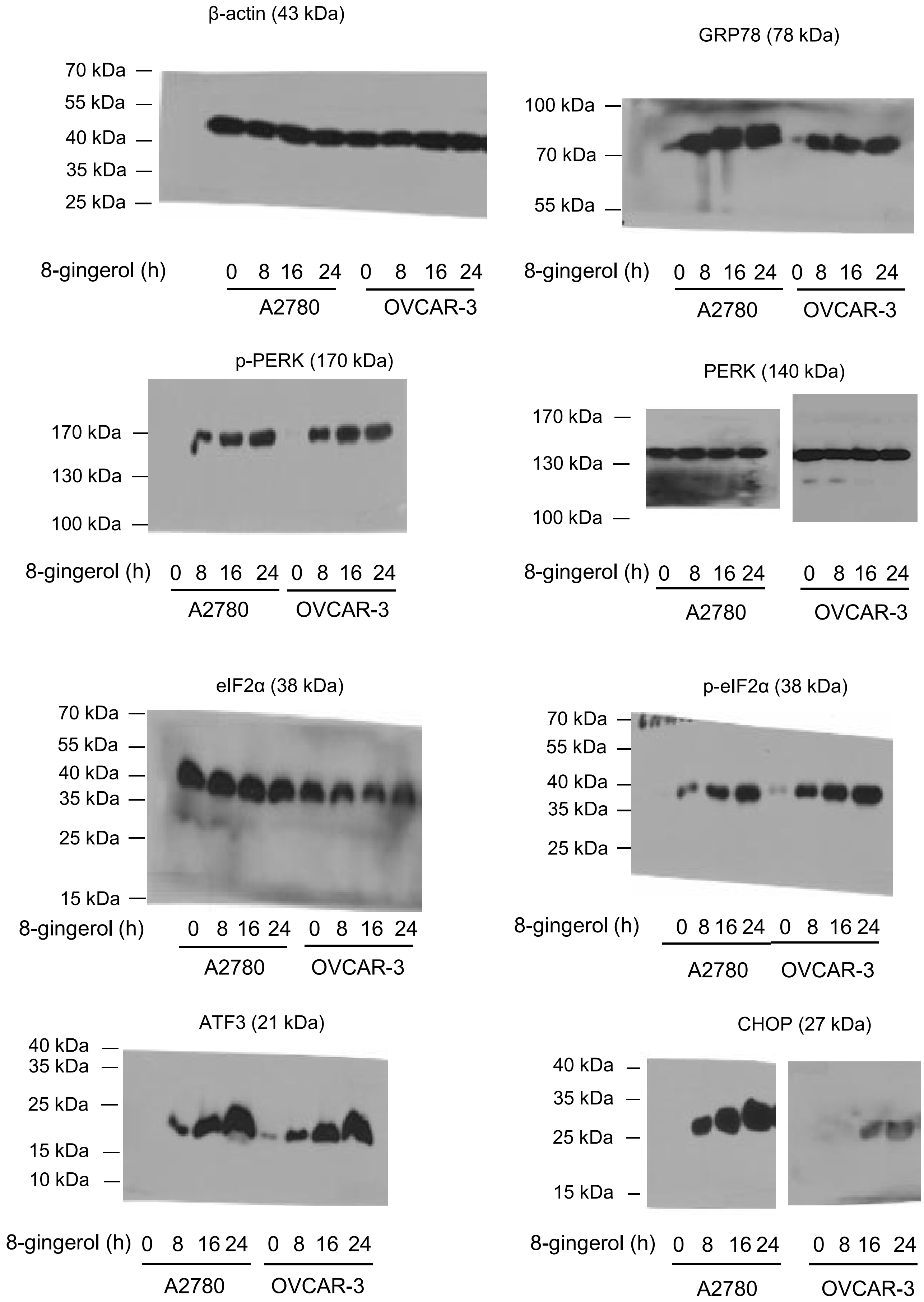

Whole blots for Figure 3D

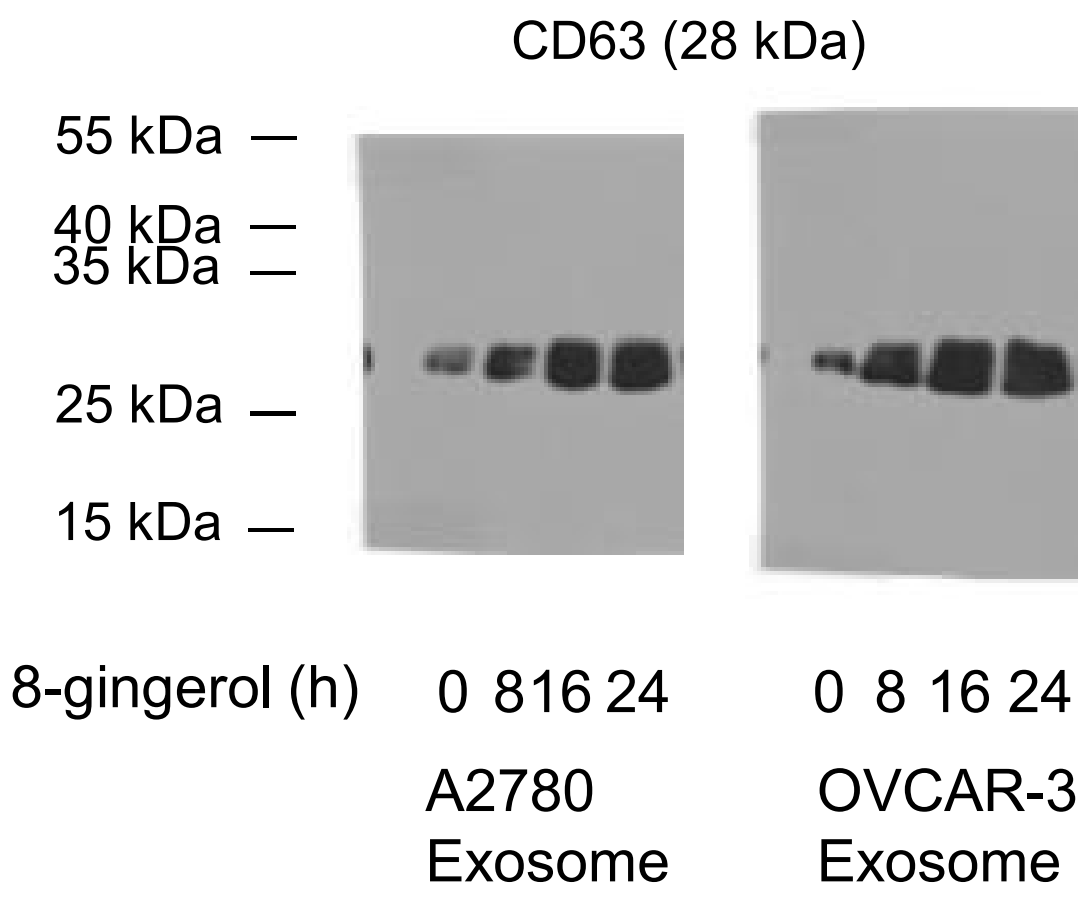

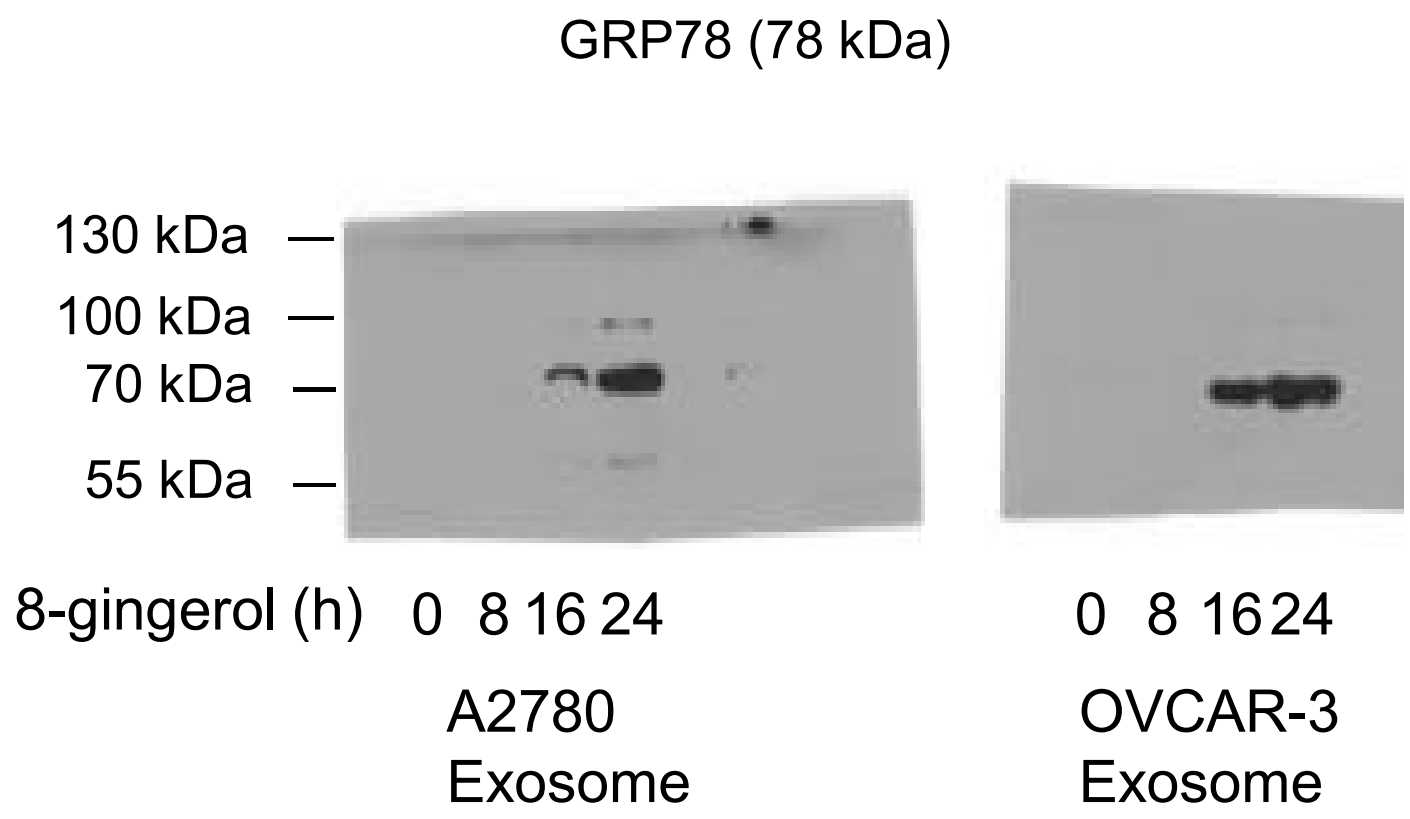

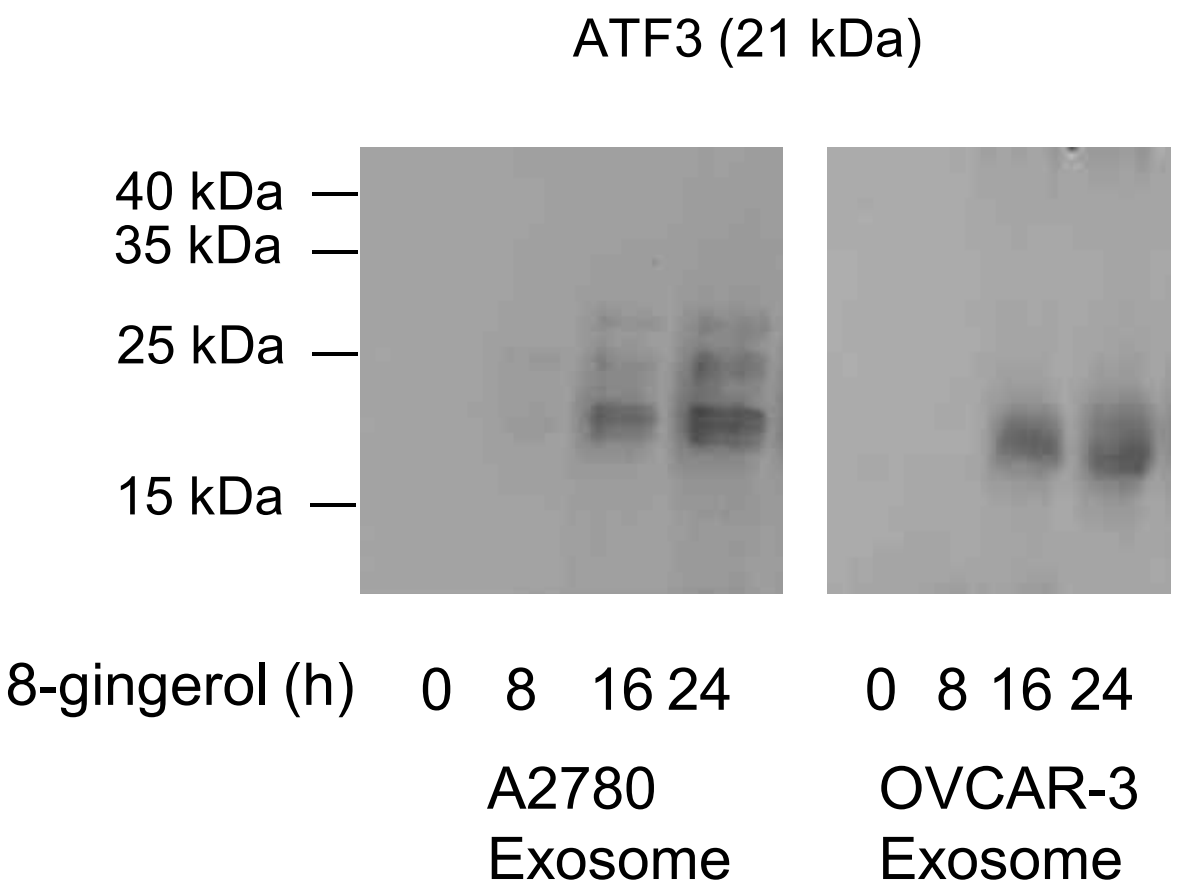

Whole blots for Figure 3D

β-actin (43 kDa)

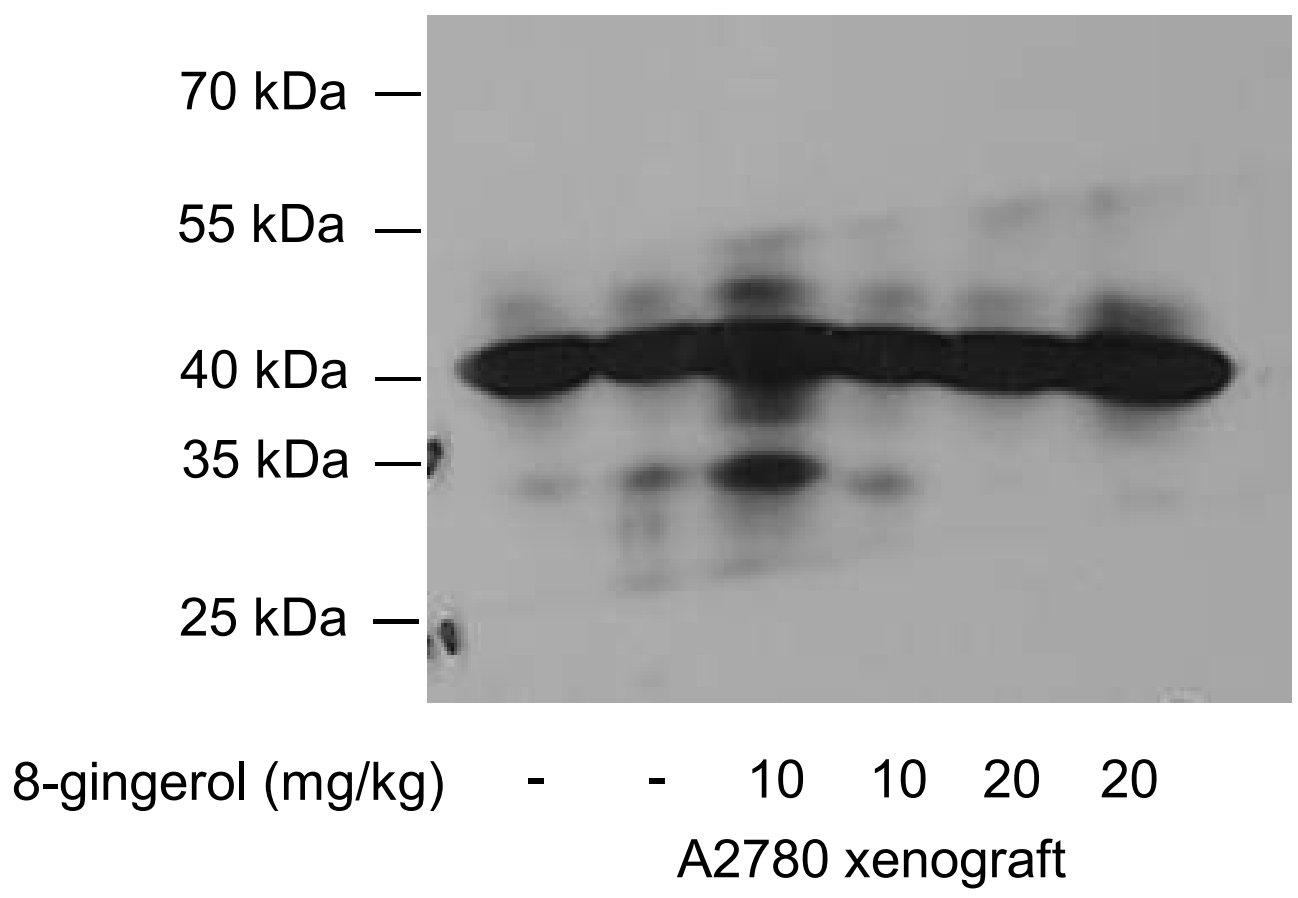

ATF3 (21 kDa)

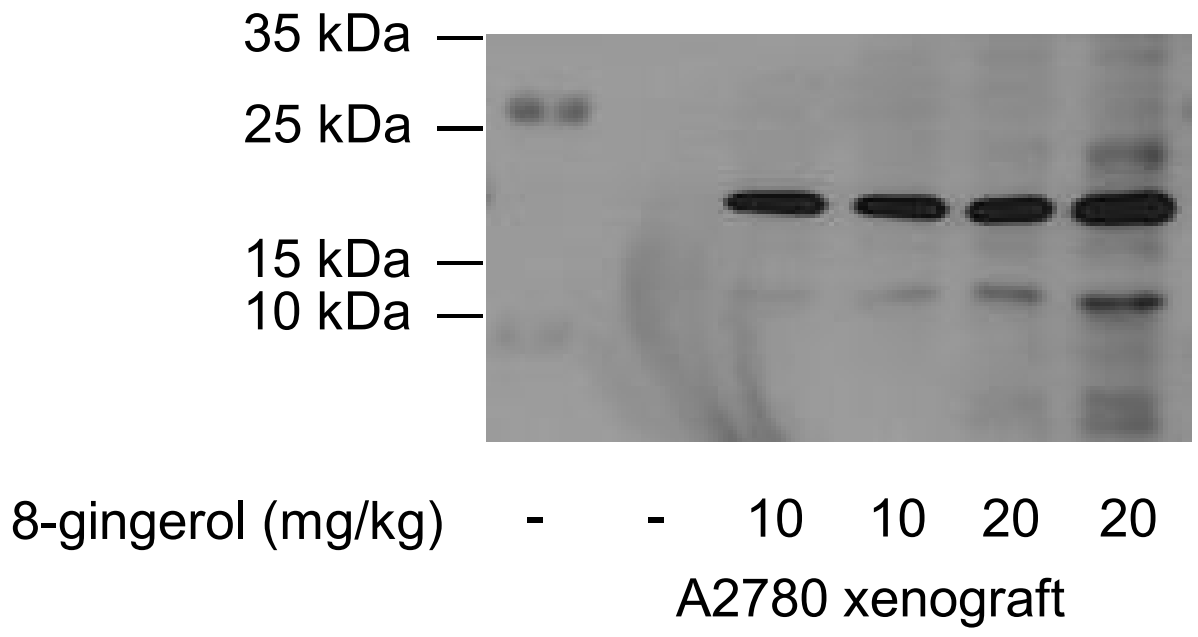

p-PERK (170 kDa)

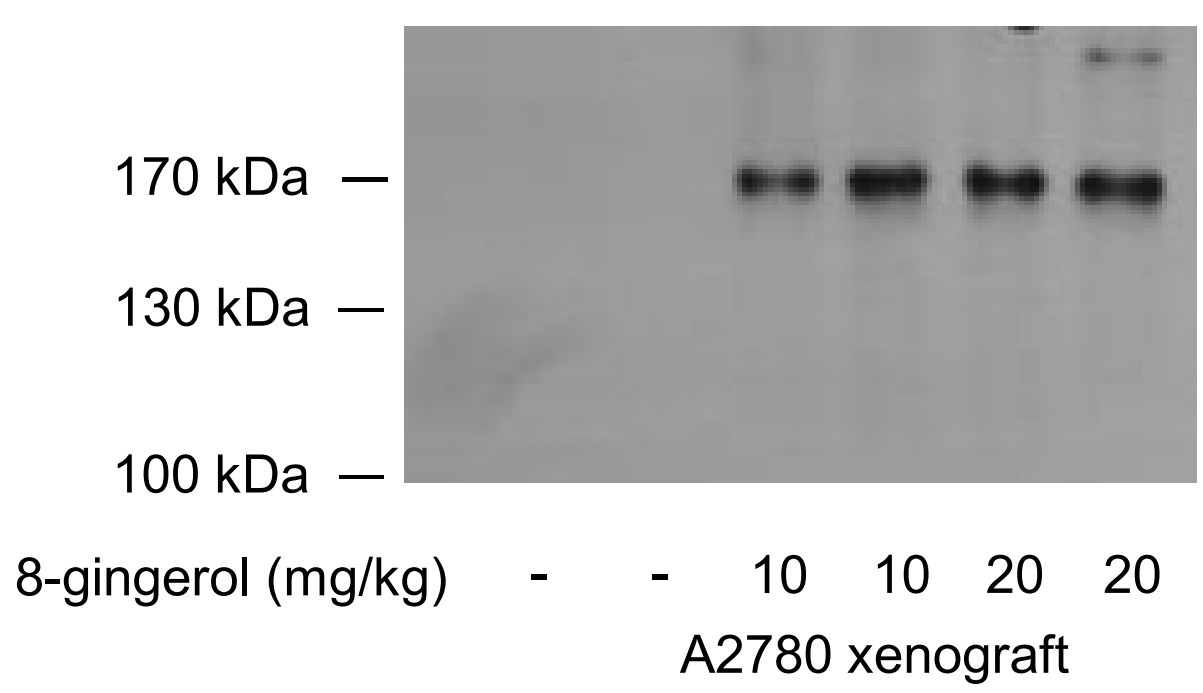

PERK (140 kDa)

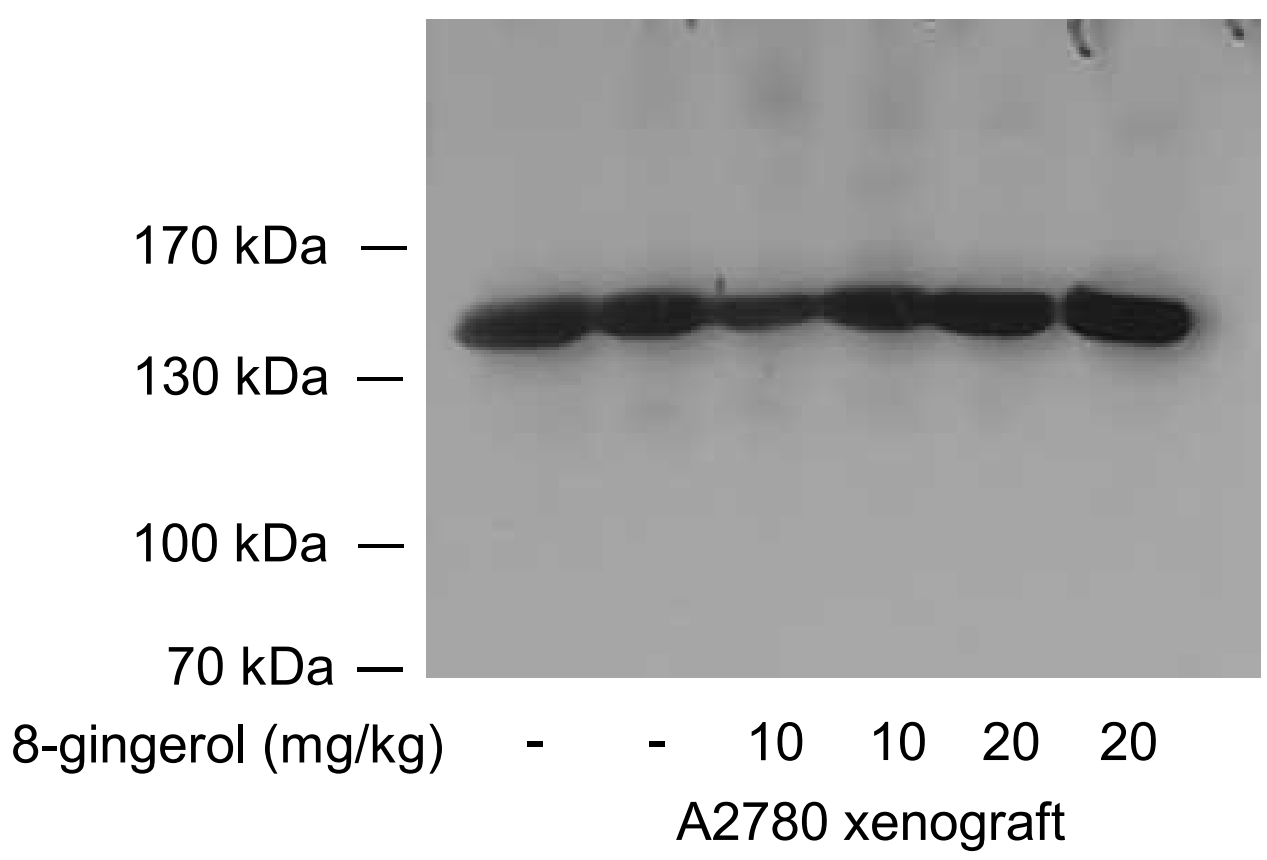

Whole blots for Figure 3H

β-actin (43 kDa)

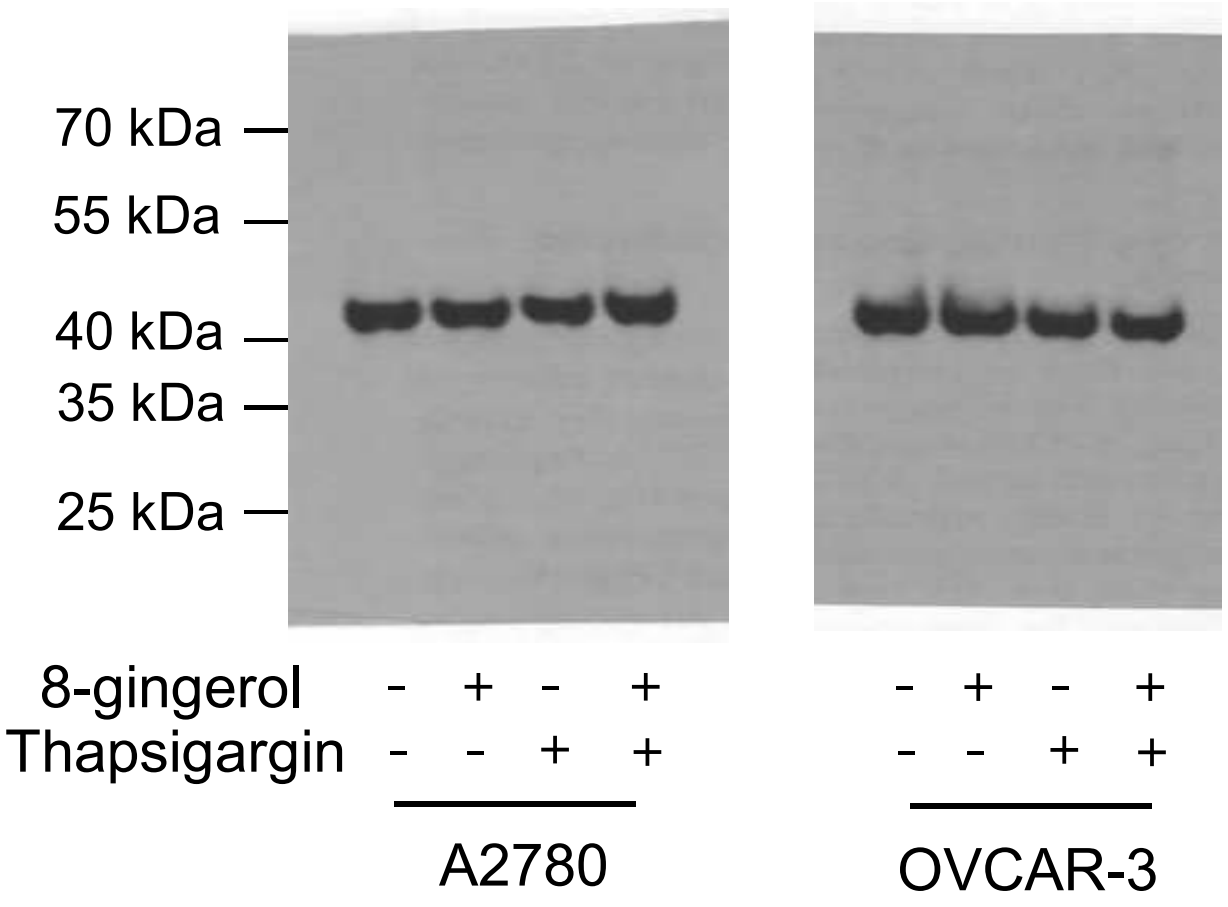

p-PERK (170 kDa)

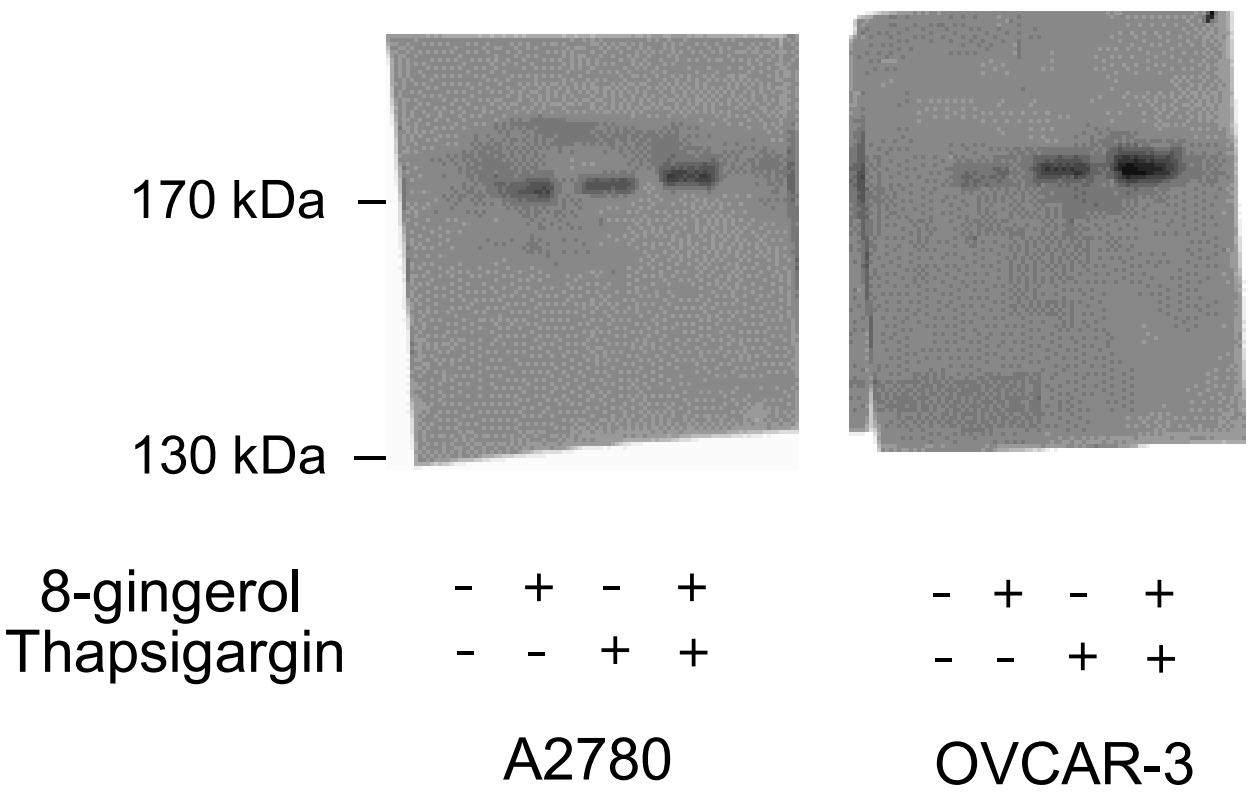

PERK (140 kDa)

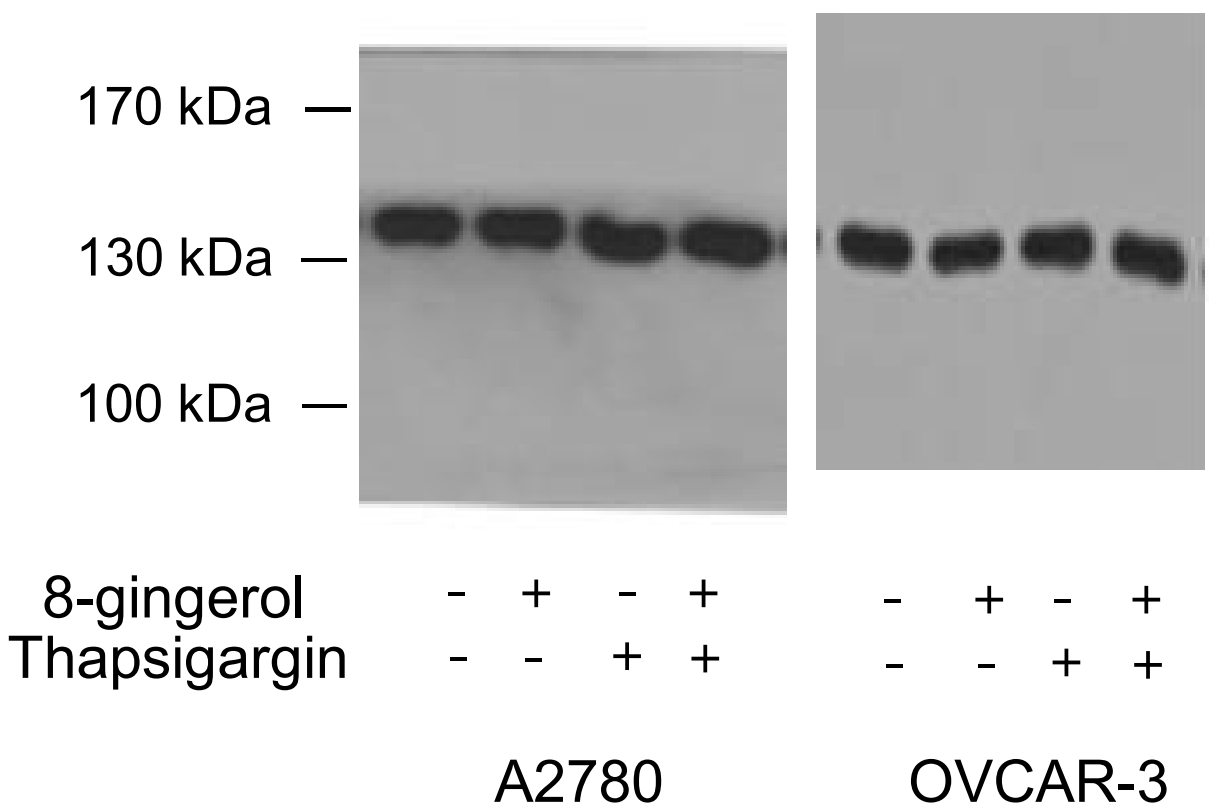

ATF3 (21 kDa)

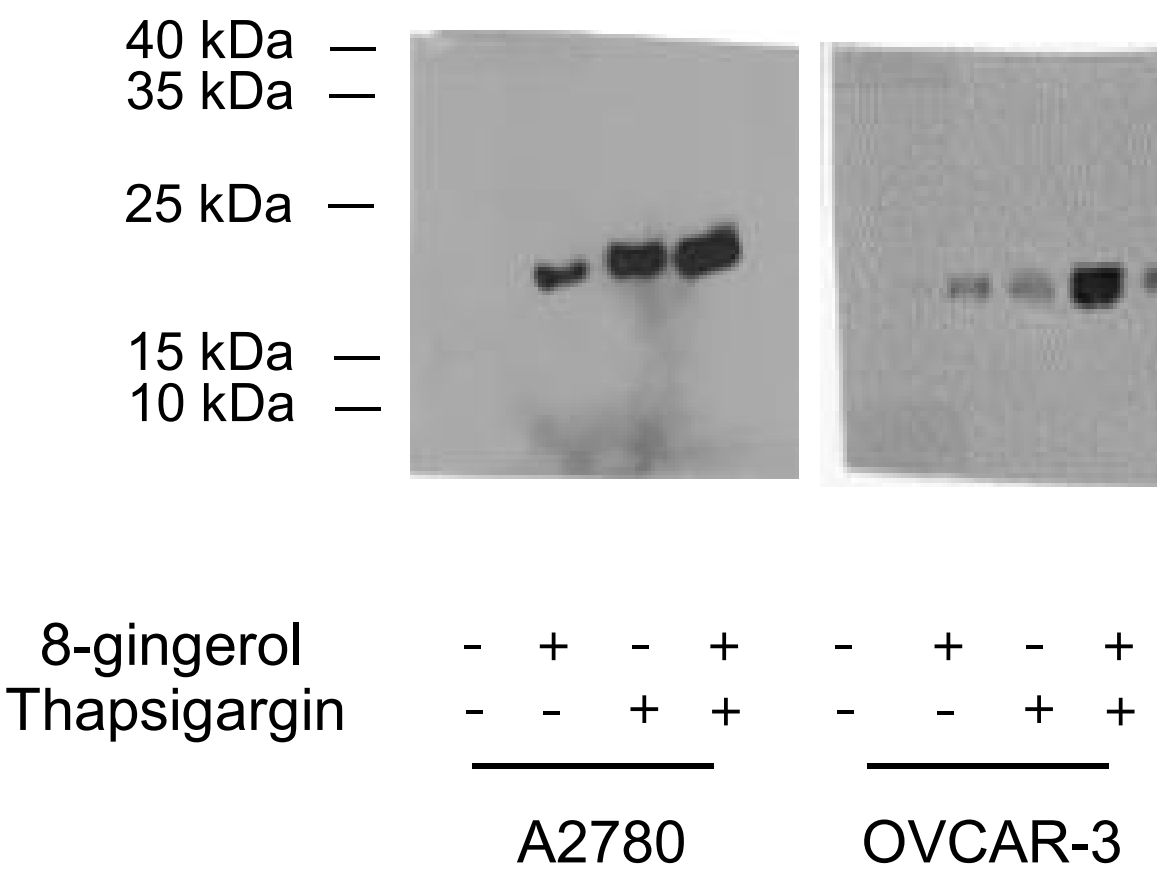

Cleaved caspase-3 (17-19 kDa)

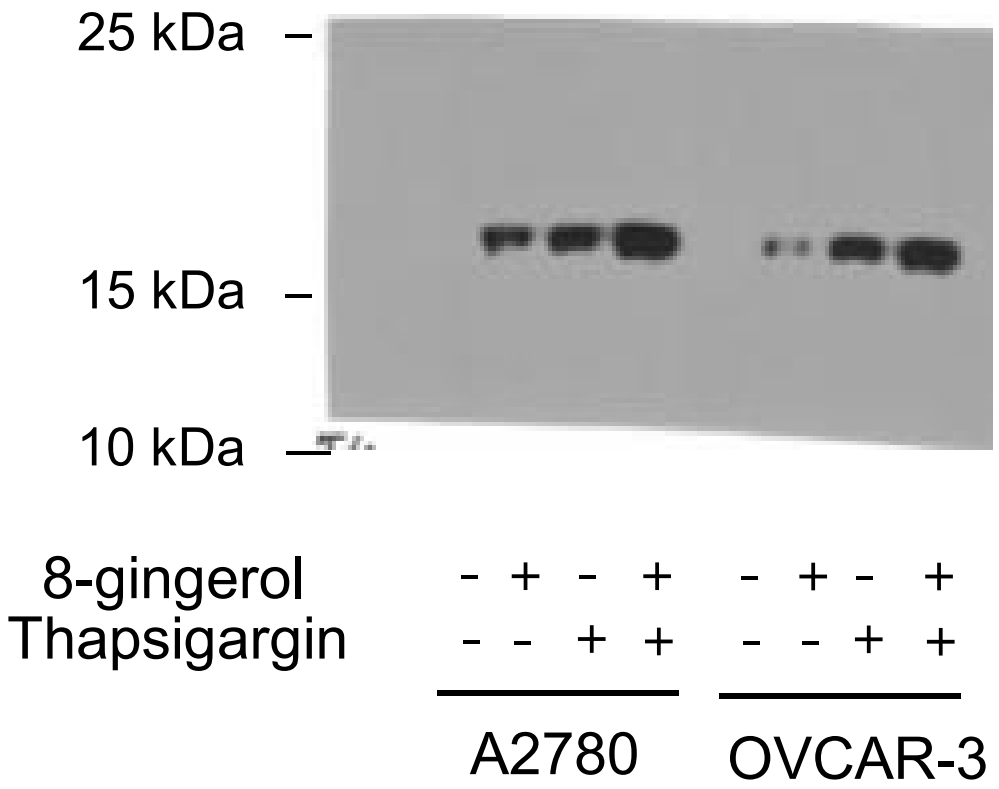

Whole blots for Figure 3L

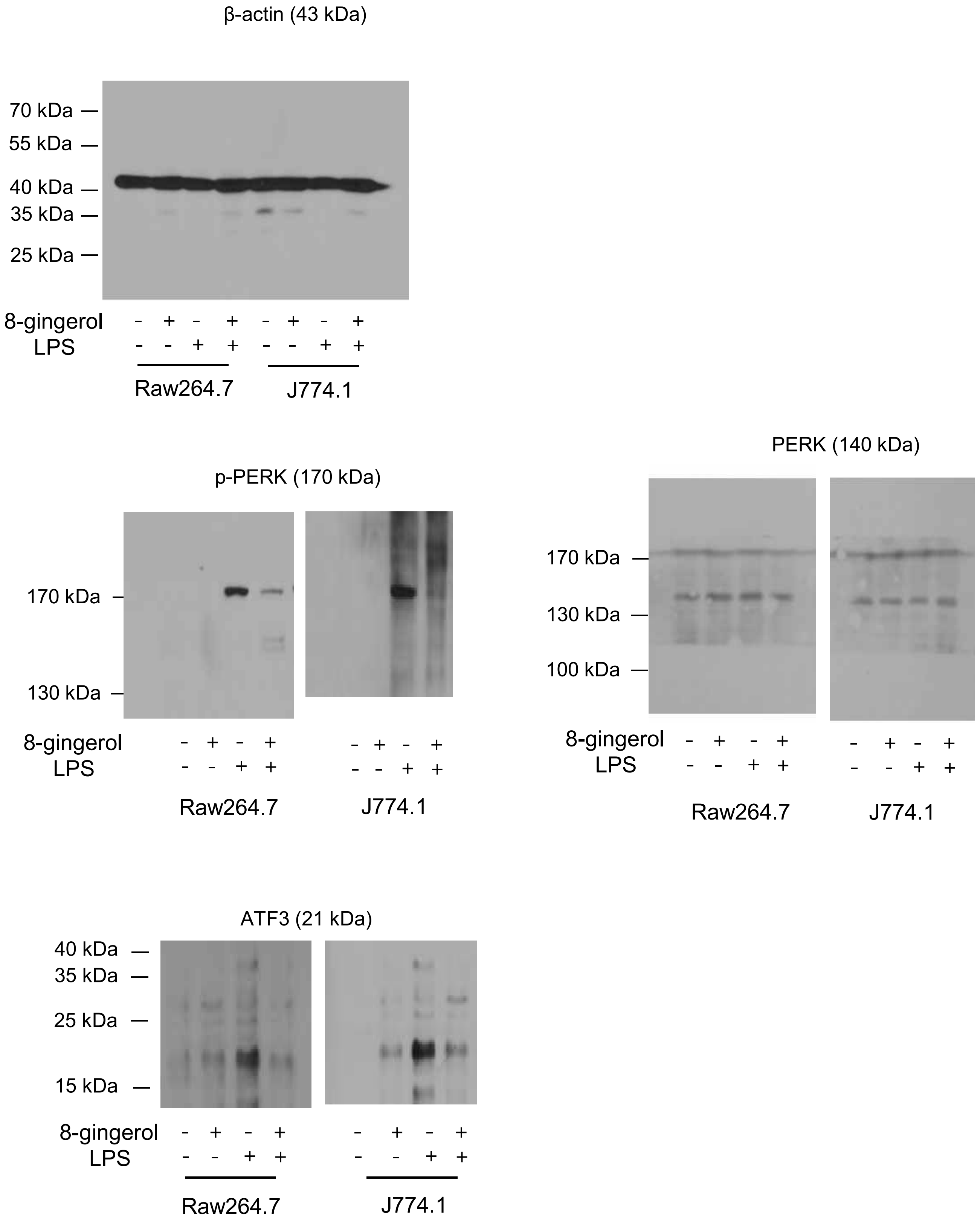

Whole blots for Figure 4C

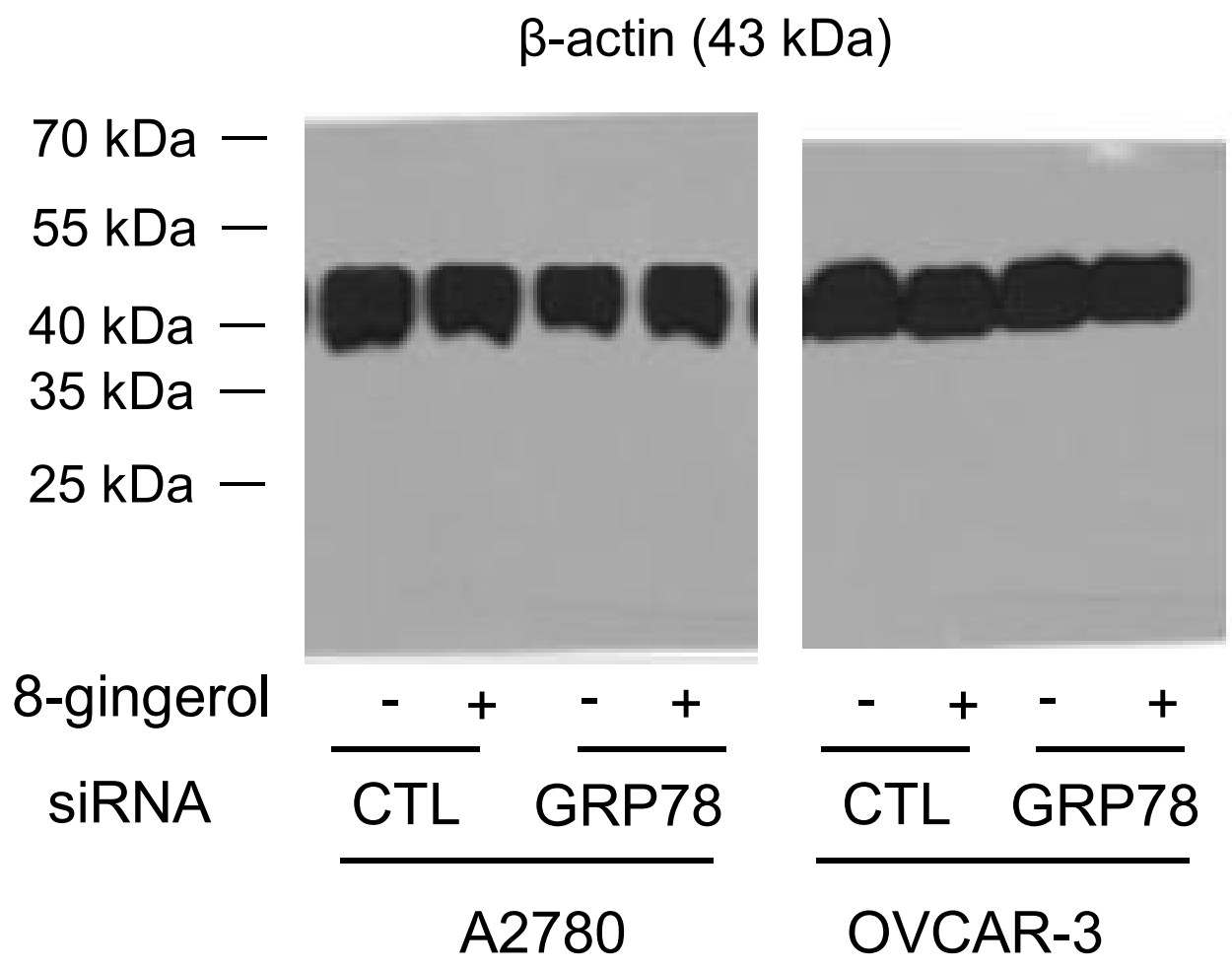

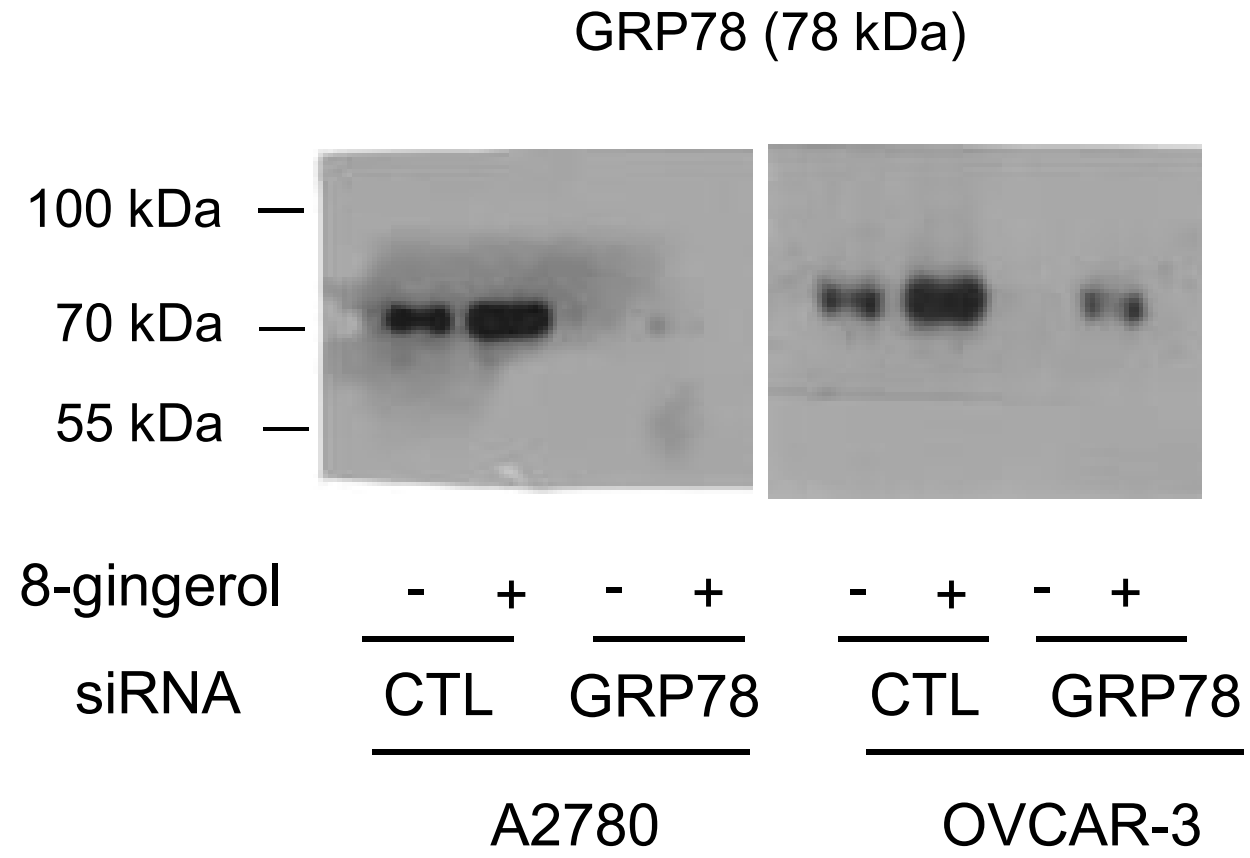

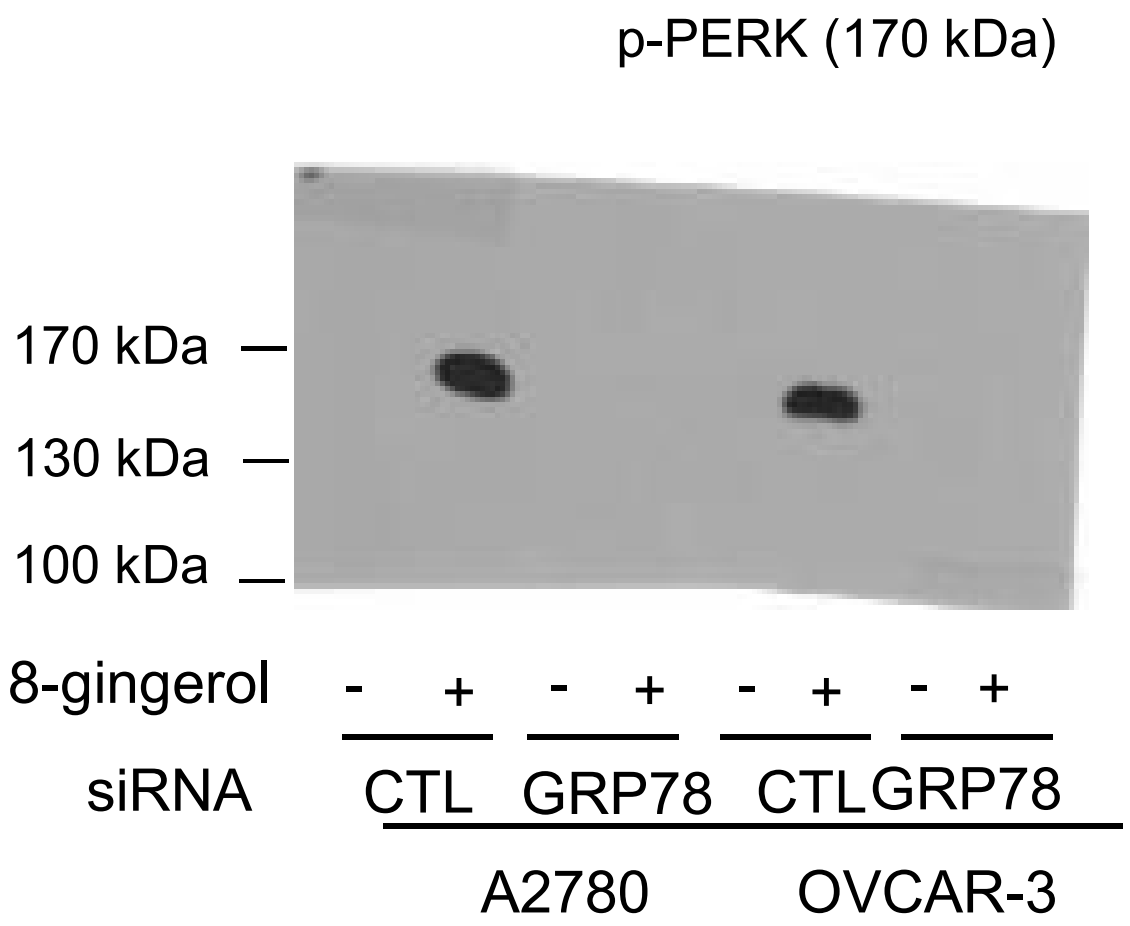

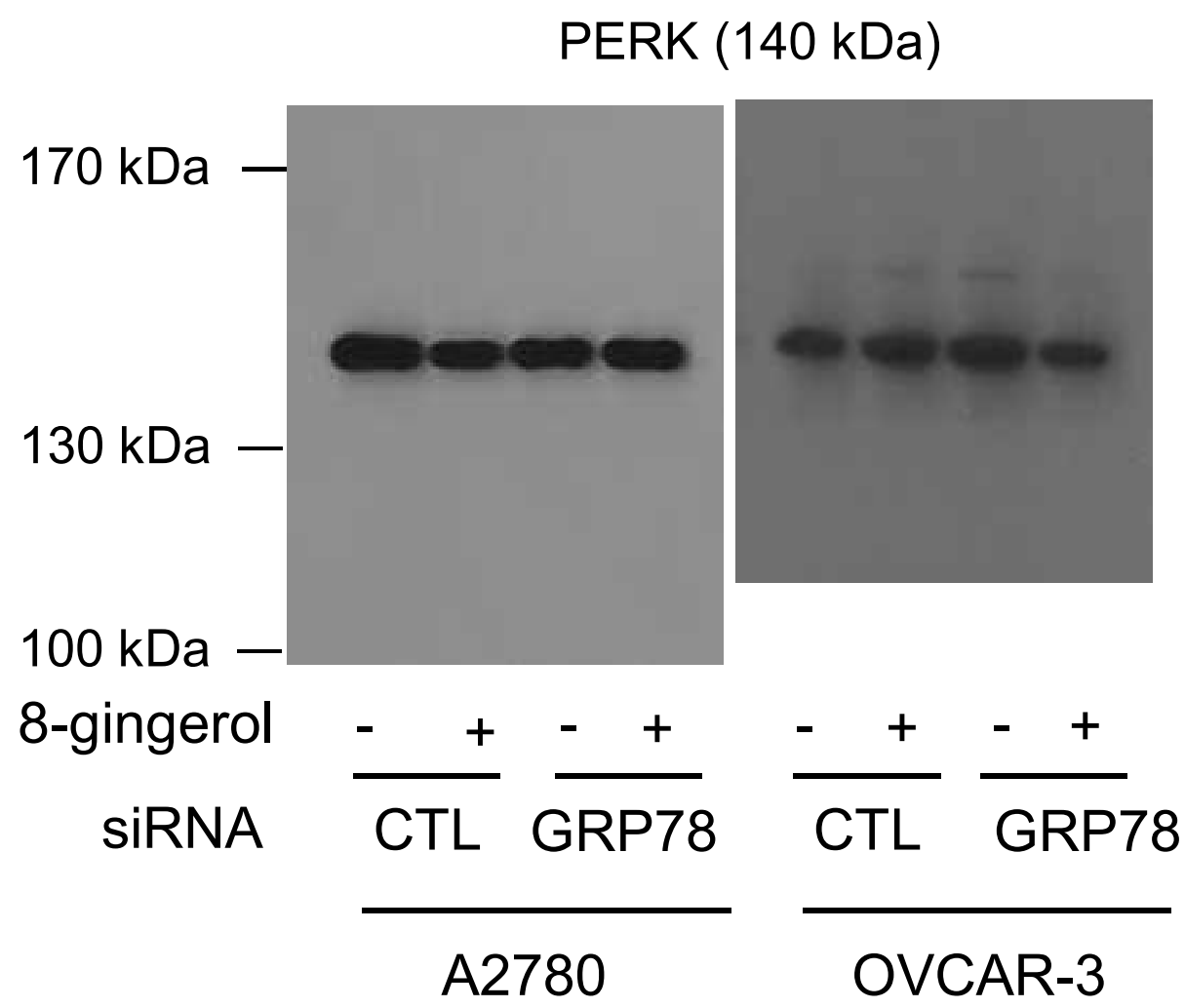

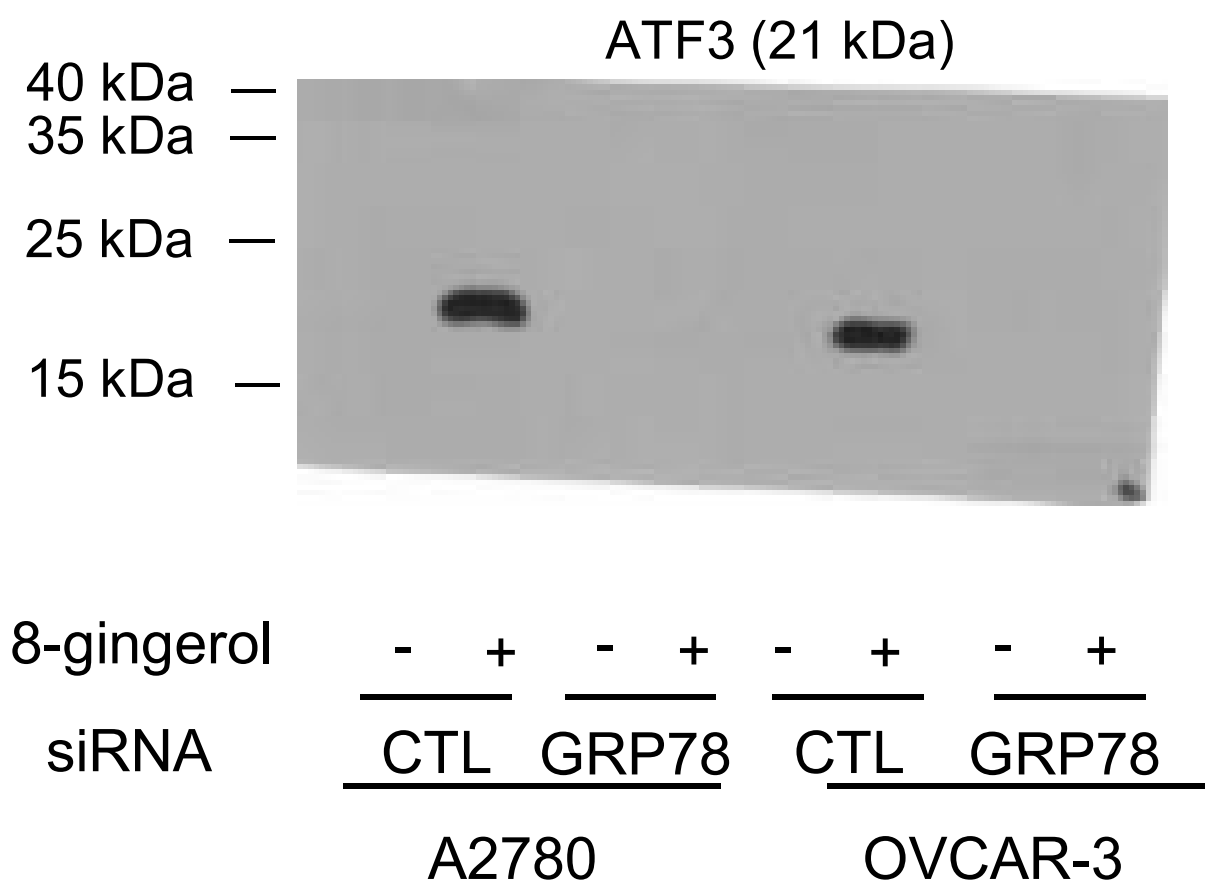

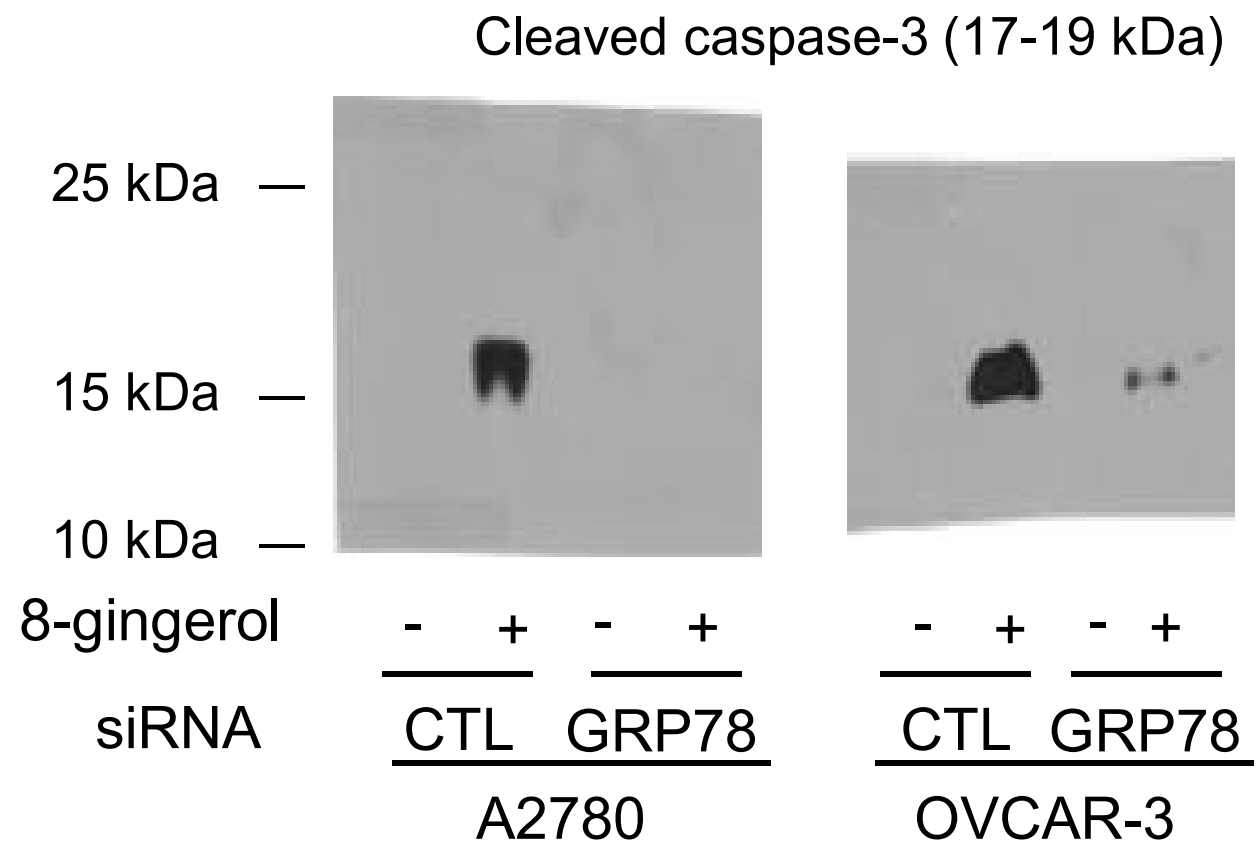

Whole blots for Figure 4F

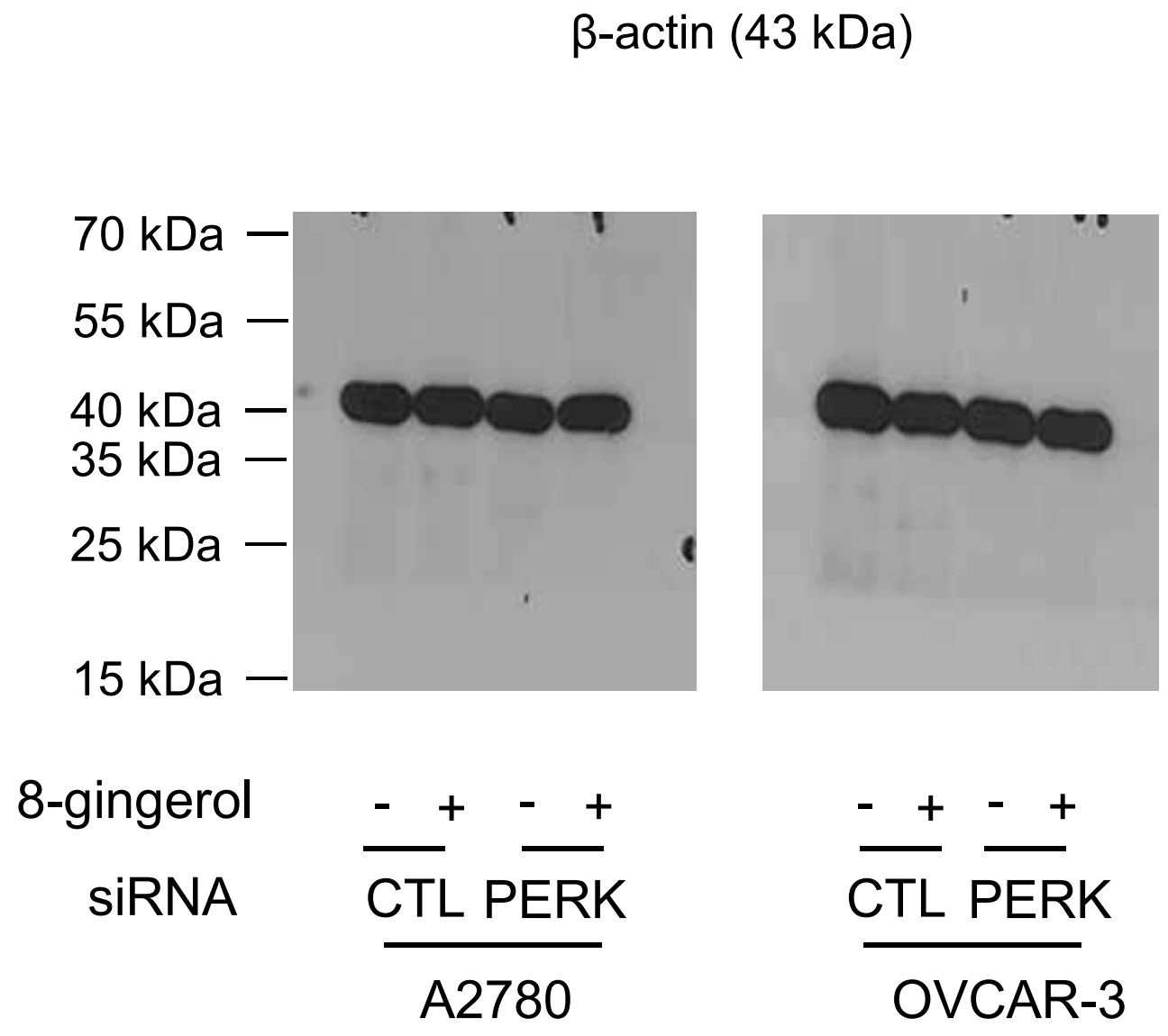

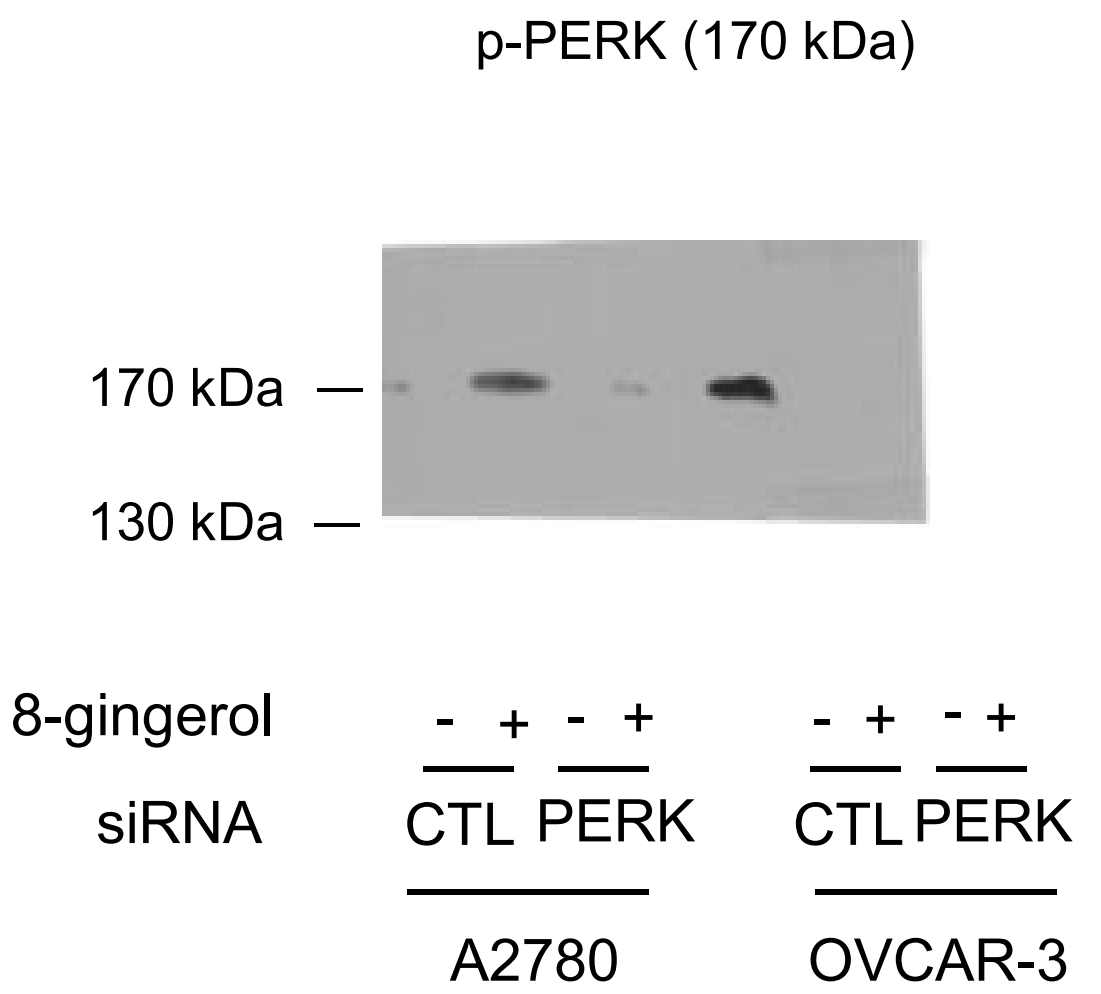

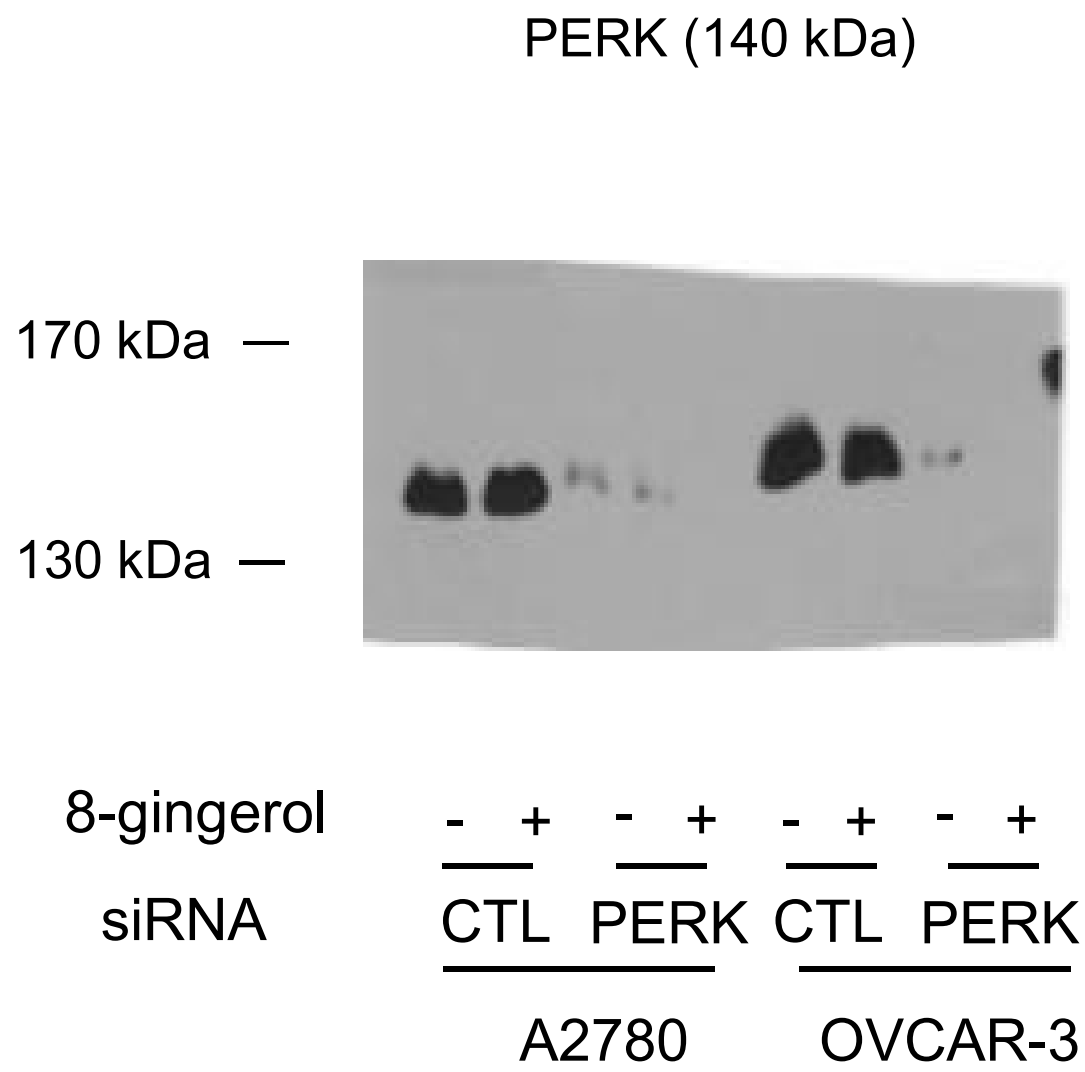

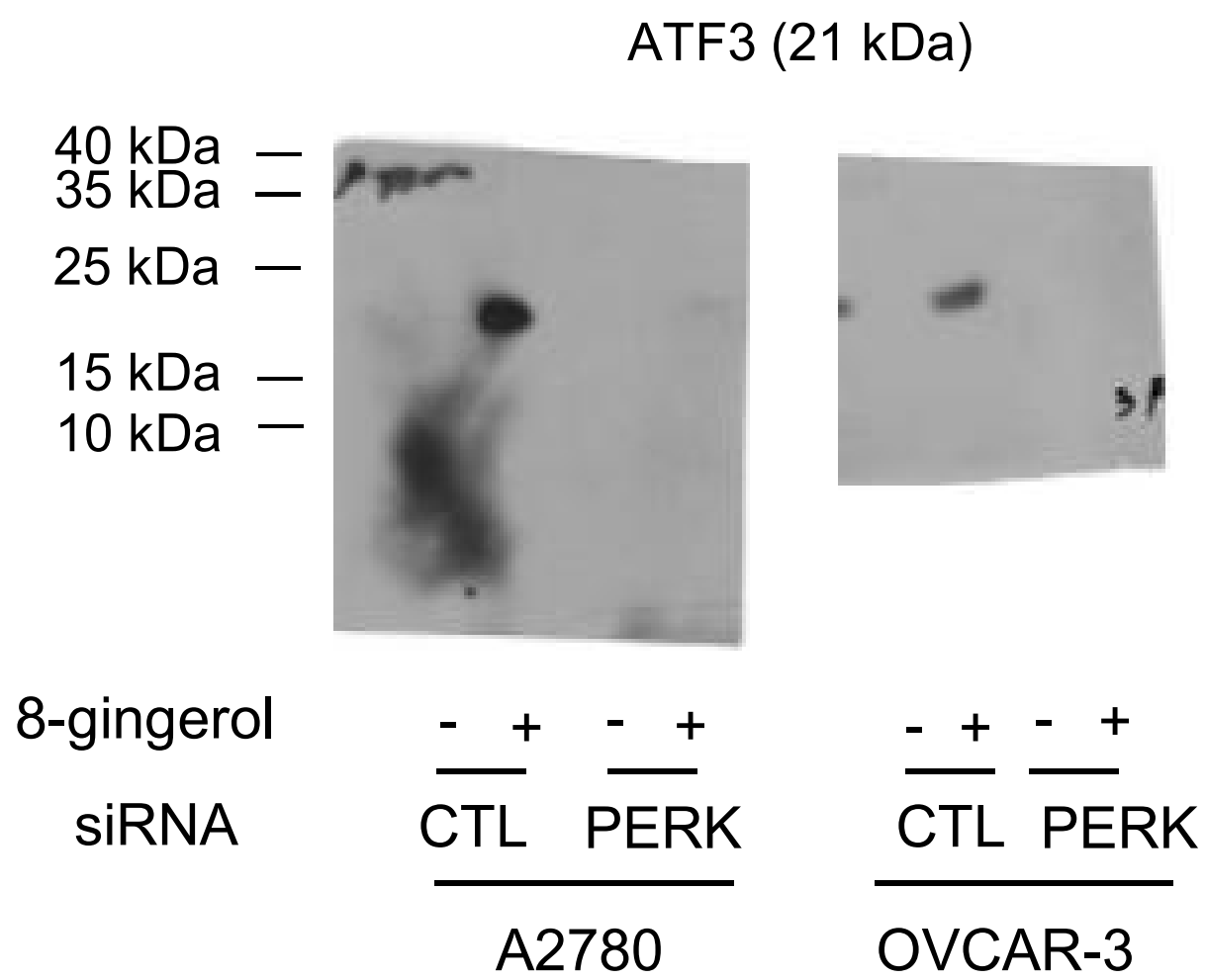

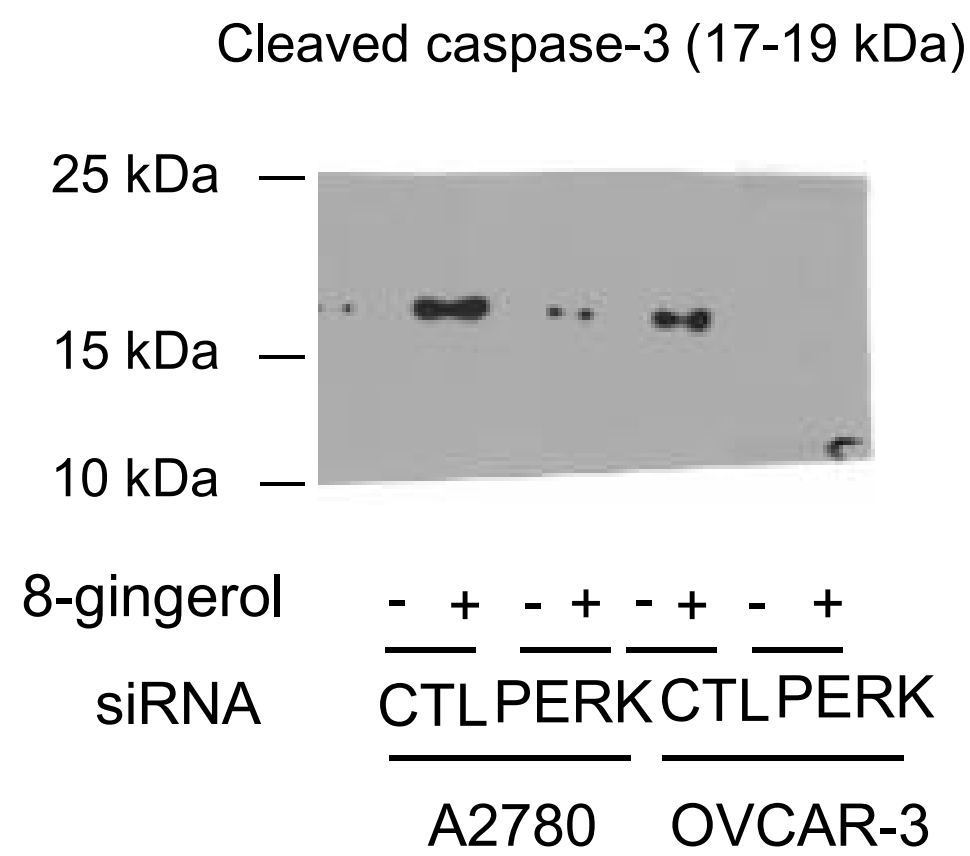

Whole blots for Figure 4I

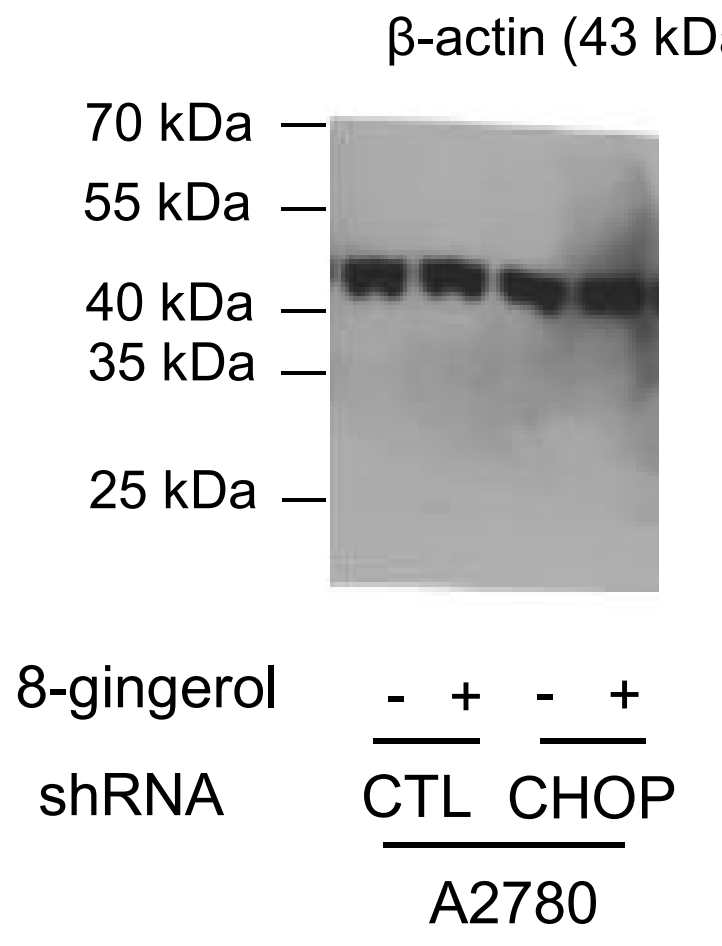

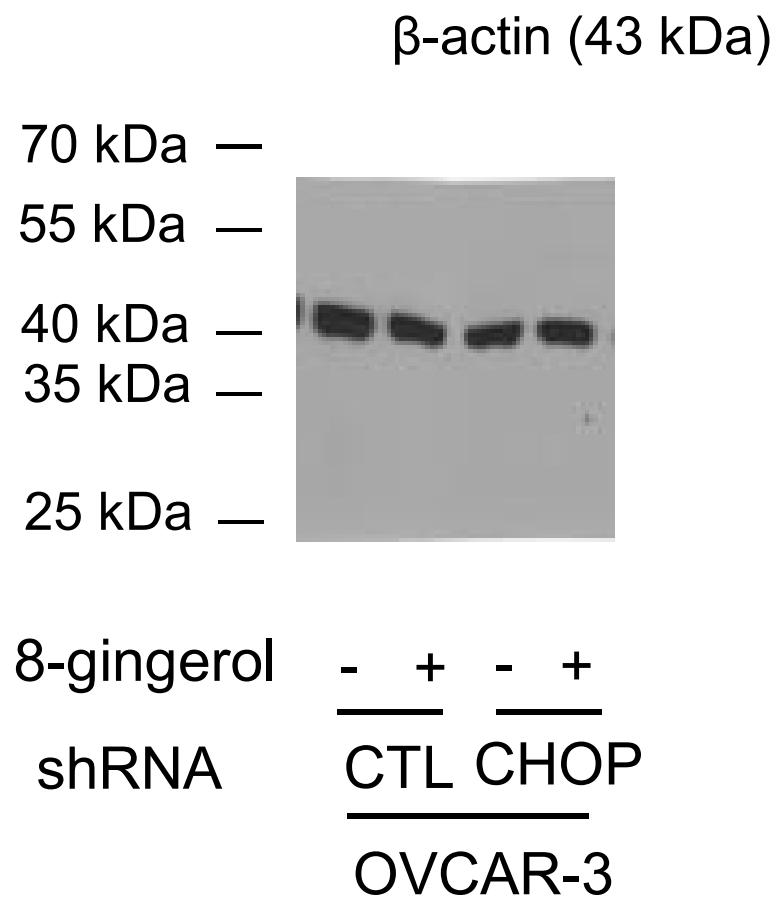

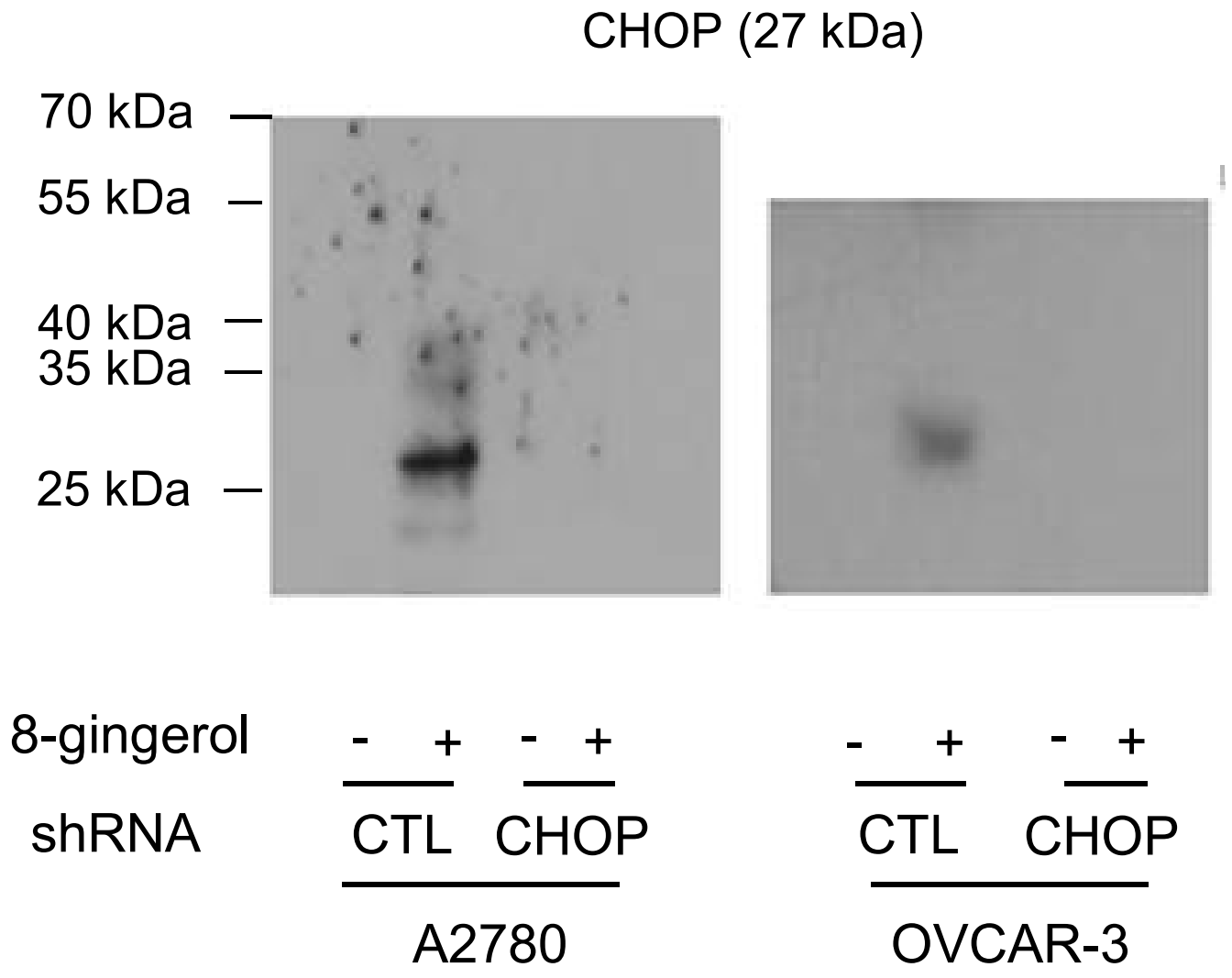

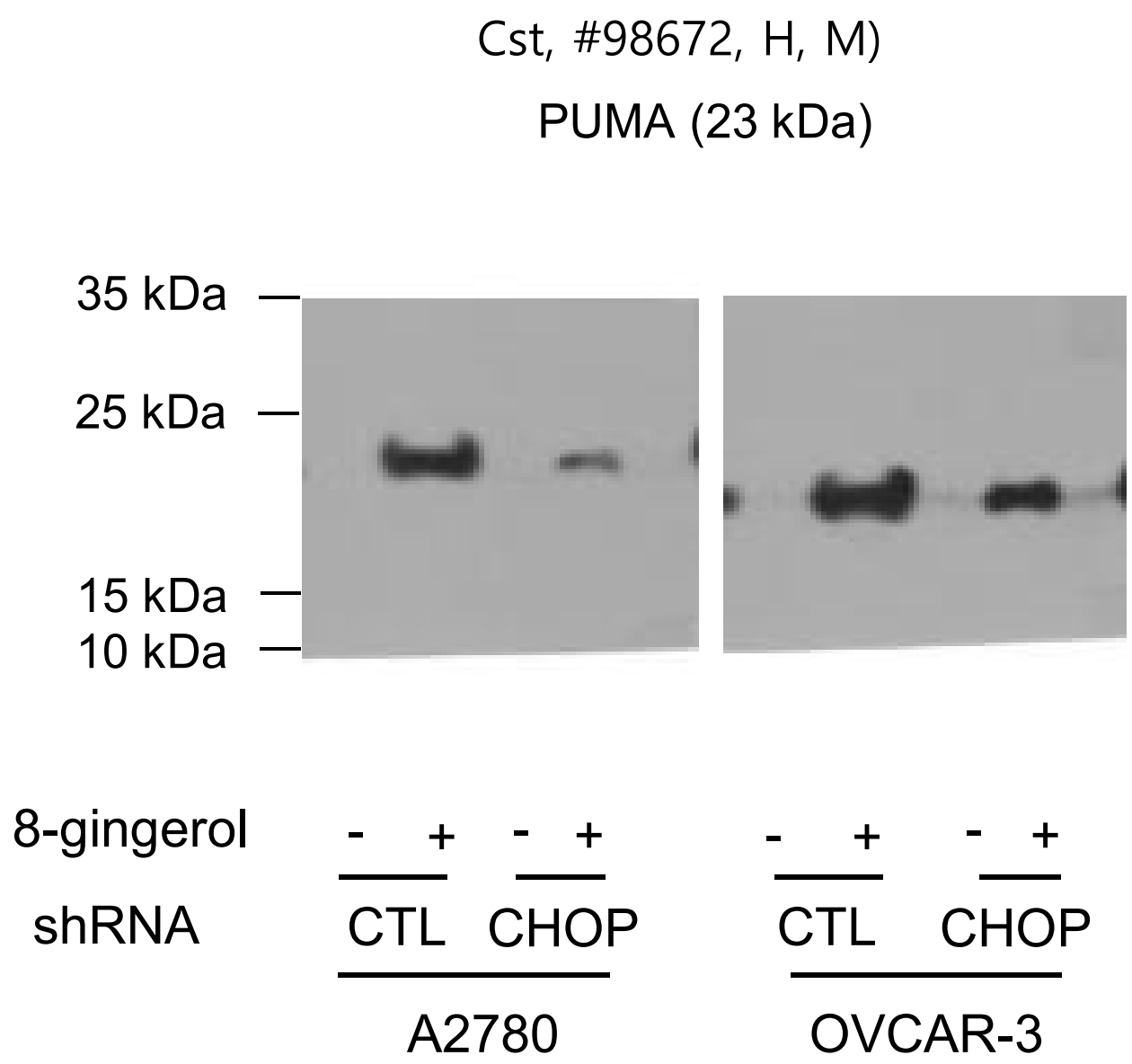

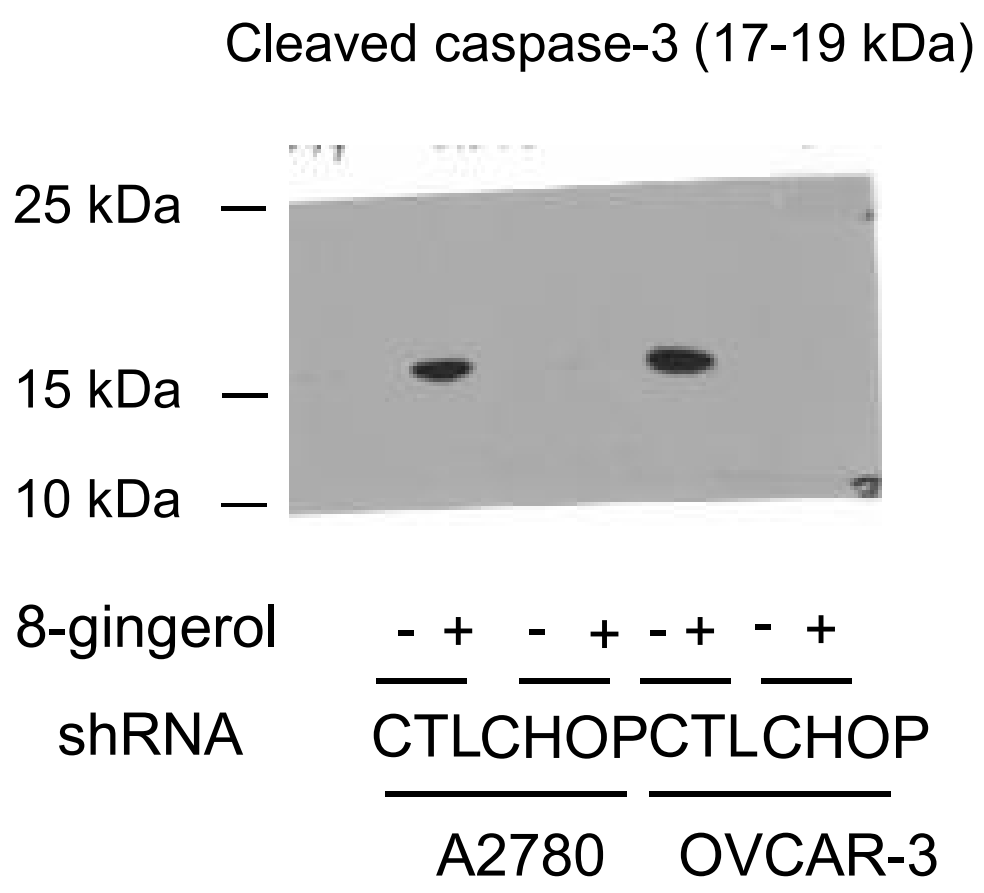

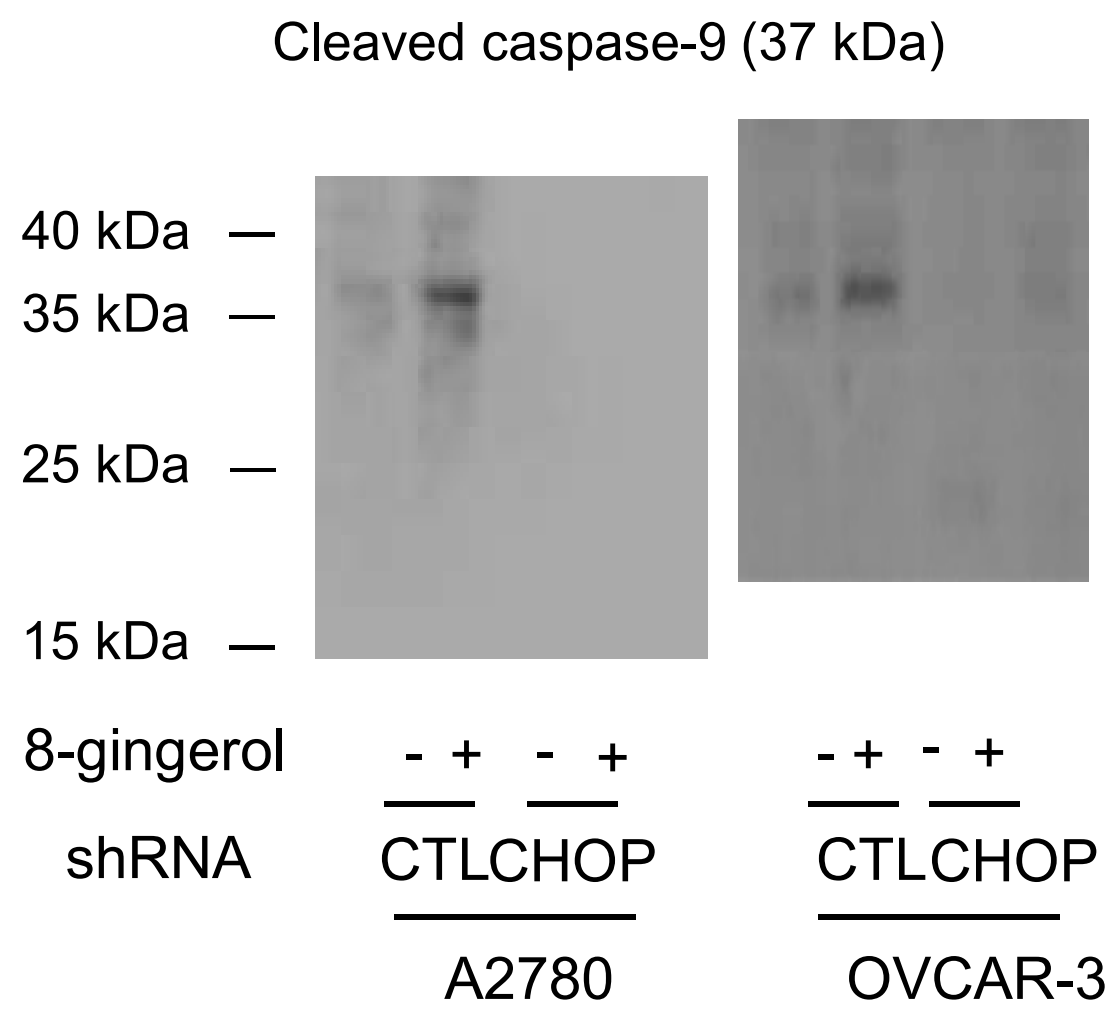

Whole blots for Figure 4L

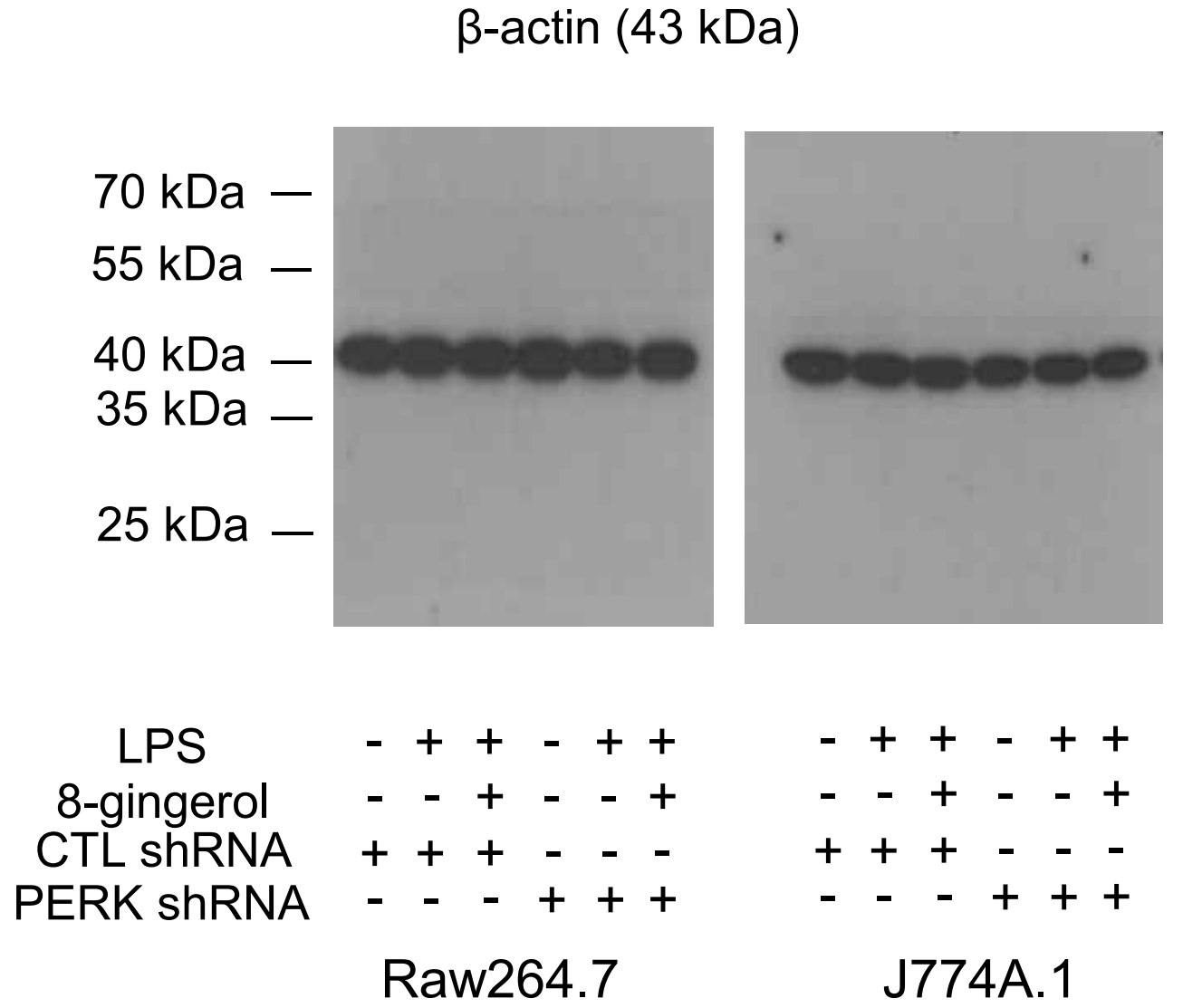

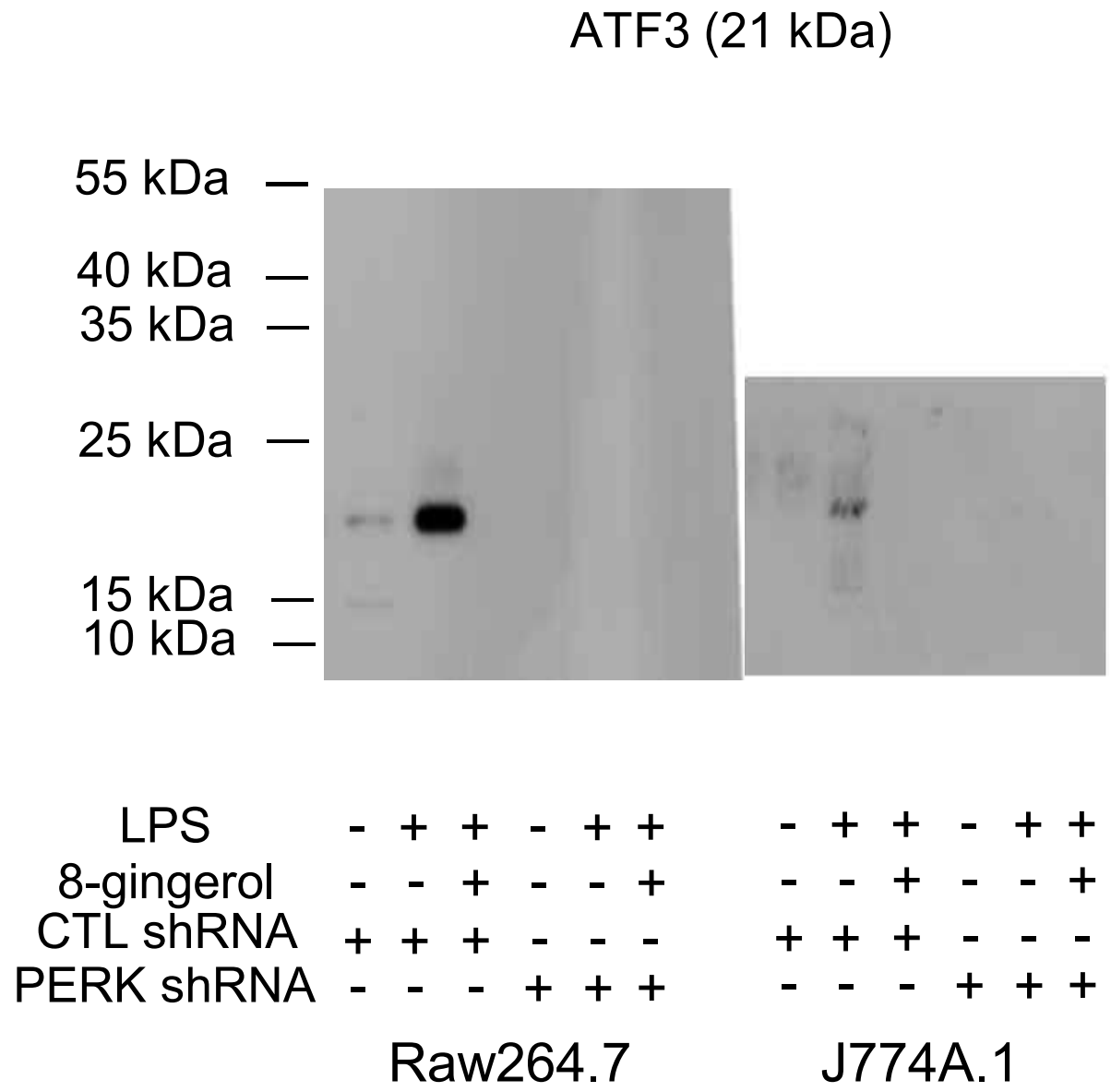

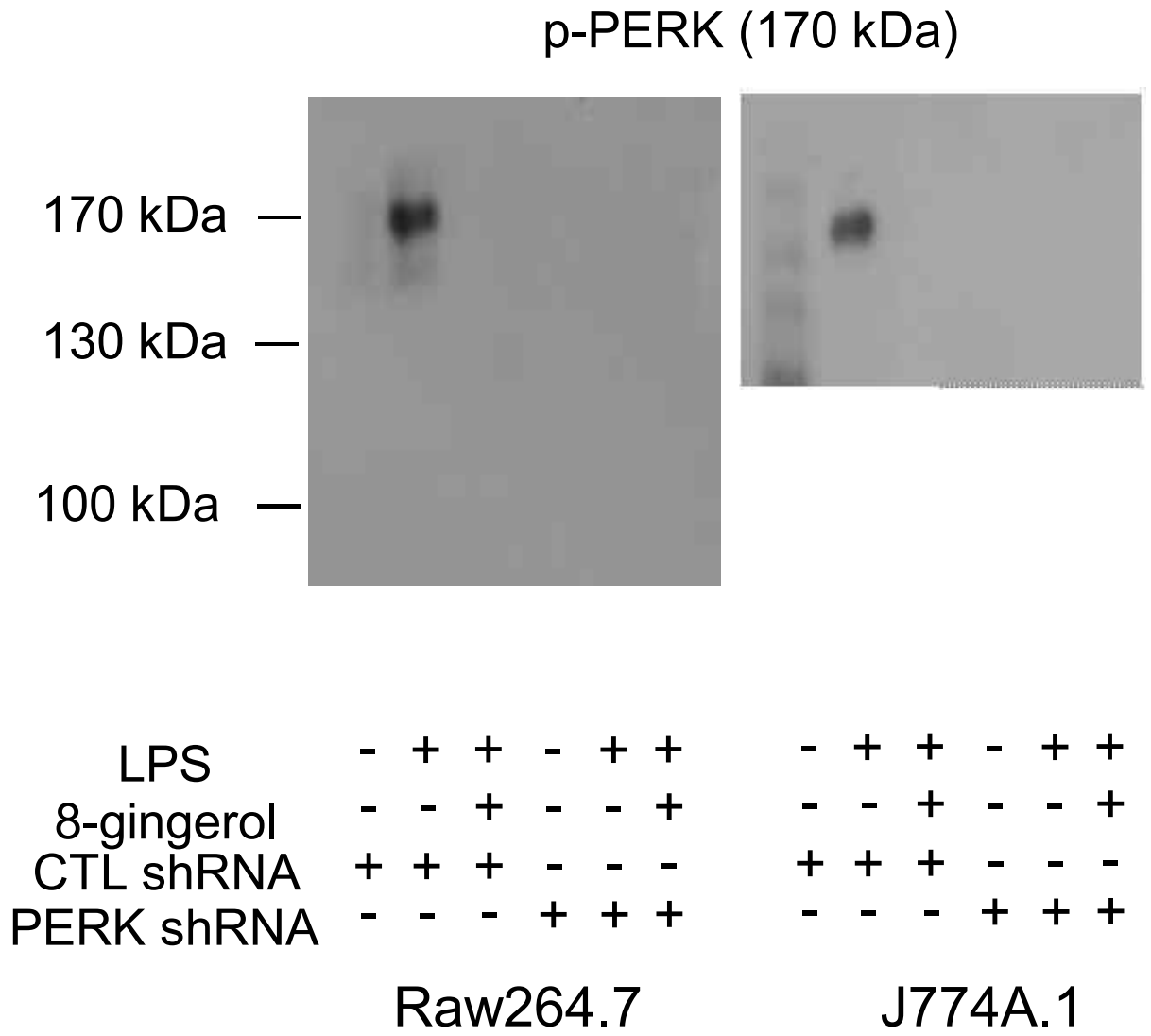

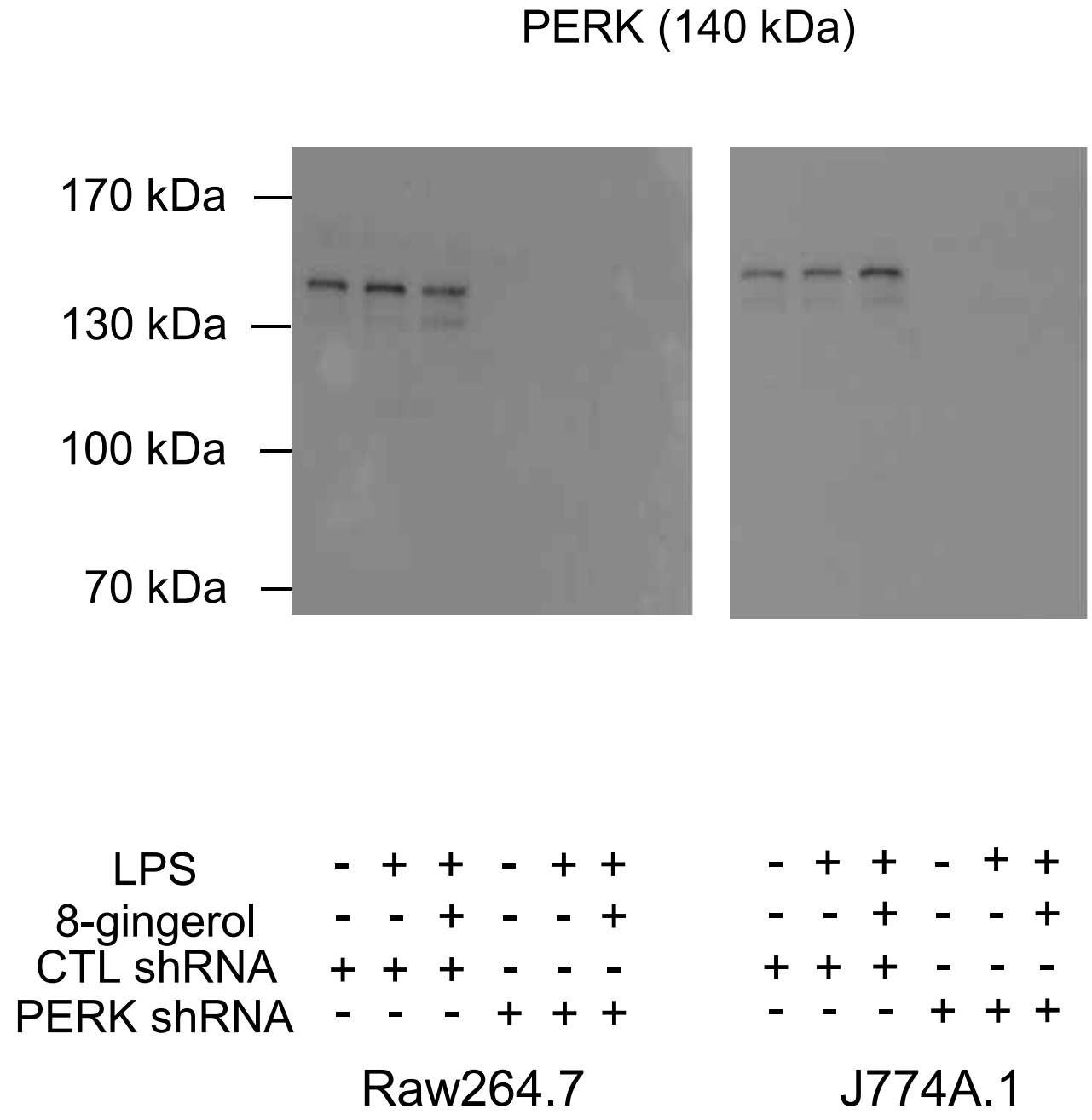

Whole blots for Figure 4L

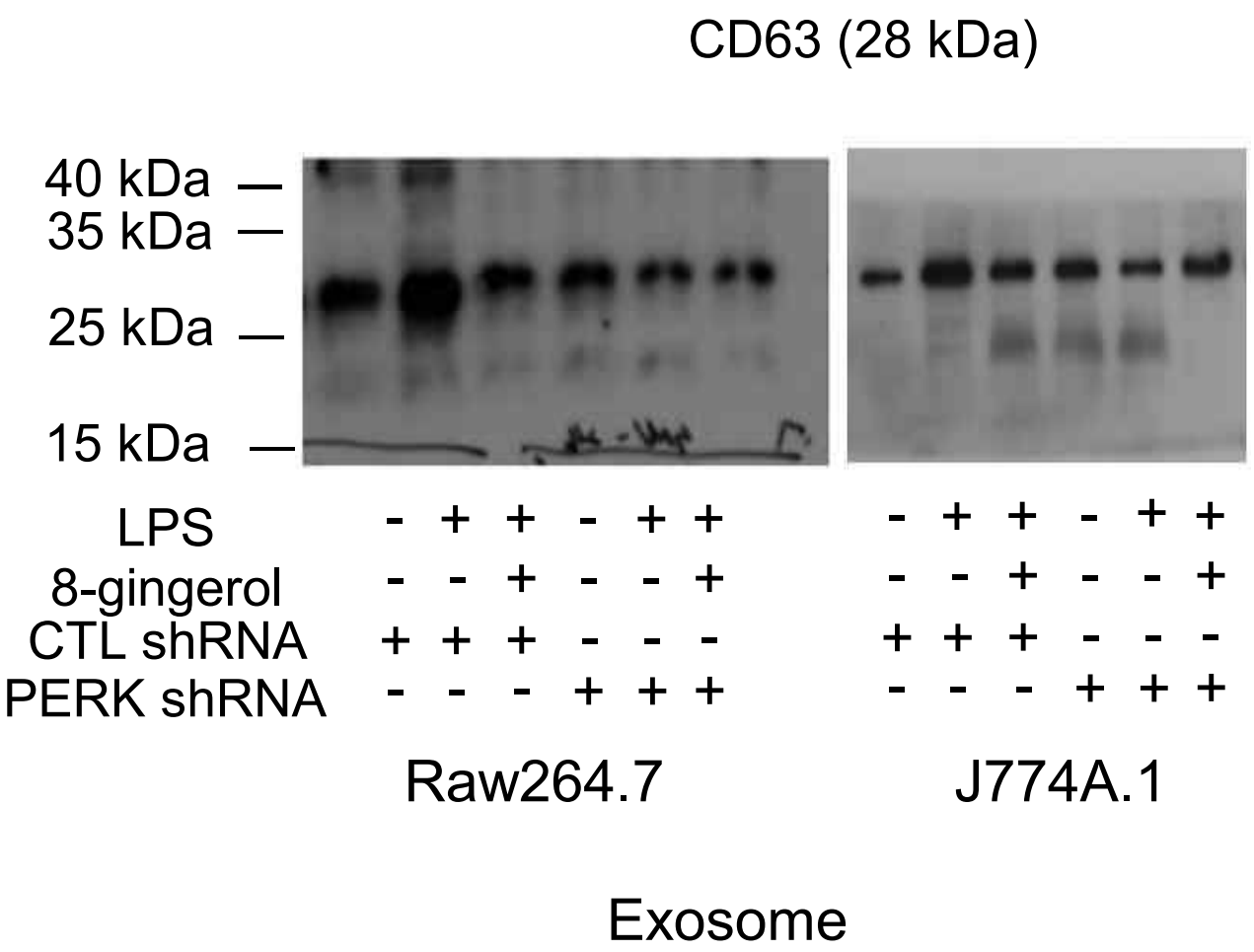

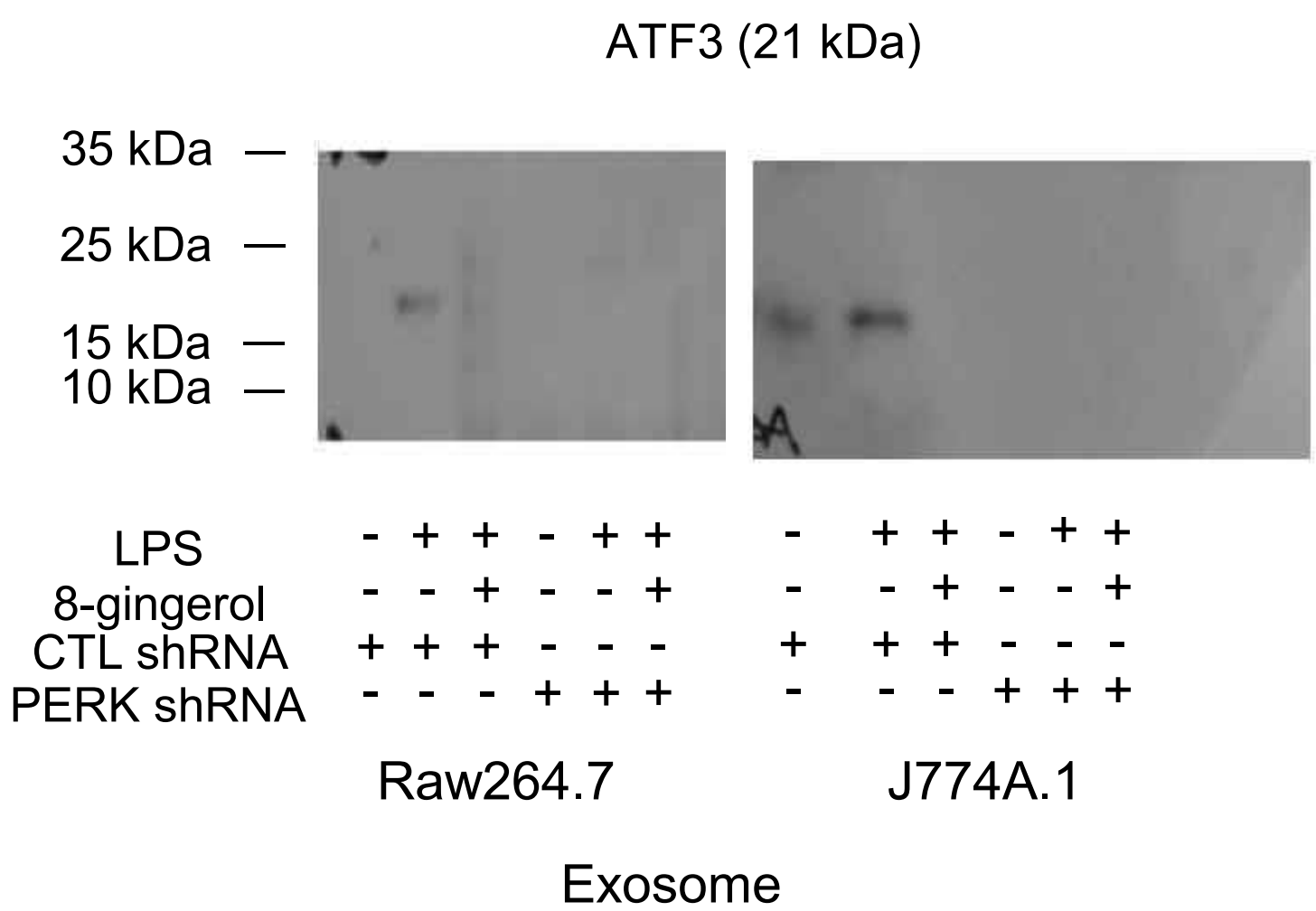

Whole blots for Figure 5I

β-actin (43 kDa)

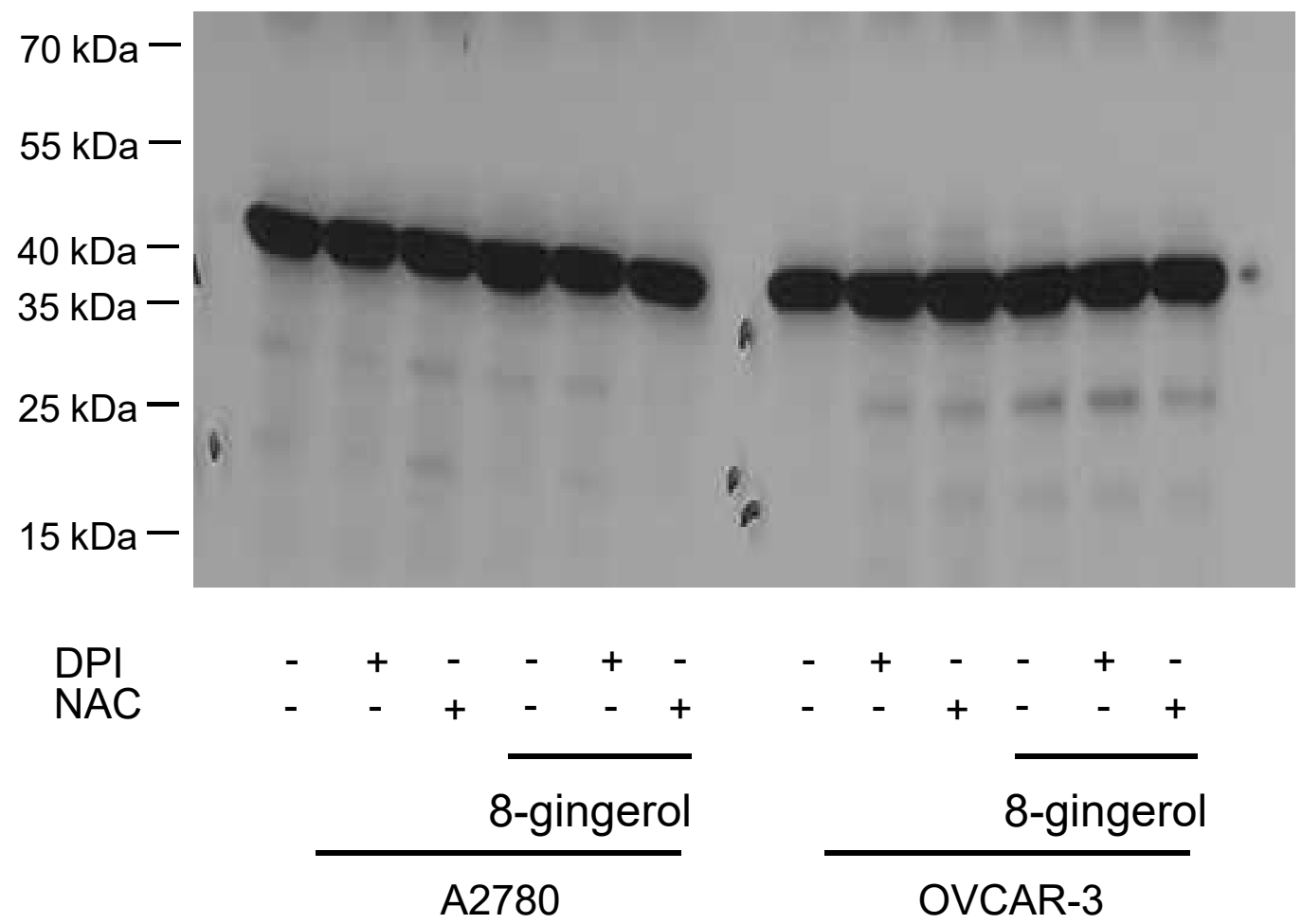

Gpx4 (17 kDa)

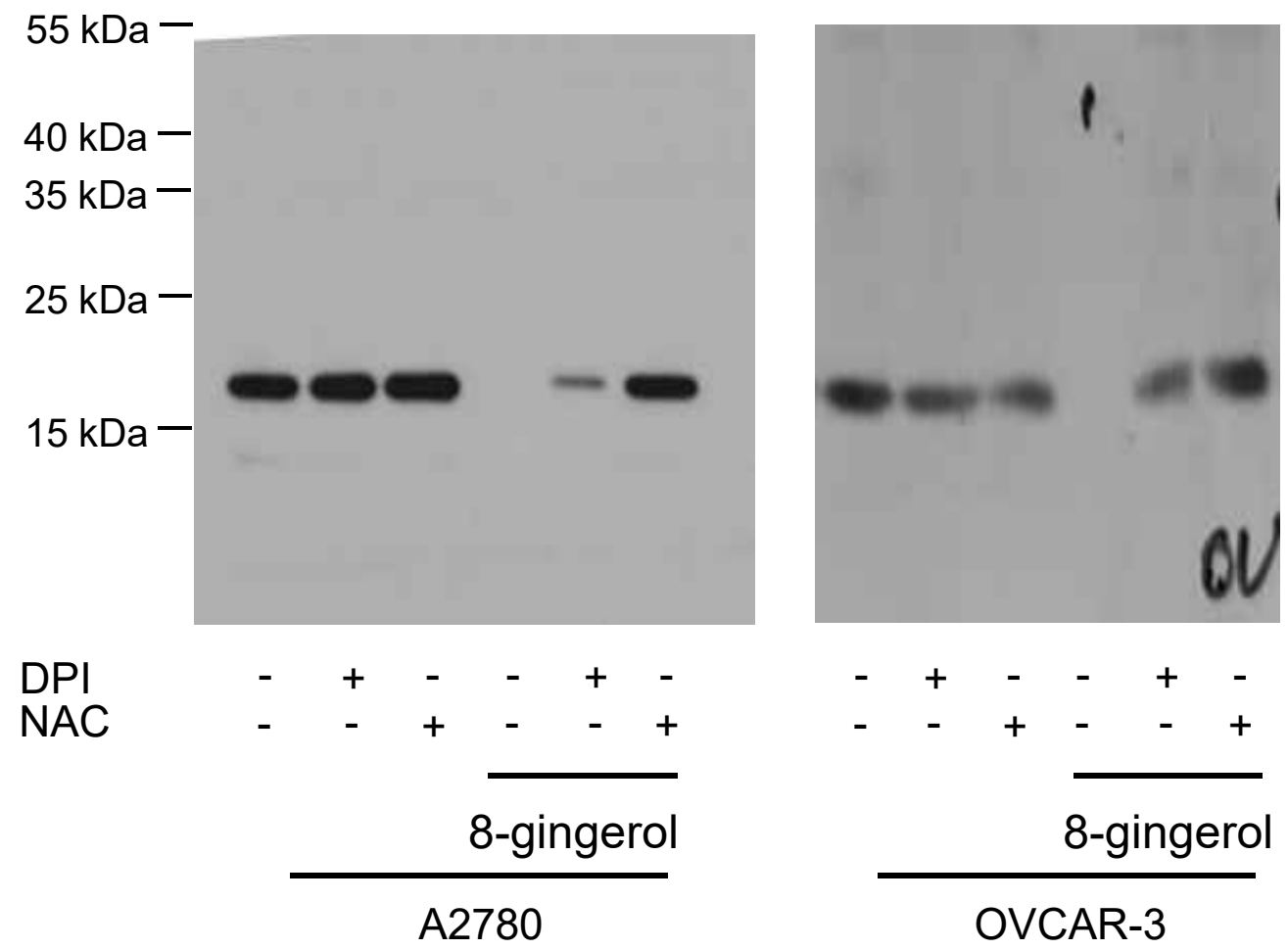

p-PERK (170 kDa)

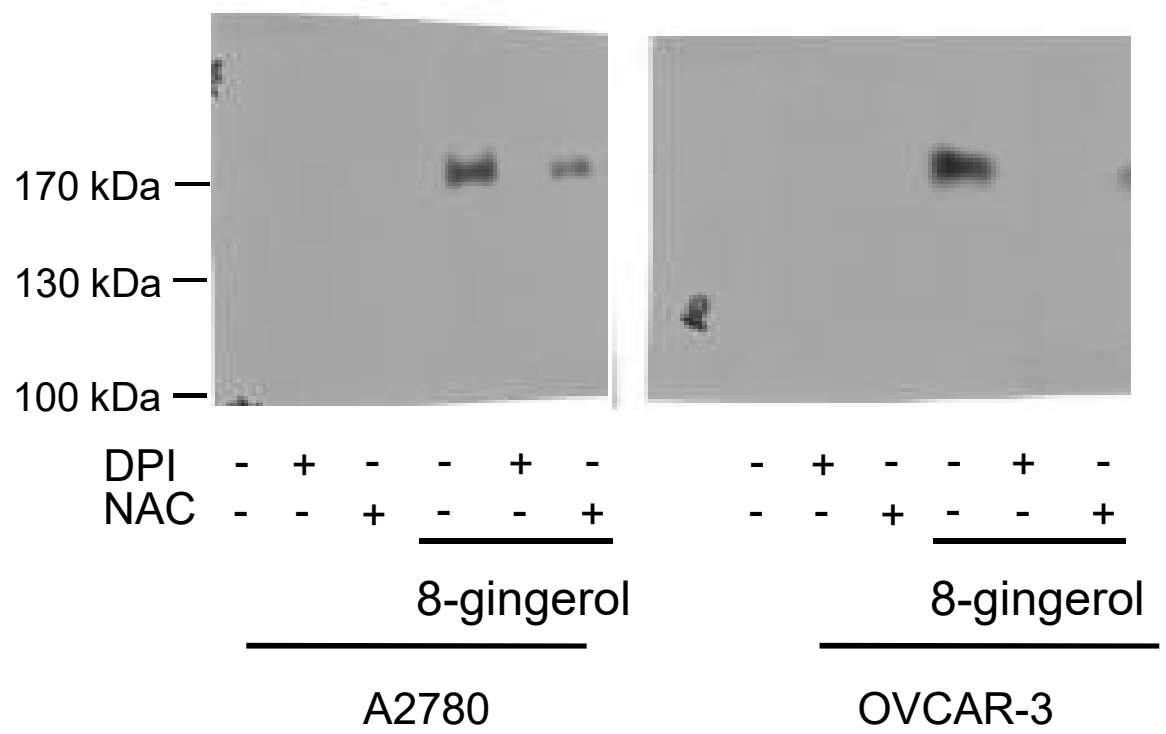

Nox4 (62 kDa)

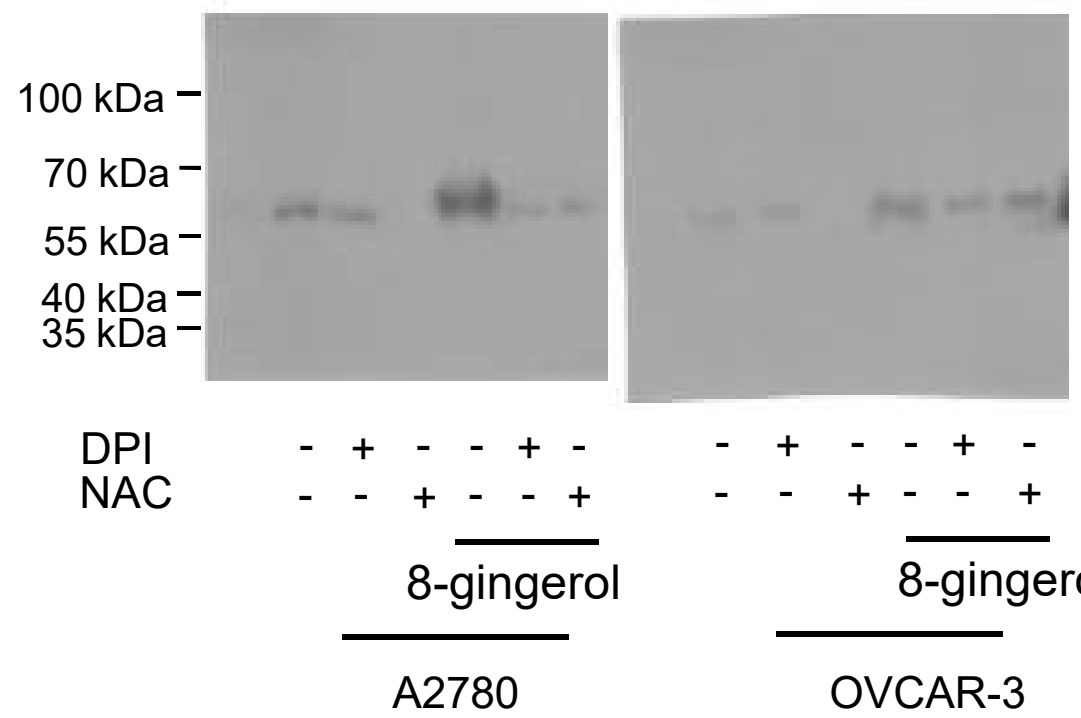

Cleaved caspase-3 (17-19 kDa)

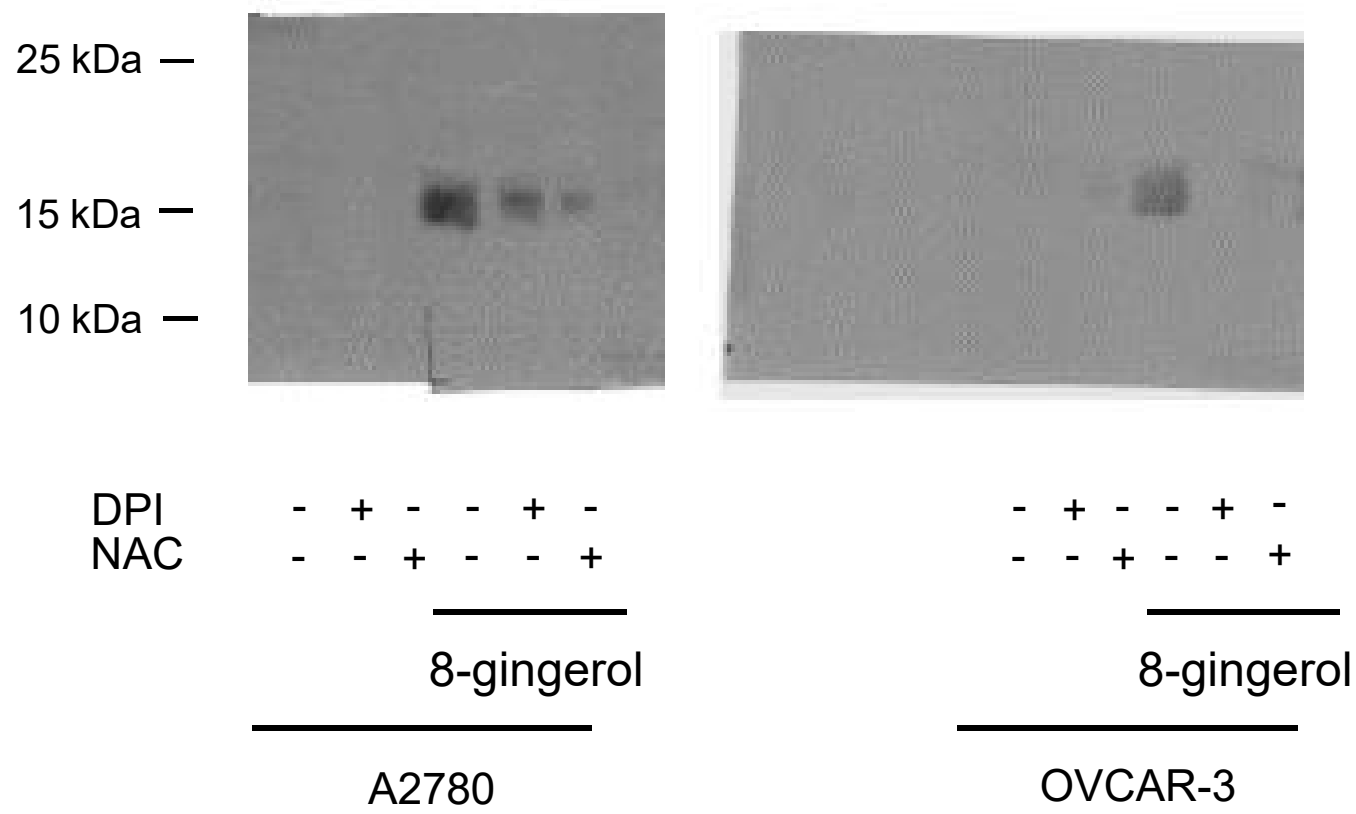

ATF3 (21 kDa)

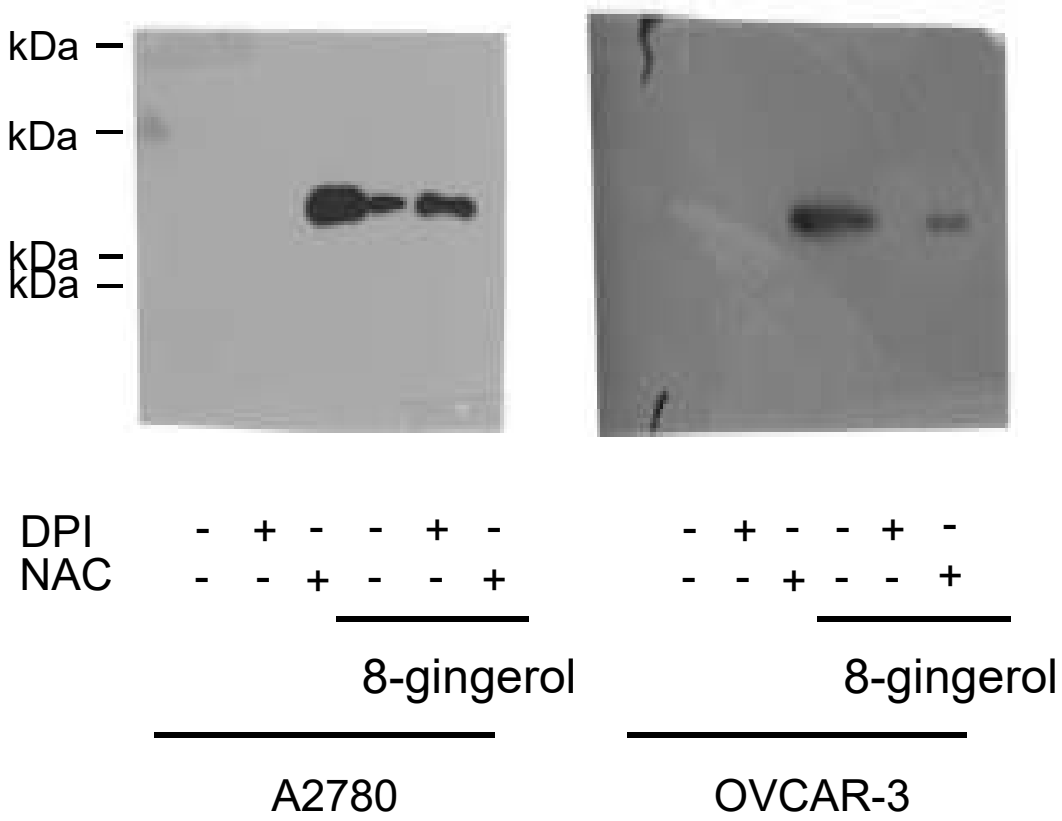

Whole blots for Figure 6A

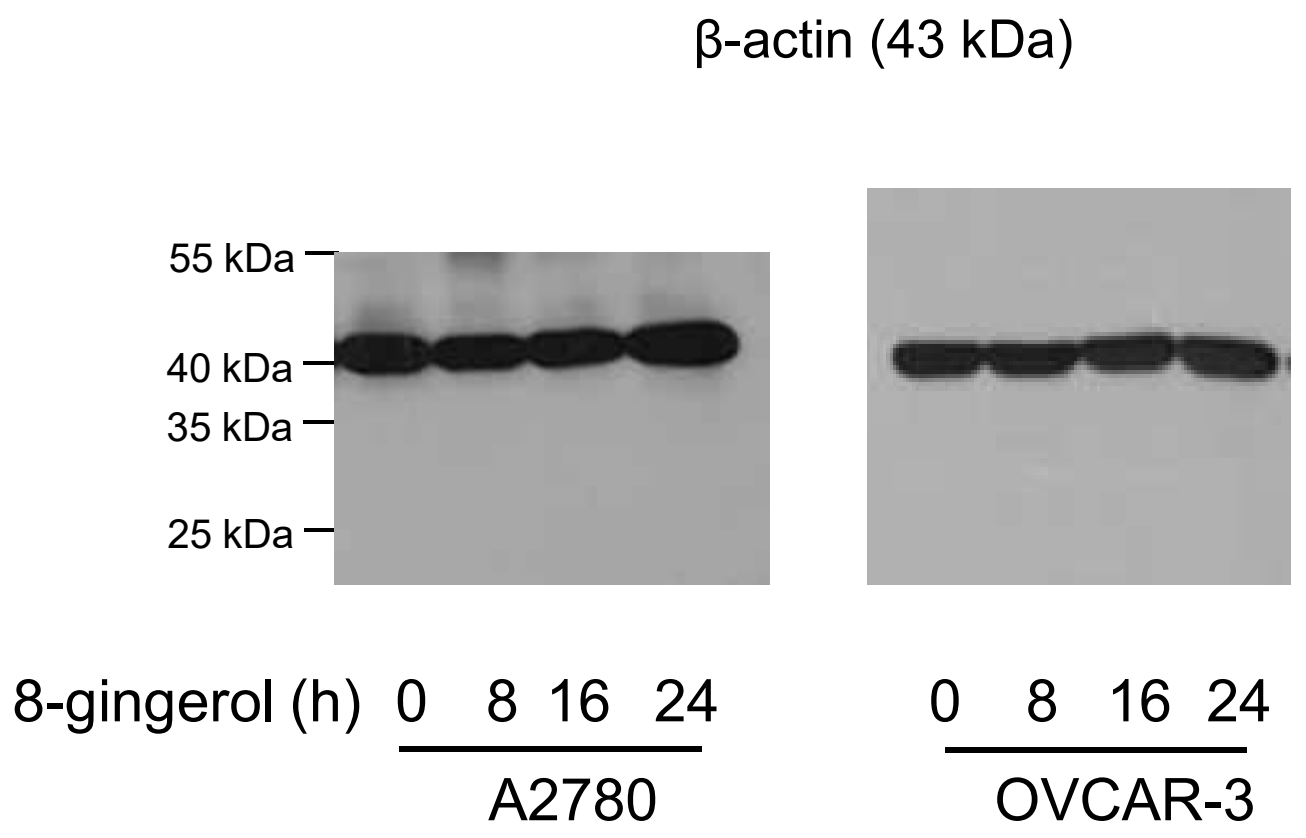

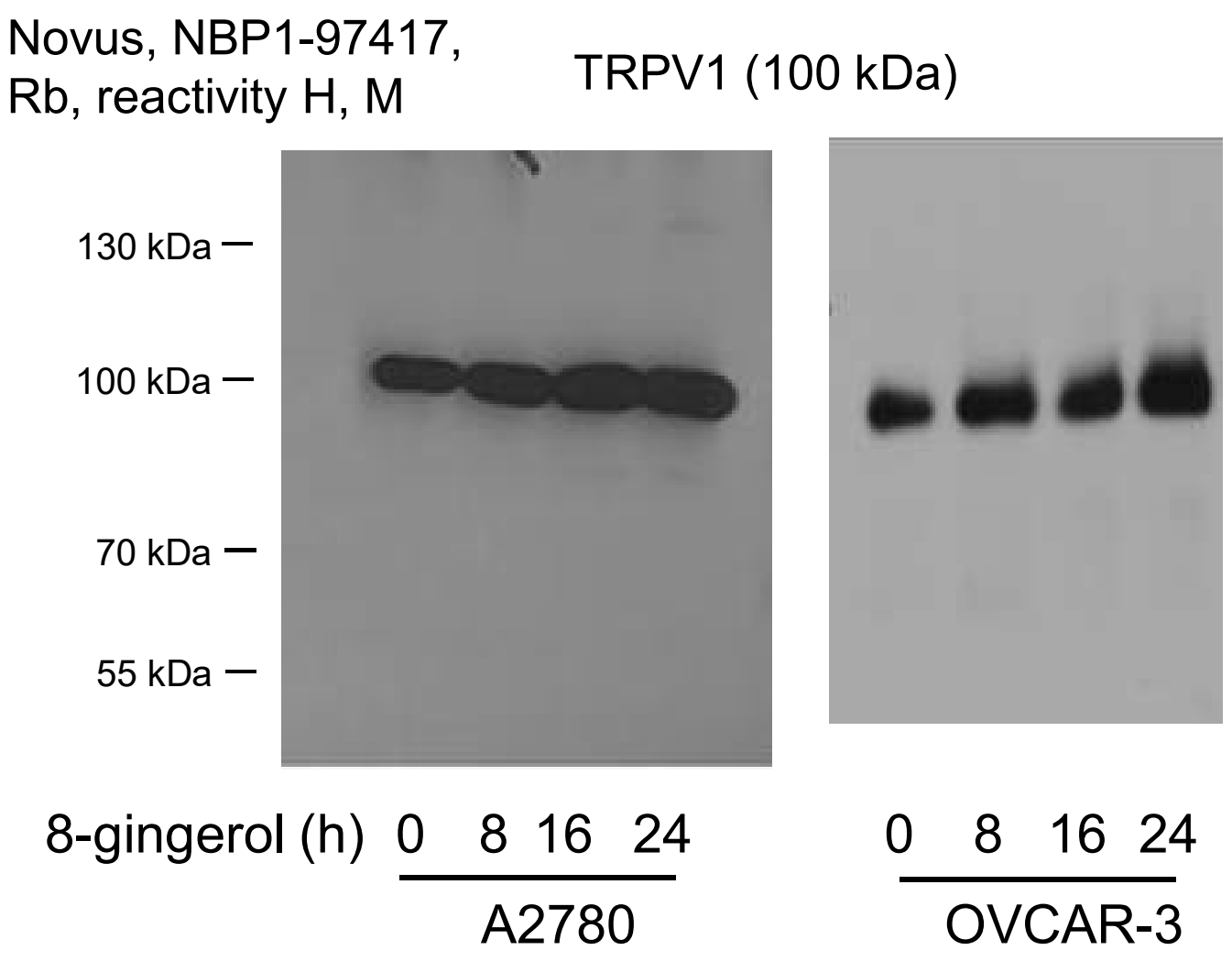

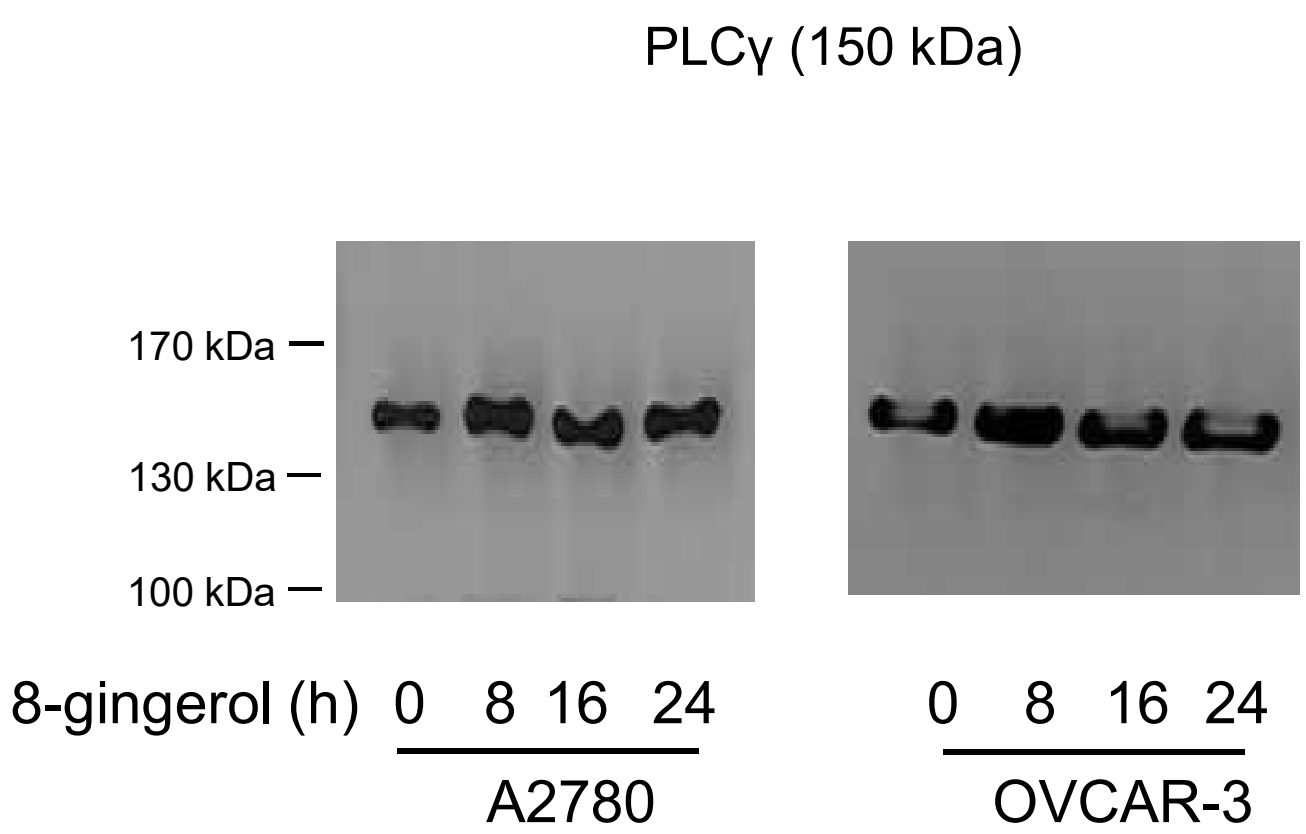

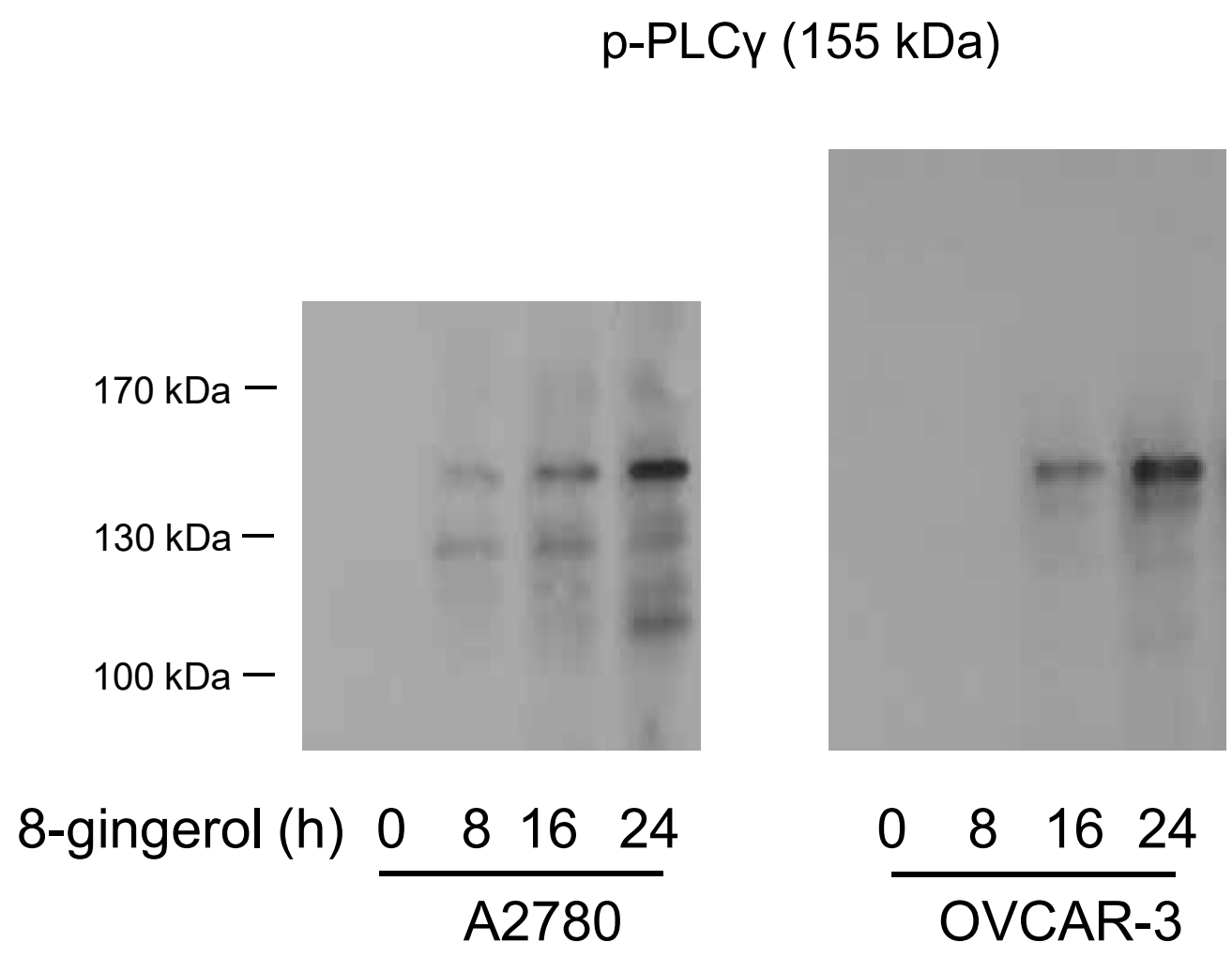

Whole blots for Figure 6A

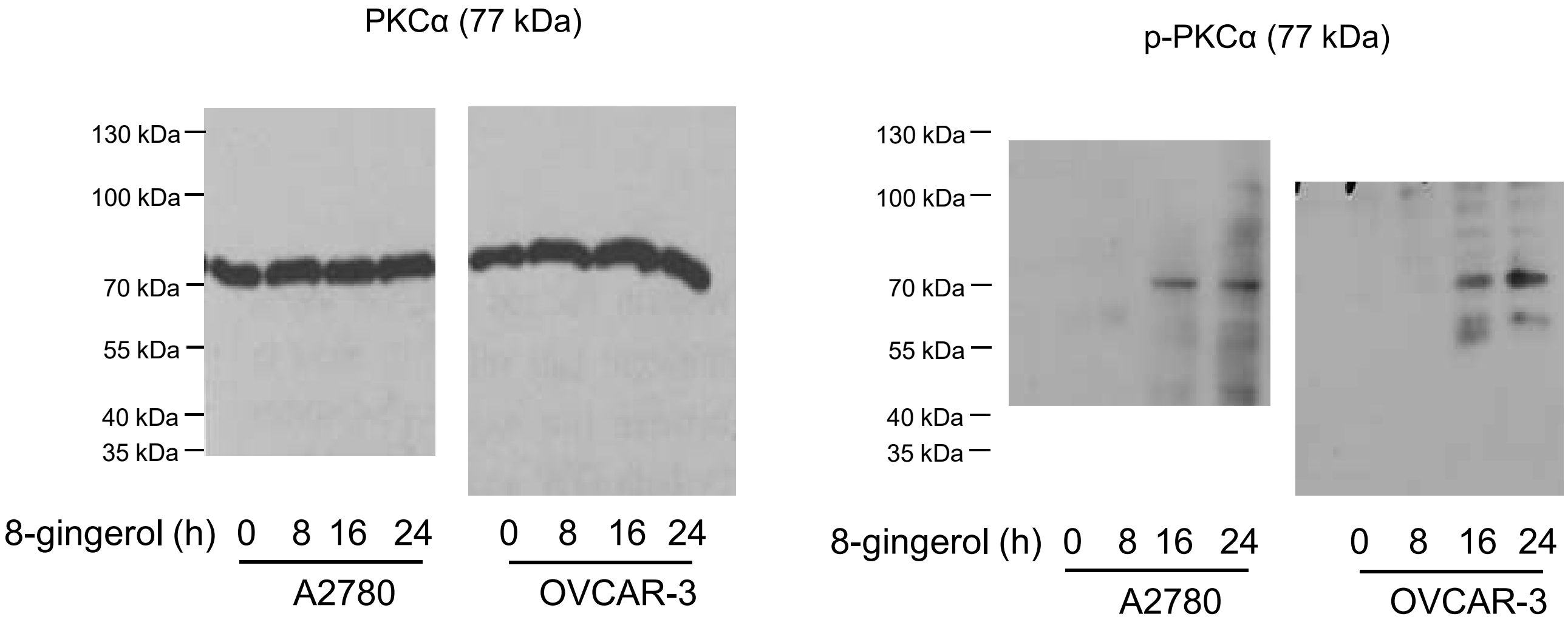

Whole blots for Figure 6F

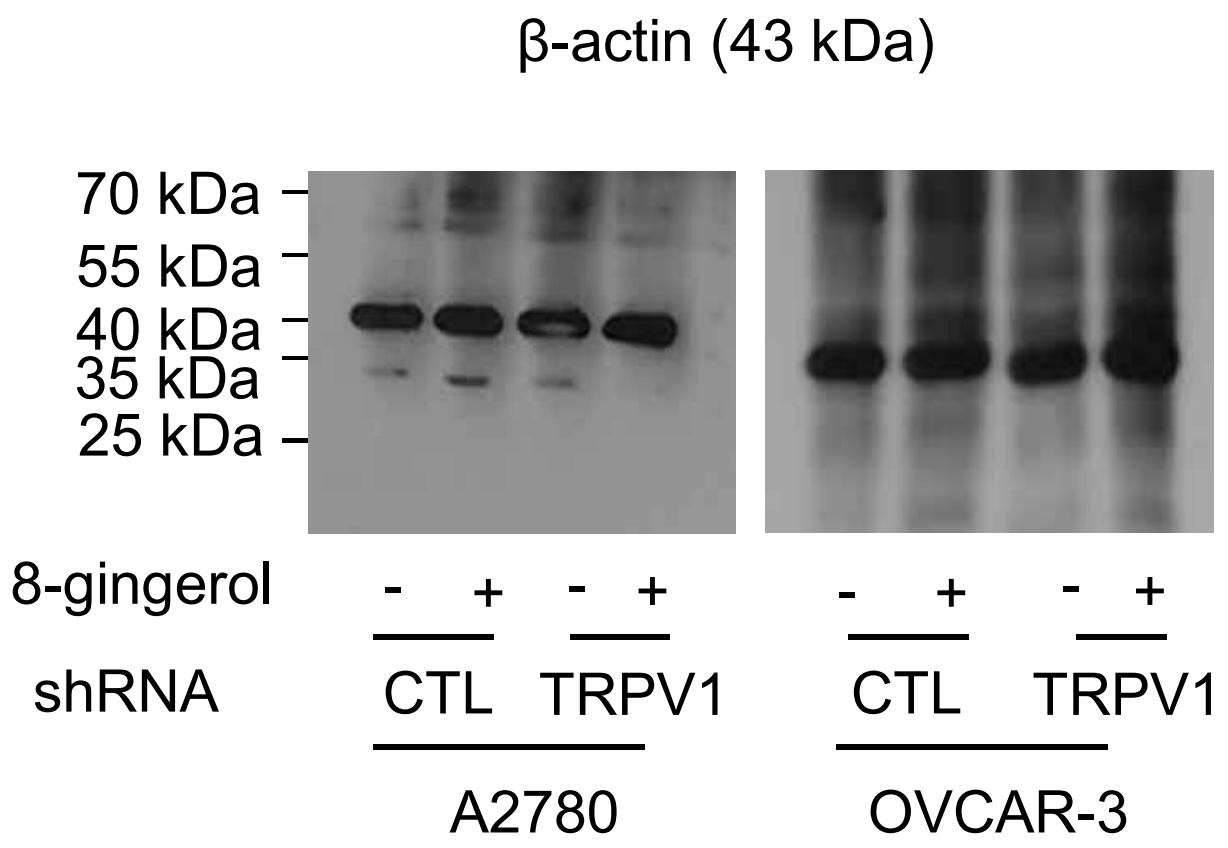

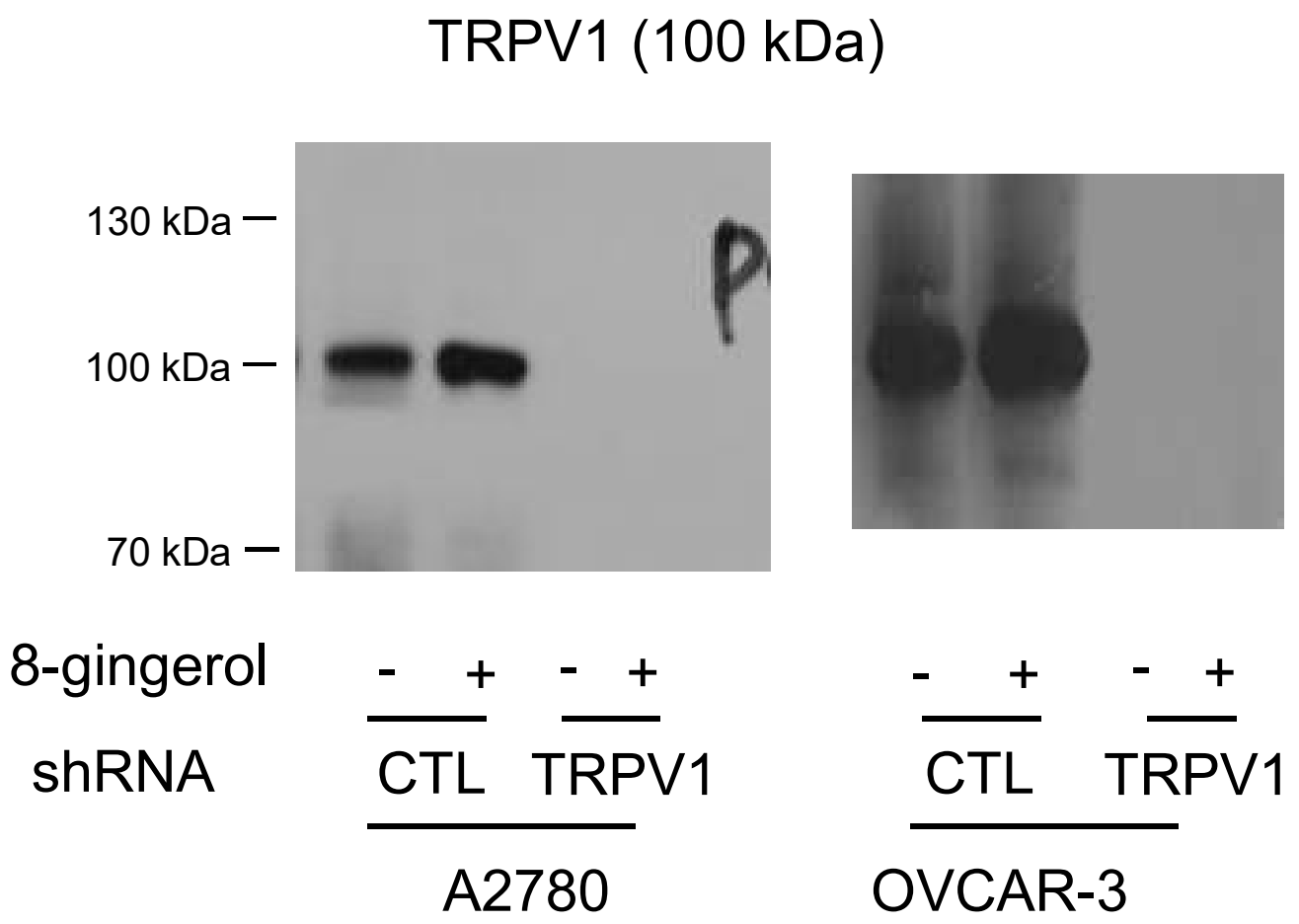

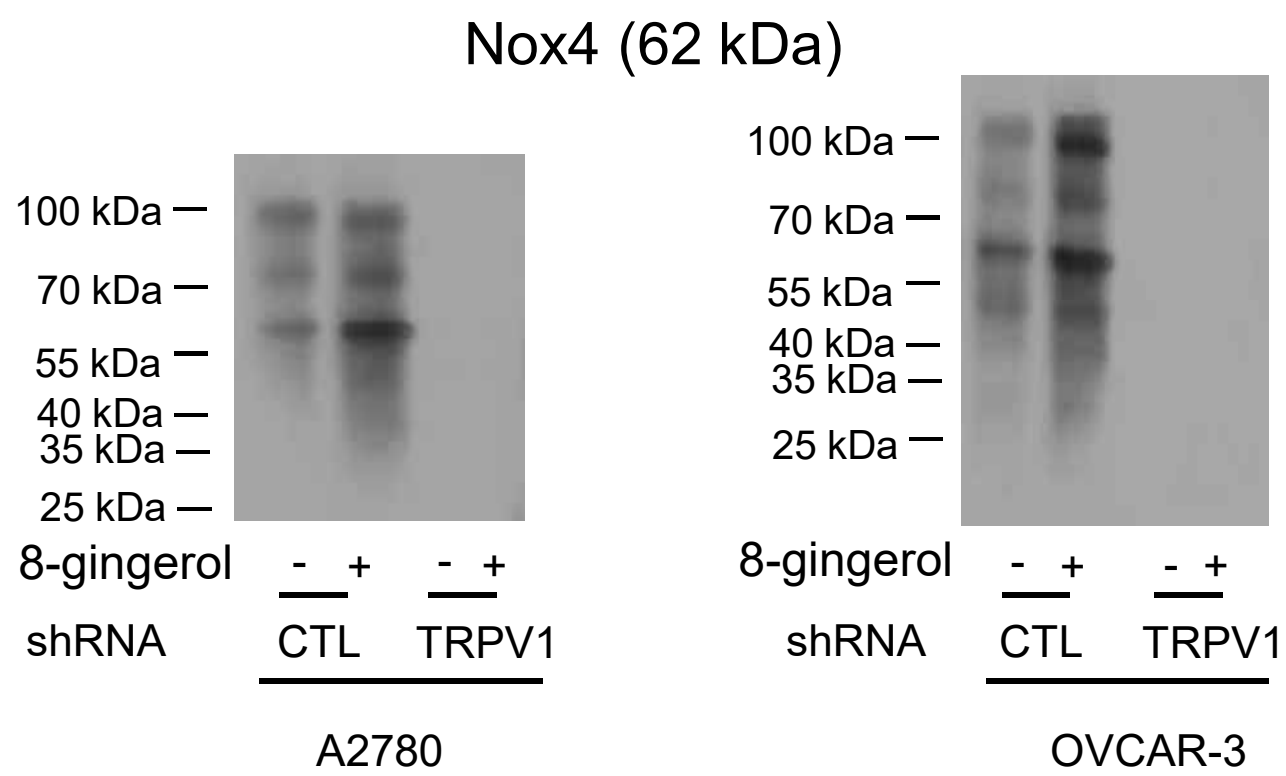

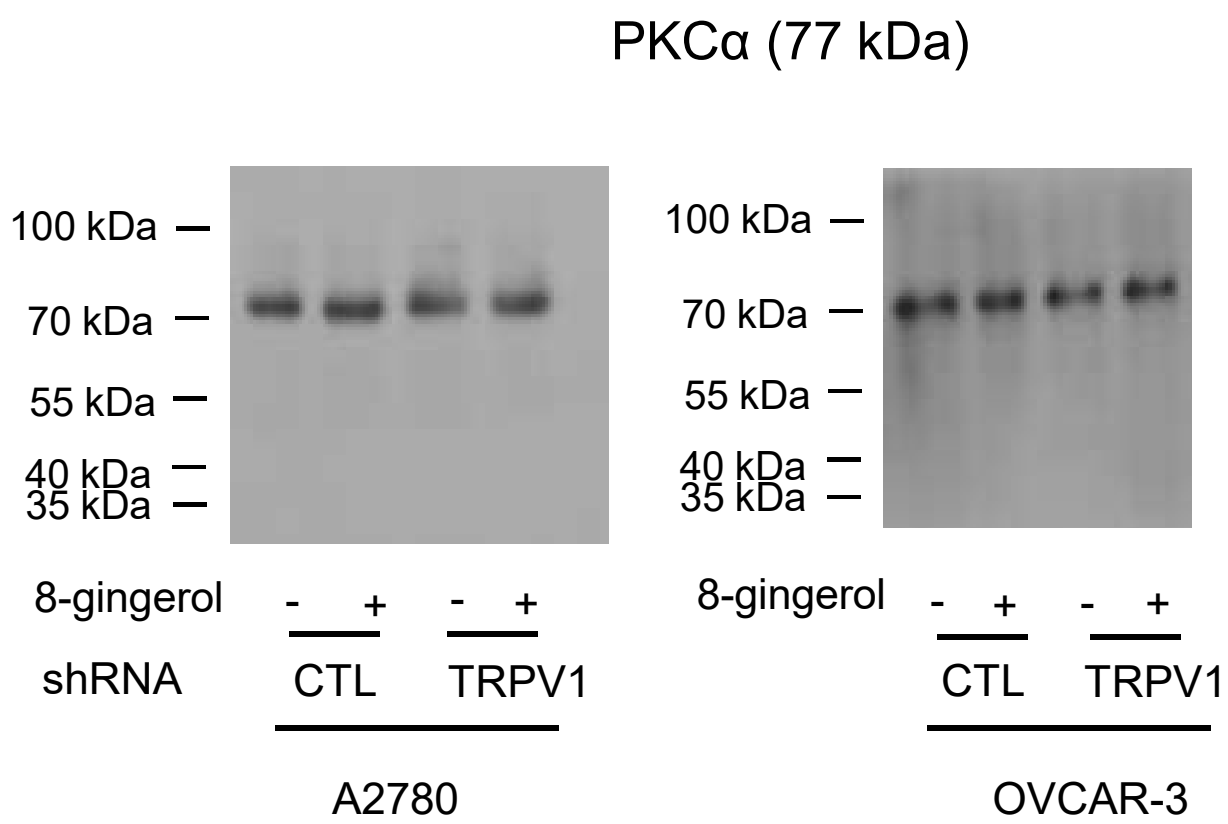

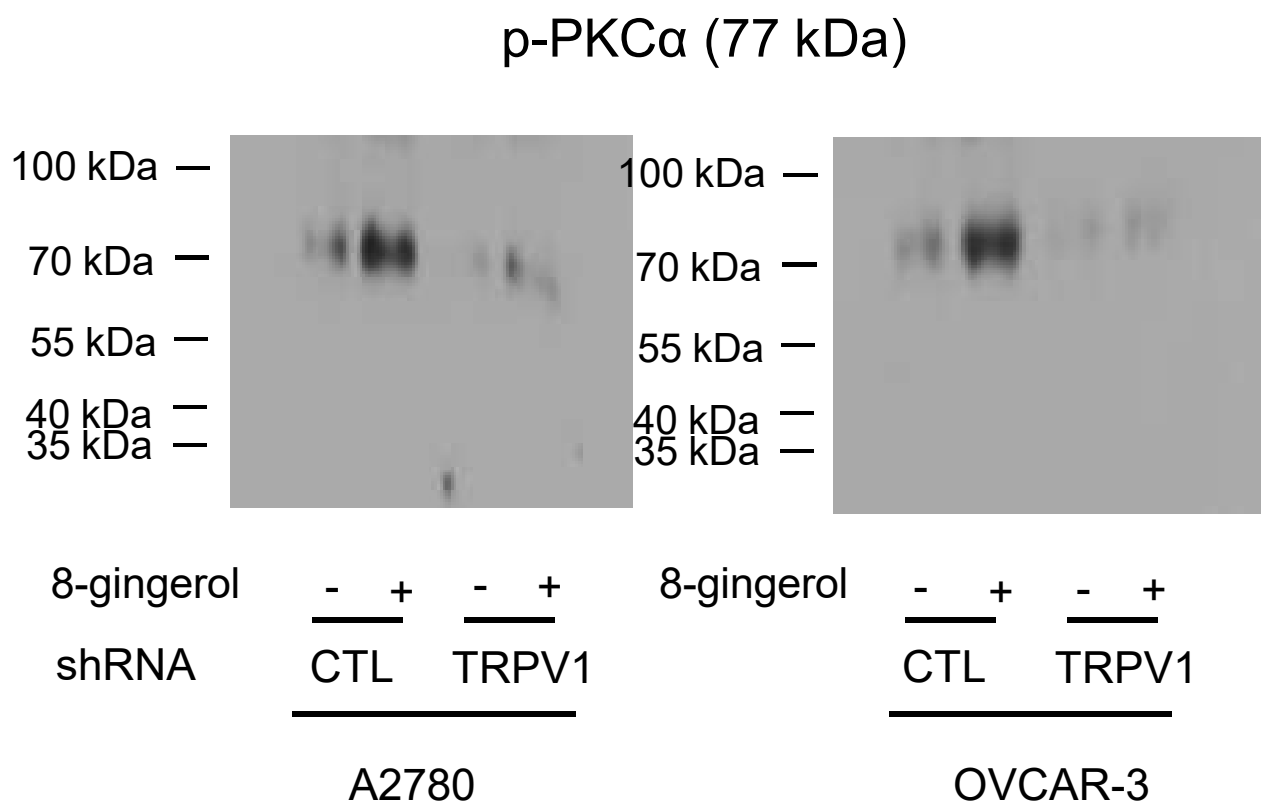

Whole blots for Figure 6L

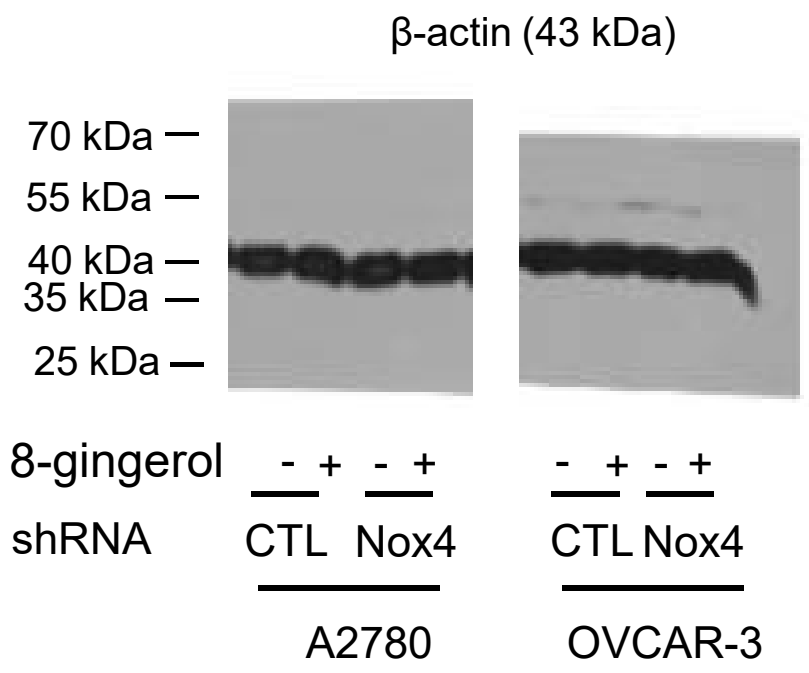

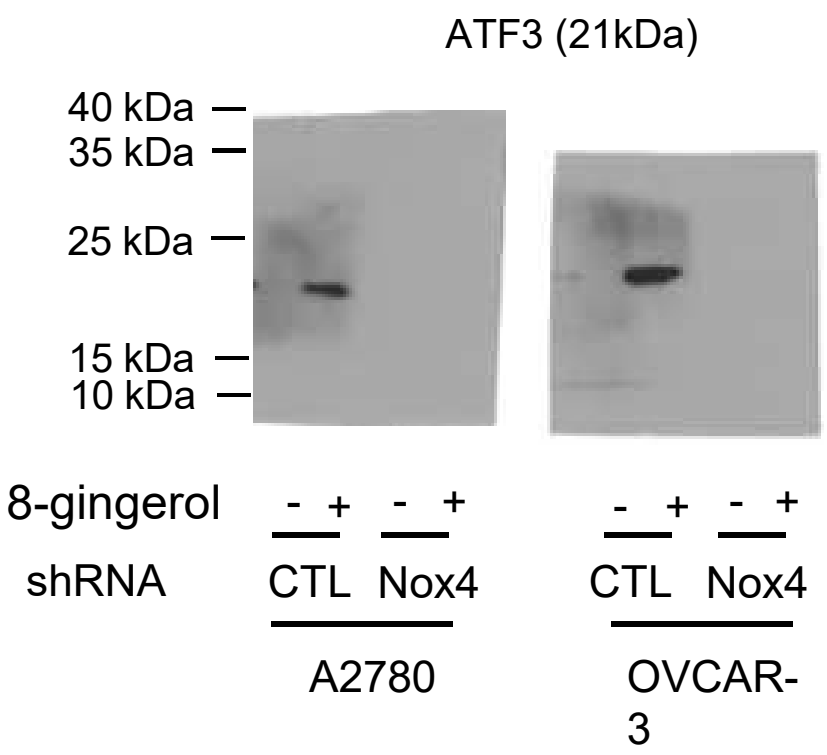

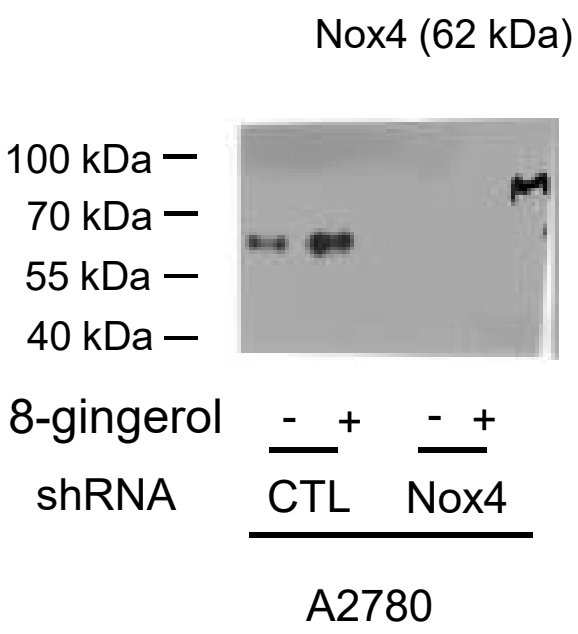

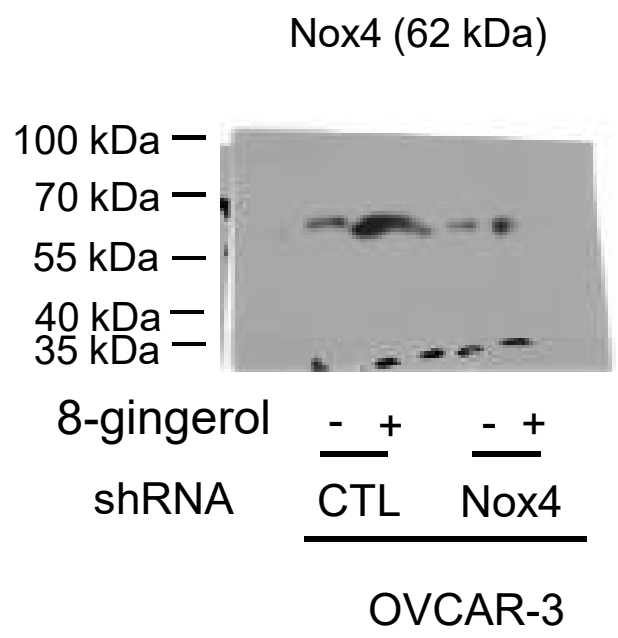

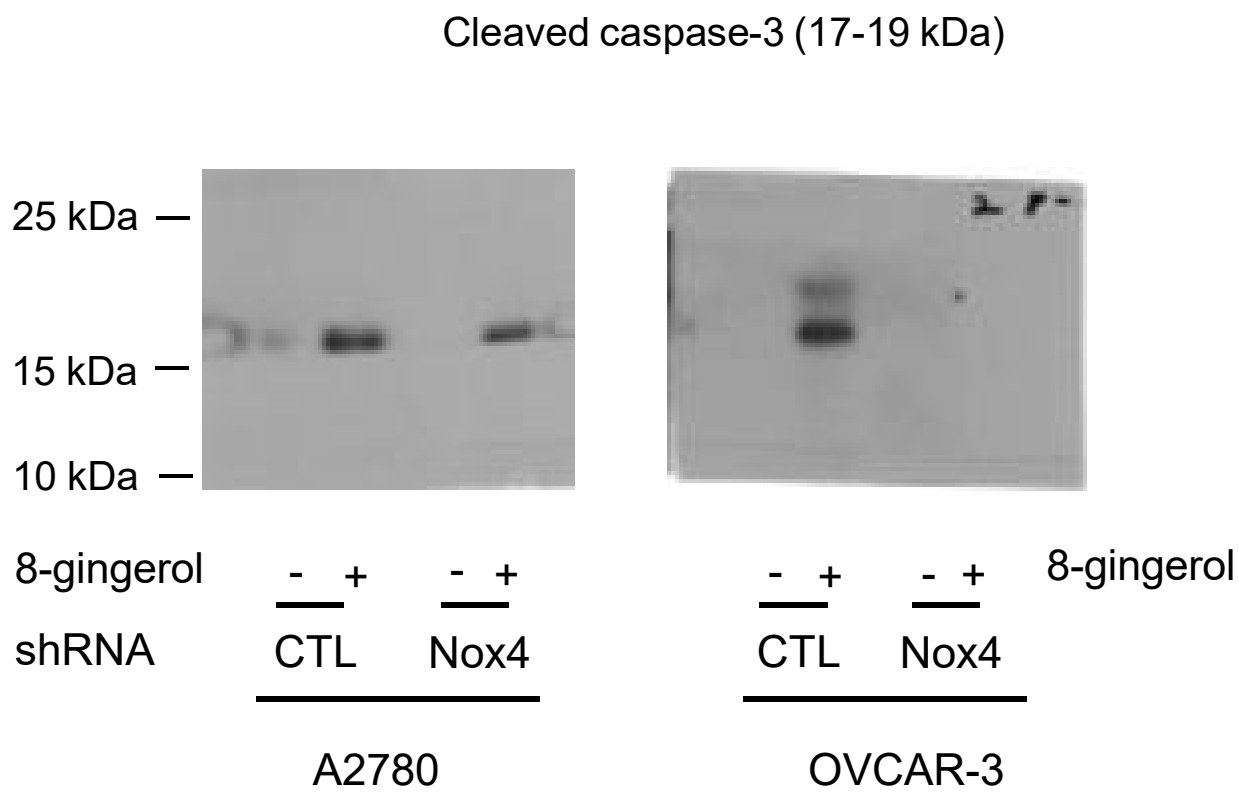

Whole blots for Figure 7B

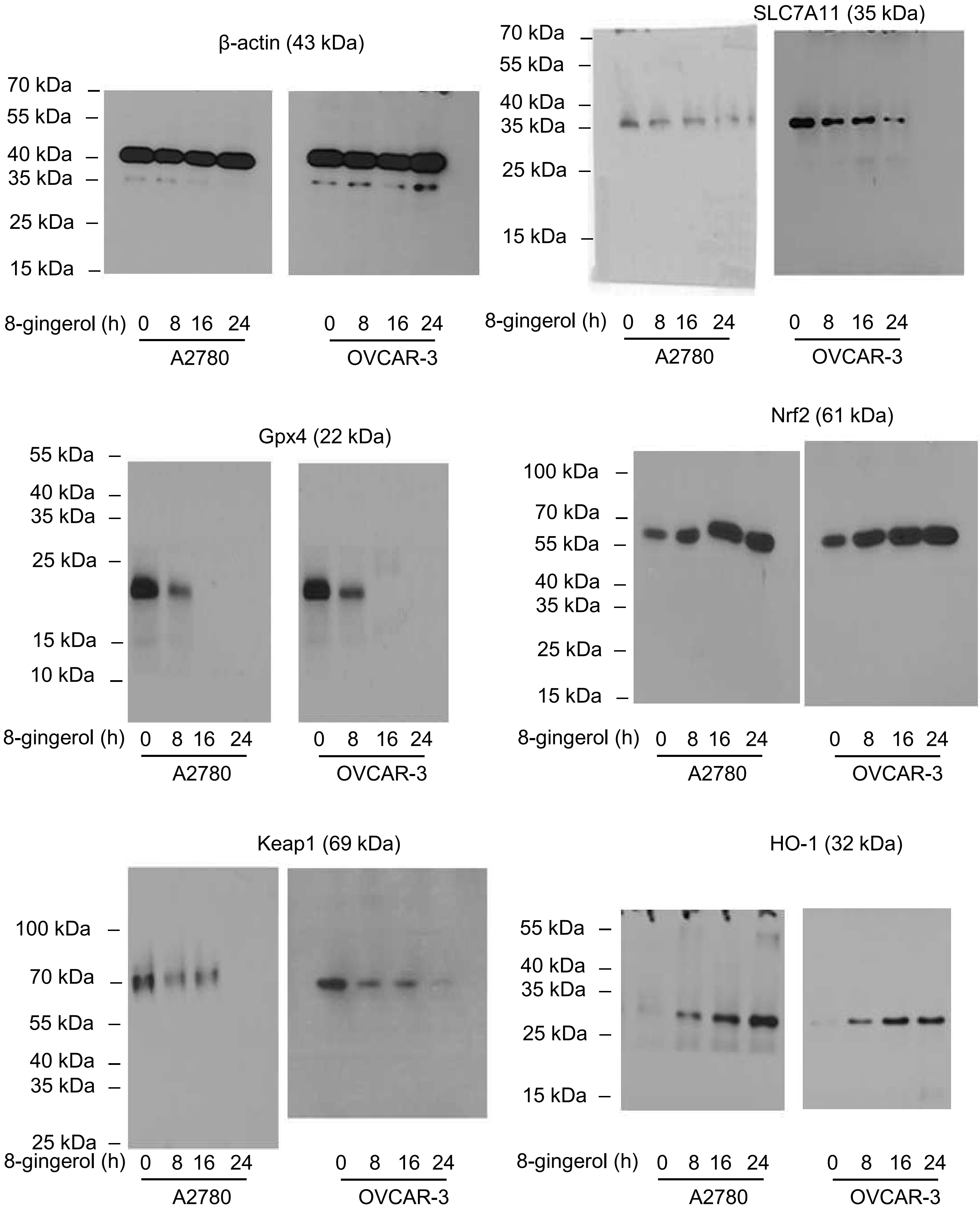

Whole blots for Figure 7H

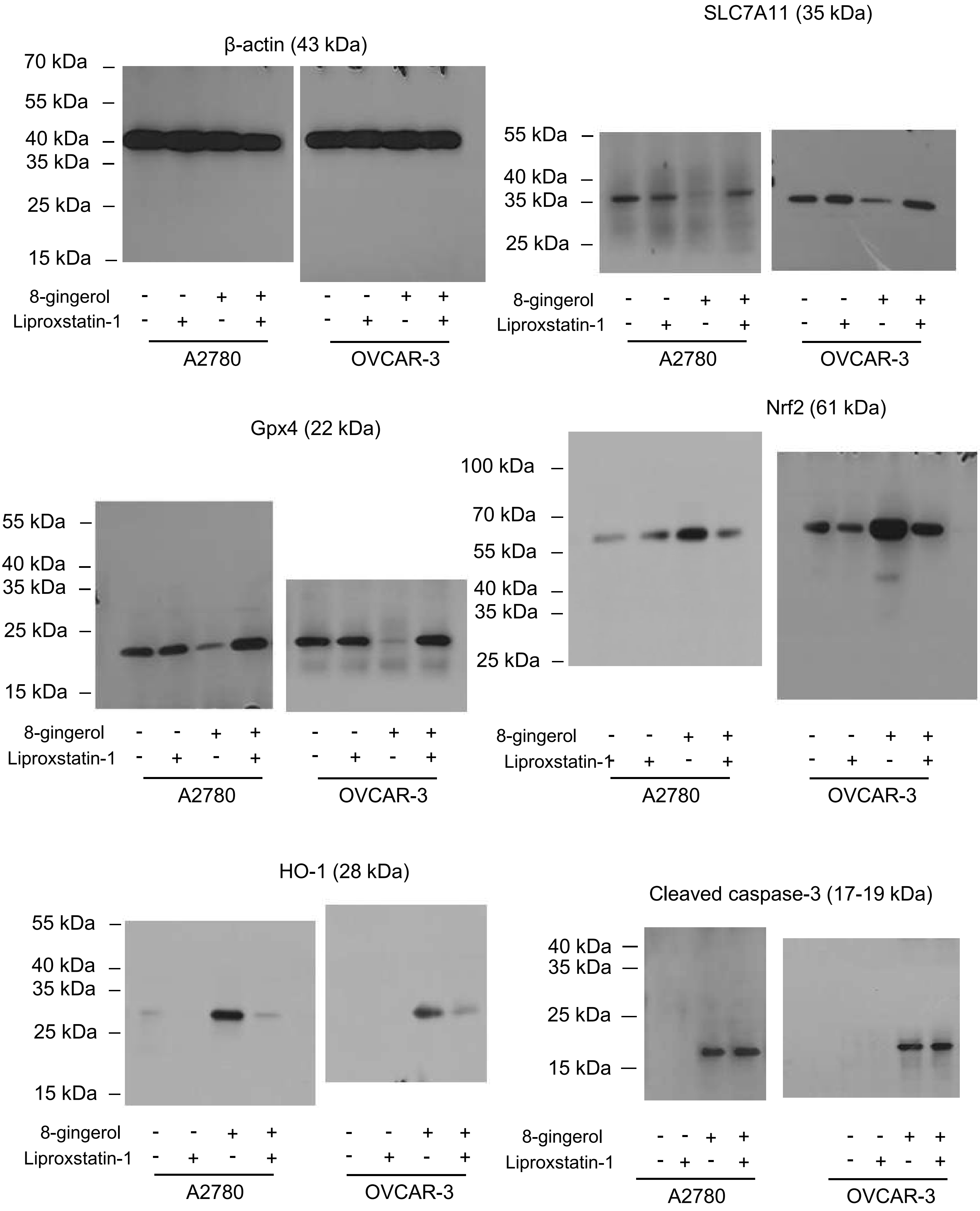

Whole blots for Figure 7L

β-actin (43 kDa)

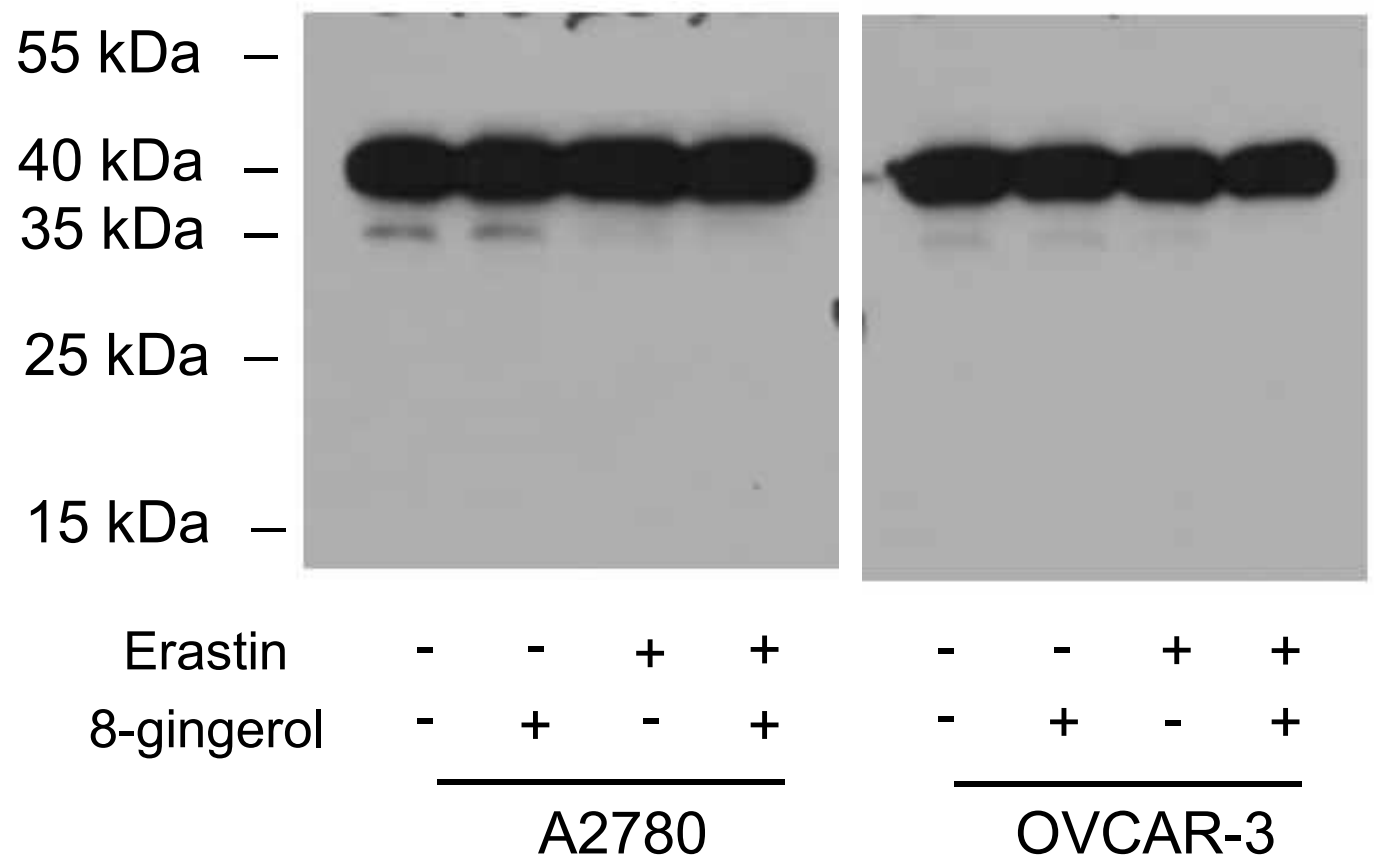

SLC7A11 (35 kDa)

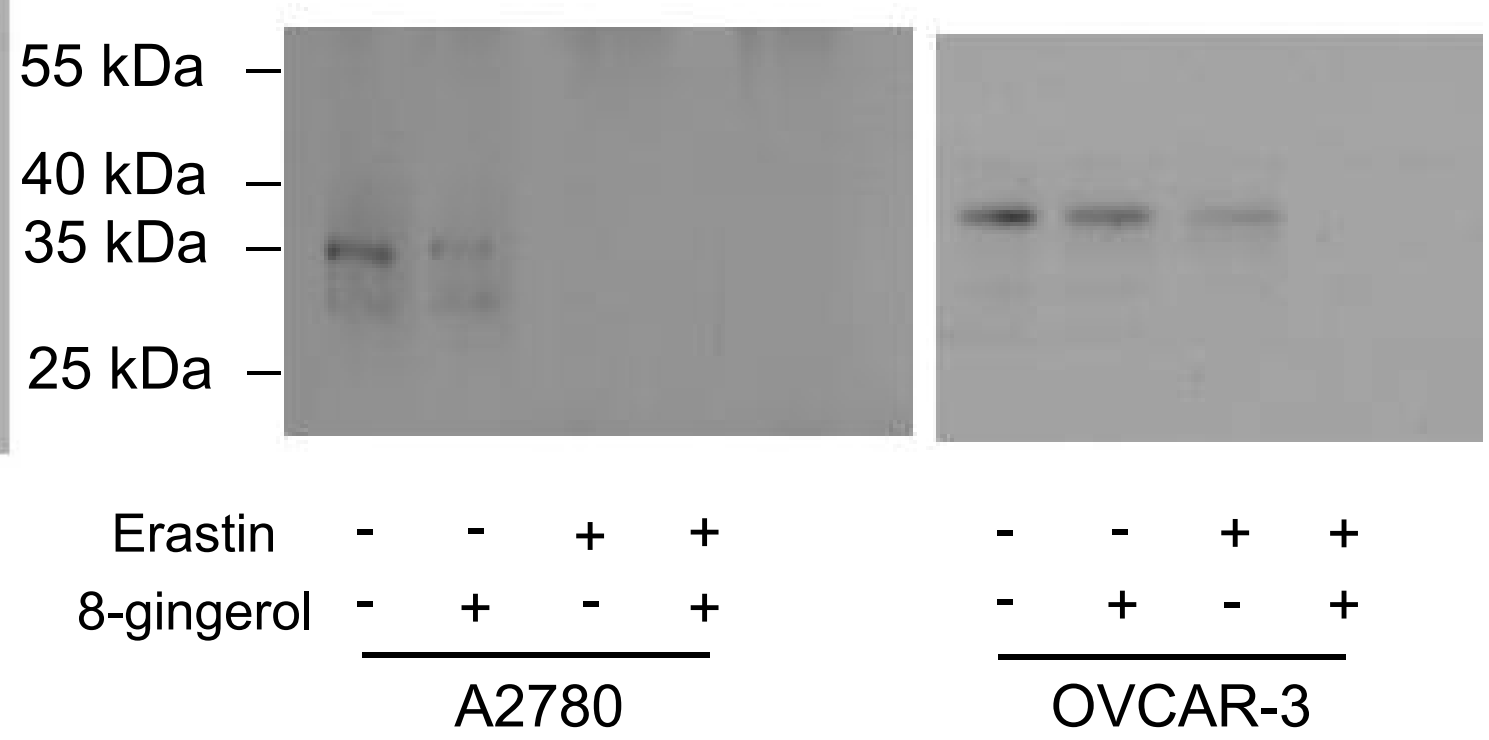

Gpx4 (22 kDa)

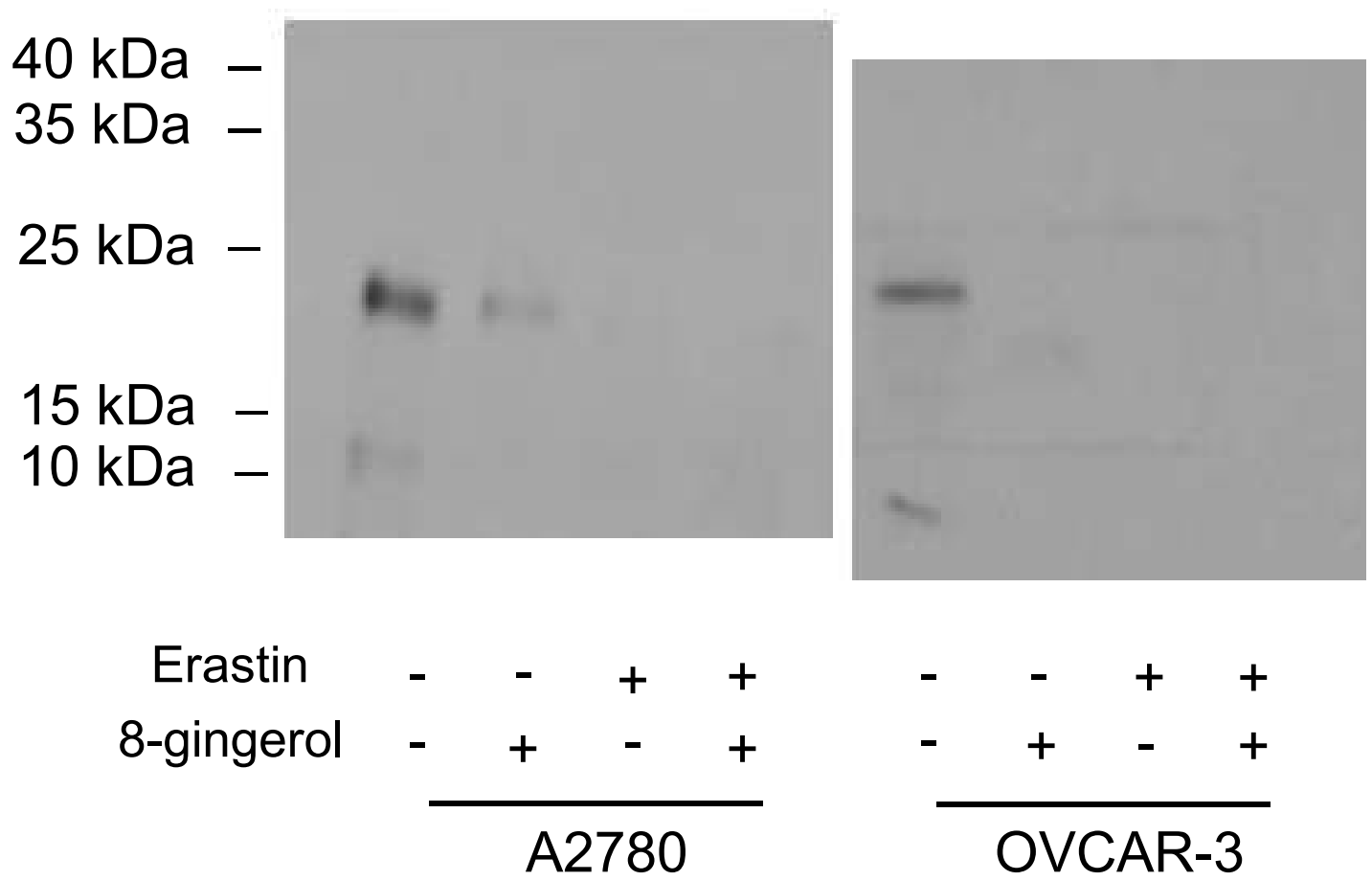

Nrf2 (61 kDa)

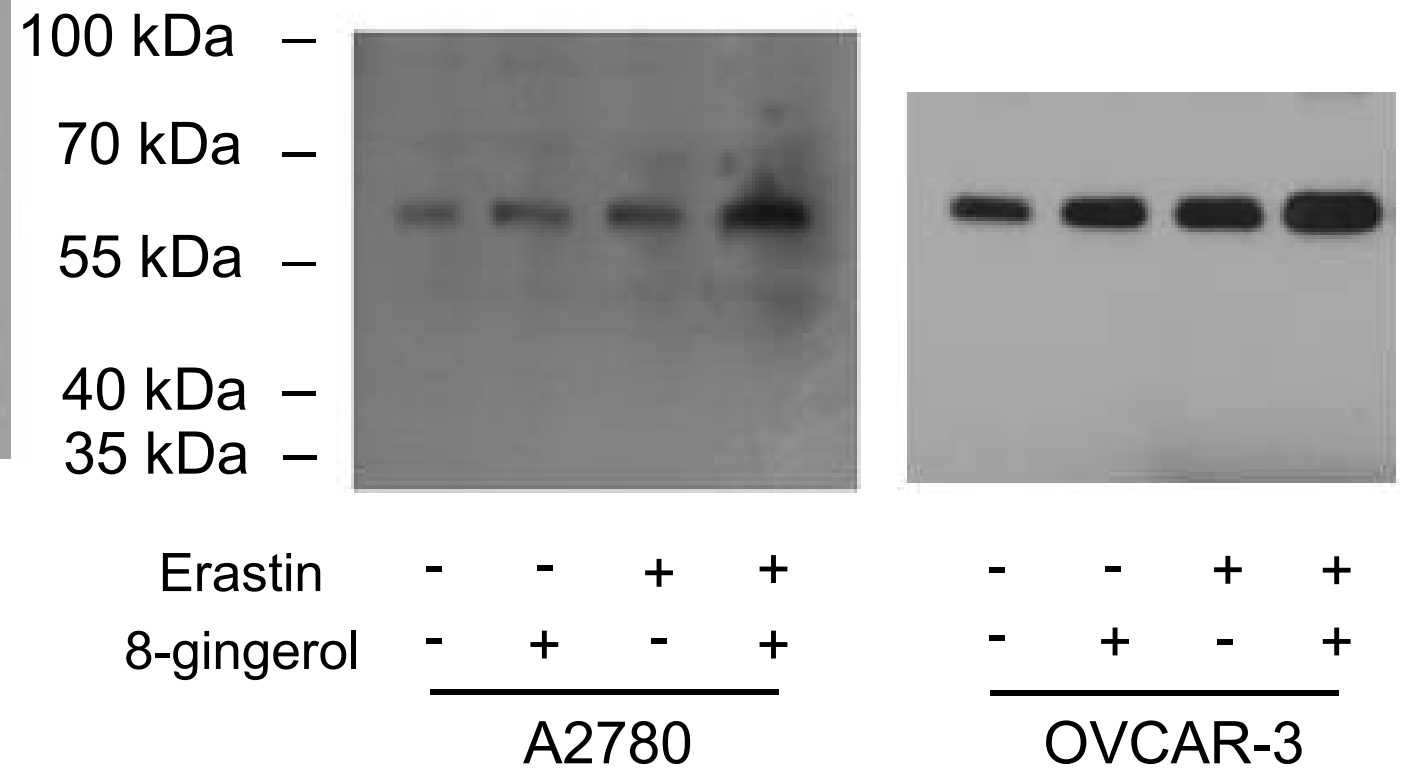

HO-1 (28 kDa)

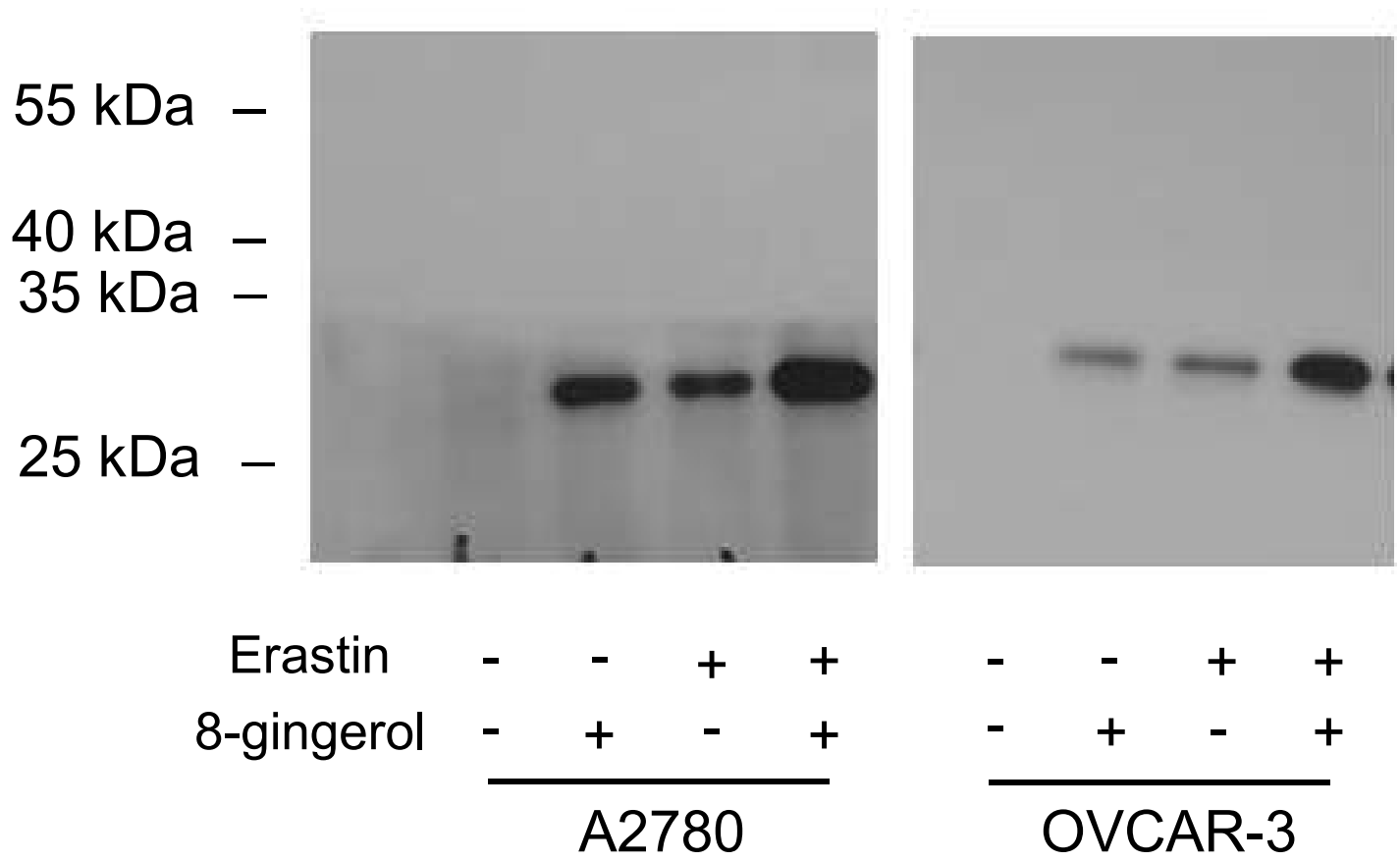

Whole blots for Figure 8A

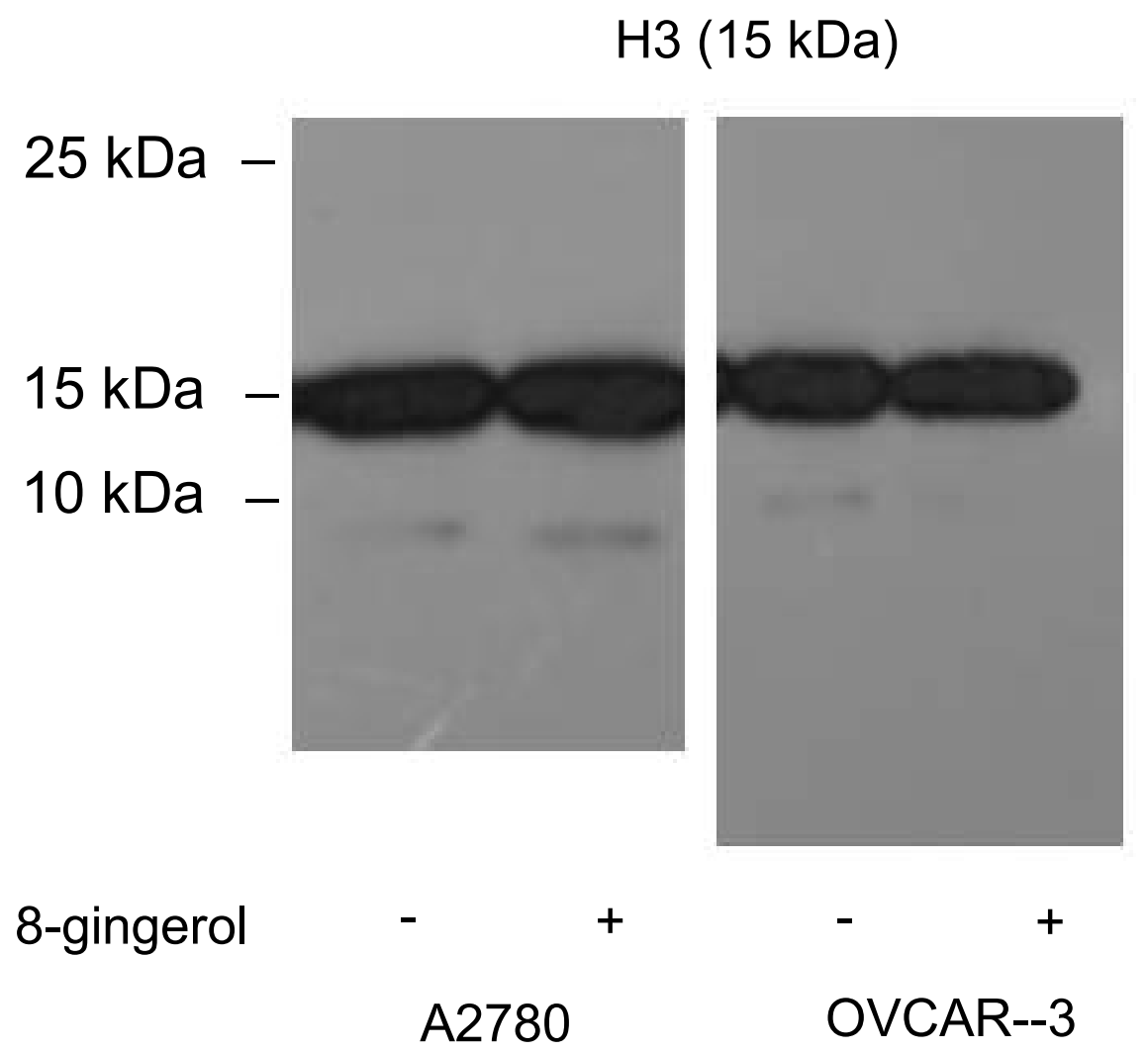

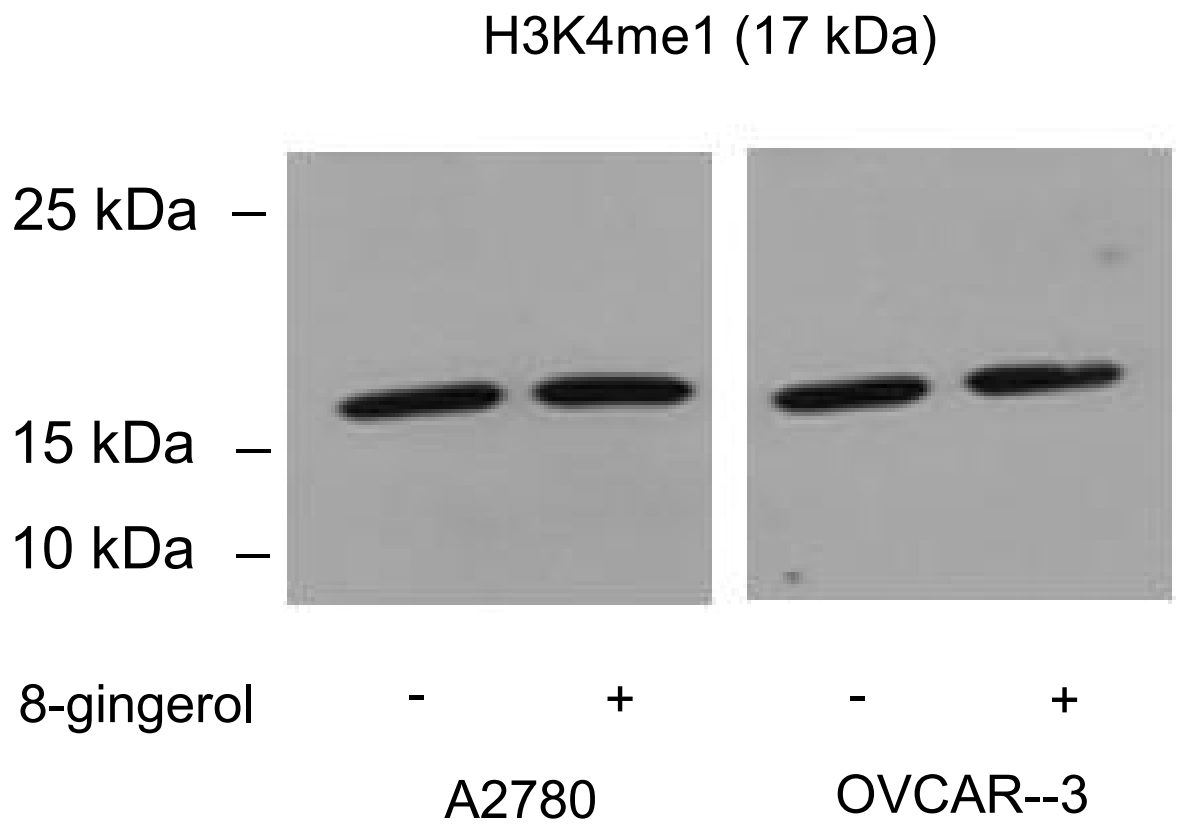

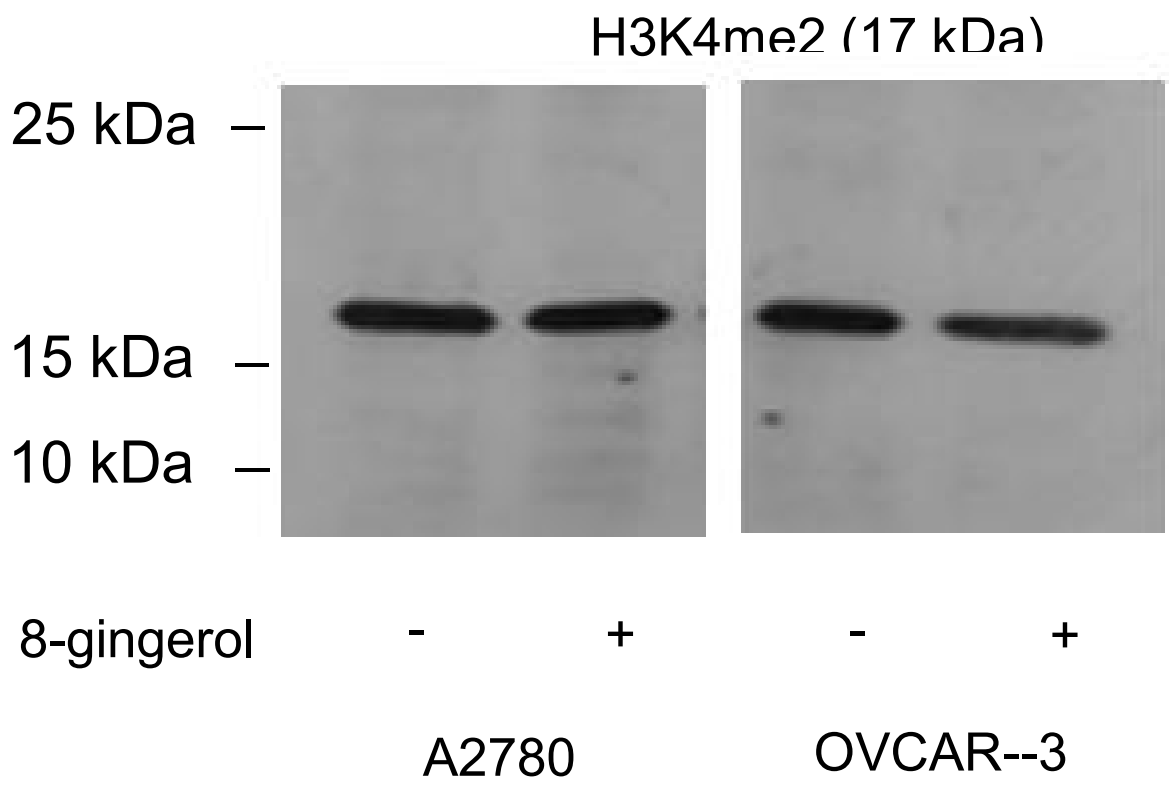

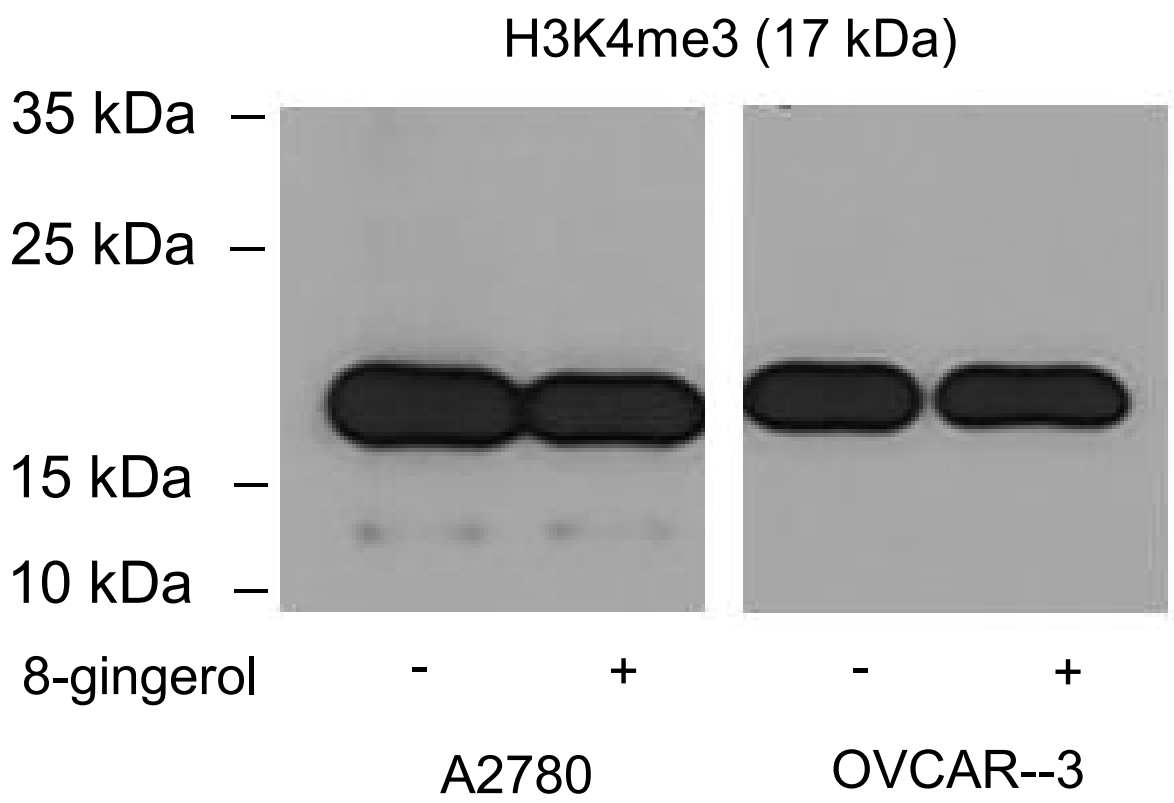

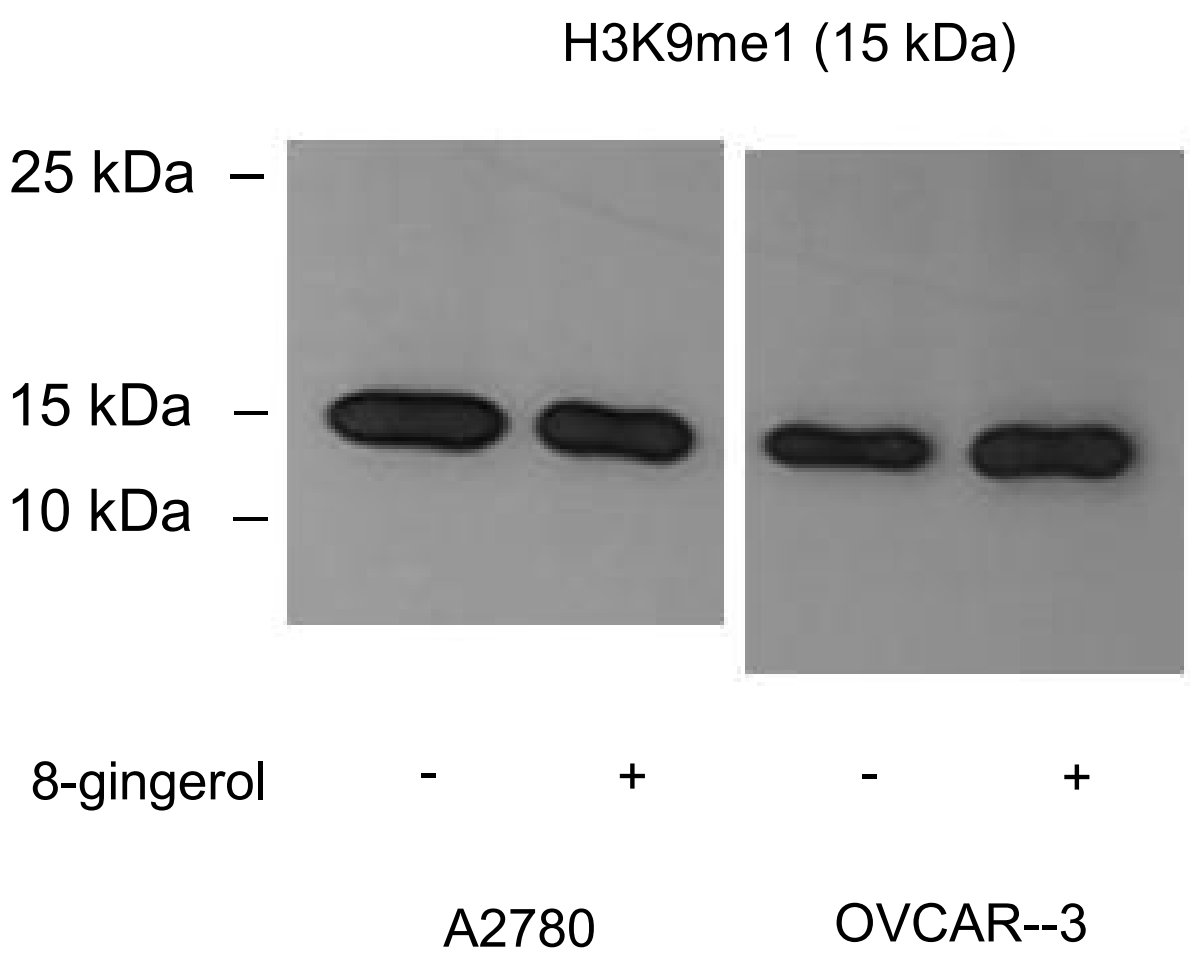

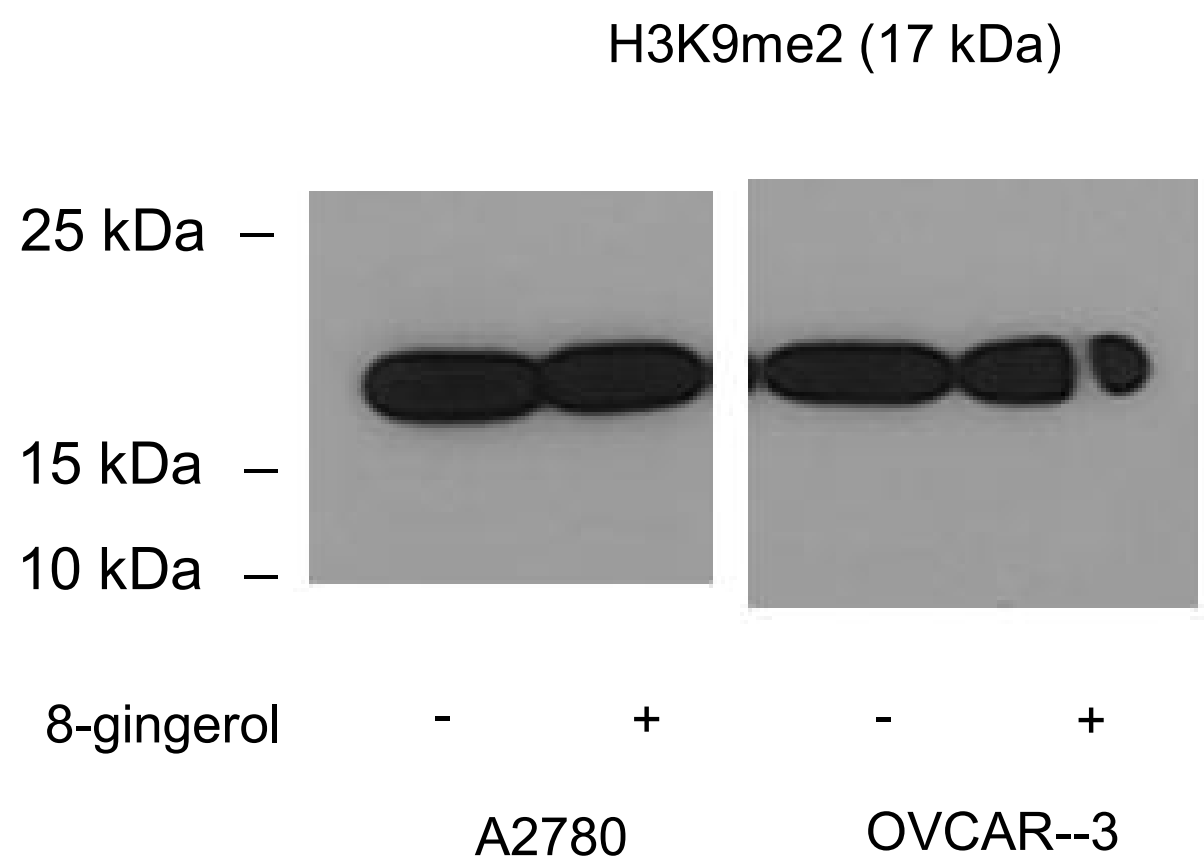

Whole blots for Figure 8A

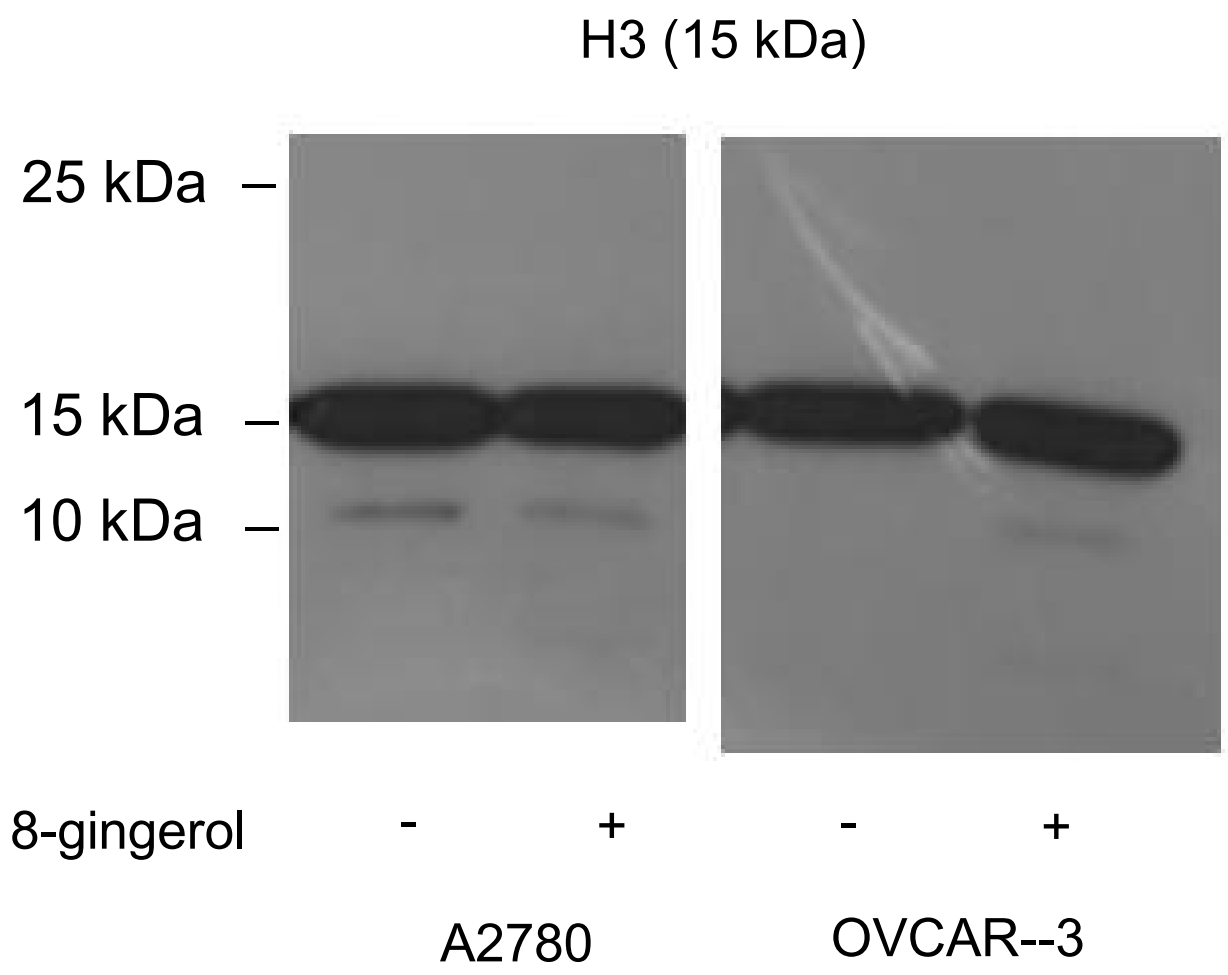

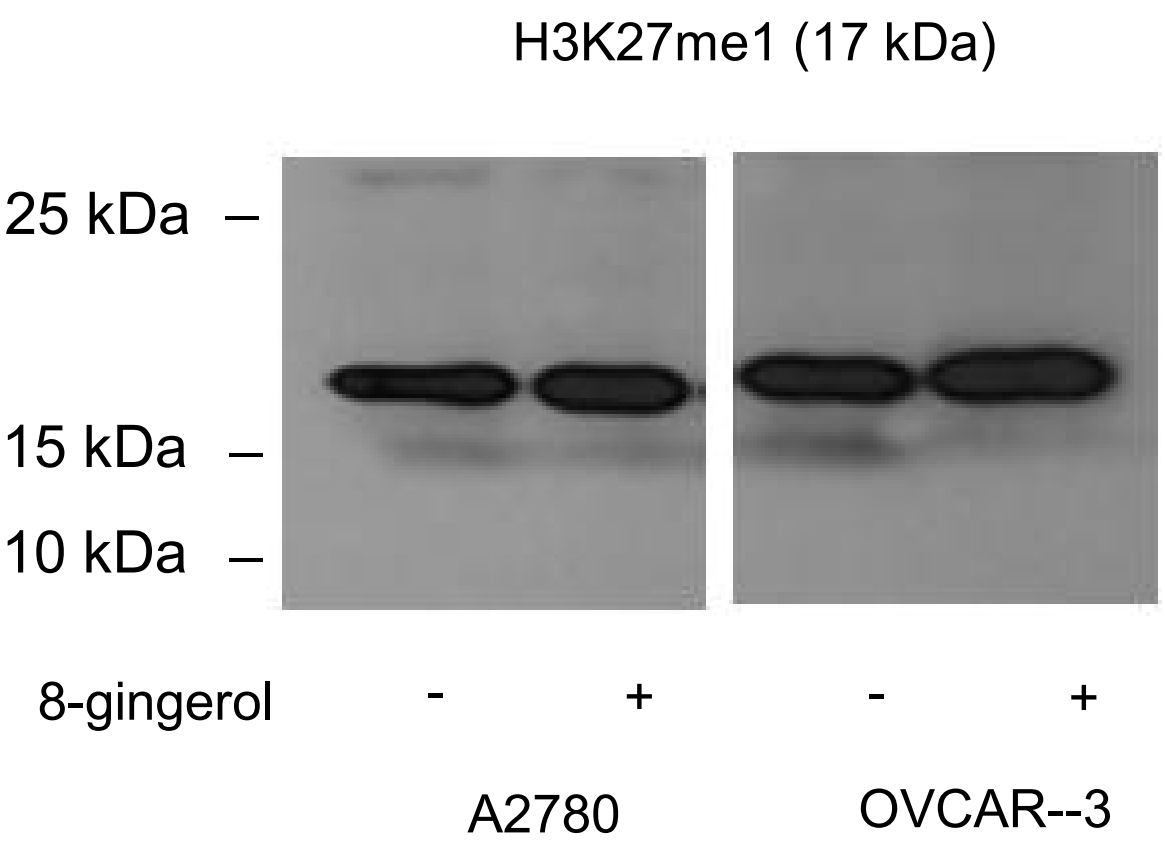

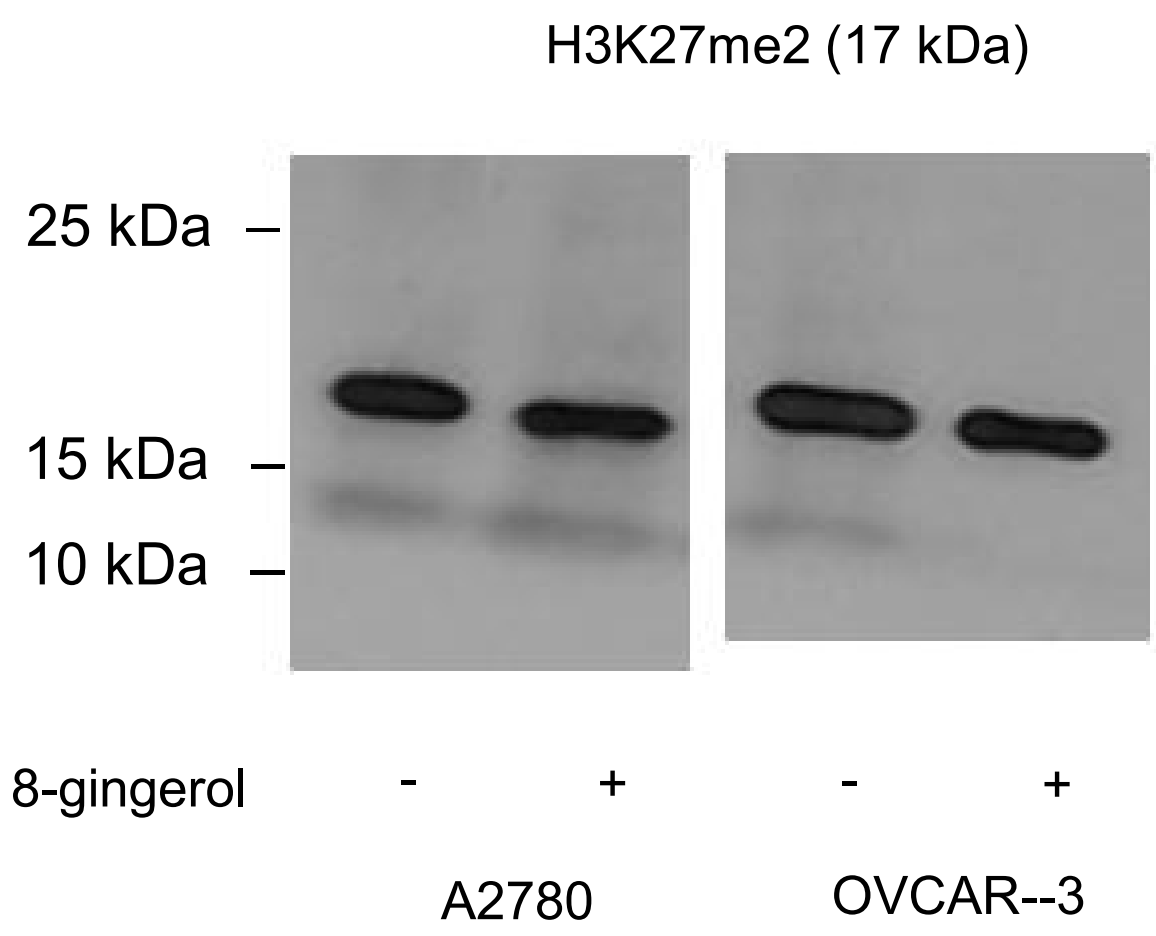

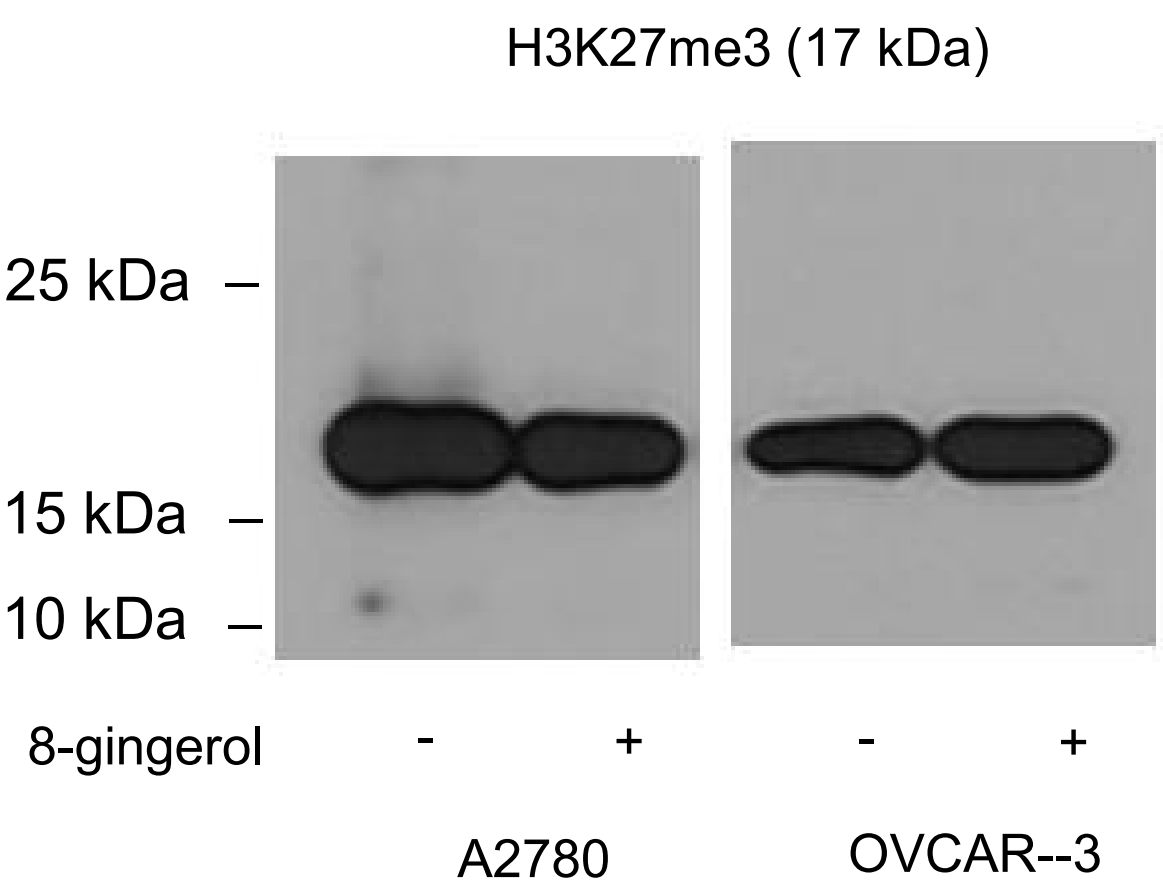

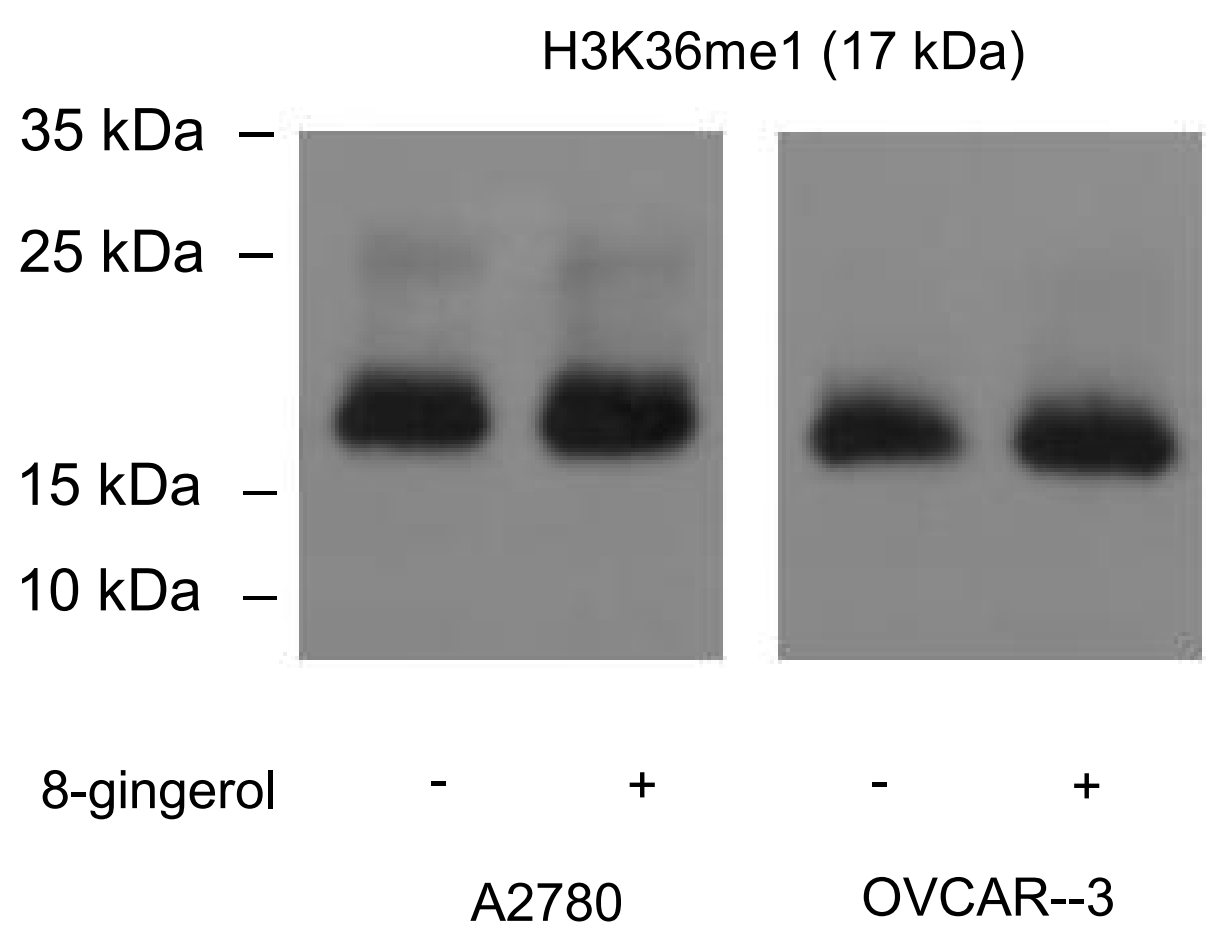

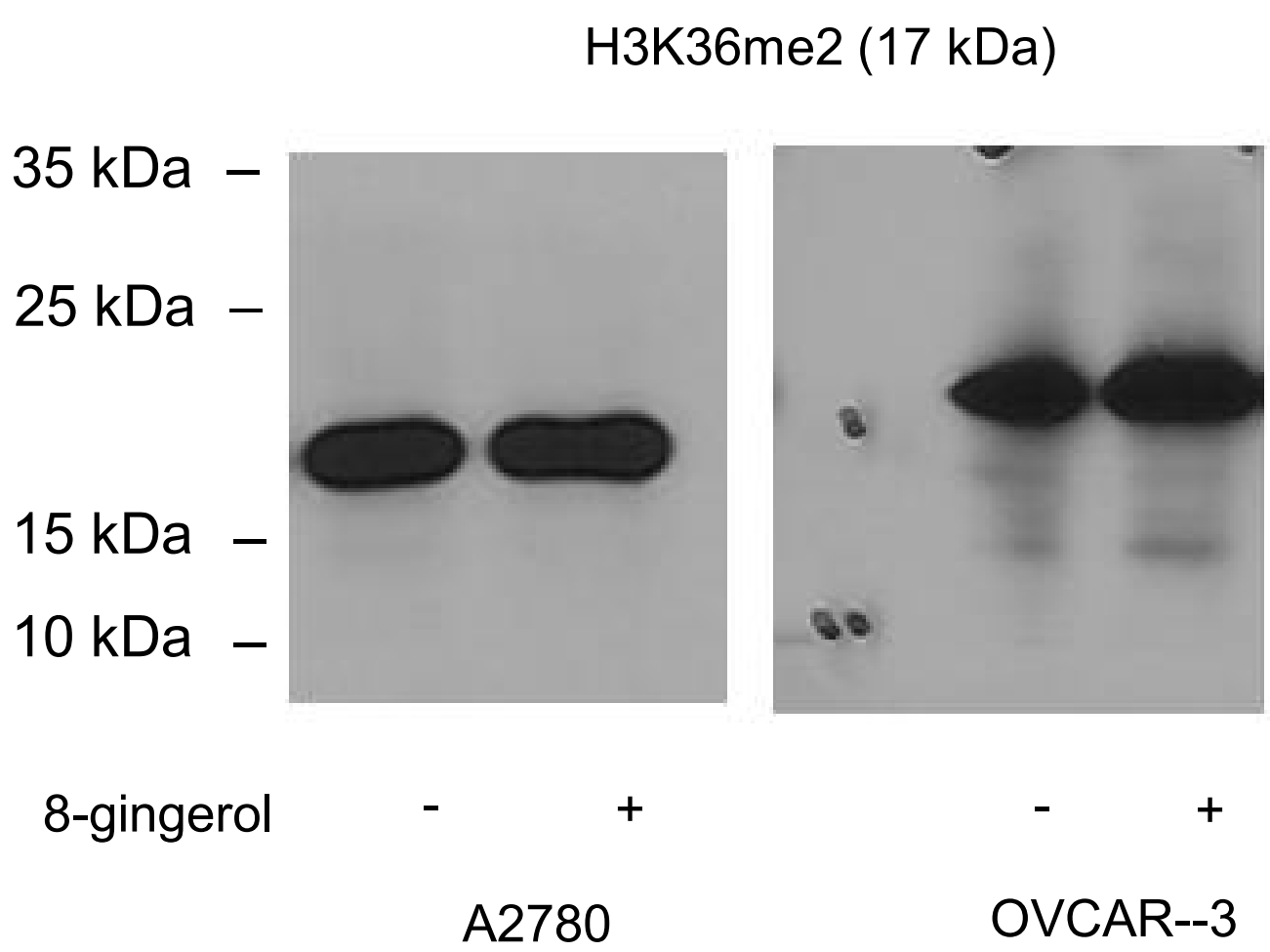

Whole blots for Figure 8A

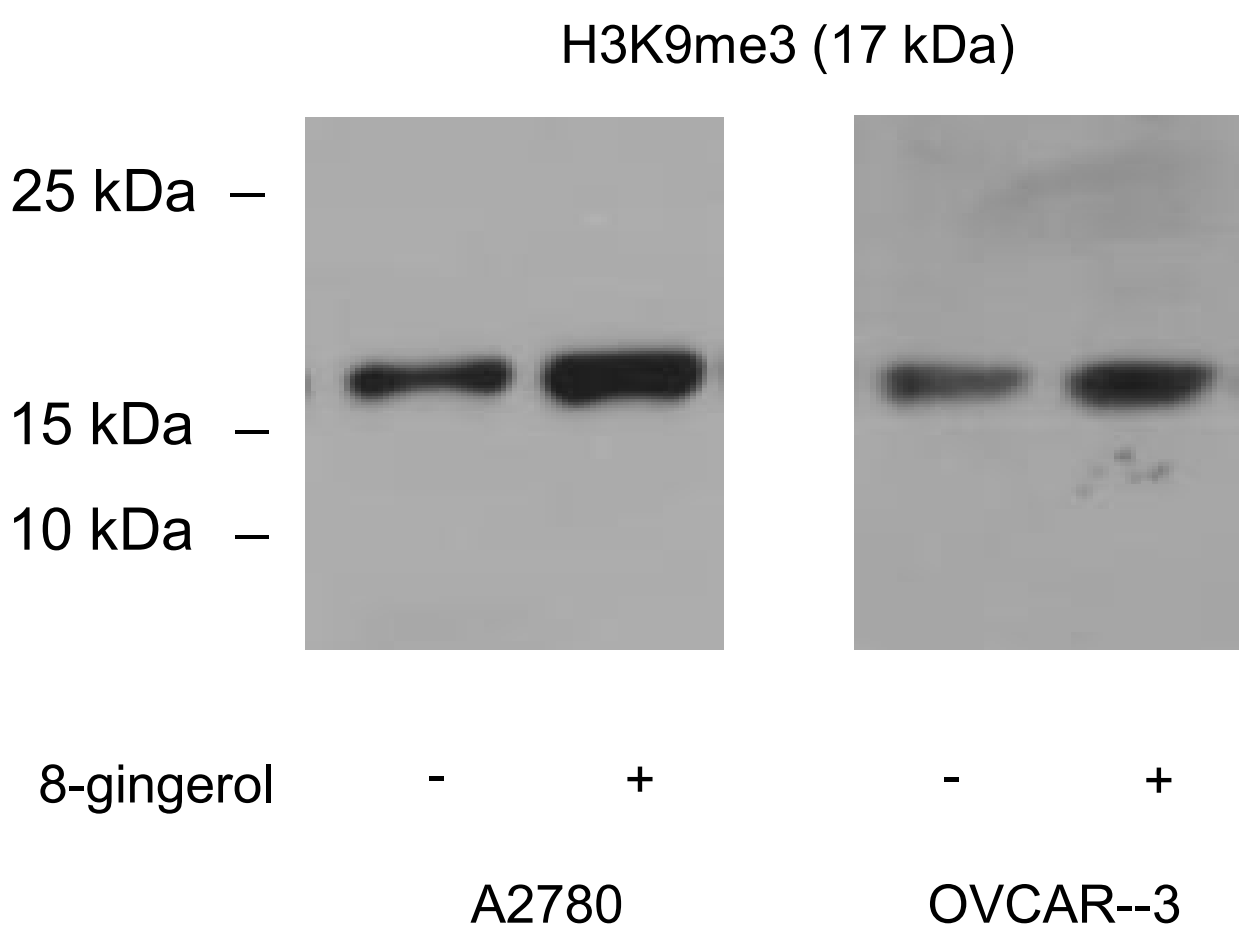

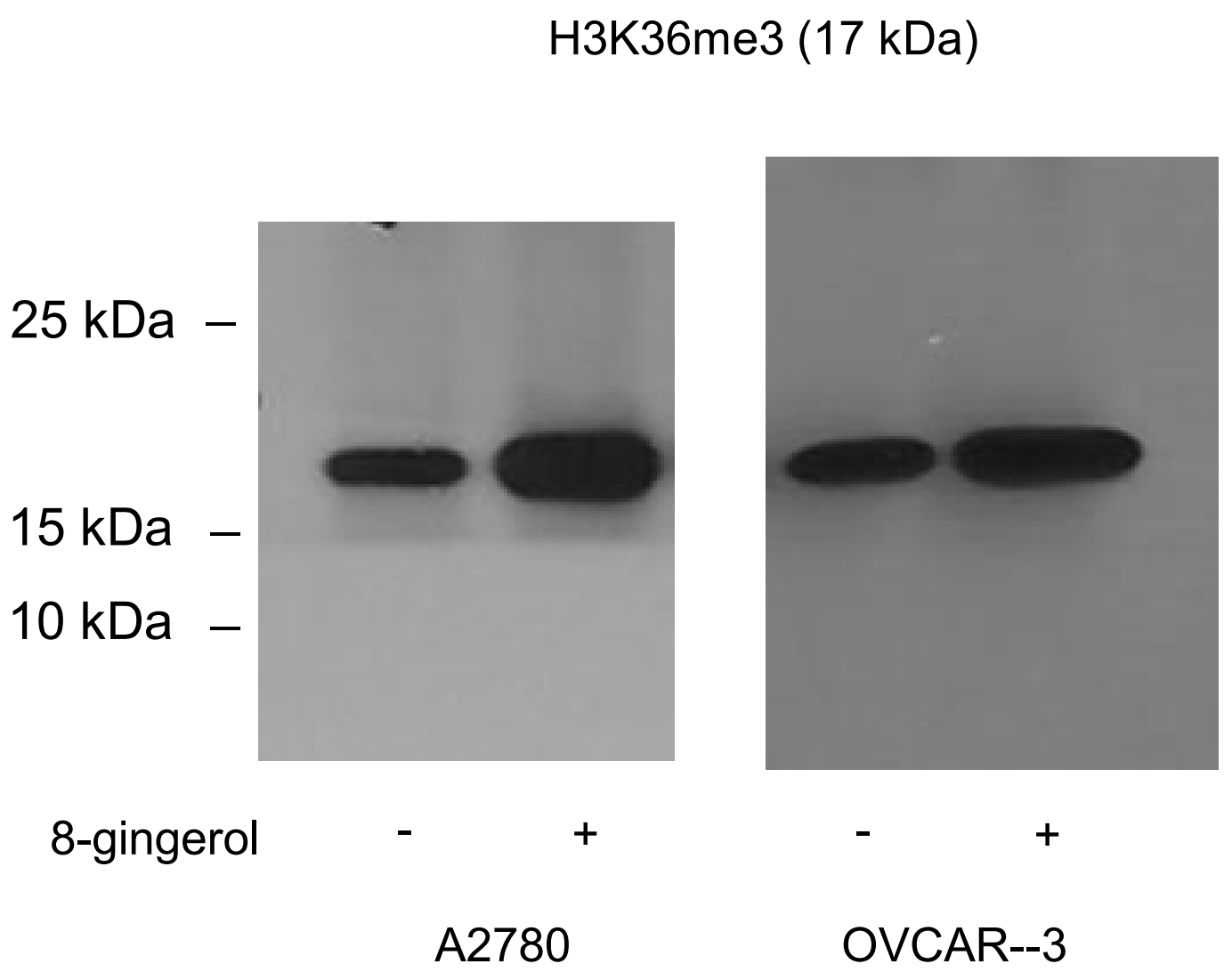

Whole blots for Figure 8B

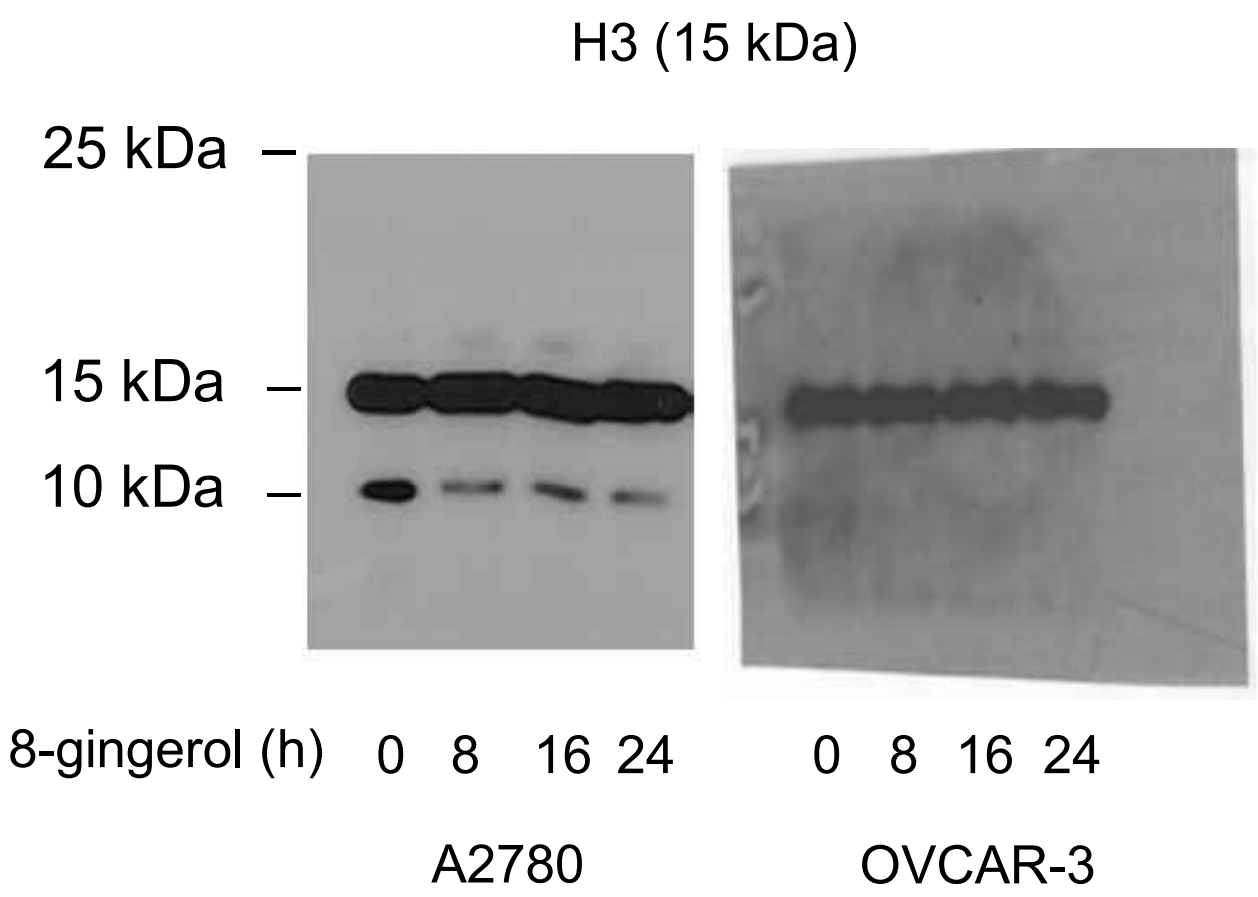

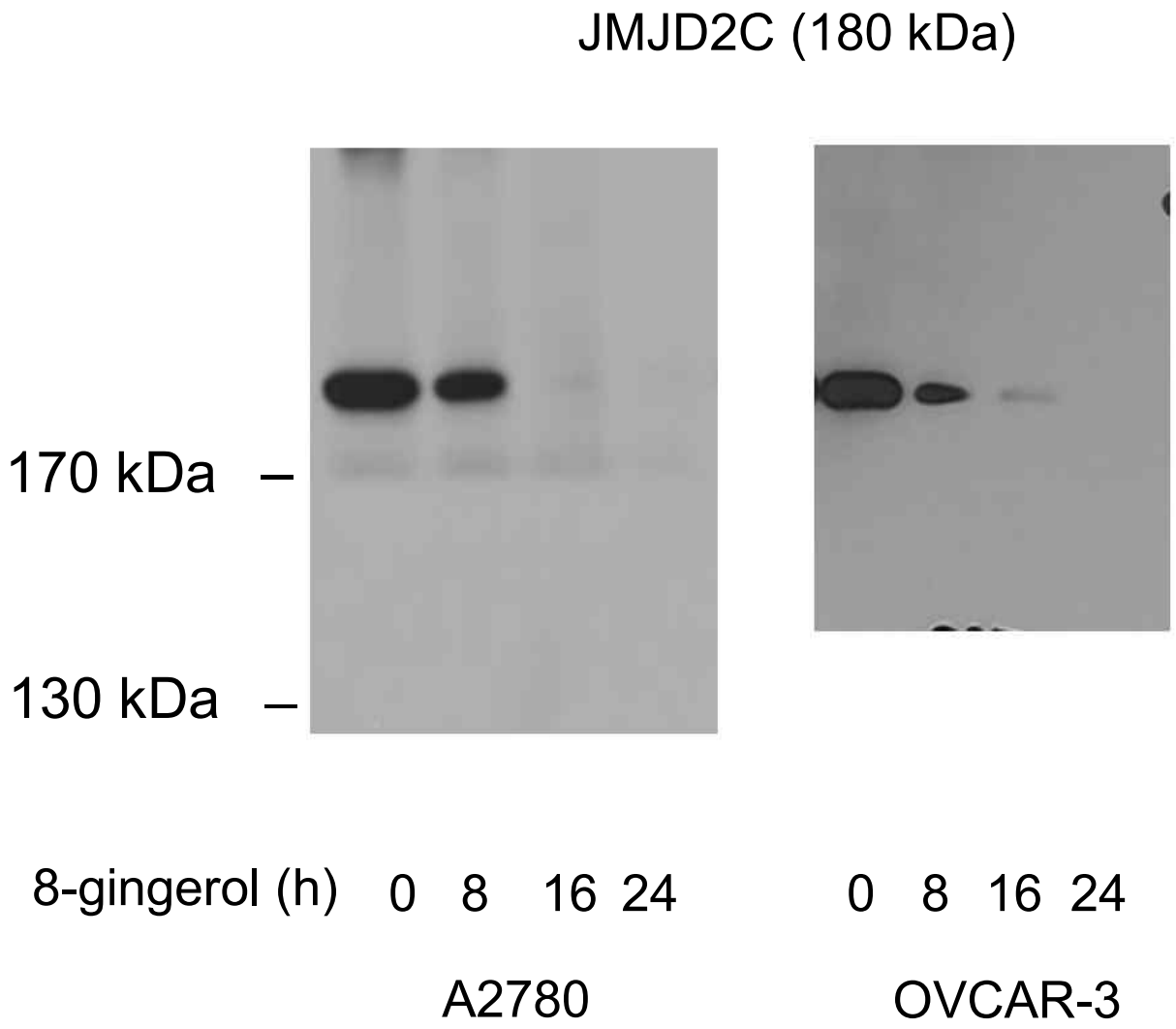

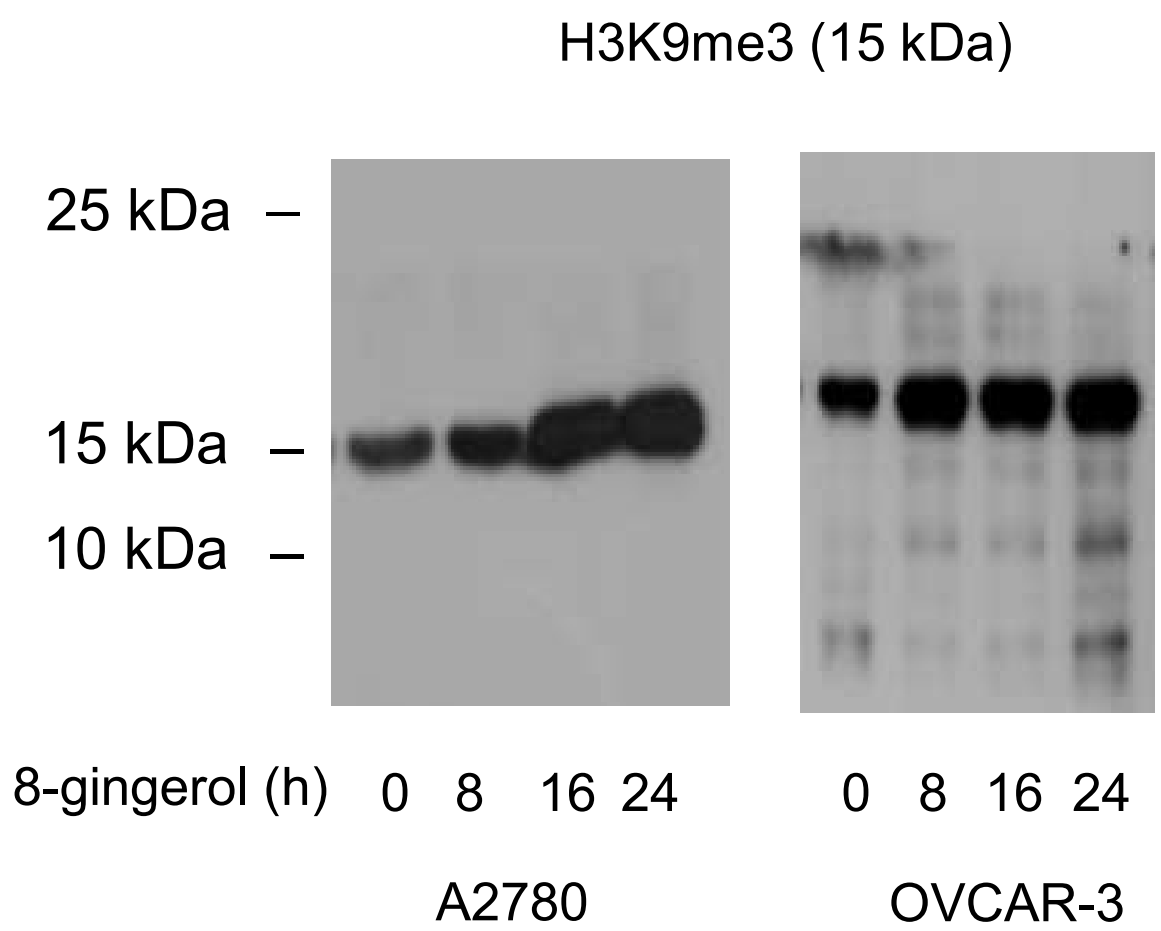

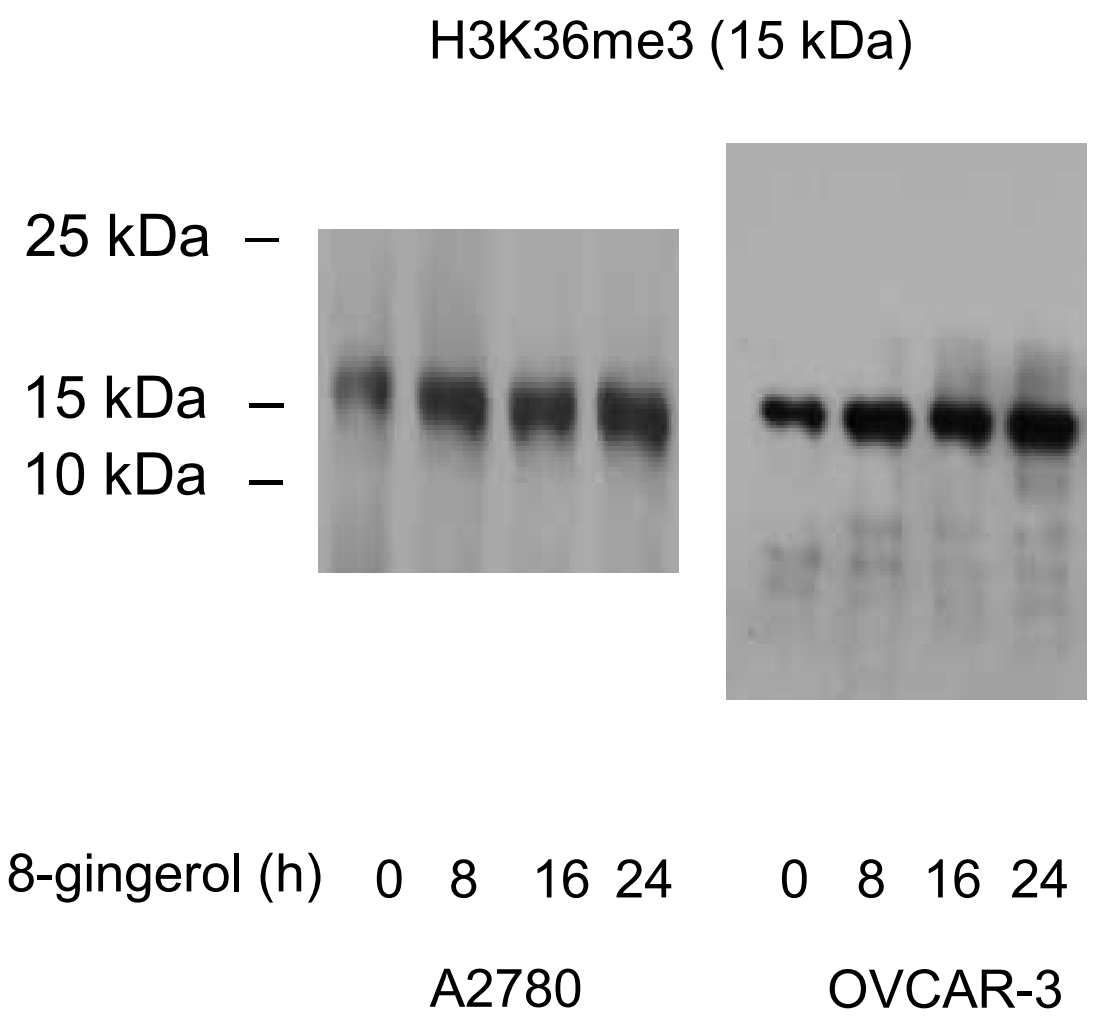

Whole blots for Figure 8H

β-actin (43 kDa)

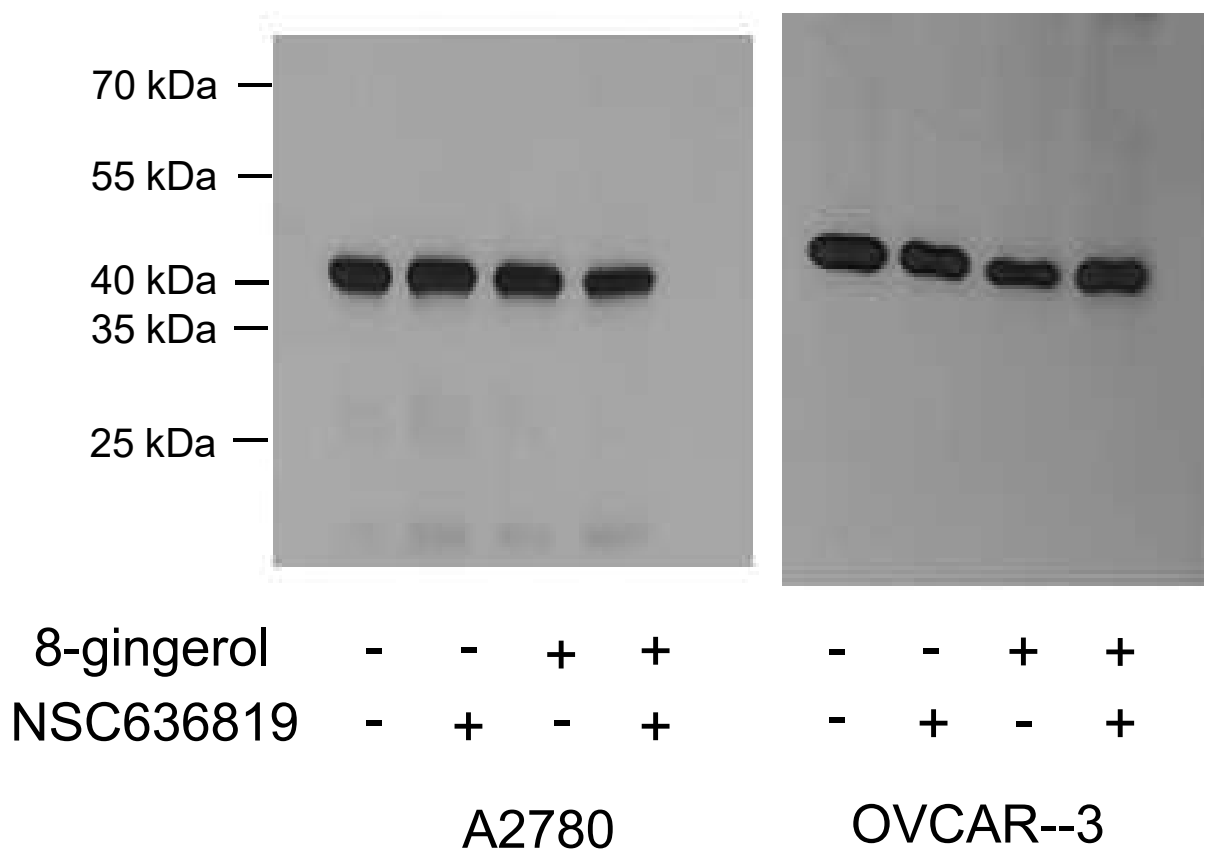

ATF3 (21 kDa)

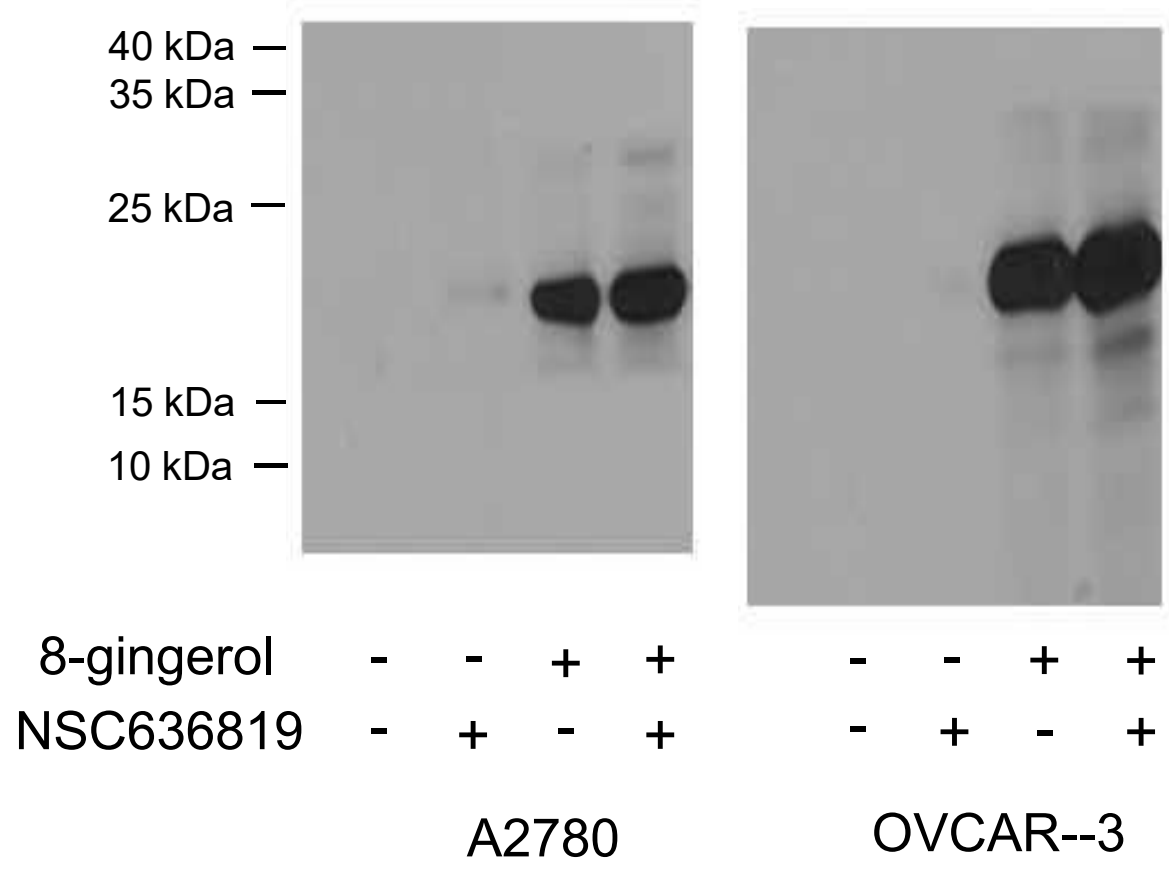

JMJD2C (180 kDa)

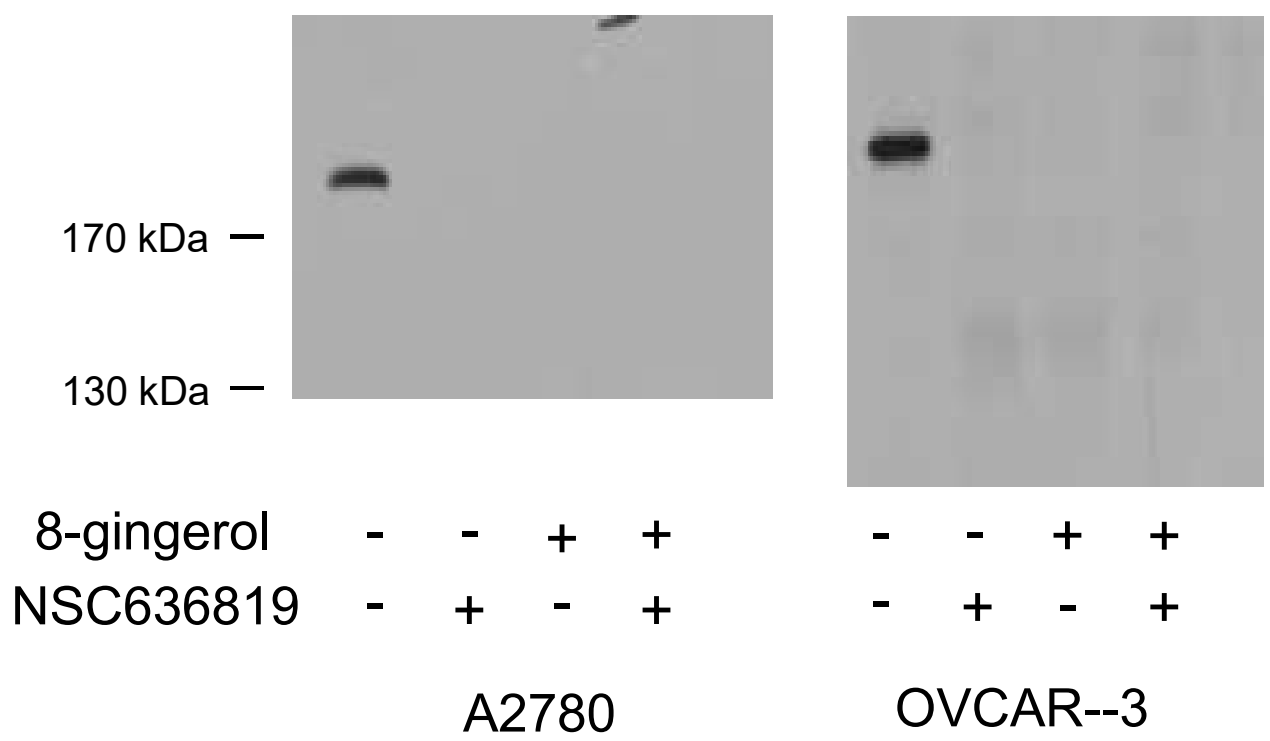

SLC7A11 (35 kDa)

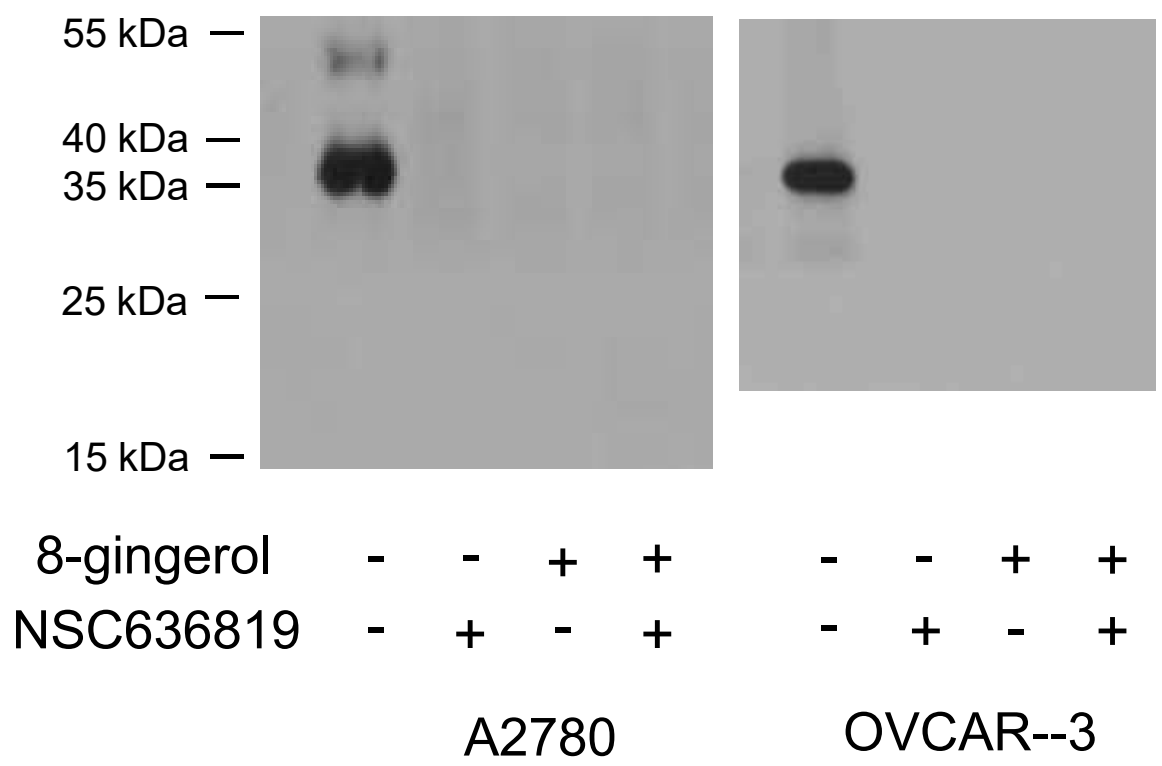

Gpx4 (22 kDa)

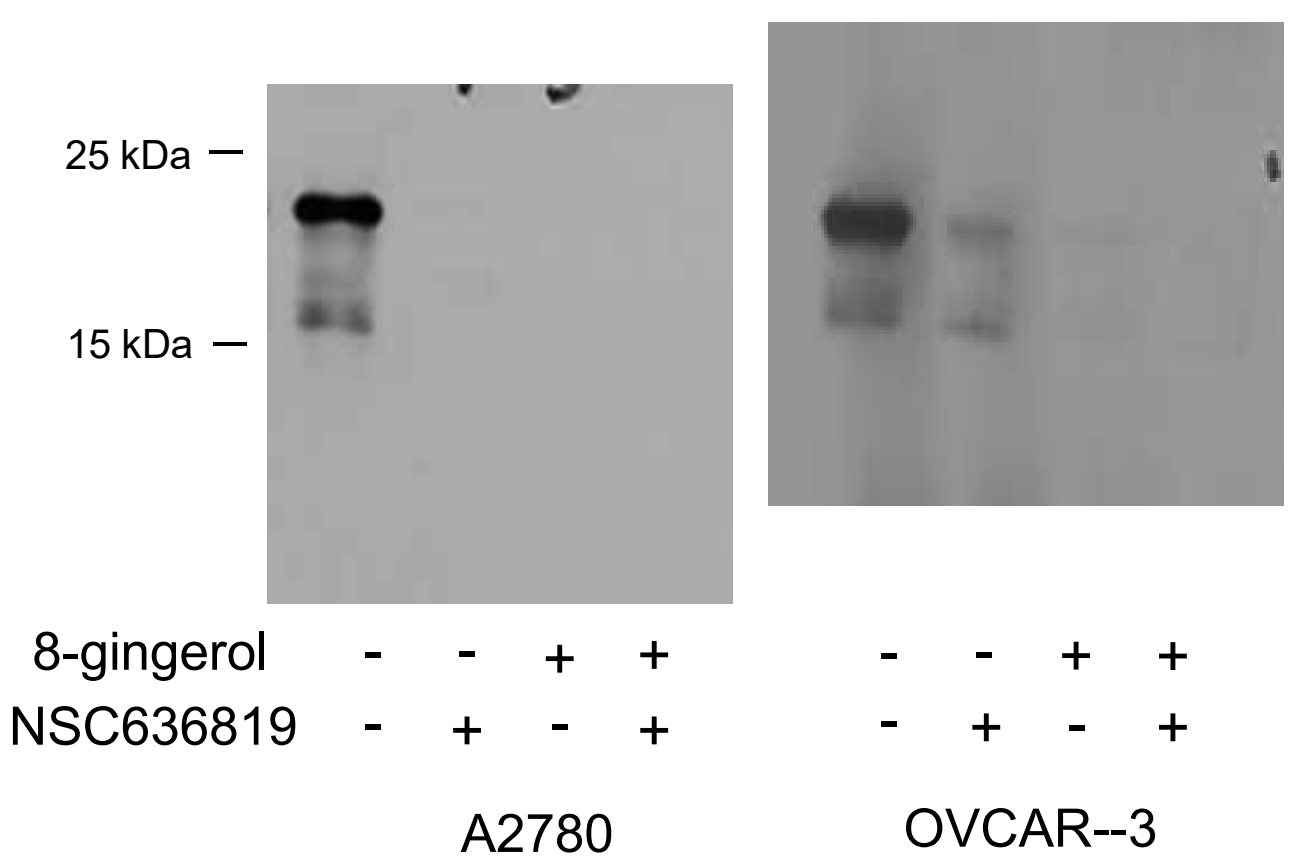

Whole blots for Figure 8K

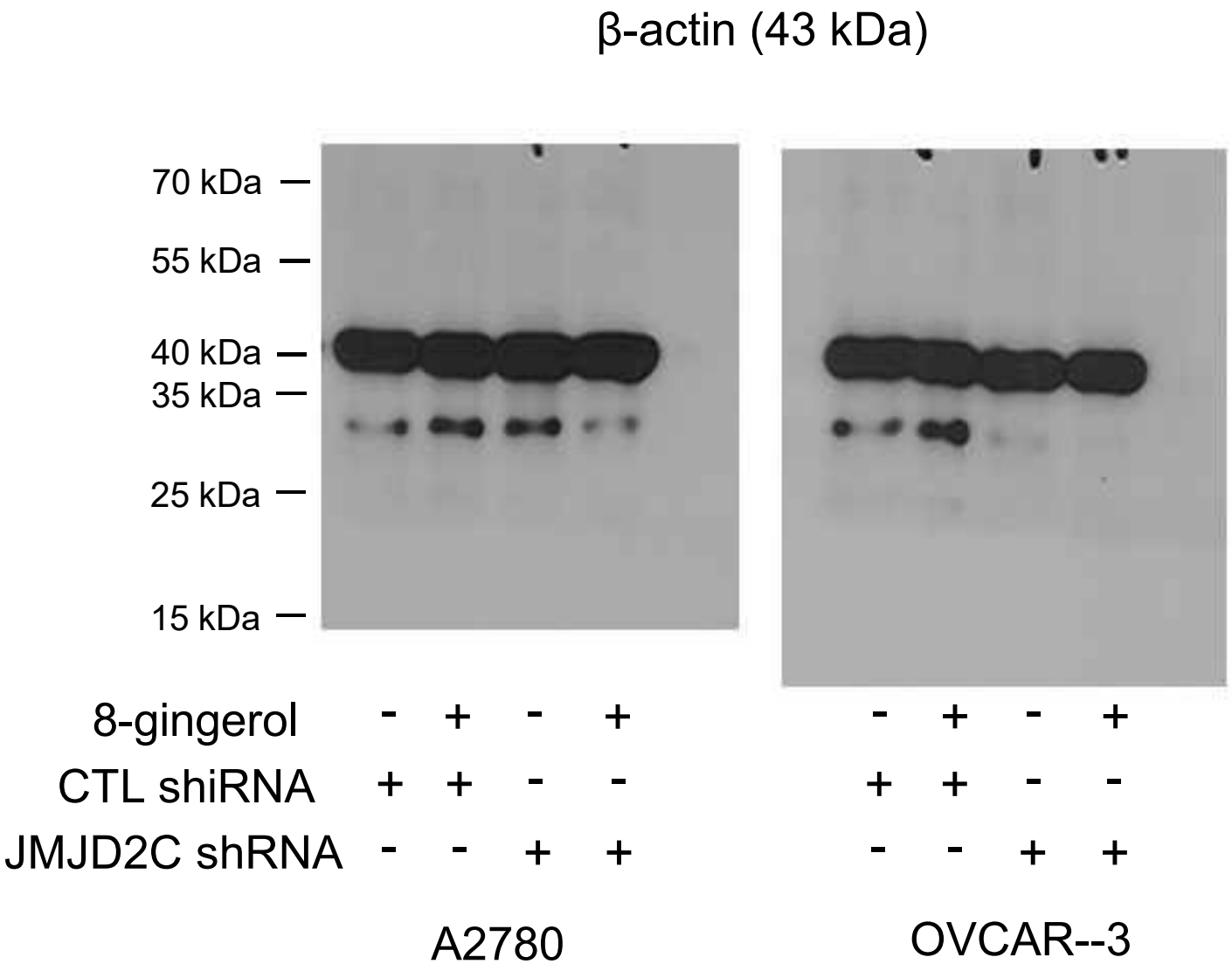

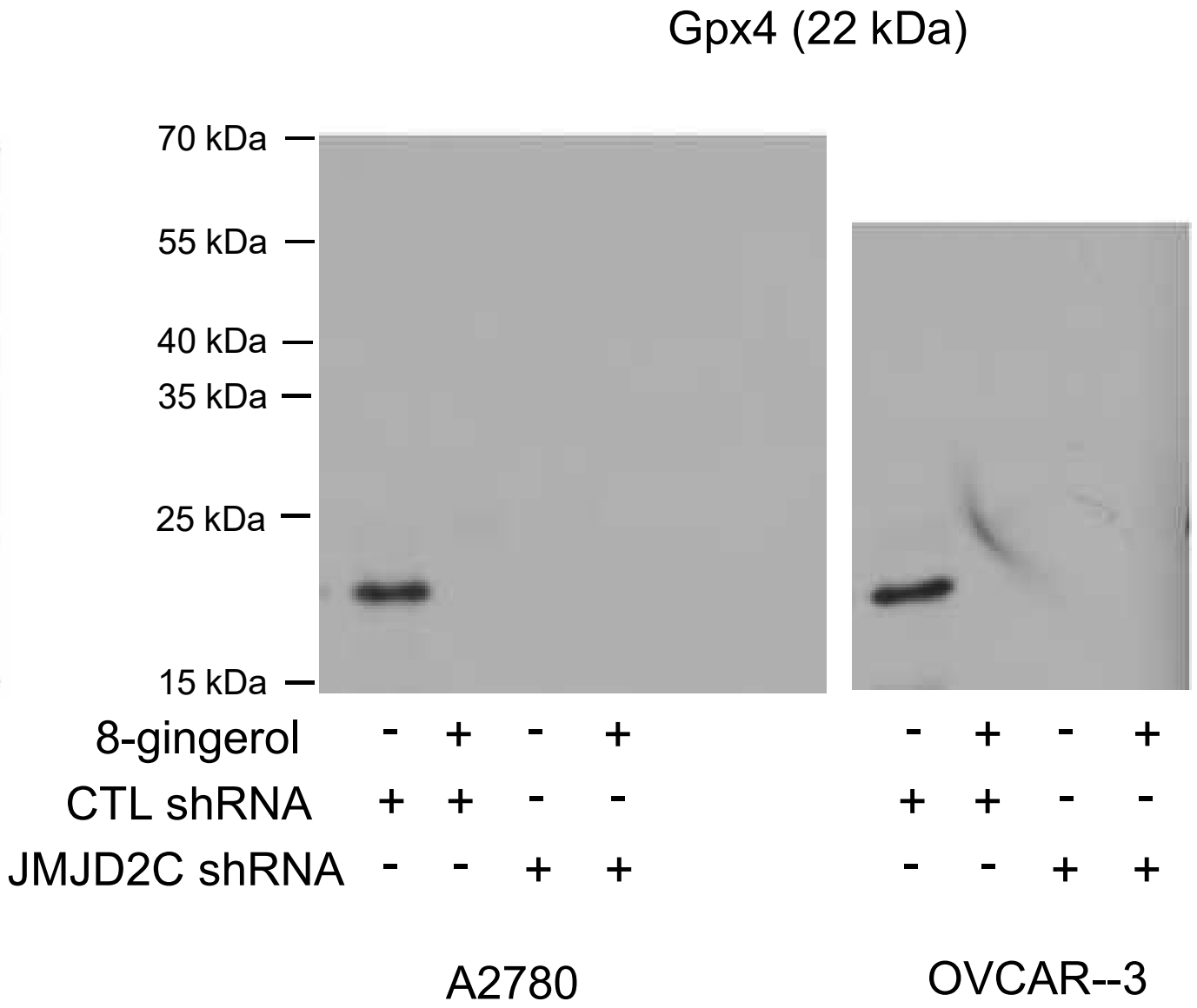

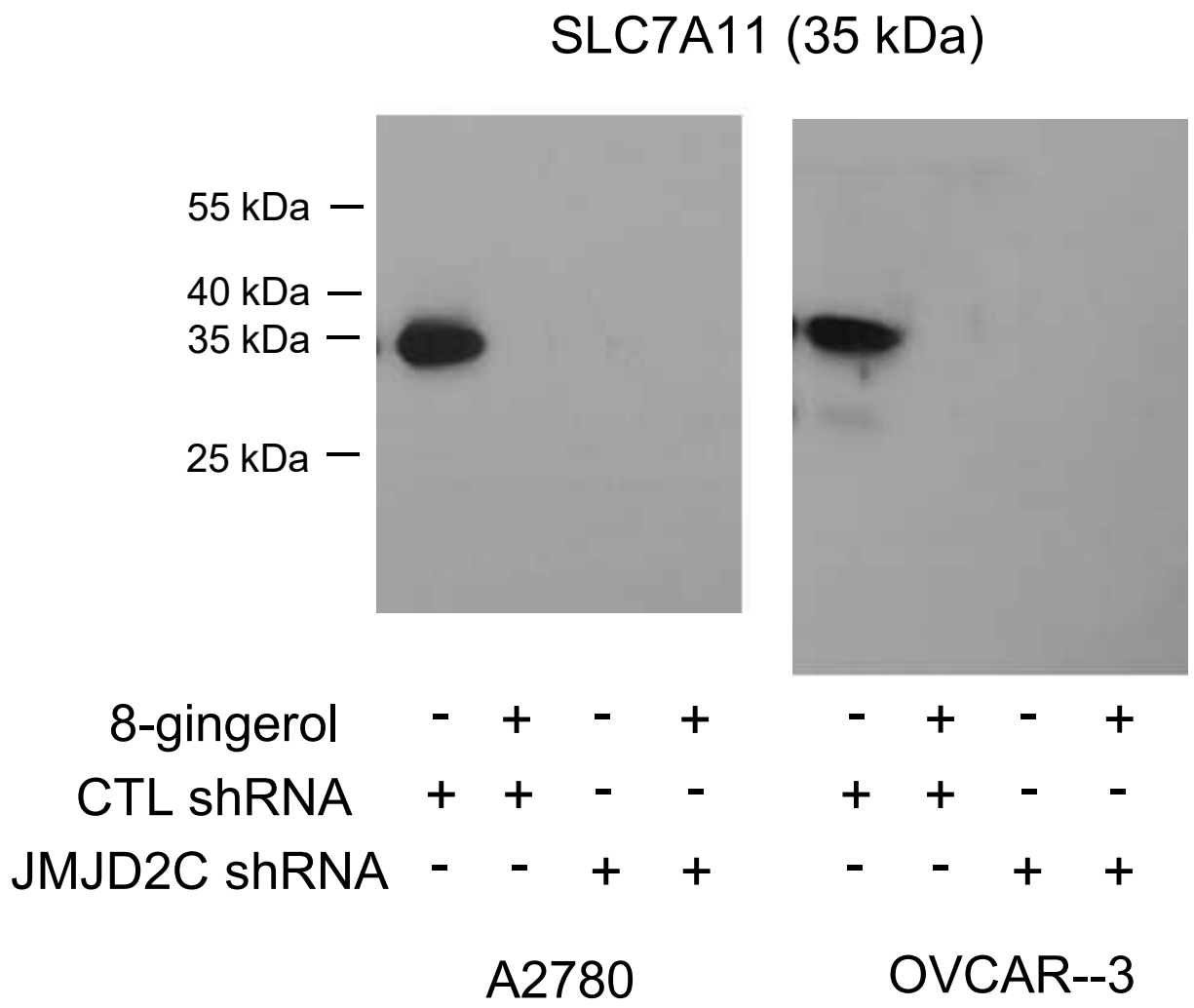

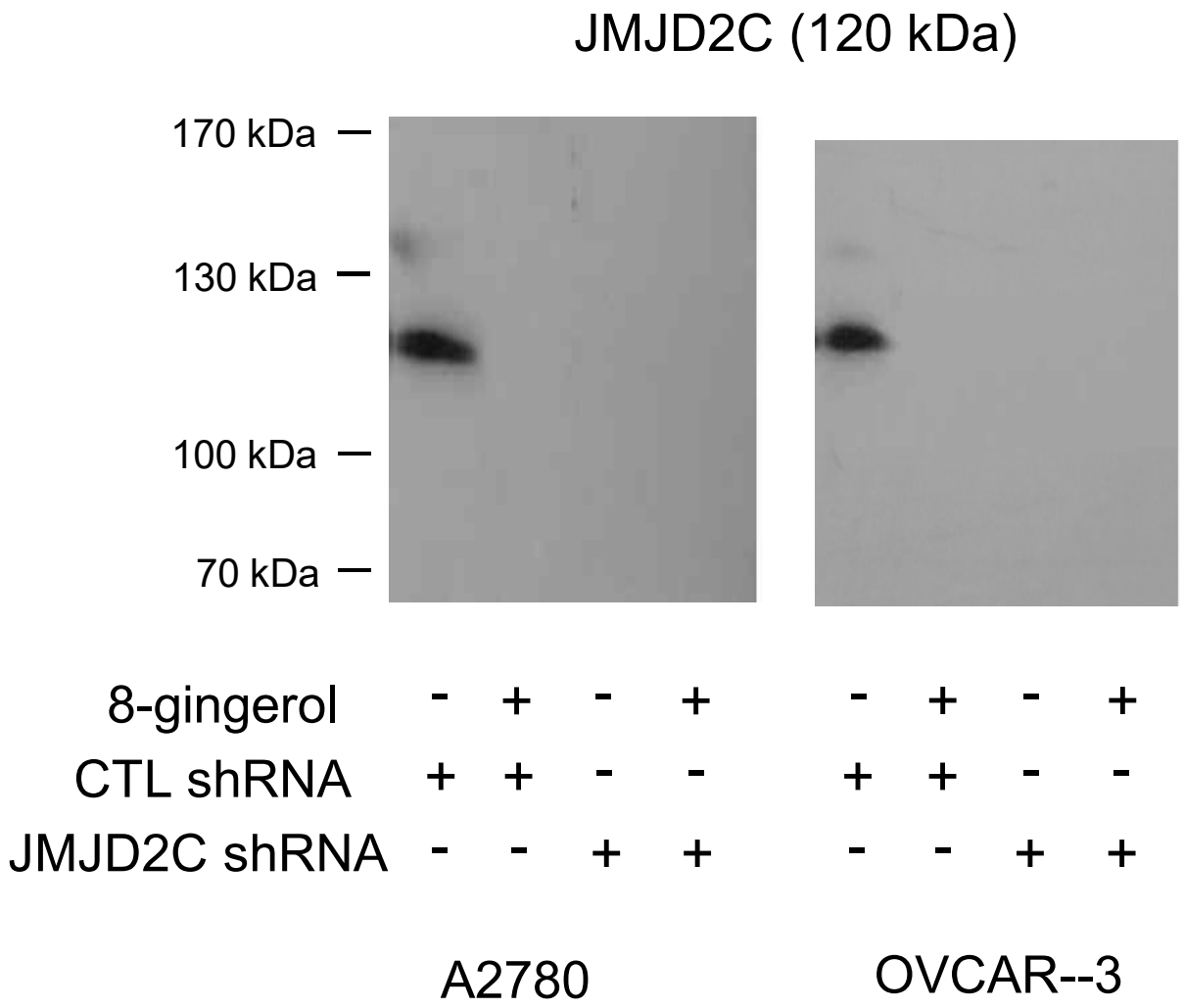

Supplement: Supplementary file 1 [file 12964_2026_3014_MOESM2_ESM.pdf]
