# Supplementary material for: 8-gingerol from Zingiber officinale induces ER stress-mediated ferroptosis and apoptosis in ovarian cancer involving TRPV1 activation
Source: Cell Commun Signal. 2026 Jul 10;24:522. doi: 10.1186/s12964-026-03014-8 (PMC13629130; doi:10.1186/s12964-026-03014-8)
Supplement: Supplementary file 2 [file 12964_2026_3014_MOESM1_ESM.docx]

**Supplementary Figures**

**Fig. 1. Effects of 8-gingerol on mRNA and protein expression levels of inflammatory response markers in** **LPS-treated Raw264.7 and J774.1 cells.** (**A, B)** Quantitative densitometric analysis of western blotting showing protein expression levels of TNF-α, IL-6, and IL-1β in lung tissues and serum samples obtained from LPS- and CLP-mediated mouse models treated with 8-gignerol. β-actin was used as a loading control. * *p* < 0.05; n.s., not significant. All experiments were performed in triplicate. (**C)** Quantitative densitometric analysis of western blotting showing protein expression levels of TNF-α, IL-6, and IL-1β in Raw264.7 and J774.1 cells treated with LPS (1 µg/mL) in the presence or absence of 8-gingerol (0, 25, 50, and 100 µM, 24 hours). β-actin was used as a loading control. * *p* < 0.05; n.s., not significant. All experiments were performed in triplicate.

**Fig. 2. Anti-ovarian tumor effects of 8-gingerol *in vivo* and *in vitro*.** (**A)** Quantitative densitometric analysis of western blotting showing protein expression levels of Bcl-2, p-NF-κB, NF-κB, cleaved caspase-9, and cleaved caspase-3 in A2780 and OVCAR-3 cells treated with 8-gingerol at the indicated time points. β-actin was used as a loading control. All experiments were performed in triplicate. (**B)** Quantitative densitometric analysis of western blotting showing cleaved caspase-3 expression in tumor tissues obtained from mice treated with 8-gingerol (10 and 20 mg/kg). β-actin was used as a loading control. (C**)** Quantitative densitometric analysis of western blotting showing cleaved caspase-3 expression following treatment with Z-VAD-FMK and 8-gingerol. β-actin was used as a loading control. All experiments were performed in triplicate.

**Fig. 3. 8-Gingerol mediates ER stress-associated cell death through intracellular Ca^2+^ release in ovarian cancer cell lines.** (**A)** Quantitative densitometric analysis of western blotting showing protein expression levels of eIF2α, p-PERK, CHOP, GRP78, ATF3, p-eIF2α, and PERK. (**B)** Quantitative densitometric analysis of western blotting showing protein expression levels of p-PERK, PERK, and ATF3 in tumor tissues obtained from mice treated with or without 8-gingerol (10 and 20 mg/kg). (**C)** Quantitative densitometric analysis of western blotting showing protein expression levels of ER stress-associated proteins, including ATF3, cleaved caspase-3, p-PERK, and PERK, in A2780 and OVCAR-3 cells treated with thapsigargin (TG; 3 μM, 24 hours) and 8-gingerol (50 μM, 24 hours). * *p* < 0.05; n.s., not significant. All experiments were performed in triplicate. (**D)** Raw264.7 and J774.1 cells were treated with 8-gingerol (50 µM, 24 hours), LPS (1 µg/mL), or a combination of 8-gingerol and LPS, followed by western blot analysis. were performed. Quantitative densitometric analysis of western blotting showing protein expression levels of ATF3, p-PERK, and PERK is presented. * *p* < 0.05; n.s., not significant. All experiments were performed in triplicate.

**Fig. 4. Targeting ER stress markers suppresses apoptotic cell death in 8-gingerol-treated ovarian cancer cell lines.** (**A)** A2780 and OVCAR-3 cells were transfected with GRP78 siRNA (30 nM, 24 hours) followed by treatment with 8-gingerol (50 μM, 24 hours). Quantitative densitometric analysis of western blotting showing protein expression levels of PERK, ATF3, cleaved caspase-3, p-PERK, and GRP78 is presented. * *p* < 0.05; n.s., not significant. All experiments were performed in triplicate. (**B)** Quantitative densitometric analysis of western blotting showing protein expression levels of cleaved caspase-3, p-PERK, ATF3, and PERK in PERK shRNA knockdown stable cell lines treated with 8-gingerol (50 μM, 24 hours). * *p* < 0.05; n.s., not significant. All experiments were performed in triplicate. (**C)** Quantitative densitometric analysis of western blotting showing protein expression levels of cleaved caspase-9, cleaved caspase-3, CHOP, and PUMA in CHOP shRNA knockdown stable cell lines treated with 8-gingerol (50 μM, 24 hours). * *p* < 0.05; n.s., not significant. All experiments were performed in triplicate. (**D)** Quantitative densitometric analysis of western blotting showing protein expression levels of p-PERK, ATF3, and PERK in PERK shRNA knockdown stable Raw264.7 and J774.1 cells treated with 8-gingerol (50 μM, 24 hours), LPS (1 µg/mL), or a combination of 8-gingerol and. * *p* < 0.05; n.s., not significant. All experiments were performed in triplicate.

**Fig. 5. 8-Gingerol induces apoptotic and ferroptotic cell death through intracellular ROS generation in ovarian cancer cell lines.** (**A)** A2780 and OVCAR-3 cells were treated with NAC (100 μM) or DPI (1 μM) followed by treatment with 8-gingerol (50 μM, 24 hours). Quantitative densitometric analysis of western blotting showing protein expression levels of cleaved caspase-3, p-PERK, ATF3, GPX4, and NOX4 is presented. All experiments were performed in triplicate.

**Fig. 6. 8-Gingerol induces apoptotic and ferroptotic cell death in ovarian cancer cell lines involve the TRPV1-PLCγ-NOX4 axis and associated ER stress signaling.** (**A)** Quantitative densitometric analysis of western blotting showing protein expression levels of TRPV1, PLCγ, p-PLCγ, PKCα, and p-PKCα in A2780 and OVCAR-3 cells were treated with 8-gingerol (50 μM) at the indicated time points. All experiments were performed in triplicate. (**B)** Quantitative densitometric analysis of western blotting showing protein expression levels of TRPV1, PKCα, p-PKCα and NOX4 in TRPV1 shRNA knockdown stable cell lines treated with 8-gingerol (50 μM, 24 hours). * *p* < 0.05; n.s., not significant. All experiments were performed in triplicate. (**C)** Quantitative densitometric analysis of western blotting showing protein expression levels of ATF3, cleaved caspase-3, and NOX4 in NOX4 shRNA knockdown stable cell lines treated with 8-gingerol (50 μM, 24 hours). * *p* < 0.05; n.s., not significant. All experiments were performed in triplicate.

**Fig. 7. 8-Gingerol induces ferroptotic cell death through the SLC7A11-GPX4-NRF2-HO-1 axis in ovarian cancer cells.** (**A)** Quantitative densitometric analysis of western blotting showing protein expression levels of SLC7A11 and GPX4 in tumor tissues obtained from mice treated with or without 8-gingerol (10 and 20 mg/kg). All experiments were performed in triplicate. (**B)** A2780 and OVCAR-3 cells were treated with 8-gingerol (50 μM) at the indicated time points and analyzed by western blotting. Quantitative densitometric analysis of western blotting showing protein expression levels of SLC7A11, GPX4, NRF2, KEAP1, and HO-1 is presented. * *p* < 0.05. All experiments were performed in triplicate. (**C)** A2780 and OVCAR-3 cells were treated with Liproxstatin-1 (ferroptosis inhibitor, 2 μM, 24 hours) followed by 8-gingerol (50 μM, 24 hours) treatment. Quantitative densitometric analysis of western blotting showing protein expression levels of cleaved caspase-3, SLC7A11, NRF2, GPX4, and HO-1 is presented. All experiments were performed in triplicate. (**D)** A2780 and OVCAR-3 cells were treated with Erastin (ferroptosis inducer, 10 μM, 24 hours) followed by 8-gingerol (50 μM, 24 hours) treatment. Quantitative densitometric analysis of western blotting showing protein expression levels of SLC7A11, NRF2, GPX4, and HO-1 is presented. All experiments were performed in triplicate.

**Fig. 8. 8-Gingerol-mediated ferroptosis is regulated through the ATF3-JMJD2C-SLC7A11-GPX4 signaling pathway in ovarian cancer cells.** (**A)** Quantitative densitometric analysis of western blotting showing protein expression levels of H3K4me1, H3K4me2, H3K4me3, H3K9me1, H3K9me2, H3K9me3, H3K27me1, H3K27me2, H3K27me3, and H3K36me1, H3K36me2, H3K36me3 in nuclear fractions isolated from A2780 and OVCAR-3 cells treated with 8-gingerol (50 μM, 24 hours). H3 was used as a loading control. (**B)** Quantitative densitometric analysis of western blotting showing protein expression levels of JMJD2C, H3K9me3, and H3K36me3 in A2780 and OVCAR-3 cells treated with 8-gingerol at the indicated time points (0, 8, 16, and 24 hours). H3 was used as a loading control. (**C)** A2780 and OVCAR-3 cells were treated with 8-gingerol (50 μM, 24 hours) and SD-70 (JMJD2C inhibitor; 30 μM, 24 hours). Quantitative densitometric analysis of western blotting showing protein expression levels of ATF3, JMJD2C, SLC7A11, and GPX4 is presented. β-actin was used as a loading control. * *p* < 0.05; n.s., not significant. (**D)** JMJD2C knockdown stable A2780 and OVCAR-3 cells were treated in the presence or absence of 8-gingerol (50 μM, 24 hours). Quantitative densitometric analysis of western blotting showing protein expression levels of JMJD2C, SLC7A11, and GPX4 is presented. β-actin was used as a loading control. * *p* < 0.05; n.s., not significant.
